# Community end user perceptions of hessian fabric transfluthrin vapour emanators for protecting against mosquitoes under conditions of routine use in Port-au-Prince, Haiti

**DOI:** 10.1371/journal.pone.0300368

**Published:** 2024-07-10

**Authors:** Obrillant Damus, Chicoye Supreme, Jean-Frantz Lemoine, Christian Raccurt, Justin McBeath, Sheila B. Ogoma, Vincent Corbel, Katherine Andrinopoulos, Daniel Impoinvil, Gerry F. Killeen, Cyrille Czeher

**Affiliations:** 1 Université Quisqueya, Port-au-Prince, Republic of Haiti; 2 Programme National de Contrôle de la Malaria, Ministère de la Santé Publique et de la Population, Port-au-Prince, Republic of Haiti; 3 Envu UK Ltd, Milton, Cambridge, United Kingdom; 4 Abt Associates, Nairobi, Kenya; 5 Institut de Recherche pour le Developpement, Infectious Diseases and Vectors-Ecology, Genetics, Evolution and Control (MIVEGEC) Unit, University of Montpellier, Montpellier, France; 6 Laboratório de Fisiologia e Controle de Artrópodes Vetores (Laficave), Instituto Oswaldo Cruz (IOC), Fundação Oswaldo Cruz (FIOCRUZ), Rio de Janeiro-RJ, Brazil; 7 Tulane University School of Public Health and Tropical Medicine, New Orleans, Louisiana, United States of America; 8 Entomology Division, Centers for Disease Control and Prevention, Atlanta, GA, United States of America; 9 Environmental Health and Ecological Sciences Department, Ifakara Health Institute, Morogoro, United Republic of Tanzania; 10 Department of Vector Biology, Liverpool School of Tropical Medicine, Liverpool, United Kingdom; 11 School of Biological Earth & Environmental Sciences, Environmental Research Institute, University College Cork, Cork, Republic of Ireland; 12 Entente Interdépartementale pour la Démoustication du Littoral Méditerranéen (EID Méditerranée), Montpellier, France; University of Glasgow College of Medical Veterinary and Life Sciences, UNITED KINGDOM

## Abstract

**Background:**

A treated fabric device for emanating the volatile pyrethroid transfluthrin was recently developed in Tanzania that protected against night-biting *Anopheles* and *Culex* mosquitoes for several months. Here perceptions of community end users provided with such transfluthrin emanators, primarily intended to protect them against day-active *Aedes* vectors of human arboviruses that often attack people outdoors, were assessed in Port-au-Prince, Haiti.

**Methods:**

Following the distribution of transfluthrin emanators to participating households in poor-to-middle class urban neighbourhoods, questionnaire surveys and in-depth interviews of end-user households were supplemented with conventional and *Photovoice*-based focus group discussions. Observations were assessed synthetically to evaluate user perceptions of protection and acceptability, and to solicit advice for improving and promoting them in the future.

**Results:**

Many participants viewed emanators positively and several outlined various advantages over current alternatives, although some expressed concerns about smell, health hazards, bulkiness, unattractiveness and future cost. Most participants expressed moderate to high satisfaction with protection against mosquitoes, especially indoors. Protection against other arthropod pests was also commonly reported, although satisfaction levels were highly variable. Diverse use practices were reported, some of which probably targeted nocturnal *Culex* resting indoors, rather than *Aedes* attacking them outdoors during daylight hours. Perceived durability of protection varied: While many participants noted some slow loss over months, others noted rapid decline within days. A few participants specifically attributed efficacy loss to outdoor use and exposure to wind or moisture. Many expressed stringent expectations of satisfactory protection levels, with even a single mosquito bite considered unsatisfactory. Some participants considered emanators superior to fans, bedsheets, sprays and coils, but it is concerning that several preferred them to bed nets and consequently stopped using the latter.

**Conclusions:**

The perspectives shared by Haitian end-users are consistent with those from similar studies in Brazil and recent epidemiological evidence from Peru that other transfluthrin emanator products can protect against arbovirus infection. While these encouraging sociological observations contrast starkly with evidence of essentially negligible effects upon *Aedes* landing rates from parallel entomological assessments across Haiti, Tanzania, Brazil and Peru, no other reason to doubt the generally encouraging views expressed herein by Haitian end users could be identified.

## Background

The *Aedes* (*Stegomia*) mosquitoes that mediate most transmission of Dengue, Chikungunya, Yellow Fever and Zika viruses often attack people during daylight hours when they are awake and active, often outdoors, so there are limits to how much protection may be reasonably expected from indoor interventions [1, 2] like insecticidal bed nets that protect sleeping spaces [3] or even insecticidal screens that protect entire houses [4]. However, a recent large-scale trial of a spatial repellent product that emanates vapour of the volatile pyrethroid transfluthrin, which was designed to protect users in outdoor spaces and open structures, successfully demonstrated that such devices may reduce incidence of arboviral infections [5]. Unfortunately, these devices and other existing repellent products currently available on the market only protect against mosquitoes for hours, days or weeks per application or dispensing dose, so they may be too expensive and impractical for continuous, indefinite use in low-income countries like Haiti [1, 2, 6], and some formulations may even be hazardous [7, 8].

More encouragingly, a low-technology transfluthrin emanator was recently developed in Tanzania that slowly and passively releases vapour of the spatial repellent transfluthrin under ambient temperature conditions without any electricity or other power source [9], providing >90% protection for >4 months against nocturnal *Anopheles* and *Culex* spp. vectors of malaria, filariasis, and several arboviruses in urban Dar es Salaam [10]. In a subsequent study in rural Tanzania, >75% protection was sustained over 6 months and at least some degree of protection persisted over 2.5 years without any evidence of diversion of mosquitoes to unprotected non-users nearby [11]. Also, equivalent efficacy was achieved over 6 months with a 10-fold lower transfluthrin dosage, which costs only €0.10 and releases vapour concentrations of only 0.00013 mg/m^3^ [11], comparing well with its registered acceptable exposure concentration of 0.5 mg/m^3^ [12]. While the initial prototype required that a long hessian strip was suspended on four poles placed around the user in an open outdoor space, a more compact and practical format has now been developed that is completely mobile and can be conveniently placed anywhere the user chooses to [10, 11].

If these transfluthrin emanator devices were to prove as effective against day-biting *Aedes* as they are against night-biting *Culex* and *Anopheles*, they could offer simultaneous, broad-spectrum daytime protection against Dengue, Chikungunya, Yellow Fever and Zika. However, the effectiveness of any such repellent technology designed to afford personal protection measure against vector mosquitoes depends not only upon its efficacy and availability but also upon its uptake and use, which depends in turn upon positive perceptions among potential beneficiary communities [13–25]. The following series of mixed-method social science investigations were therefore carried out in Port-au-Prince, Haiti, to evaluate the perceived protective effectiveness and user acceptability of transfluthrin emanators. This study also aimed to survey end user perspectives of potential pitfalls, opportunities, and optimal communication tactics for possible programmatic scale up in the future. Parallel experimentally controlled entomological studies to measure the extent of protective efficacy that these same transfluthrin emanators provided to households in the same communities against wild *Aedes* mosquitoes under outdoor field conditions, are reported elsewhere in a complementary manuscript [26].

## Methods

### Field site and study design

Overall, this mixed-method study began with the distribution of transfluthrin emanators to participating households in urban Port-au-Prince, following which brief semi-quantitative questionnaire surveys of overall end-user satisfaction with the protective efficacy of these devices were supplemented with semi-structured qualitative in-depth interviews, conventional focus group discussions (FGDs) and FGDs centred around *Photovoice* (PV) approaches [13, 27–30] to documenting community expression through participatory photography. The recorded observations were then assessed synthetically, using a content analysis approach [31] to evaluate user perceptions of protection and acceptability, and to solicit advice for improving and promoting them in the future.

**Fig 1:**
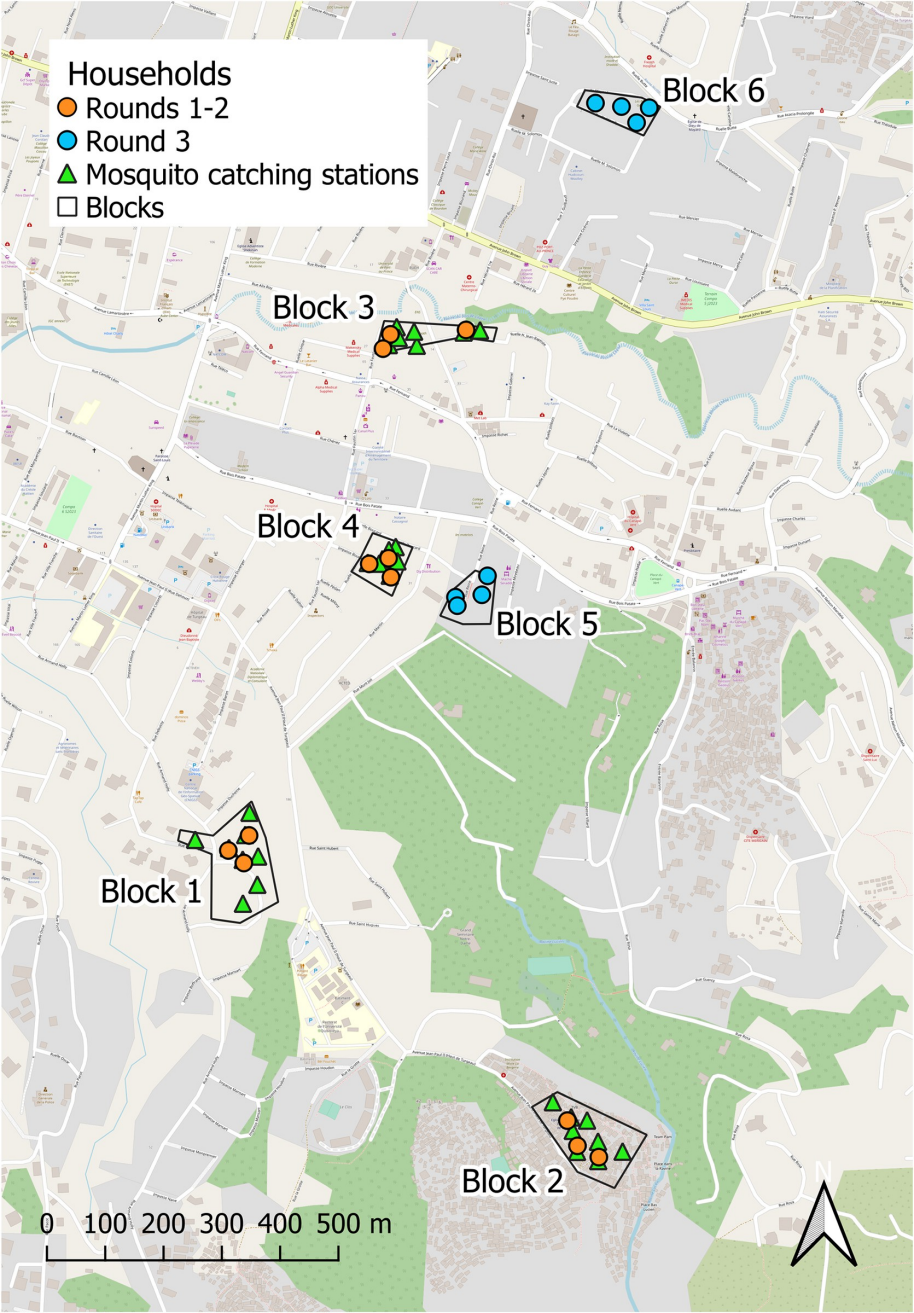
A map of the study site in the Haut-Turgeau neighbourhood in the city of Port-au-Prince, Haiti. Note how the social science assessments of user-perceived efficacy reported herein were conducted in the same four clusters of three households as the parallel entomological assessments reported elsewhere were centred around [26] for the first two assessment rounds, but in two geographically separate clusters of four households for the third assessment round. As explained in detail elsewhere [26], the three different assessment rounds otherwise differed only in that the emanators were treated with different formulations of transfluthrin and slightly different experimental procedures were used to assess their efficacy in entomological terms (Fig 2). This map was produced with *QGIS*® version 3.28.9 open source software, using a base map obtained from *OpenStreetMap*® under the Open Database License.

**Fig 2:**
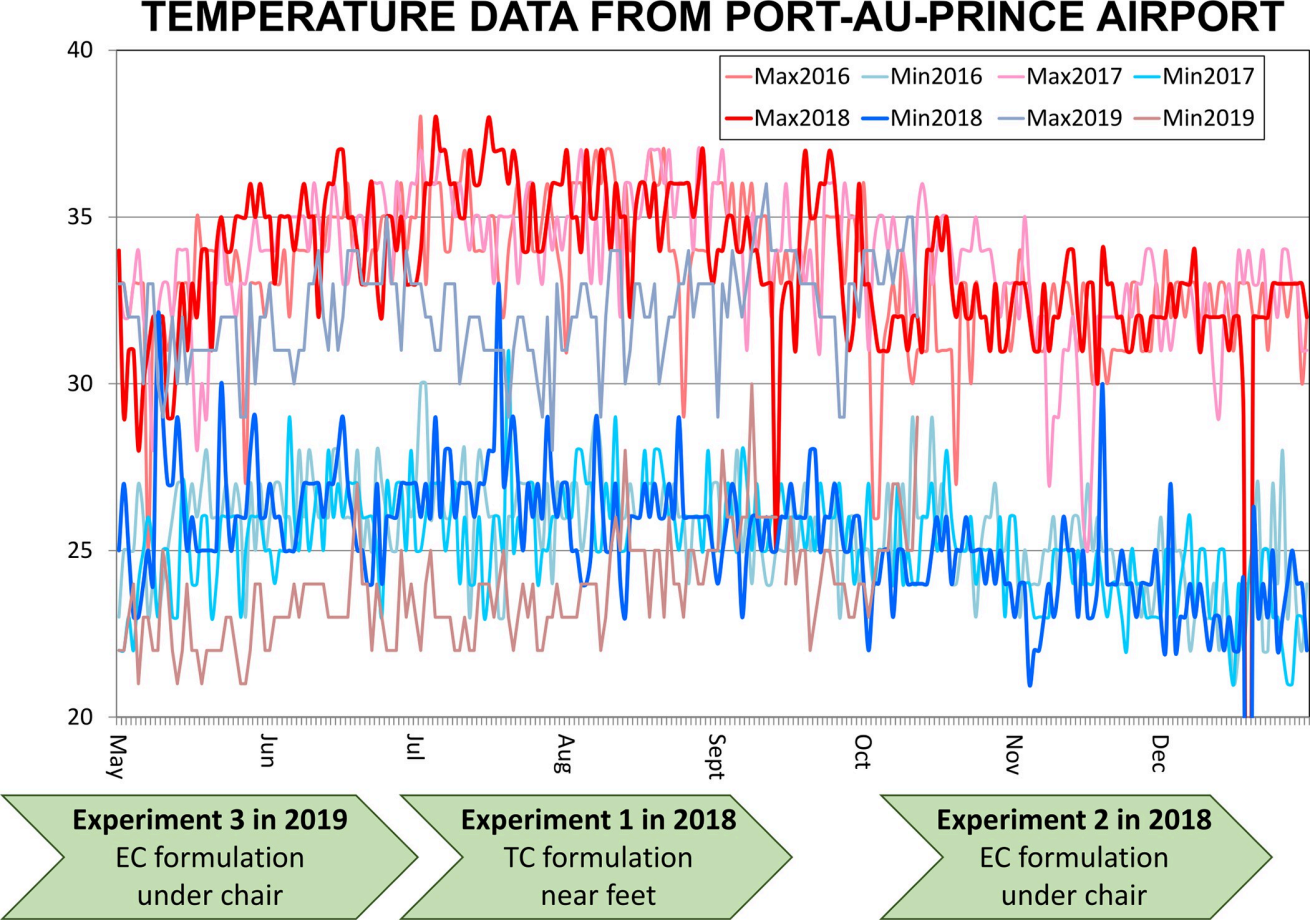
A schematic outline of how the three entomological efficacy assessment experiments described herein fitted into typical seasonal temperature trends in Port-au-Prince, Haiti. This schematic also illustrates how these three experiments differed from each other in terms of placement of the emanators relative to the human user, as well as the choice of transfluthrin formulation (Emulsifiable concentrate (EC) versus technical concentrate (TC)) used to treat them [26].

Panels of hessian fabric were treated with transfluthrin, wrapped in a protective wire mesh cover and folded into a zig-zag shaped self-standing emanator devices (See *Preparation of transfluthrin emanator devices*) before being distributed to participating households in poor-to-middle class urban neighbourhoods of Haut-Turgeau, in the city of Port-au-Prince, Haiti (Fig 1), where high population density is associated with intense poverty and deprivation [32]. In addition to their generally low socio-economic status, these specific neighbourhoods were chosen because of their proximity to Université Quisqueya and their ready accessibility via main roads, enabling the investigator team to leave the area promptly in the event of unforeseen civil unrest. Note that while their subsequent evaluation in terms of their perceived effectiveness by these community end users is reported herein, this set of social science investigations was integrated with a complementary set of quantitative entomological assessment experiments run in parallel (Fig 2), which directly measured the protective efficacy of these emanators in terms of outdoor landing rates of *Aedes* mosquitoes upon humans [26].

Each of the household clusters illustrated in Fig 1 consisted of the first three consenting households (See *Ethical considerations*) who could be identified by door-to-door convenience sampling, starting from a central point within that neighbourhood. The ages of participants ranged from 19 to 74, and it was informally observed that the vast majority of participants belonged to low-income socio-economic groups. While they lived in neighbourhoods where all social classes were observed, the homes of middle-class and wealthy people were separated from those with lower income by high walls, iron gates and barbed wire. Although an attempt was made to recruit participants from a wealthier neighbourhood in Haut-Turgeau than those illustrated in Fig 1, this effort was unsuccessful because such houses and their occupants are difficult to access and these households tended to be busier, with less availability during normal working hours. In contrast, most adult members of lower-income households, living in more modest housing, were unemployed and were generally more willing to participate in the study. However, no formal records were kept regarding who declined to participate in the study, where they lived or what their socio-economic status was.

All engagements with community members in these neighbourhoods of Port-au-Prince, including collection of the formal social science data reported herein, were carried out in fluent Haitian Creole by resident team members for whom this was their first language. These interviews and discussions were carried out privately, in absence of any community members other than the participants themselves, at their place of residence or at a suitable community venue nearby. The conversations relevant to the formal informed consent and data collection processes described below were facilitated by the male first-named author (OD), an experienced and fully trained social scientist who holds a PhD and worked on behalf of Université Quisqueya at the time, which might have unduly influenced the responses of participants. Furthermore, as for all the other investigators, the potential for further research funding in the event of these transfluthrin emanators proving effective and well-accepted by end-users represented a clear competing interest for this key lead investigator, which should be borne in mind regarding interpretation of the results. Note, however, that no personal or professional relationship existed between the investigators and these recruited households before initiation of the study, and no background information other than that contained in the participant information sheet was communicated to them except through bespoke responses to sundry queries on their part. Although no strict time limits were placed on the duration of IDIs, FGDs or PV-FGDs, the discussion guides were used to keep conversations in scope, and data saturation criteria were explained and applied to curtail excessive discussion of any given topic with any given individual or focus group. While the survey tools and procedures used to carry out the social science investigations described below drew on a wide range of directly relevant literature [14–25], it relied particularly upon the rationale and past experiences of the investigators with a similar study in urban Dar es Salaam, Tanzania [13] that also included PV methodology [27–30].

While the original intention had been to carry out the quantitative entomological assessments reported elsewhere [26] and the qualitative assessments of community end user perceptions reported herein only once, the former yielded no evidence of significant protection in terms of reductions of human landing rates. Therefore, both types of evaluation were repeated twice, with minor procedural variations to determine whether changing the transfluthrin formulation used or the position of the emanator relative to the user improved the levels of efficacy observed based on quantitative entomological indicators [26].

Although none of these three repetitions of the quantitative entomological assessment yielded evidence of significant protection [26], the first and second rounds of social science surveys reported herein yielded surprisingly encouraging levels of user satisfaction (See *Results*). Given that the social science investigations in these first two assessment rounds for which participants were carried out in the same household clusters as the entomological evaluations (Fig 1), during which householders were compensated generously for their time and inconvenience (See *Ethical Considerations*), the potential for soliciting biased perspectives was considered to be a significant risk. Correspondingly, the third round of social science assessments of end-user perspectives were carried out in geographically distinct household clusters from those used for all the entomological assessments (Fig 1), to prevent community perspectives being unduly influenced by competing financial interests or by discussions with the entomological research team during the regular monitoring visits necessitated by those procedures.

Correspondingly, semi-quantitative questionnaires and in-depth interviews (IDIs) were conducted twice in blocks 1 to 4, in September (Experimental assessment round 1) and November 2018 (Round 2), and once in the blocks 5 and 6 in May 2019 (round 3), as illustrated in Fig 1. However, an extended period of severe civil unrest during the first round of assessments, made it imprudent to attempt focus group discussions (FGDs), PV investigations or PV focus group discussions (PV-FGD). Also, the disposable cameras provided to the household members responsible for the PV component during the second round failed to save most of the pictures, and only few were usable after development and printing. This precluded completion of the planned PV group discussions in assessment round 2. Consequently, compact digital cameras were provided to households for the third round of assessment, which enabled completion of the PV-FGDs and selection of the best pictures in May 2019. Otherwise, some smaller deviations from the following protocols occurred for various practical reasons (eg. households withdrawing from the study, or some housing clusters being temporarily inaccessible during subsequent periods of civil unrest that were briefer than that which occurred during the first assessment round), but these were all relatively minor and had no obvious implications for the interpretation of the results. The overall number of individual householders who participated in each type of survey for each assessment round are detailed in Table 1.

**Table 1:** Summary of the various complementary social science methodologies applied and numbers of participants in each assessment round.

Methodology	Number of community participants in each round of assessment		
	Round 1	Round 2	Round 3
Brief questionnaire	14	18	8
In-depth interviews (IDI)	10	26	8
Focus Group Discussions (FGDs)	Not implemented	24	12
Photovoice (PV)	Not implemented	12	6
Photovoice Focus Group Discussions (PV-FGD)	Not implemented	Not implemented	5

### Preparation of transfluthrin emanator devices

Panels of hessian fabric, each measuring 70 × 40 cm, were made from jute rolls bought locally and then washed, dried and treated with 99% technical grade transfluthrin (Bayer AG, Environmental Sciences at the time, now trading as Envu AG, Germany). For each hessian panel, a mixture containing 3g of transfluthrin, 90ml of locally available liquid dish washing detergent (Apta Vaisselle, Intermarché) and 400ml of water was prepared and soaked into a the panel as evenly as possible, as previously described [11]. Control panels were also soaked into equal mixture of water and detergent but without transfluthrin. Each panel was then left to dry at room temperature indoors, for between two days and a week, and then wrapped within a wire-mesh to form a folded, self-supporting zig-zag-shaped panel [26], essential identical to that similarly evaluated against *Ae*. *aegypti* in Tanzania [33]. The wire-mesh cover was designed to prevent dermal contact of participants and researchers with the treated hessian panels.

### Provision of transfluthrin emanators and usage guidance to households

Each of the four participating households per housing cluster was provided with 2 freshly prepared transfluthrin emanators at the outset of an experiment, to be used freely by the householders following advisory discussions with the research team on how to safely and effectively deploy them. All treated emanators provided to households were taken back from them for entomological evaluations of their efficacy for only 8 days every two months. For the remainder of each 2-month evaluation cycle, when they were not being assessed through controlled entomological experiments, the emanators were used freely within the bounds of the safety instructions provided. Participants were actively encouraged to use them creatively, in whatever way they perceived to be optimal in terms of convenience and protection against mosquito bites, so long as they did not open the protective holder or use them in any way that would allow direct physical contact with the treated fabric inside it. As an illustrative example, the research team explained how one investigator placed such a device beside the front door of his house at night to prevent house entry by *Culex* mosquitoes [34].

### Longitudinal *Photovoice* and questionnaire surveys of end-user households

Each participating household was asked to record what they perceived to be the most and least effective transfluthrin emanator use practices with either disposable cameras (First set of surveys) or with digital cameras (Third assessment round) provided specifically for this purpose by the study team, similarly to our previous PV [27–30] surveys in Tanzania [13]. One participant in the photographic component of this study was recruited per household, who was responsible for taking photos on behalf of the entire household. Before using these cameras, participants took part in a short training meeting to explain the subject matter of the survey, the principles of personal data confidentiality protection, and outline acceptable ways of using the camera without compromising the safety, privacy, or other rights of individuals or the community as a whole [13].

Each household was visited once every two months, at which point the emanators were collected for experimental entomological assessments of efficacy for 8 days [26] and householders were surveyed with a very brief semi-structured questionnaire to assess their level of satisfaction with the perceived level of protection against mosquito bites. All consenting adult household members were surveyed on each occasion through such IDIs. Perceived satisfaction was recorded numerically as a graded scale from 0 to 5, with separate scores recorded for indoor and outdoor exposure. At the start of each visit to the block, any fully used disposable cameras or digital photos taken since the last visit were collected and developed or printed. One of printed hard copy duplicates was returned to the household within a few days [13], while the other was retained by the research team for use in the PV-FGDs described below.

### Conventional focus group discussions

At the end of each of the two sets of linked social science and entomological studies, two FGDs were conducted, with one voluntary participant of each gender from each of the participating households across all blocks per group. The participants in the focus group discussions included adult males and females in separate groups, and these four groups were interviewed separately to enhance participation. These semi-structured discussions were conducted using a brief topic guide to obtain thematic insights into how they perceived the value, advantages and disadvantages, affordability and practicality of these devices.

At the end of these discussions, the interviewers also canvassed the participants for advice on how best to take this technology forward through further possible product modifications, programmatic evaluation and operational research. For each focus group discussion, a facilitator coordinated discussion while one of three field assistants (All female) responsible for the audio recordings and subsequent transcription took notes on expressed verbal and non-verbal forms of communication.

### Photovoice-based focus group discussions

Once all the pictures were developed or printed and returned to the photographer participants, they were engaged in a two-stage process of participatory analysis: selecting photographs for discussion and then contextualizing or storytelling. In the first stage, developed pictures were given back to photographers at the end of the study, each of whom was given approximately one week to select what he/she considered to be his/her 10 best or most significant photographs. By selecting photographs for discussion, participants were enabled to lead the overall direction of subsequent PV-FGDs [29, 30]. The second stage consisted of contextualizing or telling stories about what each photograph meant to the photographer, during the PV-FGD. Each participant displayed his/her photographs on a table, introduced them to the group, narrated the meaning of his/her photographs, and explained his/her interpretation of the images. These PV-FGDs were conducted informally but based on an adapted version of the SHOWeD model [29, 30]. At this stage of the discussion, each photographer identified different themes that emerged after re-examining the contents of their photographs and explained where, when and why they took them. This was followed by a more specific discussion of the advantages, disadvantages and limitations of these devices, factors influencing their use, and ideas for improving the devices themselves or for optimal delivery and maintenance in the future. At the end of the discussion, the participants selected the 10 best pictures out of all the photographs taken by the group collectively. As for the conventional FGDs described above, a facilitator coordinated the discussions while one of three field assistants (All female) responsible for the audio recordings and subsequent transcription took notes on expressed verbal and non-verbal forms of communication.

### Data analysis

The semi-quantitative data collected, in the form of rated categorical levels of satisfaction with the protection provided by transfluthrin emanators, in both indoor and outdoor environments, and against both mosquitoes specifically and other pests more generally, were primarily analysed graphically by comparing the distributions of these recorded perceptions but Wilcoxon non-parametric rank sum tests were also used to compare satisfaction levels with indoor use practices versus outdoor use practices.

All of the qualitative IDIs, FGDs and PV-FGDs were conducted in Haitian Creole and digital audio recordings were made, which were subsequently transcribed verbatim (with identifiers removed) as Microsoft Word® documents [13, 35, 36]. Unfortunately, it proved practically infeasible to obtain comments and corrections with respect to these lengthy transcripts. A content analysis approach was then applied to this data by a single investigator (OD), with the transcripts analysed according to the classical inductive methods of comprehensive sociology with regard to the perceptions of the participants [31]. The data analysis process consisted of identifying themes and sub-themes in the material *post hoc*, by manually splitting (identification and coding of meaning units) and categorizing the material (grouping semantic units under various categories) but excluding observations from two participants who exhibited overt signs of strong bias, such as repeating some wording from the informed consent forms verbatim.

Selected quotes of particular relevance to interpretation were translated into English, a subset of which are presented in the *Results* section. Note that whenever similar perspectives emerged from two or more surveys, because of our concerns about potential competing interests among participants in the first and second rounds of assessment in 2018 (See the final paragraph of *Ethical considerations*), quotes from the third and final round of assessment in 2019 (Fig 2) were given priority for presentation in the *Results* section. Reassuringly, this third, hopefully less bias-prone, round of investigations yielded observations that were largely consistent with those obtained previously from the first and second assessment rounds, in which both types of investigations were conducted in the same household clusters (Fig 1). The results of all three rounds of sociological investigations are therefore presented together, synthesized as follows in terms of common themes that emerged across multiple survey formats and rounds.

### Ethical considerations

The procedures for this study were reviewed and approved by the Comité Nationale de Bioéthique of the Ministère de la Santé Publique et de la Population of the Republic of Haiti (Ref. 1718–42) and the Research Ethics Committee of the Liverpool School of Tropical Medicine in the United Kingdom (Ref. 16–037).

At the outset of the study, the concentrations of tranfluthrin vapour released by these emanator devices had previously been measured [11] as being less than one in a thousand times that considered an acceptable exposure concentration in the European Union [12]. Inhalation exposure to transfluthrin was therefore considered to present negligible risk to participants at the outset of this study.

Participants in the study were recruited between May 2018 and February 2019. Minor risks to privacy and security are associated with disseminating photographs of the exterior or interior of participants’ houses or their contents. Furthermore, minor risks to privacy and security may be incurred by presenting the facial features or other identifiable personal information in such published photographs. These risks were therefore mitigated by obtaining written permission before any specific photograph was published or shared with anyone other than the investigators, with any facial features or other identifiable personal information masked out if requested by the participant. At the request of the participants, we also masked out any personally identifiable or sensitive elements of the photographs, such as their belongings or security precautions. Correspondingly, no personally identifiable data or images are presented in this publication. The only other personal information to be collected from any participants were their name, age and gender, all of which were kept confidentially by the investigators who knew the individuals, with original hard copies stored in locked filing cabinets while all electronic files containing this data, as well as the computers it was stored on, were encrypted and password protected.

All participants in this study were fully informed of these potential risks and benefits of participation in the study, as well as their freedom to withdraw at any stage, and were given every opportunity to ask any questions they had before informed consent was documented in writing. All informed consent and data collection processes were carried out through face-to-face, in-person engagements in Haitian Creole, using the relevant participant information sheets, informed consent forms, questionnaires and discussion topic guides as a basis for oral explanations and discussions. All of these documents were translated into French Haitian Creole, pilot tested and then reviewed and approved by the Comité Nationale de Bioéthique of the Ministère de la Santé Publique et de la Population before they were used for this purpose. All participants were provided with detailed oral feedback on the finding of the studies through group meetings at local neighbourhood venues in each housing cluster. At these meetings, quotes of particular interest that were considered for inclusion in the *Results* section of this manuscript were highlighted, together with the edited forms of various photographs that were considered for inclusion in the figures.

While none of the participants in these social science investigations were remunerated in any way, participants in the mosquito landing catches required for the quantitative entomological assessments reported elsewhere [26] were compensated for their time and inconvenience caused. These MET operators were remunerated at a rate of $15 per day that had been standardized across all PNCM activities, to strike a balance between being enough to provide fair compensation for time and discomfort, without inducing volunteers to participate despite any reservations they may have. Nevertheless, this represented a significant amount of money in this low-income context, raising the possibility that community perspectives might be unduly influenced by competing financial interests and/or discussions with the entomological research team during the regular visits necessitated by those procedures [26]. For the third and final round assessments, the social science investigations reported herein and the and entomological reported elsewhere [26] were completely separated and carried out in distinct housing clusters (Fig 1).

## Results

Overall, the perspectives narrated by the participants could be categorized into 10 thematic content categories, from within which distinct, sometimes contradictory, sub-themes emerged (Table 2).

**Table 2:** Distinct content categories identified within the transcripts of the qualitative in-depth interview (IDI), conventional focus group discussions (FGDs) and *Photovoice* (PV) focus group discussions (PV-FGD).

Content categories and concepts	Prevalence (n [Proportion])		
	IDI	FGD	PV-FGD
	(N = 44)	(N = 36)	(N = 5)
*1. Pre-existing knowledge and practices regarding mosquitoes and relevant protection methods*.			
• Mosquito biting nuisance is irritating, annoying and disruptive.	41 [100%]	14 [39%]	2 [40%]
• Mosquito bites cause several diseases, including dengue, Zika and malaria.	14 [34%]	9 [25%]	0 [0%]
• Physical protection with bednets and bed sheets commonly used but unsatisfactory.	6 [15%]	5 [14%]	0 [0%]
• Fans also commonly used but can cause pulmonary illnesses.	2 [5%]	0 [0%]	0 [0%]
• Insecticidal sprays and repellent coils commonly used but expensive, unpleasant and hazardous.	6 [5%]	12 [33%]	0 [0%]
*2. Protective efficacy of transfluthrin emanators against mosquitoes*.			
• Emanators generally more efficacious than existing measures.	19 [46%]	6 [17%]	1 [20%]
• Emanators more efficacious and consistent indoors, away from wind, sunlight and moisture.	5 [12%]	2 [6%]	2 [40%]
• Staying close to emanators improves their efficacy.	21 [51%]	4 [11%]	2 [40%]
• Diverse emanator use practices associated with all common household activities indoors and outdoors.	26 [63%]	16 [44%]	5 [100%]
• Emanators protect family members and improve several aspects of the household routine.	27 [66%]	10 [28%]	3 [60%]
*3*. *Protective efficacy of transfluthrin emanators against other insect pests*			
• Efficacious against flies and cockroaches, and perhaps also millipedes and ants.	6 [15%]	2 [6%]	1 [20%]
• As for mosquitoes, protection also lower and more variable outdoors.	13 [32%]	8 [22%]	2 [40%]
*4*. *Durability of the efficacy of transfluthrin emanators*.			
• Emanator efficacy noted by some to decline within weeks or even days.	16 [39%]	7 [19%]	0 [0%]
• Persistence of efficacy related to temperature, wind and moisture, especially outdoors.	3 [7%]	2 [6%]	0 [0%]
• Stringent protection expected-even one mosquito bite a failing of emanators.	20 [49%]	4 [11%]	0 [0%]
*5*. *Advantages of transfluthrin emanators relative to existing protection measures*.			
• More convenient and comfortable to use than bed sheets, bednets, fans and electrical mosquito control devices.	0 [0%]	1 [3%]	0 [0%]
• Lack of odour or irritation a major advantage over repellent coils and creams and even bednets.	3 [7%]	3 [8%]	0 [0%]
• Free provision of emanators reduced household expenditure on pre-existing alternatives.	10 [24%]	6 [17%]	1 [20.0%]
*6*. *Disadvantages and risks of using transfluthrin emanators*.			
• Limited durability of efficacy noted by some.	16 [39%]	8 [22%]	0 [0%]
• Sharp ends of wire mesh cover prickly.	4 [10%]	0 [0%]	0 [0%]
• Need to prevent children accessing or touching the emanator.	3 [7%]	0 [0%]	0 [0%]
• Potentially harmful to health.	3 [7%]	1 [3%]	0 [0%]
• Bulky and awkward.	4 [10%]	0 [0%]	0 [0%]
• Unattractive appearance.	2 [5%]	1 [3%]	0 [0%]
*7*. *Smell of the emanator and its association with efficacy or health risks*.			
• Generally considered odourless.	3 [7%]	0 [0%]	0 [0%]
• Any odour noted associated with protective efficacy or unpleasant smell and health risks.	3 [7%]	2 [6%]	0 [0%]
*8*. *Potential role for transfluthrin emanators in strengthening links within the community*.			
• Emanators may be shared between family members and neighbouring families.	12 [29%]	5 [14%]	0 [0%]
• A collective good that may contribute to both individual and collective wellbeing.	37 [90%]	9 [25%]	1 [20%]
• Full community-wide coverage with more devices per household needed to maximize equity and impact upon mosquitoes.	12 [29%]	6 [17%]	2 [40%]
• Willingness to promote scale up if made widely available after the study.	7 [17%]	2 [6%]	1 [20%]
*9*. *Ideas for improving and promoting use of transfluthrin emanators*.			
• Improving the aesthetic appearance and ergonomic practicality devices	3 [7%]	2 [6%]	0 [0%]
• Increase dosage of active ingredients to maximize protection and ideally kill mosquitoes.	7 [17%]	4 [11%]	1 [20%]
• Monthly retreatment to sustain efficacy levels.	1 [2%]	0 [0%]	0 [0%]
• Suitable for community-based promotion.	12 [29%]	5 [14%]	1 [20%]
• Commercialize and make available for sale at an affordable cost.	1 [2%]	0 [0%]	0 [0%]
*10*. *Enhance effects of transfluthrin emanators with complementary changes in human behaviour*.			
• No protection measure is perfect, including transfluthrin emanators.	4 [10%]	8 [14%]	0 [0%]
• Importance of community-based environmental management as a complementary intervention.	2 [5%]	0 [0%]	0 [0%]

### Pre-existing knowledge, attitudes and practices regarding mosquitoes and relevant protection methods

Overall, *a priori* perspectives of the participants about mosquitoes were understandably negative and they also expressed considerable dissatisfaction with existing options for protecting themselves against them. Participants used a variety of mosquito bite prevention methods before transfluthrin emanators were introduced to their households. Broadly speaking, the households used four types of mosquito control products: natural repellent products, chemical insecticides and repellents, electrical mosquito control products, and physical interventions such as clothing, fans and bed nets, although many of the latter may have been treated with pyrethroid insecticides. Physical protection measures like bed nets, bed sheets and fans were considered to have several limitations in terms of protection level and practicality:

Quote 1 (Assessment round 1, IDI, Male): *“We used a lot of things against mosquitoes*. *An example is the fan*. *You need access to electricity to be able to use it*. *However*, *we live in a country where electricity is a scarce commodity*. *We waited for the power to be restored to turn on the fan before going to bed*. *The fan has its limits*. *The second disadvantage is that exposure to ventilation causes health problems*, *including flu*, *lung and bronchial disorders*. *So*, *it is not always recommended to sleep with a fan in your room*. *Even if you are not directly exposed to ventilation*, *it is not recommended to use a fan*.*”*

Some community members also expressed dissatisfaction with the cost, inconvenience and health hazards associated with insecticide sprays and repellent coils, often referred to specifically and generically by the common trade names *Placatox*^1^ or *Baygon*^2^, and also with natural products used for similar purposes:

Quote 2 (Assessment round 1, FGD, Male): *“As for Placatox*^*1*^, *its effectiveness is very short term*. *It soon turns to ashes after being lit*. *In addition*, *you have to stay away from it because of its unbreathable smell*. *People with asthma cannot breathe its unpleasant smell*. *I used natural fumigations to chase away mosquitoes*: *the burning of orange peels and palma-christi seeds*. *The doors of the house are closed during and after the fumigation*. *If the doors are open*, *the latter will have no repellent effect*, *hence its limitations*. *The duration of the effectiveness of the fumigation in question is very short*, *unlike that of the emanator*.*”*

Quote 3 (Assessment round 3, FGD, Female): *"Before the project arrived in my neighbourhood*, *I used Placatox*^*1*^. *They affected the quality of my life by preventing me from breathing*. *They were causing me health problems*.*”*

Quote 4 (Assessment round 3, FGD, Male): *"Children and adults cannot breathe the smell of Placatox*^*1*^. *It is harmful to my health*. *I used Placatox*^*1*^ *to chase away mosquitoes even though I couldn’t stand its smell*. *It hurts babies more*. *… I had no choice*, *since there were too many mosquitoes*.*"*

According to one participant, who also features in quotes 1 and 2, mosquitoes had developed noticeable resistance to these existing repellent emanator products:

Quote 5 (Assessment round 1, FGD, Male): *“Baygon*^*4*^ *was created to kill mosquitoes*. *But in recent years my observation is that Baygon*^*4*^ *and other chemicals have become ineffective*. *Today’s mosquitoes are highly resistant to it*. *Baygon*^*4*^ *disturbs people who are more sensitive to it than mosquitoes*. *These have adapted to the products over the years*.*”*

### Protective efficacy of transfluthrin emanators against mosquitoes

**Fig 3:**
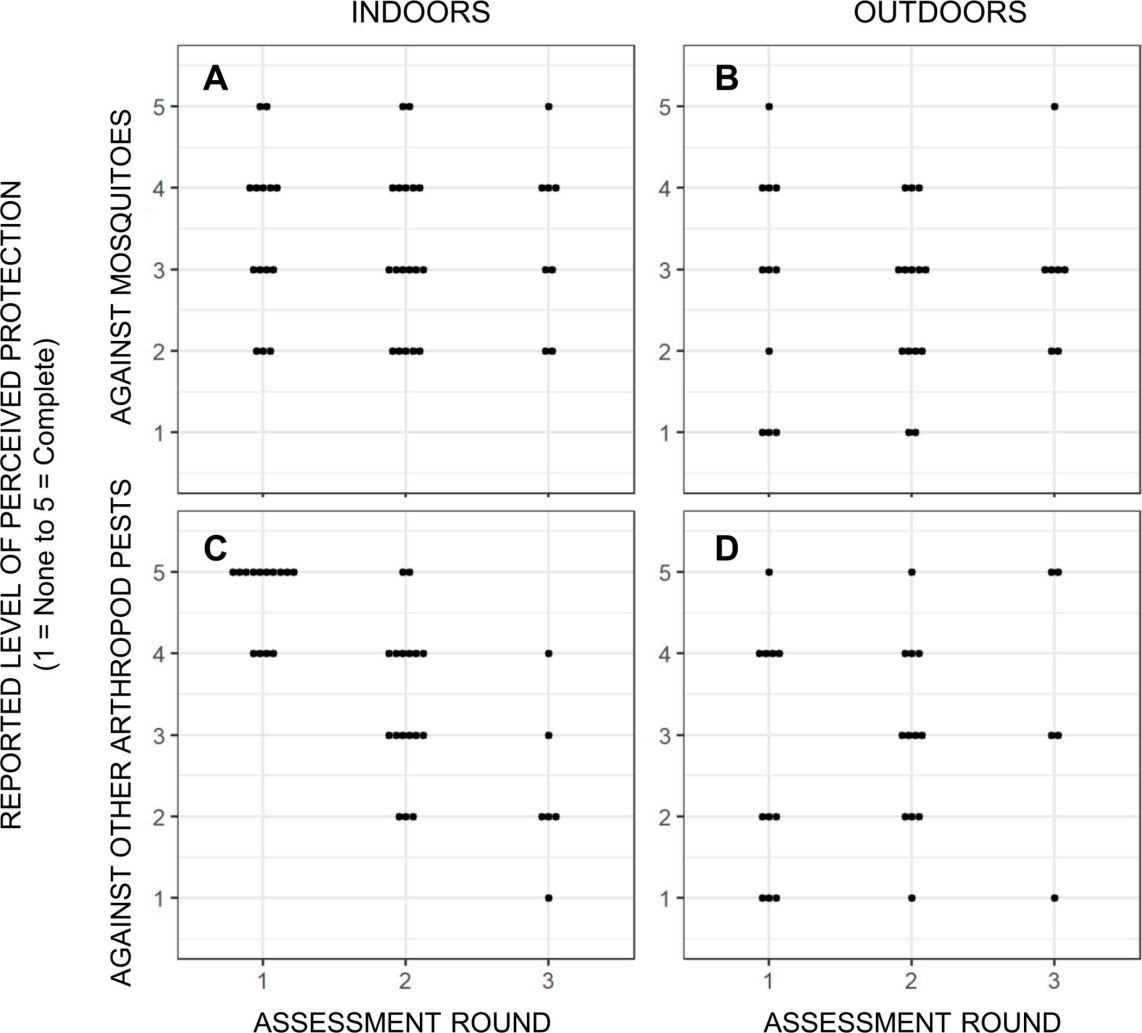
Reported perceptions of satisfaction with transfluthrin emanators by participating households provided with them for routine use indoors (**A** and **C**) and outdoors (**B** and **D**), as expressed in terms of protection against mosquitoes (**A** and **B**) and other arthropod pests (**C** and **D**) on a scale of 1 (None) to five (Complete) and recorded in a semi-quantitative questionnaire.

In contrast, transfluthrin emanators were viewed more positively by any participants, some of whom observed that they appeared to be more effective indoors, where the active ingredient seemed to dissipate far more slowly than outdoors. In the questionnaire surveys carried out during regular household visits, participants generally expressed moderate satisfaction with the emanators as a means of protection against mosquitoes and other insect pests outdoors (Fig 3). Indoors, however, greater protection against mosquitoes was perceived, ranging from moderate to high. While the perceived effectiveness against mosquitoes was generally satisfactory both indoors and outdoors, with little variation across evaluation rounds (Fig 3A and 3B, respectively), it was less robust outdoors (P = 0.049).

According to several participants, the emanators protected their family members against mosquito bites, yielding tangible improvements in their everyday lives:

Quote 6 (Assessment round 1, FGD, Male): *“As long as you have an emanator next to you*, *you can play with your children*, *manage your activities*, *cook and use a laptop computer without running any risk of being bitten by mosquitoes*.*”*

Quote 7 (Assessment round 1, IDI, Male): *“Currently*, *there are fewer mosquitoes*. *Thanks to the emanators*, *we can sleep more peacefully*. *… The presence of the emanators has improved the quality of our lives*.*”*

Quote 8 (Assessment round 3, FGD, Female): *“Thanks to the emanator I use*, *the quality of my life improves*. *… Once placed next to me*, *it repels mosquitoes while producing no odour*.*”*

Quote 9 (Assessment round 3, IDI, Female): *"The emanator protects me and my baby from mosquitoes*. *It’s protecting my baby*. *That’s the benefit I get from it*. *I no longer see mosquitoes in the house*.*"*

Some participants mention satisfactory efficacy when used in outdoor setting. Examples of diverse use practices outdoors are illustrated in Fig 5:

Quote 10 (Assessment round 3, FGD, Male): *“I have a niece who usually goes to study on the roof of the house*. *She puts an emanator next to her to repel mosquitoes*. *She’s allergic to mosquito bites*. *A friend and I are used to playing chess in my garden*. *We place one emanator on our left and another on our right*.*"*

Quote 11 (Assessment round 1, FGD, Female): “*When I am outside the house*, *I use an emanator*. *It protects me from mosquitoes*. *They’re not coming near me*. *Before*, *when I used to put an emanator in the yard of the house*, *no mosquitoes dared come near me”*.

According to some, the efficacy of the emanator depended on the distance at which it is located. For example:

Quote 12 (Assessment round 3, FGD): *"The closer it is to yourself*, *the more effective it is*. *For example*, *when you wash the dishes*, *you put it next to you*. *When you cook*, *you do the same thing*. *When we sleep*, *it is placed next to our beds*. *This is the best way to use the emanator*. *The closer it is to you*, *the more effective it is*.*"*

**Fig 4:**
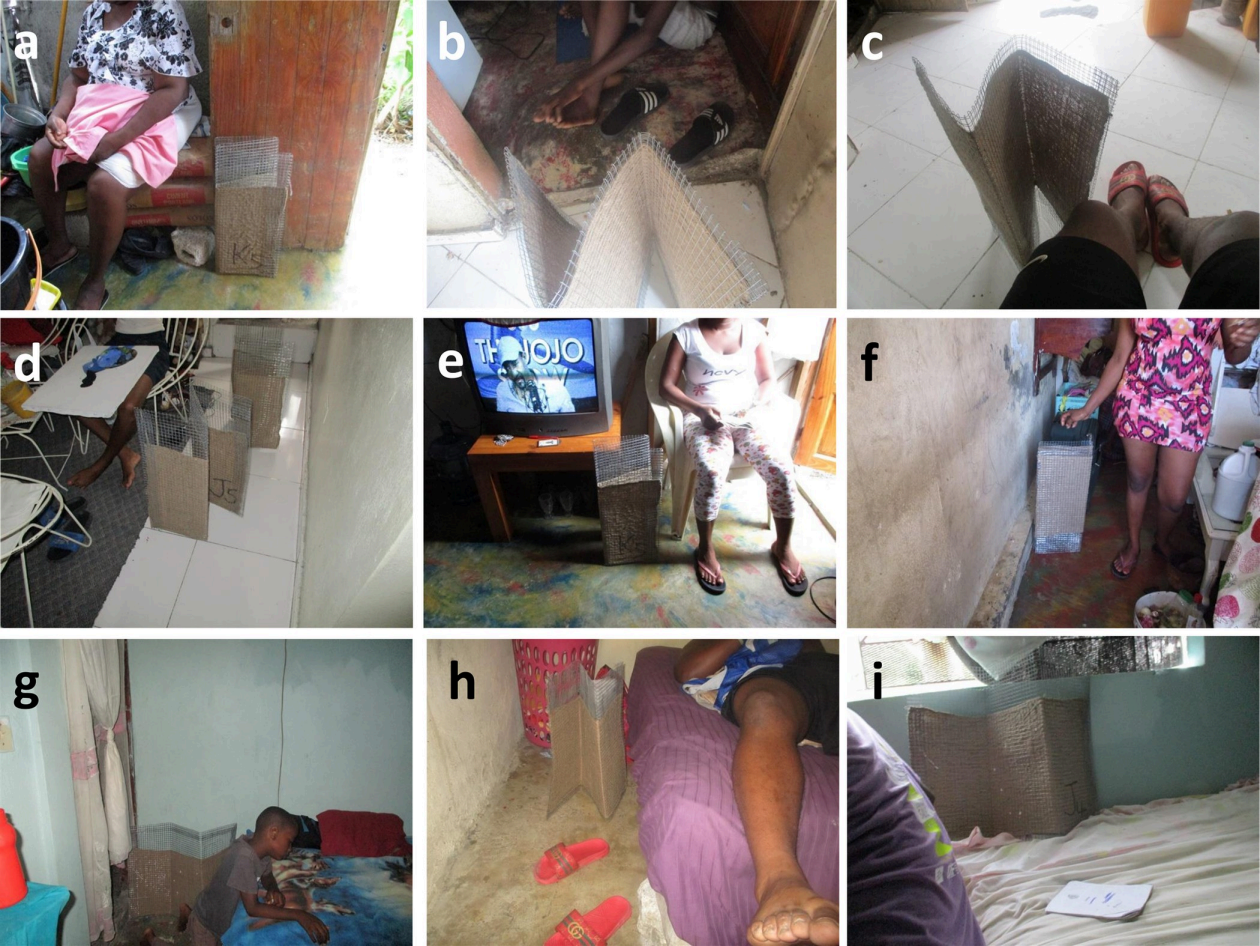
Photographs taken by household participants from the third assessment round documenting various situations of indoor emanator use. Panels **a** to **e** depict typical use practices while sitting down or resting in the living room, panel **f** while cooking in the kitchen, panels **g** to **i** while resting, playing or sleeping in a bedroom.

**Fig 5:**
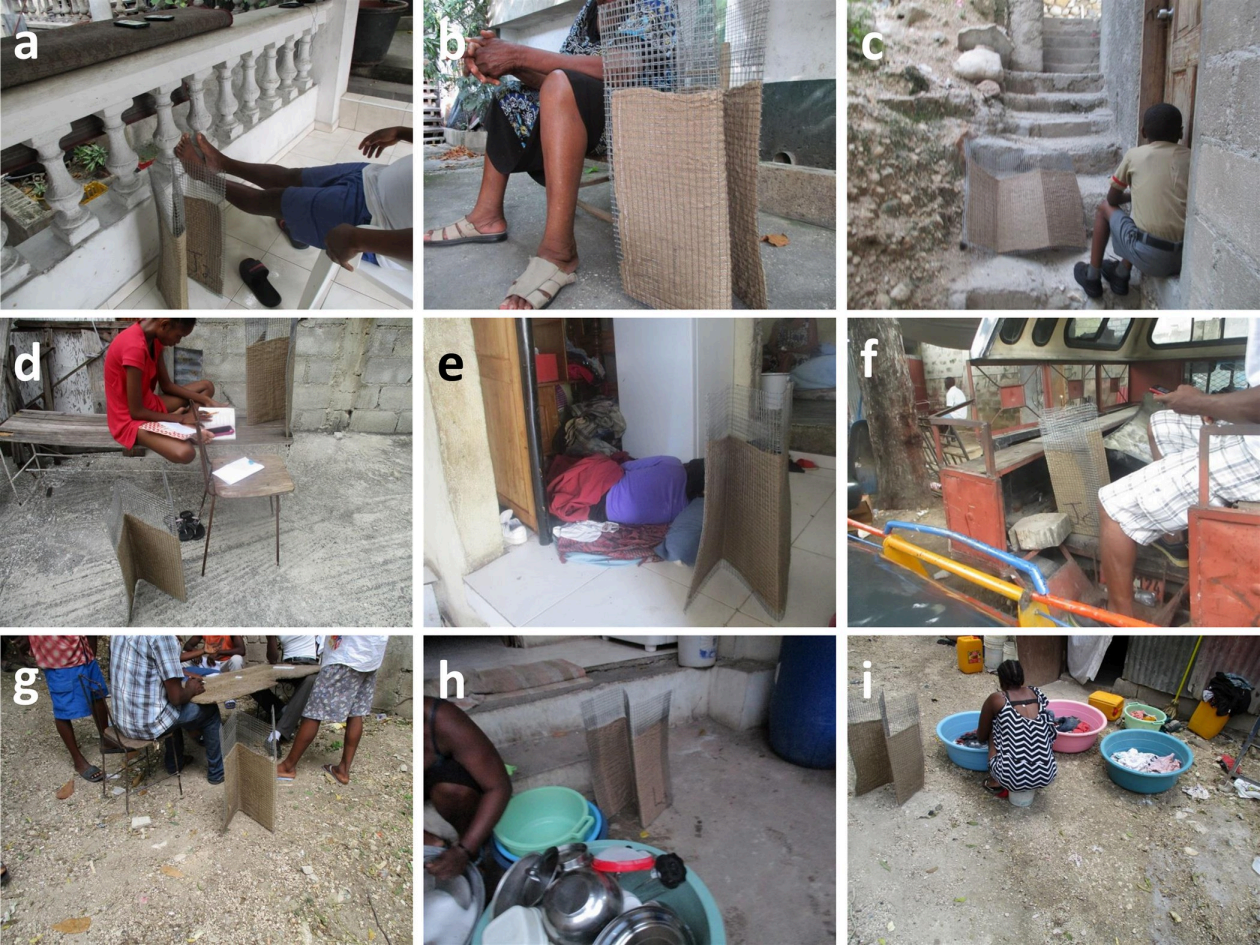
Photographs taken by household participants from the third round of assessments documenting various examples of outdoor emanator use practices by end users. Panels **a** to **c** depict use practices while resting on a porch or veranda, panel **d** while studying, panel **e** while sleeping in the daytime, panels **f** and **g** while playing or socializing, and panels **h** and **I** while washing dishes or clothes.

The photographs taken and selected by the participants in the PV component show the diversity of the emanator use practices inside and outside the houses, encompassing all the most common activities of households: laundry, cooking, ironing, cleaning, studying, recreational activities, etc. (Figs 4 and 5).

### Protective efficacy of transfluthrin emanators against other insect pests

Perceived efficacy against other arthropod pests, such as cockroaches and flies, was remarkably high indoors in the first evaluation round but then declined over subsequent assessment rounds (Fig 3C). As for mosquitoes, perceived effectiveness against other arthropod pests was somewhat lower (P = 0.022) and more variable outdoors (Fig 3D) than indoors (Fig 3C). For reasons that are not obvious, reported perceived efficacy against arthropod pests other than mosquitoes appeared to decline from assessment round 1 to round 2 and then round 3 (Fig 3C). Although one participant mentioned centipedes and another mentioned ants, several described how the emanators not only protected them against mosquitoes but also against flies and cockroaches. For example:

Quote 13 (Assessment round 3, IDI, Female): *"The emanator effectively repels flies and mosquitoes*. *Besides*, *I don’t see any more cockroaches in the house*.*"*

### Durability of the efficacy of transfluthrin emanators

The durability of the efficacy of transfluthrin emanators was the subject of contrasting perceptions that varied between individuals and households. For some people, the time since receiving the emanators did not reduce their perceived effectiveness:

Quote 14 (Assessment round 3, FGD, Female): *"The emanator has always chased away mosquitoes*. *Its effectiveness has never diminished*.*"*

For many others, however, the effectiveness of repellent transfluthrin vapour decreased within months, weeks or even days. For example:

Quote 15 (Assessment round 1, IDI, Male): *“When the emanator has just been treated*, *it is able to perfectly chase away mosquitoes*. *… After two or three months*, *it still repels mosquitoes*, *but with less effectiveness*. *Initially*, *repellent vapour is powerful*. *Time does not* [fully] *erase its effectiveness*.*”*

Quote 16 (Assessment round 3, IDI, Male): *“During the first few days of use*, *the emanator was more-or-less effective*. *After one week*, *its effectiveness has decreased significantly*. *Despite its presence at home*, *the mosquitoes come back to bite us*.*”*

Quote 17 (Assessment round 3, FGD, Male): *“As far as I’m concerned*, *its effectiveness lasted two days*. *I wonder why its effectiveness lasts longer with other people*. *The day I used the emanator*, *it was 100% effective*. *On the second day*, *its effectiveness deteriorated slightly*. *Some mosquitoes have returned home*. *On the third day*, *its effectiveness is reduced to 60%*.*”*

Interestingly, the narratives of many participants suggested that they had very stringent expectations of what level of sustained protection was satisfactory, with even a single mosquito bite considered one too many:

Quote 18 (Assessment round 1, FGD, Female): *“The emanator contains something whose effectiveness is not unlimited*. *I must say*, *I see some mosquitoes again*. *It appears that the thing in the emanator is losing its effectiveness*. *Last week*, *I heard a mosquito hum while I was lying in my bed*. *I don’t know what role he plays in the government*^*3*^. *… If some mosquitoes come back*, *it is because the substance in the emanator has weakened*.*”*.

Some participants in IDIs and FGDs perceived that emanators lose their effectiveness due to weather conditions and/or environmental conditions inside their houses. For example, in relation to exposure to moisture:

Quote 19 (Assessment round 3, FGD, Male): *“I put the emanator away from the water*. *I don’t want the transfluthrin to get wet*. *I put it in a dry place*.*”*

Having said that, some participants shared more nuanced perspectives about the role of weather conditions and the domestic environments they lived in, overtly recognizing that these directly influence mosquito biting densities, regardless of emanator efficacy. Rather than associating them with emanator efficacy loss, they explained how these highly variable environmental factors should be considered when deploying of emanators:

Quote 20 (Assessment round 1, FGD, Male): *“The emanator must be used wisely*. *In our house*, *there are rooms located to the east*, *with large windows*, *which allows them to be well ventilated*. *You won’t easily find mosquitoes in my house*, *because I live in an area where the temperature is cool*. *The rooms are very sunny*. *At night*, *it is very cool*. *There are not many mosquitoes in cool areas*. *The rooms to the west are very humid*. *In damp and hot rooms*, *there are many mosquitoes*. *Emanators must be used wisely in hot areas to be effective*. *In cool*, *well-ventilated houses*, *there are no mosquitoes*. *They like warm temperatures*. *When it is hot*, *we put an emanator in the living room*. *In the evening*, *it is left in our room*. *Sometimes it is put between two rooms*. *These techniques of use give positive results*. *In addition*, *the emanator must be used according to the temperature variation*.*”*

Some users perceived that there could be a link between air flow and environmental factors that influence it (e.g. indoor vs outdoor, different house design) and impaired effectiveness of the transfluthrin emanator. We do not know how many participating households restrained themselves from using the product outside their homes to maintain its protective efficacy, but such practices were hinted at by several participants. The following quote suggests that prior experiences with other insecticide products available on the local market may have enabled them to perceive these phenomena through observation and develop these use practices based on their own intuitive reasoning before they were introduced to transfluthrin emanators through this study:

Quote 21 (Assessment round 1, FGD, Female): “*I think that exposure to the open air has an impact on the duration of its effectiveness*. *The more it is exposed to the open air*, *the less effective it is*. *I think that if it is used in a sealed space*, *the duration of its effectiveness will increase*. *If it is taken as a mischievous pleasure to expose it outside the house*, *the duration of the emanator’s effectiveness will decrease*. *When you use a Baygon*^*4*^ *inside your home*, *it lasts longer*, *it is more effective*. *If it is used outside the house*, *it will not last*. *If used indoors*, *the evaporation of chemicals will be slower*.*”*

**Fig 6:**
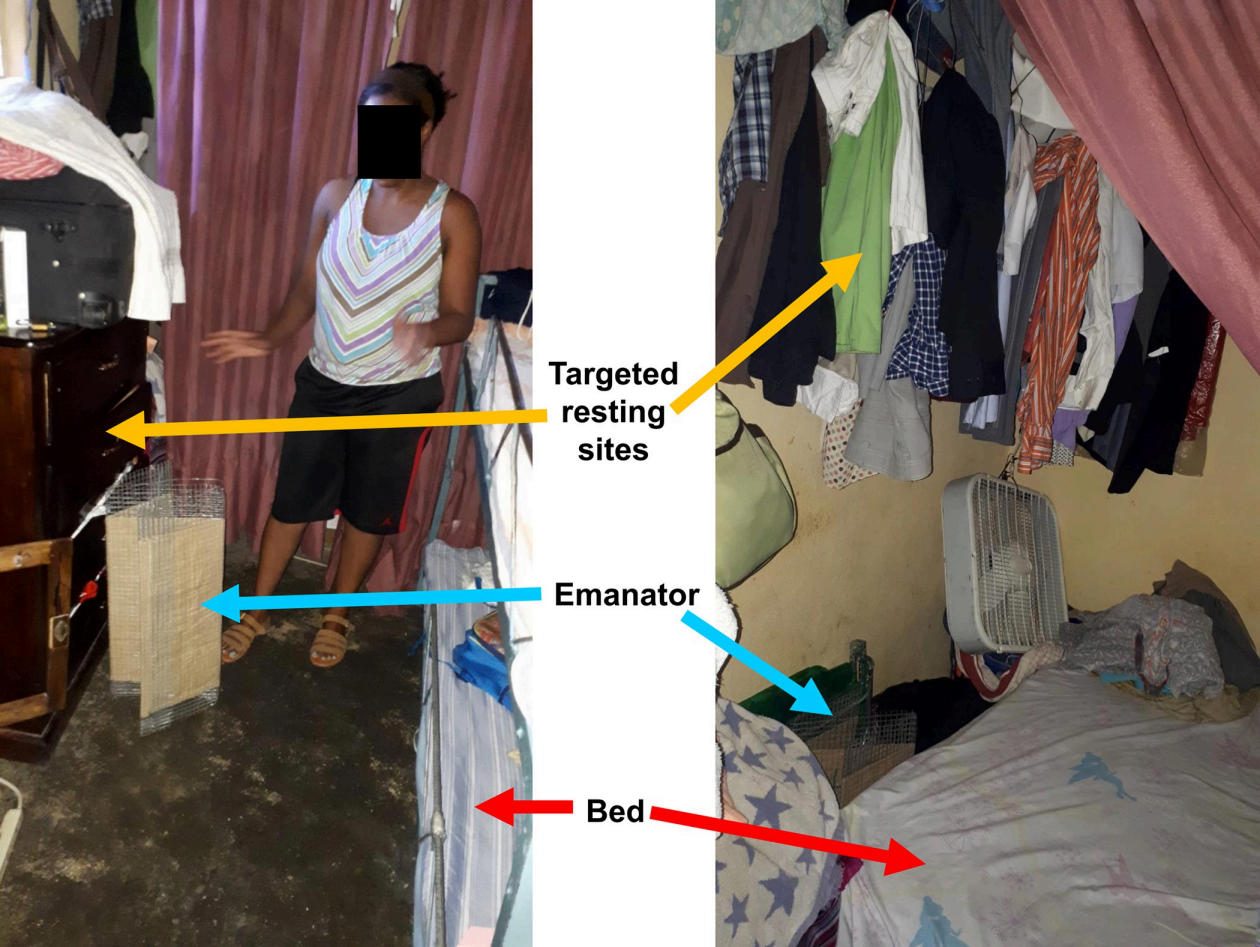
Photographs illustrating preferred use practices of one participant in the first set of sociological evaluations of perceived effectiveness, which seem specifically intended to target mosquitoes when they rest indoors rather than when they attack the occupants. Note, however, that these photos were taken during an informal field visit to follow up after the first set of social science assessments, rather than during those formal photovoice surveys.

Because several end-users perceived the emanators to be most effective indoors and at night, we hypothesized that they might be more effective against endophilic, nocturnal *Culex quinquefasciatus* than against the exophilic, diurnal or crepuscular *Aedes* species that were the primary target species of the study. Also, some informal discussions with participants while following up on the first of these formal sociological evaluations, and soon after the third round of entomological assessment [26], confirmed that view: the usage pattern illustrated in Fig 6 strongly suggested the participant in question was actually targeting mosquitoes while they rest indoors, rather than when they attempt to land and bite. Such perspectives noted during the first and second round of these social sciences investigations prompted us to change the design of the entomological assessment protocol when it was repeated for a third time, to focus human landing rate measurements on the 6-hour time window on either side of dusk (4pm to 10pm) and conduct these trapping activities both indoors and outdoors rather than indoors alone [26].

### Advantages of transfluthrin emanators relative to existing protection measures

Some participants indicated that occupants of low-income households do not use bed nets, mainly due to limited access and lack of affordability, and resorted instead to covering themselves with one or more sheets to protect themselves against mosquito bites. Also, because of overcrowding and tropical heat, they perceived the net as a cumbersome product that was difficult to accommodate in cramped living spaces and also prevented them from breathing well and sleeping at ease. One notable point of concern is that some participants reported sleeping without needing to use a bed net as a specific advantage of emanator use:

Quote 22 (Assessment round 1, FGD, Female): *“The net produces heat*. *One of the nice advantages of the emanator is that it allows us to sleep without covering ourselves*. *Although it is bulky and unsightly*, *it allows us to sleep well*. *We are not hot because of its presence*.*”*

Quote 23 (Assessment round 1, FGD, Female): *“I had a mosquito net*. *I couldn’t sleep without covering myself*. *Thanks to the use of emanators*, *I can sleep without covering myself*. *Mosquitoes no longer bother me inside and outside the house*. *… Thanks to the project*, *I no longer see mosquitoes*.*”*

Some households using transfluthrin emanators attributed preventive health benefits to doing so, because they were considered a safer, odourless (See *Smell of the emanators and its association with protective efficacy and/or health risks*) alternative to mosquito repellents, insecticide treated bed nets and other chemical products that were considered hazardous:

Quote 24 (Assessment round 3, FGD, Female): *"As far as I’m concerned*, *I have to tell the truth about the emanator*. *I really love it*. *After lighting Placatox*^*1*^ *coils*, *I couldn’t breathe in their smell*. *I had a feeling of suffocation*. *Sometimes I would use a mosquito net*, *but it caused itching and burning when my body touched it*. *It contained a substance*. *I have nothing to blame the emanator for*. *It’s perfect*.*"*

Quote 25 (Assessment round 3, FGD, Male): *"The advantage I get from the emanator is that it doesn’t emit any unpleasant odours*. *We can’t breathe the smell of Placatox*^*1*^ *and Baygon*^*2*^. *The emanator does not produce any odours*. *It does not affect health*. *It does not waste our health*. *It is not harmful*…*"*

Consistent with the testimonies described in previous sections, several users of the emanators described having stopped buying other protective products (Bed nets, mosquito coils, lotions, insecticide sprays, electric rackets etc.) and considered this to be an important economic benefit:

Quote 26 (Assessment round 3, IDI, Male): "*Another advantage is that I no longer buy Placatox*^*1*^. *For me*, *this product no longer exists*. *I bought Placatox*^*1*^ *every day*. *Ten gourds*. *Sometimes I would buy two mosquito repellent spirals*. *Now my money stays in my pocket*. *I buy other things with my money*. *I no longer buy Placatox*^*1*^.*"*

One exception was a participant in a male FGD who mentioned the time-limited nature of its free provision and the possibility that it might enter the market at a prohibitive price one day:

Quote 27 (Assessment round 1, FGD, Male): *“For the time being*, *we can say that*, *economically speaking*, *the emanator costs us nothing*, *since it has been given to us free of charge*. *We didn’t spend a cent to get it*. *If one day its price is set at twenty thousand US dollars*, *will we say that it still offers us an economic advantage*? *For my part*, *the answer is no*.*”*

### Disadvantages and risks of using transfluthrin emanators

Other than the limited durability of efficacy noted by some participants, several other disadvantages were narrated. While the majority of participants did not mention the inconvenience of the device’s bulk, some did raise this as a concern:

Quote 28 (Assessment round 1, FGD, Female): *“The great disadvantage of the emanator is that it is too large*. *If we reduce its size*, *it will allow us to move it more easily*.*”*

Another user, who lived in a small home where there was not enough room, specifically mentioned the possible inconvenience and physical hazard in households with active children living in similarly cramped conditions:

Quote 29 (Assessment round 1, FGD, Male): “*For my part*, *the emanator does not pose a problem in terms of inconvenience*, *because I do not have children in my home*. *…*. *I don’t have anyone who can run into the emanator*. *It can’t hurt us*. *I live with my mother*.*”9*

Indeed, the need to prevent children accessing or touching the emanator was described as a major disadvantage by a few users. While a few adults mentioned being harmed by the sharp ends of the wires of the mesh covering, children were a particular concern in relation to this minor physical hazard:

Quote 30 (Assessment round 1, FGD, Male): “*The other day*, *one of them got pricked by touching it*. *This is a disadvantage*. *The emanator’s protective support pricks the children*. *As an adult*, *I take precautions*. *But we can’t stop children from touching everything*.*”*

In addition to being too bulky and awkward (Quotes 28 and 29), the emanators devices were also described as unattractive by a few participants. All who expressed an opinion considered that the aesthetics of the device had been compromised in favour of utility:

Quote 31 (Assessment round 1, FGD, Male): *“…the emanator is sorely lacking in beauty*. *… we have to rethink the aesthetics of the product*.*”*

A few participants also expressed concerns that a potential toxicity hazard was associated with the transfluthrin active ingredient, through inhalation, dermal and oral exposure routes. For example, one parent who feared that transfluthrin vapour was harmful to the health of his children:

Quote 32 (Assessment round 1, IDI, Male): *I fear that the emanator will have a side effect on children*. *I have children*. *I have one who’s six years old*. *I don’t want him to touch the emanator or bring it to his mouth*. *I don’t know what will happen to him if he puts it in his mouth*.*”*

Indeed, health concerns regarding exposure to transfluthrin vapour were sometimes described as one of the reasons for not using it. However, perceptions of the health risks associated with transfluthrin varied between and within households. On the one hand, for example, one 27-year-old female student feared that transfluthrin vapour might be hazardous:

Quote 33 (Assessment round 1, IDI, Female): “*I was told that it is impregnated with a chemical product*, *which can make me sick*. *This product is not visible to the naked eye*. *It can impact my health*. *Perhaps the harmful effect of the chemical will manifest itself in the long term*. *At the moment*, *I don’t know*. *I’m just expressing my fear*.”

On the other hand, she lived under the same roof as her brother (23 years old) whose perspective differed somewhat:

Quote 34 (Assessment round 1, IDI, Male): “*I respect the precautions when using the emanator*. *I know how to touch it*. *I’m not touching the burlap* (hessian). *In my opinion*, *transfluthrin vapour has no impact on our health*.”

### Smell of the emanators and its association with protective efficacy and/or health risks

As indicated above in quotes 8, 24, 25 and 35, a few participants expressed the view that the emanators had little or no smell and considered this an advantage over alternative mosquito control products, especially in relation to the health hazards they specifically associated with odorous insecticides and repellents:

Quote 35 (Assessment round 1, FGD, Male): *“I am allergic to insecticide products available on the market*. *I also have to be careful about the type of perfume I use*. *When I was told about the arrival of the project in my block*, *a repellent transfluthrin emanator project*, *I was perplexed*, *if not hostile*. *I thought it would have serious consequences on my health*. *I have asthma*. *… The smell of the product does not make me suffocate or disturb me*. *Other mosquito repellents triggered my allergy*, *gave a burning sensation in my nose*, *made me scratch my throat*, *prevented me from breathing well*. *I had a feeling of suffocation*. *Although I use the emanator all day long in my immediate environment*, *it has no impact on my health unlike other chemicals used to control mosquitoes*.*”*

There were also, however, some very different views expressed. For example, although all the other women who participated in the first female focus group testified that the emanator did not emit any odour, one who described herself as asthmatic and unusually sensitive to all smells disagreed:

Quote 36 (Assessment round 1, FGD, Female): “*Yes*, *the emanator gives off an odour*. *When it’s too close to me*, *I can smell its strong odour*. *The latter decreases and disappears with time*. *Its smell is not very pleasant*. *I am an allergic person*. *Anything can make me sneeze*, *like for example*, *the smell of a perfume*.*”*

**Fig 7:**
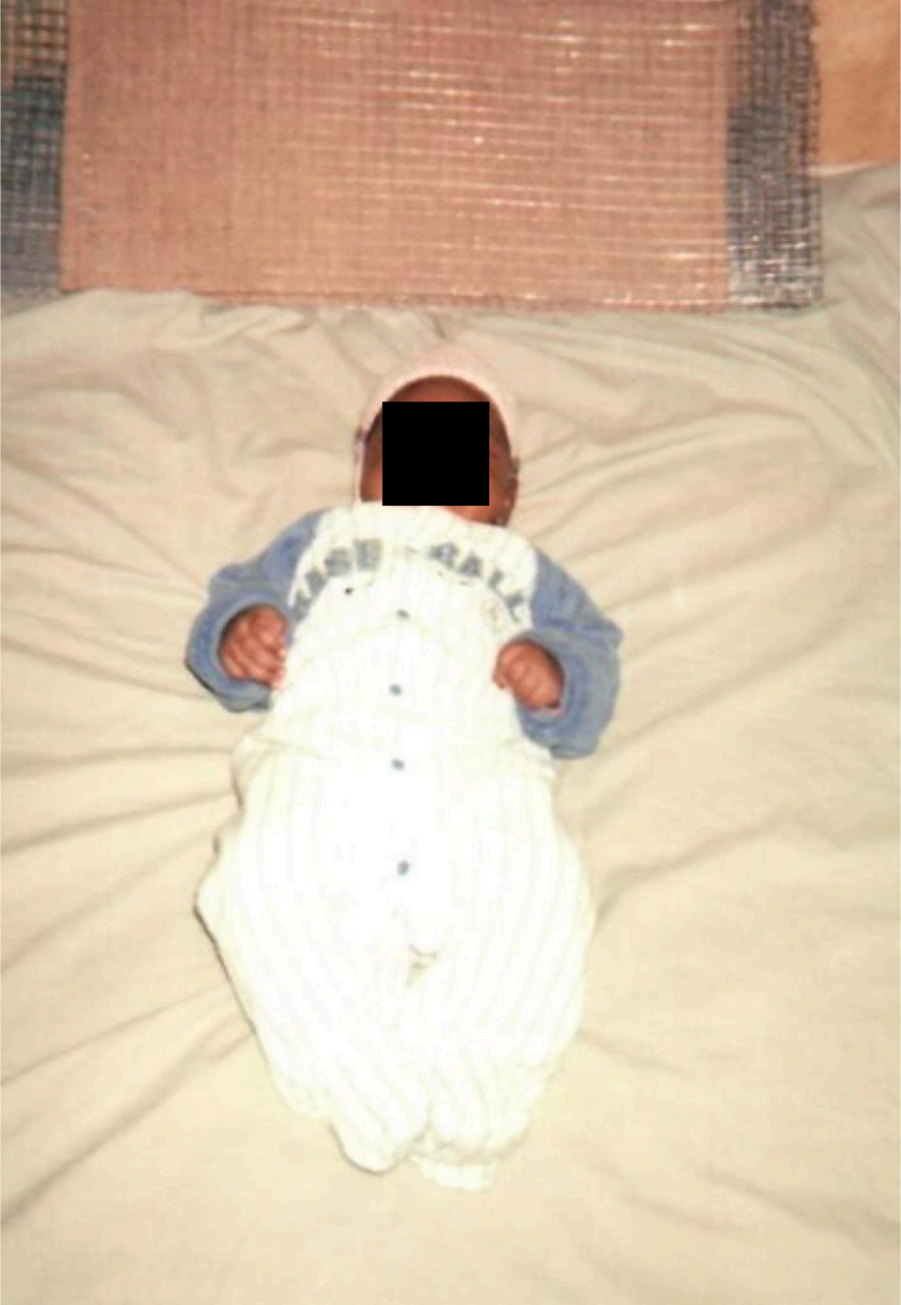
An unforeseen use practice recorded during the first round of *Photovoice* surveys conducted alongside the first round of entomological evaluations in mid-2018, in which an infant was placed on the ground right beside emanator, with its nose and mouth closest to the emanator where transfluthrin vapour concentration is expected to be greatest.

Furthermore, the apparent lack of perceived odour or inhalation exposure hazard among most participants, combined with the common perception that proximity to the emanator device maximizes protection Quote 12), may also have underpinned one quite worrying use practice identified through the first round of *Photovoice* surveys. Fig 7 illustrates how one end user placed an infant lying on a bed right beside an emanator. This use practice, which places the nose and mouth of an infant close to the emanator where transfluthrin vapour concentration is expected to be greatest, was not foreseen at the outset of the study but was then immediately acted upon to advise participants against this use practice, and then to inform design of the experiments to measure vapour inhalation exposure measurements in Tanzania [33].

Households were accustomed to using certain mosquito repellents that emit a readily perceptible odour, and a few perceived a smell from the transfluthrin emanators that they similarly associated with their protective efficacy:

Quote 37 (Assessment round 1, IDI, Male): *“…it was more effective during the first few months of use because it smelled very strong*. *The smell has decreased*. *When it has just been treated*, *it is more effective*, *so we hardly saw any mosquitoes*.*”*

For one male participant, the efficacy of the emanators was associated with its strong smell, which he also considered could potentially make his children sick:

Quote 38 (Assessment round 1, IDI, Male): “*The emanator was more effective during the first months of use*, *because its smell was very strong*.… *It gives off a smell that can harm the health of my children*.”

Having said all that, it is unclear if some participants perceived the smell of the hessian, the scented liquid detergent used to mix the transfluthrin, the transfluthrin itself, or merely the placebo effect of a device they expect to have an odour. Also, it seems that some participants might have indirectly used the word “smell” to describe the mosquito repellent activity of the transfluthrin vapour, suggesting that perhaps they may not have actually noticed any particular odour *per se*:

Quote 39 (Assessment round 1, FGD, Male): *"Outside the house*, *it repels mosquitoes*. *Let’s say 5 people sit outside*. *The smell of the emanator may not reach the fifth person*. *It depends on how we sit*. *The emanator is more effective in a limited space than in a large space*.*"*

### Potential role for transfluthrin emanators in strengthening links within the community

An unforeseen advantage of the emanators was that they were viewed by many participants as devices that could enhance community solidarity, through sharing of devices within and between households: This point is illustrated by the following detailed narrative explaining various sharing practices within one extended family, and then by two briefer accounts of sharing more broadly with neighbours:

Quote 40 (Assessment round 3, PV-FGD, Male): *"This picture shows my nephew drawing a fish*. *He was assisted by his aunt*. *The emanator was placed next to them*. *I took a picture of them*. *While he was sleeping*, *I put an emanator near his bed to protect him from mosquitoes*. *I took a picture of him*. *There was an emanator in the room*, *another one in the gallery*. *The third picture is of my mother*. *Every afternoon*, *my wife and I go up to the roof of the house to play games with pawns*. *My mother told me ‘Every afternoon I see you on the roof of the house*. *Today*, *I want to enjoy your company*. *Today*, *I want to enjoy your little* [sun]*bed*.*’ She then says*, *‘I see you have brought your emanator’*. *I replied*: *‘Mosquitoes disturb us*, *harm our health*. *We protect ourselves against mosquitoes that come from the nearby ravine’*. *The fourth picture is of my niece* [Fig 5D]. *She said to me*, *"Uncle*, *lend me an emanator*. *I told him*: *‘The emanators are here*, *why do you want to borrow them*?*’*. *She replied*, *‘I’m going to study’*, *so I allowed her to take it*…*"*.

Quote 41 (Assessment round 1, FGD, Male): *“We gave an emanator to a household that didn’t have one*. *… Three people can use an emanator while watching television*.*”*

Quote 42 (Assessment round 3, FGD, Male): *"Young men in my neighbourhood use my emanators when they have fun*, *when they drink beer and when they play dominoes*. *I lend them to them*. *I put them next to them*. *Mosquitoes don’t come to bite them*.*"*

Emphasizing the importance of sharing among neighbours, one participant noted the potential for incomplete community coverage to exacerbate existing inequities of exposure to mosquitoes and cause friction between neighbours by diverting mosquitoes from protected emanators users to nearby non-users:

Quote 43 (Assessment round 1, FGD, Female): *“She said there were no more mosquitoes in her house*. *This means that the repelled mosquitoes reinforced those that were in his neighbours’ homes*. *If she wants everyone to use an emanator*, *it is because she likes to share*. *I agree with her*.*”*

### Ideas for improving and promoting the use of transfluthrin emanators

A few participants indicated that the aesthetic appearance and ergonomic practicality of this emanator prototype needed to be improved upon:

Quote 44 (Assessment round 1, FGD, Male): “*I thought it would be more attractive if it was aesthetic*. *If it was beautiful*, *we would explain its benefits to the guests*. *If it was beautiful*, *we would put it anywhere*, *we wouldn’t have to hide it behind a chair*, *or in a corner*. *If it had a decorative aspect*, *it would give us more results*. *I think we need to improve its material presentation*.*”*

Also, several made recommendations for improving their efficacy in various ways. Based on their lived experiences as end-users, some participants indicating that increasing the number of emanators would increase their protective effects:

Quote 45 (Assessment round 1, FGD, Male): “*I had given an emanator to* [name]. *I still have one left*. *When I had two*, *it was more efficient*. *Now I only have one*. *It lacks efficiency*.”

As an alternative, some end users suggested increasing the dose of active substance in the emanator:

Quote 46 (Assessment round 1, FGD, Male): *“We must increase the quantity of substance in the emanator*. *The more the emanator is used*, *the more the substance it contains decreases*. *If these substances are not increased*, *the expected efficiency of the emanator will not be achieved*. *If we do*, *we will allow it to repel more mosquitoes*.*”*

However, another participant expressed concern that it might be dangerous to increase the dosage of transfluthrin:

Quote 47 (Assessment round 1, FGD, Male): “*Many people want to increase the dosage of the products used to treat the emanator to extend its durability*. *My question is this*: *If we do*, *won’t we have health problems*? *The dosage of any chemical must be respected*. *Overdosing of a product can have lethal effects*.*”*

One participant called for the regular treatment of hessian cloth strips with transfluthrin in order to mitigate against the perceived decline in their efficacy over time:

Quote 48 (Assessment round 1, FGD, Male): “*The efficiency of the emanators decreases gradually*. *I don’t know if it is possible to increase the effectiveness of the insecticide every month*.”

For some participants, the perception that the emanator does not kill mosquitoes was considered a disappointment:

Quote 49 (Assessment round 1, FGD, Female): *“It chases mosquitoes*, *it doesn’t kill them*. *I wish it would kill these pests*.*”*.

Indeed, some echoed the above theme that the transfluthrin dose should be increased specifically to kill rather than repel mosquitoes:

Quote 50 (Assessment round 3, IDI, Female): "*The emanator’s dose of transfluthrin must be increased to allow it to kill mosquitoes*".

Furthermore, the verbs "kill" and "destroy" occurred frequently in relation to mosquitoes throughout all IDIs, FGDs and PV-FGDs, reflecting the frustrations of emanator users who expected such definite impacts from the device.

Most transfluthrin emanator users expressed a desire to see it become a mosquito repellent product that is accessible and affordable for local communities in Haiti. One participant emphasized the need to commercialize the emanator product and make it available for sale at an affordable cost:

Quote 51 (Assessment round 3, FGD, Female): *“I would like to share the idea that the emanator should become a product available in all households*. *I know that it will not be possible to distribute it free of charge*. *It should be sold at an affordable price*. *It should be sold at a modest price*. *If the price is not too high*, *we can offer it to people*. *People who have already experienced it will be able to offer it to homes*. *They will convince them to buy it*. *The emanator is very useful*. *I received it for free*, *but I think it could be commercialized*. *People who have already experienced it will convince households of the benefits of its use*, *so that each household buys one or more emanators*.”

Some emphasized the need for full community-wide coverage with these devices, as well as more devices per household, to maximize equity and impact. Many participants considered the emanators to be suitable for community-based promotion and some expressed an altruistic desire to distribute emanators across their respective neighbourhoods:

Quote 52 (Assessment round 1, FGD, Female): “*If you give me more emanators*, *I will share more with my neighbours*. *I cannot benefit from something that my neighbours also need*.”

Quote 53 (Assessment round 3, FGD, Male): “*I sincerely hope that more emanators will be brought to me so that I can distribute them in the neighbourhoods*. *People stop me from using mine*. *They’re borrowing it*. *Today*, *a person uses it*. *Tomorrow*, *another person will use it*. *It is better to lend it to people who have never tried it before than to keep it for yourself*. *For this reason*, *I would like to have more so that I can give it to people*, *which will allow me to use mine*.”

Indeed, one participant spontaneously compiled a waiting list of interested households in his neighbourhood, while another went so far as to mobilize community demand through a neighbourhood-level petition. Interestingly, one PV participant expressed her wish to use photos of the emanators for promotional and marketing purposes, in the event of emanator products being offered for sale on the market:

Quote 54 (Assessment round 3, PV-FGD, Female): “*These pictures mean a lot to me*. *They are the result of my imagination*. *They represent realities*. *They reflect the way we used the emanator*. *… These photos show that the emanator has been used in various ways*. *… I took some souvenir pictures of it*. *When the emanator is for sale*, *I will use its photos to convince people to buy it*. *I will explain to them how I use it*.”

### Complement emanators with community-based environmental management approaches

Some participants shared their view that neither these transfluthrin emanators nor any other protection measure is perfect and therefore emphasized the importance of community-based environmental management as a complementary intervention. For example:

Quote 55 (Assessment round 1, FGD, Male): “[The emanator] *will not be effective if it is not accompanied by preventive behaviours in the environment*, *which must be well maintained*. *I told you I have a water basin in my kitchen that produces mosquitoes*. *As soon as I started using the emanator*, *I destroyed the water basin*. *The mosquito breeding ground no longer exists*. *You need to clean the gutters and collect the mess from your neighbourhood if you want to better evaluate the effectiveness of the emanator*. *If the environment is not clean*, *it will be more difficult to assess the effectiveness of the emanator*. *The environment must be prepared before using its emanator*.*”*

## Discussion

Consistent with previous reports, participants in this study expressed hostile attitudes towards mosquitoes [13–25], as well as considerable dissatisfaction with existing options for protecting themselves against them [13, 14, 16, 17, 19, 22, 24, 25]. Similar to previous studies, some of which reported similar perspectives on insecticide-treated clothing [19, 22], physical protection measures like bed nets, bed sheets and fans were considered to have several limitations in terms of protection level, practicality and comfort [13, 14, 16, 18, 20],. Also, similarly to previous studies, the cost, inconvenience and hazards of insecticide sprays and coils, which have to be repeatedly reapplied, were considered major drawbacks [13, 14, 16, 17, 19, 22, 24, 25].

In contrast, transfluthrin emanators were viewed more positively by many participants and were considered by some to address most of the issues associated with current options (Table 2 and references [13, 14, 16, 17, 19, 22, 24, 25]). Having said that, some participants expressed concerns, similar to those reported for other repellents [13, 14, 16, 17, 19, 22, 24, 25], about their limited durability of efficacy, smell, health hazards, bulkiness, aesthetic unattractiveness and potentially high cost once priced for sale on the market (Table 2).

Consistent with another assessment of similar devices for passive emanation of transfluthrin in rural Cambodia [16], most participants in this Haitian study indicated that these emanator prototypes provided useful protection against mosquitoes. Similarly to a previously assessed repellent cream product [14], several also noted effects upon other arthropod pests, specifically mentioning flies, cockroaches, ants and millipedes. A wide variety of indoor (Fig 4) and outdoor (Fig 5) use patterns are reported herein, including some that were unforeseen (Fig 6) and even undesirable (Fig 7), but some participants noted that they appeared to be far more effective indoors, where they perceived that the active ingredient dissipated far more slowly than outdoors. Interestingly, some use patterns were documented that seemed to target indoor-feeding, night biting mosquitoes like *Cx*. *quinquefasciatus* in their resting places within houses (Fig 6), rather than protect householders outdoors against day-biting *Ae*. *aegypti* or *Ae*. *albopictus*, as originally intended by the investigators. Interestingly, this unanticipated use practice was consistent with the reported impact of another transfluthrin emanator device [5, 18, 37] upon densities of *Ae*. *aegypti* mosquitoes inside houses in Iquitos, Peru [5].

Despite the apparent loss of efficacy within weeks or days that was noted by many participants, the perceived durability of protection provided by these prototype devices nevertheless compared well with most other repellent products currently available, the most promising of which is also a passive transfluthrin emanator device [38]. Consistent with the impressions of investigators during previous assessments of earlier prototypes of the devices assessed herein [11], the narratives of many participants suggested that they had very demanding expectations of these devices, with any mosquito bites whatsoever considered a notable shortcoming. It is therefore possible that community user perceptions of efficacy loss over time might only be associated with modest increases in actual biting exposure, although that could not be confirmed because parallel entomological assessments yielded no evidence of any meaningful reductions in mosquito landing rates [26].

User perceptions that loss of efficacy was associated with outdoor use, specifically exposure to wind, sunlight and moisture, may be readily rationalized in well-established physical chemistry terms. Regarding the latter factor, it is notable that these perspectives compare well with those of Cambodian forest communities provided with a similar transfluthrin emanator device, who expressed concerns about it “getting wet from the rain” and its “ability to withstand rain” [16].

It is encouraging that transfluthrin emanators were sometimes perceived as a superior alternative to the use of fans and bedsheets, which are commonly used as a rather unsatisfactory means of physical protection against mosquitoes in Port-au-Prince and elsewhere across the tropics [13, 14, 16, 18, 20], and also to existing chemical options like insecticidal sprays and coils that were often perceived to be more inconvenient, expensive and hazardous by this community and several others [13, 14, 16, 17, 19, 22, 24, 25]. Nevertheless, it is of notable concern that some participants viewed these emanators as a preferred alternative to bed nets, rather than a supplementary measure. Indeed, similarly to users of a topical repellent cream product [14] and another transfluthrin emanator prototype [16], some participants mentioned that they stopped using bed nets once they had received emanators. This potential risk of undermining coverage with existing protective measures would need to be mitigated in any future scale up of transfluthrin emanators, especially in the many settings across the tropics where significant malaria transmission is mediated by predominantly nocturnal malaria vectors that usually attack people while they are asleep [39].

Like some users of a similar transfluthrin emanator prototype in Cambodia [16], many of the participants in this Haitian study considered these devices, which some of them described as odourless, to be safer than existing alternatives like repellent creams and combustible coils. However, while several participants recommended increasing the transfluthrin treatment dosage to maximize protection and ideally kill mosquitoes, a few others expressed reservations about its safety. Furthermore, observations of potentially hazardous unforeseen use practices (Fig 7) support the view that caution should be emphasized going forward. Indeed, preliminary studies in Tanzania that were informed by this observation (Fig 7) confirmed that transfluthrin vapour concentrations so close to an emanator treated with a higher dose than used here (15g active ingredient versus 3g) may be close enough to the regulatory limit of acceptable human inhalation exposure to cause concern [33].

It is encouraging that the emanators were commonly considered a technology that brought households and neighbours together through shared use, and that some participants emphasized the need for equitable access to adequate numbers of these devices across the whole community as a collective good. Similarly to one user of another transfluthrin emanator prototype in rural Cambodia [16], some Haitian participants expressed their willingness to help promote the devices if they became available after the study, and it is encouraging that two even made preliminary preparations for doing so on their own initiative. Regarding potential for commercial production, distribution and sale, as recommended by one Haitian end user, it is regrettable that neither monthly expenditure on pre-existing protection measures nor willingness to pay for this new option were specifically assessed here. Having said that, estimates of US$0.19 to US$3.40 for the former from Sri Lanka and US$0.49 to US$4.92 [23] for the latter in Cambodia [16] may give some idea of what might represent an affordable price in such low-income tropical settings.

These generally encouraging perspectives shared by end user households contrast strikingly with the consistently disappointing results of parallel experimental entomological evaluations of the exact same emanator devices in the same setting in Port-au-Prince Haiti [26] and in Dar es Salaam, Tanzania [33], as well as a sandal format prototype in a large cage semi-field system in Tanzania [40] and in the field in urban Brazil (Alvaro Eiras, Personal communication). Furthermore, while a recent large-scale assessment of a different transfluthrin emanator device that successfully demonstrated protection against *Aedes*-borne arboviruses in Peru also confirmed significant reductions of indoor-resting *Ae*. *aegypti* densities [5], those observed reductions were also remarkably modest. Indeed, focusing upon the blood-fed mosquitoes that represent the most direct indicator of human exposure, their results were essentially identical to ours (12% versus 13% reductions in Morrison *et al*. [5] and Supreme *et al*. [26], respectively). While the results of these complementary entomological studies would otherwise lead us to conclude that current emanator prototypes and transfluthrin formulations appear to provide little if any protection against *Aedes* in these urban, coastal tropical contexts, the perspectives shared herein by end-users in Port-au-Prince seem consistent with evidence of substantial epidemiological benefit in urban Peru [5].

Researcher biases towards their own preferred findings represent a pervasive problem across all fields of science [41, 42] and qualitative social science investigations are particularly prone to such systematic errors [43, 44] because “the researcher is the instrument in semi-structured or unstructured qualitative interviews,” [45]. Indeed, such qualitative sociological assessments of repellent products specifically have proven vulnerable to quite severe subjective biases [14] and particular care is merited in cases like this, where the scientific profile and livelihoods of the investigators and their research teams stand to benefit from evidence of a successful intervention that can leverage further funding [41–45]. Given that all the IDIs, FGDs and PV-FGDs were facilitated by first-named author of this article (OD), who was remunerated as a consultant for this specific work, it is certainly possible that subjective investigator bias was communicated to the participants. Also, the fact that he held a doctorate and an affiliation with a respected local university could well have exacerbated any such confirmation biases that arose from the natural preferences of scientific investigators to report results that are encouraging and/or aligned with their own *a priori* views, not to mention their own competing academic and financial interests [41–43]. Also, in the first two rounds of assessment, such potential biases originating from the investigators seem likely to have been matched by competing interests among participants because all the various questionnaires, interviews and discussions involved the same households that benefited substantively in financial terms from participation in the parallel entomological assessments of protective efficacy [26] (See *Ethical considerations*).

Nevertheless, these triangulated social science studies with several complementary survey methods yielded generally similar results in the third round of assessment, during which the occasional entomological assessments [26] were carried out in different households to those provided with the emanators for daily routine use, specifically to eliminate such competing interests among the latter (See *Ethical considerations*). Furthermore, several of the perspectives, ideas and use practices shared by these community end users were not anticipated by the investigators (Fig 6, for example), and one unforeseen was use practice was considered alarming enough in terms of participant safety (Fig 7) to merit corrective action by the investigators, so this is an encouraging sign that many of the views they shared were independently conceived and, therefore, presumably authentic. Indeed, informal follow-up discussions with end users, conducted only after the formal studies were completed to avoid influencing the views of the participants, confirmed these observations and also allowed us to obtain further in-depth understanding of the rationale behind some innovative use practices (Fig 6). Furthermore, similar assessment of a sandal format of emanator prototype [46] in Brazil yielded similar contrasts, with generally encouraging feedback from end users in the community, whereas experimentally controlled entomological assessments in the same setting indicated only modest and inconsistent levels of protection at best (Alvaro Eiras, Personal communication). So, while social science assessments of repellent product use may sometimes be prone to sometimes extreme subjective biases [14], some of which may well be introduced or exacerbated by the investigators themselves [44], the authors could not identify any unambiguous reason to disregard the positive views commonly expressed by community participants in this study.

### Conclusions

Overall, participants generally viewed emanators positively and outlined several advantages over current alternatives, although some expressed concerns about smell, health hazards, bulkiness, unattractiveness and future cost (Table 2). Many participants expressed moderate to high satisfaction with protection against mosquitoes (Fig 3A and 3B), especially indoors. Protection against other arthropod pests was also commonly reported, although satisfaction levels were highly variable (Fig 3C and 3D). Diverse use practices were reported (Figs 4 and 5), some of which probably targeted nocturnal *Culex* resting indoors, rather than *Aedes* attacking them outdoors during daylight hours (Fig 6). One other use practice was identified, in which an infant was placed on the ground with its head within centimetres of the device (Fig 7), that could be hazardous in terms of potential pulmonary exposure to transfluthrin vapour if higher treatment doses were used [33]. Perceived durability of protection varied: While some participants noted some slow loss over months, others noted rapid decline within days, and a few associated efficacy loss with outdoor use and exposure to wind, sunlight or moisture. Many expressed stringent expectations of satisfactory protection levels, with even a single mosquito bite considered unsatisfactory. Emanators were frequently considered superior to fans, bedsheets, sprays and coils, but it is concerning that some preferred them to bed nets and consequently stopped using the latter.

Having said all that, it is not clear why the broadly encouraging results reported herein from sociological assessment of transfluthrin emanators among end users in Haiti, as well as recent epidemiological assessments from Peru [5], differ so much from those of entomological assessments across Haiti [26], Tanzania [33], Brazil (Alvaro Eiras, Personal communication) and Peru [5], all of which indicate little if any protection against *Aedes* mosquitoes. While incongruence of results obtained with these distinct and complementary evaluation approaches remains to be resolved, and substantive risks of confirmation bias are obvious in relation to this qualitative study, no unambiguous reason could be identified to doubt the generally encouraging views expressed by Haitian end-users of transfluthrin emanators. It is concluded that the community end user perspectives reported herein should be interpreted at face value until reliable evidence to the contrary emerges.

### Footnotes

^1^ While community use of the word *Placatox* refers to specific mosquito repellent coil product, manufactured by Plagotox S.A. of Villa Nueva, Guatemalla, which is commonly used by households in Port-au-Prince, this term is often used generically to refer to mosquito coils of any brand or specification.

^2^ While community use of the word *Baygon* refers to specific mosquito repellent coil product, manufactured by S.C Johnson & Son. of Racine, Wisconsin, United States of America, which is commonly used by households in Port-au-Prince, this term is often used generically to refer to mosquito coils of any brand or specification.

^3^ Haitian joke, depicting someone who behaves in a self-important manner.

### Supporting information

S1 Checklist *PLOS ONE* clinical studies checklist. (DOCX)

S2 Checklist COREQ (COnsolidated criteria for REporting Qualitative research) checklist. (PDF)

S1 Data An anonymized form of all the questionnaire data used to generate Fig 3. (XLSX)

S2 Data An anonymized form of all the IDI transcripts, in the original Haitian Creole, that were analysed (Table 2) and selectively translated for quotation in the *Results* section. (DOCX)

S3 Data An anonymized form of all the conventional FGD transcripts and PV-FGD transcripts, in the original Haitian Creole, that were analysed (Table 2) and selectively translated for quotation in the *Results* section. (DOCX)

## Abbreviations

PNCM: Programme National de Contrôle de la Malaria of the Ministère de la Santé Publique et de la Population in the Republic of Haiti
FGD: Focus Group Discussion
IDI: In Depth Interview/
PV: Photovoice methodology [13, 27–30]
PV-FGD: Photovoice Focus Group Discussion
