# Supplementary material for: Community end user perceptions of hessian fabric transfluthrin vapour emanators for protecting against mosquitoes under conditions of routine use in Port-au-Prince, Haiti
Source: PLoS One. 2024 Jul 10;19(7):e0300368. doi: 10.1371/journal.pone.0300368 (PMC11236173; doi:10.1371/journal.pone.0300368)
Supplement: S2 Data — (DOCX) [file pone.0300368.s004.docx]

**TEAZ Project Haiti – Social Science Data**

**Transcripts of individual In-Depth Interviews (IDIs)**

Rounds 1 & 2 - Blocks 1 to 4 (August and December 2018)

Round 3 - Blocks 5 and 6 (May 2019)

[IDI Block 1 4](#_Toc153896730)

[Household 1 / member HM6 Male / Round 1 4](#_Toc153896732)

[Household 2 / member HM1 Male / Round 1 (Excluded) 8](#_Toc153896733)

[Household 2 / member HM2 Male / Round 1 11](#_Toc153896734)

[Household 2 / member HM3 Female / Round 1 (Excluded) 13](#_Toc153896735)

[Household 1 / member HM5 Female / Round 2 15](#_Toc153896736)

[Household 1 / member HM6 Male / Round 2 20](#_Toc153896737)

[Household 2 / member HM2 Male / Round 2 22](#_Toc153896738)

[Household 2 / member HM3 Female / Round 2 (Excluded) 24](#_Toc153896739)

[Household 2 / member HM8 Male / Round 2 29](#_Toc153896740)

[Household 2 / member HM12 Male / Round 2 31](#_Toc153896741)

[Household 3 / member HM9 Female / Round 2 33](#_Toc153896743)

[Household 3 / member HM10 Male / Round 2 34](#_Toc153896744)

[IDI Block 2 36](#_Toc153896745)

[Household 1 / member HM14 Male / Round 1 36](#_Toc153896746)

[Household 1 / member HM21 Male / Round 1 39](#_Toc153896747)

[Household 1 / member HM22 Female / Round 1 40](#_Toc153896748)

[Household 2 / member HM20 / Round 1 42](#_Toc153896749)

[Household 3 / member HM17 Female / Round 1 44](#_Toc153896750)

[Household 3 / member HM18 Male / Round 1 46](#_Toc153896751)

[Household 1 / member HM16 Female / Round 2 47](#_Toc153896752)

[Household 1 / member HM22 Female / Round 2 49](#_Toc153896753)

[Household 1 / member HM13 Male / Round 2 52](#_Toc153896754)

[Household 1 / member HM14 Male / Round 2 54](#_Toc153896755)

[Household 2 / member HM15 Female / Round 2 57](#_Toc153896756)

[Household 2 / member HM19 Male / Round 2 60](#_Toc153896757)

[Household 3 / member HM17 Female / Round 2 63](#_Toc153896758)

[IDI Block 3 65](#_Toc153896759)

[Household 1 / member HM26 Male / Round 2 65](#_Toc153896760)

[Household 1 / member HM30 Male / Round 2 68](#_Toc153896761)

[Household 2 / member HM27 Male / Round 2 71](#_Toc153896762)

[Household 3 / member HM29 Male / Round 2 73](#_Toc153896763)

[Household 3 / member HM28 Female / Round 2 76](#_Toc153896764)

[Household 3 / member HM25 Male / Round 2 78](#_Toc153896765)

[IDI Block 4 80](#_Toc153896766)

[Household 1 / member HM31 Female / Round 2 80](#_Toc153896767)

[Household 1 / member HM34 Male / Round 2 84](#_Toc153896768)

[Household 2 / member HM32 Male / Round 2 86](#_Toc153896769)

[Household 2 / member HM35 Male / Round 2 89](#_Toc153896770)

[Household 3 / member HM33 Male / Round 2 92](#_Toc153896771)

[IDI Block 5 94](#_Toc153896772)

[Household 1 / member HM36 Female / Round 3 94](#_Toc153896773)

[Household 2 / member HM37 Male / Round 3 95](#_Toc153896774)

[Household 3 / member HM38 Female / Round 3 97](#_Toc153896775)

[Household 4 / member HM39 Female / Round 3 99](#_Toc153896776)

[IDI Block 6 102](#_Toc153896777)

[Household 1 / member HM43 / Round 3 102](#_Toc153896778)

[Household 2 / member HM41 Male / Round 3 104](#_Toc153896779)

[Household 3 / member HM40 Female / Round 3 106](#_Toc153896780)

[Household 4 / member HM42 Male / Round 3 108](#_Toc153896781)

# IDI Block 1

## Household 1 / member HM6 Male / Round 1

ITV: Isi sa se kay ki kote, kay…nimero kay la, nou pap met nimero nap mete kay ou, kay kwa, nap mete kay jilyana… kay kiyès nap di isi a.

Enfòmatè: La se kay… kay HM6

ITV: La se Kay M. ok, M. komanl ekril konsa

Infomate: HM6

ITV: HM6 ok

HM6: Non an se HM6

OD Sex. maskilen …dat la se 20 out…

HM6: 21 out

ITV: Oke 21 out 2018.

Oke nap premye… nap komanse

Nan ki nivo ou panse emanatè a pwotejew kont marengwen kap modew lèw nan kay la?

Eksplike nou… nan koman sa pwotejew lew nan kay la?… nan ki nivo?

HM6: Bon… Komsi anvan… anvan potko gen emanatè a, mka di gen yon ti amelyorasyon ki fèt tou piti, menm si li pa elimine yo konplètman… toujou genyen

ITV: Dakò, lèw konpare avan ou wè te gen plis moustik men lèw vin ak emanatè a vin gen ti amelyorasyon?

HM6: Wi

ITV: Dakò

Eskew ka bay plis detay? Eske se mwens moustik ki modew oubyen kòman ou ka esplike sa?

HM6: Bon mka di gen… se mwens ki mòde

ITV: Dakò

Oke fè yon ti wonn nan youn nan repons ki pi ba yo oswa skrèt nòt ki pi ba a pou di bon… pou chwazi kisa ki apwoprye pou ou. Eske li pa pwotejew, eskel pwotejew tou piti, eske li byen, eske Li trè byen, eske li pwoteje nèt. Wap chwazi youn nan ti rektang sa: li pa pwoteje. lipwoteje tou piti…

HM6: Mtap pran de a

ITV: Tou piti

HM6: Wi

ITV: Ebyen vwala chwazi

HM6:: fonw…Dakò

ITV: Oke nan ki nivo pou emanatè a pwotejew ak lot moun nan kay ou a kont lot vèmin… kont lòt vèmin yo lèw nan kay la?

Fè yon ti wonn nan youn nan repons ki pi ba yo oswa sèke nòt ki anba, si pa gen repons wap di.. Nan ki nivo pou emanatè a… pwotejew ak lòt moun nan kay ou a kont lot vèmin yo lè yo nan kay la?

HM6: Bon.. donk janm ta vle li ye?

ITV:Wi…

HM6:bon fèl pwoteje nèt

ITV: Bon vwala!

ITV: Nan ki nivo emanatè a pwotejew kont marengwen lèw deyò nan lakou a? Nan ki nivo ou santil pwotejew?

Fè yon ti wonn nan youn nan repons ki pi ba yo oswa se ki nòt ki anba a, si pa gen okenn repons tou, wap chwazi pa gen okenn repons, wap di lèw deyò nan ki nivol pwotejew.

HM6: Bon mpa konn itilize l deyò non, se toujou anndan.

ITV: Se toujou andan ou itilizel? Ou pa konn itilizel deyò?

Oke…dakò wap koche ou pa gen repons oubyen wap di ou pa konn itilizel anndan. Oke!

ITV: se toujou menm fòm kesyon yo. Nan ki nivo emanatè a pwotejew ak fanmiw kont lòt vèmin, lèw deyò kay la? Fè yon ti wonn nan youn nan repns ki pi ba yo oswa sèke not ki anba a. Donk ou te di talè a ou pa konn…nan ki nivo emanatè a pwoteje w avèk moun nan kay la kont lot vèmin, lòt vye bagay ki lèw deyò kay la. Eskew santil pwotejew? Hen?

lèw deyò ka la? Ou di ou pa konn itilizel deyò?

HM6: non

ITV: Dakò! men lèw deyò kay la ou pa santi okenn pwoteksyon? Ou santi se sèlman andan li pwotejew? Se sèlman anndan l pwotejew? Ou santil pwotejew?

lèw anndan kay la sèlman men lèw deyò ou pa santi okenn pwoteksyon?

HM6: non

ITV: Ebyen wap koche youn nan repons sa yo.

Pòz

ITV: Wap bay kotew ye

HM6:Wi mwen wè ki kotem ye men mpa konn itilizel deyò.

ITV: ou pa konn itilize l deyò?

HM6:non

ITV: Site kat (4)…epala e kat (4). Nan ki nivo emanatè a pwotejew ak fanmiw kont lòt vèmin lè ou deyò kay la? Eske lè ou deyò kay la, ou santi emanatè a pwotejew menm lèw pa metel deyò?

HM6: An.. oke bon…mka di non

ITV: li pa pwotejew deyò? Ou pa santi sa?

HM6: non

ITV: Eben wap di li pa pwotejew …wap chwazi.

ITV: Site ak dekri nenpòt lòt benefis oswa dezavantaj ke emanatè a bay. Ou mèt pale aprè sa wa ekri.

Dekri…bay kèk benefis oubyen dezavantaj ki bay benefis yo kounya.

HM6: Bon! Benefis lan sèke anvan, nou te konn jwenn anpil moustik andann an, men avèk emanate a li… mka di mwen jwenn mwens pa rapò avèk avan.

ITV: Dakò! Donk sa sonw benefis pou ou …e ki dezavantaj ou wè ki genyen si ou wè?

HM6: Mpa vrèman wè dezavantaj non.

ITV: Dakò! Eben ekri sa la, saw sot di a, wap ekril oubyen siw gen lòt ti bagay ou ka di ou ka ekril…

HM6: Mka ekril anba a la?

ITV: Wi Wi

ITV: Oke mèsi nap kontinye ak kesyon sis (6). Klase enpòtans avantaj, dezavantaj sa pa youn nan repons ki anba yo oswa nenpot nan bagay sa yo pa aplike oswa pa gen repons: gwo dezavantaj pa bon menm, de tit avantaj pa tro mal, dezavantaj fèb, avantaj fèb ti avantaj, gwo avantaj. Komsi wap klase enpòtans avantaj yo ak enpòtans dezavantaj yo. Wap chwazi vwala!

Mpanse se yon kwa wap fè sou… wap wè si gen gwo avantaj, si se yon ti zavantaj, si pa gen avantaj ditou wap koche la gwo dezavantaj pa bon menm, ti avantaj pa tro mal, dezavan…

Kisa wap pran eske se gwo avantaj oubyen eske wap pran ti dezavantaj mpa konnen.

HM6: Kom dezavantaj kisam ta di… kom dezavantaj sèke sim…komsim ta metel bò yon kabann sa ka rive tankou sèm nan konn di… bon gen pwodwi… yo di yo tretel avèk pwodwi…li ka pè komsi lèm metel tro pre poul pa touche avèl.

ITV: An mkonprann li pè poul pa touche avèl. Anh..Dakò

HM6: Nou ka di sa kòm ti dezavantaj mta ka pran tou de sa yo…

ITV: Eben fè li

HM6: Mka pran tou de?

ITV: Mpa konnen, wi!.ou tap pran ti avantaj?

HM6: Mtap pran avantaj fèb

ITV: Dakò! Avantaj fèb la sa vle di se yon bagay ki bon ki pa tro efikas epou dezavantaj? Ou pran dezavantaj fèb?

HM6: Wi

ITV: Eben dakò donk li di li pè eske w santi ou men sa gen yon enpak sou santew?

HM6: Bon pa gen anyen non

ITV: Men poukisa ou pran avantaj fèb?

HM6: Bon paske se sa sèlman…

ITV: Hen?

HM6: Se sa sèlman se… se pè li… komsi nou pa vle trò touche avèl …menm menmenm lèm map kenbel tou se nan tèt anlè, mpa trop kenbel nan kote… nan bagay ki tankou sak la… menl pa vrèman fè okenn efè, okenn enpak sou sante nou.

ITV: Ou pa santi sa?

HM6: wi

ITV: Site ak dekri nenpot lòt benefis oswa dezavantaj emanatè a bay? Ou ka di yo la, paskew ekri yo, ou met pale.

HM6: Site ak dekri nenpot ki benefis…..Oubyen dezavantaj. Ki benefis…

ITV: Se komsi se saw sot di yo la, yo mande w esplikasyon.

HM6: Dezavantaj yo sèke se sam sot diw lan, nou pa vle akòz pwodwi yo met ladann lan gen yon enpak sou sante nou men avrèdi li pa janm fè enpak vre.

ITV: Men eske w gen yon ide de pwodwi ki gen ladann yo?

HM6: Non mpa konnen

ITV: Yo pat diw anyen sou sa?

HM6: Yo pat dim anyen

ITV: Se sa ou ka we pou dezavantaj? E pou avantaj benefis?

HM6: Benefis sèke to marengwen an diminye menm sil pa diminye konplètman, genyen toujou men li te pi mal avan.

ITV: Ebyen ekri sa la byen vit pounn ka kontinye.

ITV: Ok ou fini nap kontinye avèk kesyon yo, kesyon wit (8) la. Klase enpotans avantaj, dezavantaj saa pa youn nan repons ki anba yo oswa nenpot nan bagay sa yo pa aplike, oswa pa gen repons vwala!:

Wap klase yo toujou se si gen yon repetisyon nan 1-gwo dezavantaj, pa bon menm, 2-ti zavantaj pa tro mal,.3- ti zavantaj fèb 4-avantaj fèb 5-ti avantaj , gwo avantaj, wap klase wap chwazi.

ITV: Mentnan pou dezavantaj. Site ak dekri nenpot lot benefis oswa dezavantaj emanatò a bay nan saw pat di avan la? Ki lòt benefis ou wè li bay e answit ki dezavantaj dabò benefis. Ki benefis ou wè li pote nan kay la?

HM6: Lòt benefis…mpa tro wè sam te ka di…

ITV: Ou pa wè lòt benefis apa saw sòt di yo la?

HM6: Non

ITV: Dakò men epou dezavantaj. Ki dezavantaj li pote? li bay?

HM6: Dezavantaj sèke …mta di menm sim pa sèvi avè l mka toujou kreye yon espas poum ka metel. Wè paskem we li pran espas.

ITV: Li toujou bezwen espas …Se byen saw di a.

Poz

ITV: Eben ou pa gen yon ide de lòt benefis li pote nan kay la, emanatè a?

HM6 Non

ITV: Dakò! Men siw gen yon ide ou kadil menm aprè antretyen an… aprè transkripsyon an paske se pat trò ta. 10- Oke klase enpotans avantaj dezavantaj sa pa youn nan repons ki anba yo oswa nenpot nan bagay sa yo.M panse se menm bagay yo ki ….

HM6: se kom mpa di avantaj yo map chwazi dezavantaj selman

ITV:Bon oui paske se sa ou obseve , se sa ou viv.E pou avantaj?

HM6 Kom mpa vreman te di ki avantaj

ITV: An okeMen la ou te chwazi ti avantaj…Mgen lenpresyon se menm kesyon yo repete plizye fwa pou yo wè siw gen koerans …Klase enpotans avantaj, dezavantaj sa fòw fèl pou tou de la, fow fèl pou avantaj tou, wap chwazi youn nan repons sa yo pouw klase sa vle di wap wè dapre ou… men si li bay gwo avantaj oswa li bay yon ti avantaj oswa avantaj lan fèb etc…

Eske pa gen anyen ou ta di sou bagay emanate a, pa gen anyen te ka di?

HM6: Bon mka di pou … kom taleu a mte pran nan …kijanm ta vle emanatè a ye…Bon mta vle pou lè komsi nou genyen yo …pou yo komsi pi efikas pou lè nou mete yo on kote pounn preske pa jwenn marengwen, pou lè nap itilizel.

ITV: Dakò! Ebyen Mèsi anpil HM6, map femen aparèy yo ,ou mèt rele sèw la vin ranplasew.

## Household 2 / member HM1 Male / Round 1 (Excluded)

OD (Obrillant Damus): Nou pral kòmanse la, ou okipe paske ou ap travay, nan ki nivo, HM1 ou panse emanatè a pwoteje ou kont marengwen k’ap mὸde ou lè ou nan kay la? Yo pwopoze ou plizyè repons, ou ap chwazi repons ki bon pou ou, èske li pa pwoteje ou ou ap pran 1, èske tou piti si nwi ou ap pran 2, byen ou ap pran 3, si se trè byen ou ap rpan 4, si se pwoteje nèt si wi ou ap pran senk, ou ap chwazi repons ki apwopriye pou ou a, nan ki nivo li pwoteje ou.

Dakò dezyèm kesyon an, nan ki nivo pou emanatè a ta pwoteje ou ak lὸt moun lakay ou kont lòt vèmin yo lè ou nan kay la? Nan ki nivo pou l pwoteje ou? Epi ou ap chwazi ki repons ki apwopriye pou ou.

SP (supervisor): Kesyon an mal poze wi.

ITV: Kòman ou te renmen kominike l?

ITV2: Nan ki nivo ou panse emanatè a ta dwe pwoteje ou.

ITV: Vwala ta dwe , trè byen.

ITV2: Se kòmsi yo ta ba ou l o konsidyonèl pa vre?

ITV: Vwala. Se sa menm. Nan ki nivo ou ta renmen li pwoteje ou?

ITV2: Wi.

ITV: Oke, dezyèm kesyon se deja oke, twazyèm kesyon nan ki nivo emanatè a pwoteje ou kont marengwen ,lè ou deyò nan lakou a? Nan ki nivo?

HM1: Byen.

ITV: Ebyen chwazi repons ou , vwala, oke, nan ki nivo pou emanatè a pwoteje ou ak fanmi ou kont lòt vèmin yo, lè ou deyò nan lakou a? Nan ki nivo, trè byen nou nan kesyon senk la pa vre? M espere m pa sote kesyon sa paske yo mal mete l. Manke espas ant kat ak senk la, site ak dekri nenpòt lòt benefis oswa dezavantaj ke emanatè a bay? Ki lòt benefis li bay?

HM1: Pou benefis lan m kapab di, menm bagay madam HM3 te di talè a, emanatè a pèmèt m pa achte blakatòks ankò, li ede m nan sans sa, kisa blakatòks la te konn fè m, li konn fè m fè depans, men avèk emanatè a m pa janm fè depans ankò. L a li ede mwen san ke m pa depanse on goud.

ITV: Ki dezavantaj ou wè l bay, daprè oumenm? Èske gen dezavantaj?

HM1: Jiskaprezan, m poko wè dezavantaj emanatè an bay, m poko wè dezavantaj.

ITV: Èske ou vle ekri sa ou di yo la an kreyòl touswit la?

ITV2: Ou di ou pa wè dezavantaj?

HM1: Non.

HM2: M ka pale kounya?

ITV: Wi, ou se mesye ki sot patisipe talè a.

HM2: Mwen sa m ta renmen yo fè, m ta renmen yo pran jan ke yo vin avè l la, pou yo pa ta fè bak avè l, pou yo ta renfòse l plis,pou tout kote ta jwenn li, paske gen kote ki poko janm konnen kòman sa rele.

ITV: Ou bezen l se on bagay ki popilè?

HM2: Wi. Paske l’ap bon pou tout moun, paske jan m wè l bon pou mwen an, m ta renmen tout an pwovens , tout kote moun ap malad, yo gendwa pa konn sa yo genyen, men si chak kote zòn ta kòmanse fè l,yo ta mete l tout kote nèt moun t’ap santi yo soulaje, paske se pa sèl pòtoprens pou yo ta rete avè l, gen moun an pwovens marengwen ap manje yo konsa tou, y opa konn ki maladi yo pran, y’al lopital fasil, kote ke m ta renmen yo ta pase men pòtapiman, okay, tigwav tout kote nan peyi a pou yo ta pase avè l.

ITV2: Pou l vin on pwogram nasyonal.

HM2: Pou l vin on pwogram nasyonal, pou tout moun ta satisfè.

ITV: Dakò.

HM1: M plis wè emanatè a pa gen dezavantaj ladan paske lè m rive o nivo ni begond ni balakatòks, lè m te konn flite begond nan kay lan alepòk, nou te konn oblije soti, odè a konn tèlman fò, ni timoun ni madanm nan nou tout soti nou bay kay la pou odè a, men avèk emanatè a pa gen sa.

ITV2: Depi ou itilize?

HM1: Begond avèk blakatòks, siitou begond lan, se soti nou soti kite odè a degaje avan nou antre nan kay la.

ITV2: Mwen di, mwen pa wè dezavantaj emanatè a bay okontrè li banm plis avantaj ke tout lòt yo, mwen pa gen pou m achte begond avèk blakatòks, lè pou itilize begond fòk mwen kite kay la se sa ou te di wi?

HM1: Wi.

ITV2: Blakatòks la bay lafimen ki pa bon pou sante pa vrè? Emanatè m mèt rete toupre l, li pa fè m anyen.

ITV: Dakò, mèsi anpil n’ap kontinye avèk mesye HM1, nou nan kesyon sis la, klase enpòtans avantaj sa ou sot di yo la,pa youn nan repons ki anba yo, chwazi youn nan repons ki anba yo pou klase avantaj sa yo. Èske se on gwo avantaj ,èske se on ti avanataj  emanatè a bay?

HM1: Gwo avantaj.

ITV: Sèt la, site ak dekri nenpòt lòt benefis oswa dezavantaj emanatè a bay? Si ou konnen li gen on lòt benefis li bay di sa.Si gen on lòt dezavantaj li bay di sa, nenpòt bagay.

HM1: Lòt benefis la m sot reponn li nan kesyon 5 lan, kote ke ekonomi ke m fè m pa achte ni begond, ni balaktòks ankò. M pa wè dezavantaj, paske m konn achte blakatòks,kote rad yo anndan fin gen sant balaktòks la,begond nan tou lè m flite l menm nan rad yo odè begond nan konn rete avèk emanatè a pa gen sa.

ITV: M konprann.

HM1: Si ta gen dezavantaj se pwodui m konn achte alepòk yo,avan yo ki pat bon pou mwen.

ITV: Oke ou vle ekri sa yo la?

HM1: M’ap ekri yo?

ITV: Wi.

HM1: Bare li?

ITV: Non pa bare, ekri sa ou sot di yo la,repons la, sa ou sot yo la ou ap redi yo, epi l’ap ekri pou ou.

ITV2: M te tande wi, pwen sèt?

ITV: Wi dakò.

ITV2: Pwodui m te konn itilize avan yo, yo te konn degaje odè, kounya , lòt pwodui yo te konn degaje vye odè pa vre?

HM1: Wi.

ITV2: E nui sante m. M ekri l wi.

ITV: Oke, mèsi anpil n’ap kontinye HM1, avantaj sa yo ou sot di yo la, ou ap chwazi youn nan repons sa yo pou klase yo.

HM1: M kapab di gwo, li se on gwo avantaj.

ITV: Dakò. Sa bay gwo avantaj, kesyon nèf la ki menm bagay avèk sis la prèske, site ak dekri nenpòt lòt benefis oswa dezavantaj emanatè a bay? Se prèske menm bagay.

ITV2: Li pèmèt mwen sere lajn, lajan blakatòks la m sere, m sere lajan.

ITV: Bon, se HM1 ki pou pale, se HM1.

HM1: M fè ekonomi, emenatè a pèmèt m fè ekonomi, m pa itilize kòb ankò pou m achte dè pwodui avèk emanatè an tout bagay anfòm.

ITV: Dakò, se avantaj sa ou sot di a la klase enpòtans li nan sa.

HM1: Li se on gwo avantaj.

ITV: Vwala, la nou fini,,nou twa kisa nou ta renmen di, èske nou wè gen dezavantaj, fòk nou pa ezite di sa paske se pa on pwoblèm, sa p’ap kont ou si ou di gen dezavantaj.

ITV2: Ki dezavantaj li genyen se poutèt bagay kesyon ou pa ka kite l kot timoun, apresa m pa wè lòt, sa sèlman

ITV: Oke, se byen.

HM1: Sèl sa timoun pa ka manyen l, poukisa timoun pa ka mayen li?

ITV2: Paske transfritrin nan se on pwazon li ye, m panse sa, menm jan li ka detui ensèk la,m panse si timoun nanmanyen l epi li mete men nan bouch li li kapab anpwazone l, se sèl dezavantaj sa wi. Paske m oblije ap veye l avèk Zeyila.

ITV: Ou pa gen lòt dezavantaj ankò ou wè?

ITV2: Non.

ITV: Dakò.

ITV2: A mwens ke li gen efè segondè a.

ITV: Ke ou pa konnen?

ITV2: Ke m pa konnen , tout sa ke ou inyore ou pa ka pale de li.

ITV: Oke ,mèsi anpil se on bon antretyen nou sot fè la, ki endividyèl,twa antrteyn, men li gen on aspè koloktif paske nou tout pale ansanm lè n’ap fè entèvyou a nou pale dedezavnatj ki trè minim, mèsi pou atansyon nou, nou prale nan twazyèm fwaye a HM3 ap mennen nou, epi nou ta vle fè ak ou tou ou se….

ITV2: Sipèvizè.

ITV: Vwala.

## Household 2 / member HM2 Male / Round 1

ITV: Kounya mesye HM2, nou pral fè on ti pale, mèsi pou patisipasyon ou davans, nan ki nivo ou panse emanatè a pwoteje ou kont marengwen k’ap mὸde ou lè ou nan kay la? Men repons yo la, si li pwoteje ou nèt ou ap di senk, si se trè byen ou ap di kat, ou ap chwazi youn nan repons sa yo. Eseye patisipe, kòm se madanm li, l’ap di sak , repons ki apwopriye , epi vwala.

ITV2: Pwoteje nèt.

ITV: Ebyen, fè on kwa sou li,vwala. Ebyen de a, nan ki nivo pou emanatè a pwoteje ou ak lὸt moun nan kay ou kont lòt vèmin yo, lè ou nan kay la? Nan ki nivo pou l ta pwoteje ou? Dakò, misye pa nan pale anpil limenm.

Twa, nan ki nivo emanatè a pwoteje ou kont marengwen, lè ou deyò nan lakou a? Ou ap fè on tiwonn oubyen on kwa sou youn nan repons sa yo ki apwopriye, ou wè l pwoteje ou, nan ki nivo li pwoteje ou? Lè ou sou lakou a èske se byen, trè byen?

Kat, nan ki nivo pou emanatè a pwoteje ou ak fanmi ou kont lòt vèmin yo, lè ou deyò nan lakou a?

Sis, klase enpòtans avantaj, dezavantaj sa pa youn ki nan repons anba yo. Gen on kesyon ki sote, mpoko wè l, gen youn ki sote vrèman.

Senk, site ak dekri nenpòt lòt benefis oswa dezavantaj ke emanatè a bay?

HM2: M gentan ekri ladan l deja.

ITV: Ou ka ekri la, ou di l tou wi, ki lòt benefis li bay emanatè a?

HM2: Lòt benefis li bay, jan marengwen te konn ye nan kay la, nou vin pa jwenn li konsa ankò,li vin on lòt avantaj pou nou, paske se kote ou plase l la, si ou wè gen marengwen ou plase l, si ou ap gade tan, jan tan ye, si ou te gen dlo on kote ki gen, ki ka fè moustik, ou jete tout dlo, tout bagay ki ka fè moustik la,pou pwoteje ni aparèy la ki kont li a tou, pou ede tèt ou.

ITV: M konprann men, èske ou wè gen dezavantaj li bay, emanatè a?

HM2: Bon, nòmalman m pa wè li bay on dezavantaj non, paske m wè l bay on avantaj, paske pou m te achte blakatòks,mwen pran sans kote bò marengwen yo ye a, kote m tande yo a, m jis plase l epi m vin wè li vin pi mye.

ITV: M konprann, èske ou vle ekri sa ou di yo la oubyen n’ap kontinye? Ou vle ekri kèk bagay an kreyòl?

HM2: M pa konnen.

ITV: Ou vle di sa l di yo oubyen ekri yo pou li?

ITV2: Ou pa ekri kreyòl? Se fransè nou ekri?

ITV: Kreyòl nou ekri. Se kreyòl nou pale.

ITV2: Site oswa dekri lòt avantaj emanatè a bay?

ITV: Bon, li pat di anpil bagay non , m panse ou gentan fini, pa ajoute plis non. Kounya nou prale nan suivan ,sa li sot di yo la, ou ap klase enpòtans avantaj , dezavantaj sa pa youn nan repons ki anba yo, oswa pa nenpòt bagay sa yo, si ou te pale de avantaj ou ap di èske se on gwo avantaj , ti avantaj ki fèb, ou ap kwoche , vwala. Si ou ap pale de dezavantaj…

Sèt, site ak dekri nenpòt lòt benefis emanatè a bay, ki lòt benefis ou wè l bay, apa sa ou sot di yo la?

HM2: Lòt benefis , mwen wè l bay, pa egzanp, si ou te leve nan maten ou te depoze l nan chanm sa epi ou depoze l on lòt kote pou l ka alwaye bagay ki genyen yo.

ITV: Ki dezavantaj ou wè li bay?

HM2: M vin pa egzakteman wè marengwen ankò.

ITV: Dakò,ebyen ann ale, uit la klase avantaj sa ou sot di yo la, pa youn nan repons ki anba yo, ou ap chwazi.

ITV2: Doktè Damus m ka fè on ti li papye l la stp?

ITV: Wi. Nou nan kesyon nèf kounya, site ak dekri nenpòt lòt benefis oswa dezavantaj ke emanatè a bay, ki lòt benefis ou wè l bay?

HM2: Lòt benefis m wè l bay pa egzanp si ou wè solèy la move konsa, te gen, tankou lè ou leve le maten, gen marengwen ki vin dòmi, genyen tou k’ap soti ki prale se tankou se on wout yo sot fè, men pandan ke ou depoze l, genyen ki tou ret deyò, ki tou pa antre.

ITV: M konprann, li chase yo, oke, sa se sa ou sot di yo la, klase enpòtans avantaj, dezavantaj sa nan youn nan repons ki anba yo, jan ou te konn fè l pou lòt la, èske se on gwo avantaj, èske se on ti avantaj ou wè l ye?

HM2: On gwo avantaj.

ITV: Ebyen, èske ou gen lòt bagay ou te vle di sou emanatè a sou fason ou itilize l nan kay la? Èske oumen HM3 na tou gen on bagay ou te anvi di, ke ou pat di?

ITV2: Non.

ITV: Ebyen, mèsi anpil, nou pral pran twazyèm moun nan nan de minit.

## Household 2 / member HM3 Female / Round 1 (Excluded)

ITV: HM3, se on plezi pou nou ansanm jodya pou n fè on ti koze sou aparèy ke ou ap itilize depi kèk tan, emanatè a.Nou prezan maten la pou nou wè nan ki mezi nou kapab evalye aparèy la dakò?

HM3: Wi.

ITV: HM3 nan ki nivo ou panse aparèy la pwoteje ou kont marengwen k’ap mòde ou anndan kay la?

HM3: Bon dòzedeja mèsi, m kontan resevwa ou, m fyè tou lefèt ke m se youn nan patisipan pwojè TEAZ, non pa m se HM3, m kapab di emanatè a li jwe yon wòl vrèman enpòtan nan sante nou nan kay la. Paske otrefwa nou te gen anpil marengwen la, men depi lè pwojè TEAZ ateri nan zòn na yo te ban mwen de emanatè ke se pa achte m te achte yo, yo te fè m kado. Epui piske se te nan staj pilòt yo t’ap gade pou yo wè efikasite emanatè a, mwen gendwa di ke emanatè a li vrèman bon, paske otrefwa kantite marengwen m te genyen kounya m chita alèz san pwoblèm. Ou ap gade pou wè nan salon an la m enstale yo, kote ke nan salon an m pa wè marengwen ditou, e menm nan chanm ki te plen marengwen m pa konnen èske transfritrin ki ladann nan , èske kantite a li adekwat paske menm nan chanm nan konn gen marengwen m pa prèske wè depi emanatè a anndan kay la, m kapab di ke se pa on mens afè, emanatè a vrèman bon.

ITV: Donk li byen pwoteje? Trè byen pwoteje? Pwoteje nèt?

HM3: Trè byen pwoteje.

ITV: Nan ki nivo ou panse aparèy la pwoteje ou ak lòt moun ki nan kay la , lè ou anndan kay la?

HM3: Bon, m pa ka esplike nivo a, paske otrefwa nou pat ka dòmi, lè pou n dòmi n’ap veye kouran paske otomatikman ke pa gen kouran, ou oblije fè lè deyò, paske ou pat kapab vrèman sipòte avèk marengwen, non sèlman yo mòde ou , yo chante nan zòrèy ou epitou yo te kapab bay maladi, m sonje tout moun isi a te gen chikounkounya ke se on maladi ki sòti nan moustik, men depi ke nou gen emanatè a nou pa prèske gen okenn pwoblèm, nou pa gen dout ni pou n tounen panse ke n pral gen ni fyèv marengwen bay, nou pa gen dout ke nou pral pran kenn risk, nou jis dòmi ke gen kouran, ke pa gen kouran, nou fonksyone alèz nou dòmi alèz bon m panse ke nivo a nou pa ka vrèman esplike l.

ITV: Oke men lè ou deyò kay la , èske li pwoteje ou? Lè ou nan lakou a li pwoteje ou avèk fanmi ou kont marengwen?

HM3: Nòmalman si m deyò a, piske deyò a li pa pwoteje pa emanatè a,m bezwen chita fè on nonb de tan deyò , m deplase avèk emanatè m, m chita , m mete emanatè m nan direksyon m panse ke moustik lan kapab vini , direksyon m santi moustik la, epi m chita deyò a alèz. Epi avantaj ke m wè amanatè a genyen, se paske li pòtatif, m gendwa di m pral an pwovens, mwen byen anbale l epi m’ale avèk li kèlkeswa kote m prale m ka deplase avèk li se avantaj sa plis li genyen.

ITV: Ki lòt avantaj ou wè li gen ankò?

HM3: Avantaj li genyen se sou kesyon sante a, kounyeya menm lè ou ta gen on maladi moustik bay m pa gen ankenn krent paske m konnen ke lakay mwen pwoteje avèk emanatè a.

ITV: Ki dezavantaj?

HM3: Dezavantaj li genyen an, tout moun konnen l se paske se pa on materyèl ki ka rete kote timoun ye, pou timoun pa manyen l se sèlman dezavantaj sa li genyen, kòmsi si yo te ka fè li kote ke li gen on izolan, kòmsi menm lè l’ap fonksyone anndan kay la on timoun ta manyen li p’ap gen pwoblèm se ke li t’ap a sanpousan bon se sèl dezavantaj li genyeen pou timoun.

ITV: HM3 si n te ka fè on klasman gwo dezavantaj, ti dezavanyaj, gwo avantaj, ti avantaj kisa ou t’ap chwazi ladan yo? Si n te ka klase enpotans avantaj yo, dezavantaj yo.

HM3: M kapab di ti dezavantaj li genyen, dezavantaj la pa twò enòm, paske mwen menm m panse kwoke l nan mi, on fason on otè pou timoun pa jwenn li. Se on ti dezavantaj li ye , men se pa on gwo dezavantaj li ye vrèman ansòt.

ITV: Dakò, ki benefis aparèy la pote pou ou? Ki bebefis ou jwenn?

HM3: Li fè m fè ekonomi lajan, paske otrefwa m te konn ap achte pil pwodui, m’ achte òf pou pase sou po, ou achte sprey pou flite kay la, ou achte blakatòks se te ondepans ki te anplis. Men kounya kòb sa ,m ekonnomize l, m ka fè on lòt bagay itil avèk lòt bezwen.

ITV: Oke, ki enkonvenyan?

HM3: Lè ou pale de enkonvenyan, ou pale de emanatè a?

ITV: Wi.

HM3: M pa wè l gen okenn enkonvenyan paske mwen fè rechèch sou li, li pa pral nui sante, la pa pral banm pwoblèm, m wè gen frè m ki soufri asmatik aparèy la la, li dòmi avèk li okontrè, li konn ap goumen di m HM3 banm emanatè a pou m mete bò pye m, paske orefwa m apt ka pase blakatòks la lè li la, m pat ka tou flite tou lè li la, pakse otomatikman m pase blakatòks la maladi a pran l, mwen flite maladi a pran l.

ITV: Oke si n te ka fè on klasman ankò dezavantaj enpòtan, gwo avantaj ti dezavantaj kisa ou te ka pran?

HM3: Gwo avantaj enpòtan m t’ap pran.

ITV: HM3 si nou ta ka bay aparèy la on nòt ant zewo a dis ki nòt ou t’ap ba li?

HM3: M t’ap ba li nèf senkant (9,5).

ITV: Oke, poukisa ou retire demi pwen an?

HM3: demi pwen an paske li te sipoze aksesib avèk timoun pakse ou paka esklav on aparèy pandan ke ou gen timoun anndan on kay. Se sak fè m retire demi pwen an.

ITV: Aksesib kòman?

HM3: Aksesib kote ke m t’a reflechi si l te vin nan on bwat , kote ke bwat la te kabap twouye, on bwat kèlkonk, li kapab an fè, li ka an plastik, bwat la genda te twouye kote ke podui ke degaje a transfritrin nan ka pase ale san pou otan menm lè timoun nan manyen bwat la li patap deranje l, se sak fè m retire demi pwen an.

ITV: HM3 si ou te gen kèk rekòmandasyon ak moun ki ap dirije pwojè a kisa l t’ap ye?

HM3: Rekòmandasyon ak moun k’ap dirije pwojè yo, sensèman m fè pati de pwojè a, m pa gen anyen m ka repwoche. Kisa m te ka sijere? M sijere ke pwojè a pa kanpe , ke pwojè avanse paske pwojè a ki vini an, li sove on pakèt moun., li sove elèv lekòl kite dwe lekòl ki pa ka peye kounya , kolektè a ka vini li travay , li konnen li ka travay nan maten, li ka chache on kou li ale nan wikend, li ka al lekòl apremidi li ka fè on pakèt bagay. Sesi di otomatikman ke pwojè a kanpe mwen viv pwojè a, nan chak zòn pwojè a pase li pran nèf moun pou travay, e gen plis ke nèf moun nan chak zòn, apa de 8 kolektè plis on sipevizè nan chak blòk, ou gen oumenm, gen lòt moun yom tou ki travay na pwojè a se si di ke se pa sèlman kolektè avèk sipèvizè ki benefisye de pwojè a se tout moun ki benefisye ladan li, m panse ke si pwojè a ta avanse vè lavan li t’ap bon e anplis tou, pou pwojè a ta elaji nantout teritwa nasyonal la kote ke nan peyi m moustik ap manje moun yo ki se pòtapiman, moustik ap manje moun yo, e tan dòt kote ankò gen an il yo, moustik ap manje moun yo, m panse ke si pwojè a ta avanse li pa kanpe, l’ap itil lòt popilasyon menm jan li itil debisi, site kanade , li itil batis, li itil pedo an gwo se sa m t’ap sijere.

ITV:HM3 nou di ou on gran mèsi nou eskize nou difèt ke nou pran tan ou,ou te disponib pou nou, sa fè nou plezi e nou ba ou garanti ke sa ou di nou yo ap rete konfidansyèl pa gen moun ki ka di se HM3 ki di tèl bagay done a pral kode, mèsi nou pran tout sa ou di yo an konsiderasyon.

HM3: M remèsye ou tou, m remèsye tout responsab pwojè a. Mèsi

## Household 1 / member HM5 Female / Round 2

ITV: Ebyen nou pral kòmanse… koman ou rele ankò

HM5: HM5

ITV: HM5? Dakò oke. Nan ki nivo ou panse emanatè an ap pwotejew kont marengwen kap mòde’w lè’w nan kay la? Donk e …yo baw… yo poze’w repons yo epi wap koche fè yon ti wonn nan youn nan pòs ki pi ba yo oswa sèke nòt ki pi ba an a few wonn jan’w vle ann ale! vwala…Eske li pa pwoteje? Eske li pwotejew tou piti? Eske se byen? Eske se trèbyen? Eske li pwoteje’w nèt?

HM5: Toupiti

ITV: Ebyen koche

HM5: Ebyen li ta sanble se ta yon mo

ITV: Banm retire sa pou’w pa pran poul

HM5: fon’w ti wonn - ri

HM5: yo antoure chif la

ITV: Jan’w vle, yon ti kwa jan’w vle a

ITV: Vwala, nan ki nivo emanantè a pwoteje’w ak lot moun nan kay ou kont lot vèmin yo lè’w nan kay la? Nan ki nivo?

HM5: m’tande’w pale de lot vèmin komsi de lòt bèt?

ITV: Wi, vwala se sa wi, nan ki nivo poul pwoteje’w? Epi fè yon wonn nan youn nan repons ki pi ba yo oswa sèke nòt ki pi anba an ki pa gen repons. Nan ki nivo pou emanatè an pwoteje’w? Nan ki nivo?

HM5: Nan ki nivo mwen ta renmen’l pwoteje’m?

ITV: Wi se sa wi

HM5: m’ta renmen li byen pwoteje’m.

ITV: Ebyen se sa wi se senk (5) lan

HM5: poukisa ou pa di’l nan sa

ITV: Humm

HM5: Poukisa ou pa li’l nan sa?

ITV: Nan pa’l la, pou’w pa pran poul

HM5: Pou’m pa pran poul?

ITV: Ri… dakò! kesyon twa (3) nan ki nivo pou emanatè a pwoteje’w kont marengwen? Sa se pou’l ta pwoteje’w, pou’l pwoteje’w, e…travay ou obsève… nan ki nivo pou emanatè an pwoteje’w lèw deyò nan lakou a? Eske lè ou deyò nan lakou a ou santil pwoteje’w?

HM5: Kont moustik

ITV: Wi

HM5: Deyò an pa gen moustik non

ITV: Donk fè yon wonn nan youn nan repons ki pi ba yo oswa sèke nòt ki anba a siw pa gen repons menm.

HM5: pa gen repons

ITV: Ebyen wap koche la

HM5: map koche la?

ITV: Wi, dakò! Sa se la mwen ye kounya la

HM5: Nan kat (4) la?

ITV: Oke nan kat (4) la. Nan ki nivo pou emanatè an pwoteje’w ak fanmi’w kont lòt vèmin? e……. vèmin yo lè’w deyò kay la?

HM5: M’pa gen repons ankò si’m pa konnen li pwoteje’m… m’pa ka konn sa.

ITV: Dakò! Donk fè youn… fè yon ti wonn nan repons ki anba a

HM5: m’pa gen repons, m’pa konn pran’l soti deyò avèl

ITV: Dakò! Site ak dekri nenpòt lòt benefis ak dezavantaj emanatè an bay? Kounya e…an nap pale benefis , ki benefis li bay? ou mèt pale aprè sa nap ekri yo, ou mèt pale.

HM5: Benefis emanatè an bay?

ITV: Wi

HM5: Eske’m konn pran tan’m obsève sa? li ta kont moustik li ta sipoze repouse… repouse tou piti moustik yo

ITV: Ou santi sa?

HM5: Lontan yo te konn mòde’m plis men aprè sa mwen pa wè lòt benefis non.

ITV: Ebyen e kounya la ki dezavantaj? Wi

HM5: Yo pale’m de pwodwi chimik, HM6 pran’l li depoze’l sou tèt mwen lè’m ap dòmi.

ITV: Sou tèt ou anlè a?

HM5: wi , eske sa paka fè ‘m malad?

ITV: Non ban’m esplike’m koman ou pè?

HM5: Paske yo di li gen pwodwi chimik si’m ap dòmi prè yon bagay ki gen pwodwi chimik m’panse ke se yon sibstans li ka fè’m malad.

ITV: oke

HM5: Mwen fon’w ti pè’l paske…

ITV: Son’w dezavantaj? Eske’w santi li gen yon enpak sou sante’w piske ou pè? Ou santi li ka gen yon enpak sou sante’w?

HM5: Non a l’œil nu ou gen dwa pa wè li gen yon enpak sou sante’m oubyen petèt ke se a lontèm li ka fon’w enpak… pou kounya la m’pa konnen m’jis pè.

ITV: Li plis yon dezavantaj psikolojik.

HM5: wi

ITV: Plis psikolojik dakò! Wi HM6 di sa lè li mete’l yon kote ou toujou pè sa’w di a konfime sa HM6 te di a dakò! Sèl bagay m’ap di, epa sa’m vin fè la, pa gen ankenn efè segondè li gen sou sante moun ke emanatè an genyen, daprè sa yo di nan pwotokòl ankèt la.

HM5: O.. oke dakò mèsi

ITV: Ou ka ekri petèt sa’w fenk sòt di an rapid la

HM5: Ekri sa’m di yo?

ITV: wi ekri’l wi

HM5: oke

ITV: Map vin pou ou wi

HM7: li te okipe avek tiz

ITV: Dakò

HM5: N’a ekri’l aprè

ITV: An kreyòl wi ekri’l jan’w kapab

HM7: Ban’m ekri’l pou ou, li m’ap ekri’l pou ou

HM5: M’di konsa ke komsi nan benefis yo li fè yon ti elwanye moustik yo tou piti men kòm dezavantaj on bagay psikolojik ki nan tèt mwen e ke li ka fè’m malad.

HM7: Men ki dezavantaj ou avantaj?

HM5: Li elwanye moustik yo tou piti

HM7: M’pap mete toupiti map mete elwanye li elwanye

HM5: Nou gen tou piti nan a

HM7: E vre?

HM5: wi

HM7: <soupi> oke mete mwen panse ou mwen kwè

HM5: kwè lè’m fin fè konsta an

ITV: All right vwala! E byen n’ap vans e nou pa konnen nap vanse e byen…

HM5: m’ m’…

ITV: Klase enpotans avantaj ak dezavantaj emanatè an bay? Ki lòt benefis li bay?

HM7: M’panse gen lòt benefis li bay

ITV: Non e pa ou se ak li m’ap pale

HM5: Lòt benefis li bay?

ITV: wi … en..EN

HM5: M’pa wè lòt benefis li bay m’pa fè konsta sa m’pa remake sa

ITV: Dakò e ki lot dezavantaj

ITV: Ebyen ekri’l ekri’l la rapid, m’kwè ou te bay yon repons taleu an pavre? An tiz li menm li pi jeni pase nou li gentan bay repons yo e chak fwaye pa yo nou pa ka anpile yo.

HM7: An ok

ITV: M’ap vin kote ou taleu

HM7: a a a ……..

ITV: Ou genleu pat la leu yo te pase an

HM7: Wi o kontrè m’te gen randevou ak doktè Cyril li kanape vè m’t jwen ak doktè Chikòy nan fomasyon an nan 4^e^ blòk lè’m di’l m’te gen randevou avè’w li di’m non donk li sanble ke yo pat koòdone twòp.

ITV: wè oke

HM7: Kiyès? Ki bagay yo ouvè an? G pa la ebyen map fon’w bagay pou li

ITV: Oke trè byen nou nan kesyon uit (8) kounya la. Klase enpotans avantaj, dezavantaj pa youn nan repons ki anba yo oswa nenpòt nan bagay sa yo pa aplike oswa pa gen repons ki...Vwala! W’ap pran avantaj ou dezavantaj.

HM5: Kesyon an eske mwen pa konn vrèman fè jounen mwen nan kay la?

ITV: Oke ou ka di’l wi

HM5: Depi lè an mwen pa ekri Kreyòl non

HM7: Oke o m’dakò

ITV: Dakò mwen ekri , mwen wè’w ekri byen sanble yo lizib jan’w ekri an. Oke pou afè dezavantaj la ou ap chwazi repons a ki saw chwazi nan repons a yo pou avantaj ak dezavantaj?

HM5: M’di, pa vrèman

ITV: Ebyen eske pami avantaj w’ap chwazi sa yo pami dezavantaj w’ap chwazi eske gen ti avantaj? eske se dezavantaj fèb? Ki sa’w chwazi avèks sa en kelke sòt?

HM5: Wi men li enplike sa li konekte ak sa m’te di’w pa vrèman paske m’pa vrèman ret nan kay pou’m ka fè obsèvayon an.

ITV: Ebyen oke ou pa gen repons nou nan kesyon nèf(9) la site ak dekri nenpòt lòt benefis oswa dezavantaj emanatè an bay? Ki lòt benefis li bay? Ki lòt benefis ou wè’l bay? Talè an m’te di benefis ou wè’l bay.

HM5: Si pa gen benefis… pa gen lòt benefis

ITV: Non ou pa ka di li pa gen lòt benefis paske talè an ou di ke li chase kèk ti moustik, sa son’w benefis ou avantaj li ye

HM5: Wi m’konnen yo te di dekri a site benefis ……

ITV: A se nan yon lòt kesyon nou ye la

HM5: M’konnen

ITV: SIte ak dekri nenpot lot benefis sa vle di li pa oblije yon bagay , se pa di yon bagay map atann de ou, de bagay ou wè, ou ta wè, ou li ni mwen pa la pou’m korije’w pou’m di sa’w di an se pa sa. Ki lòt benefis ou wè’l bay? Ki lòt dezavantaj li bay? W’ap ekri’l pito?

HM5: m’ m’ m’ m’…

ITV: Oke lè’w ekri repons pou dezyè’m kesyon an, klase enpotans avantaj ak dezavantaj sa pa youn nan repons anba yo oswa nan nenpòt nan bagay sa yo wa bay avantaj ak dezavantaj w’ap klase nan sa yo

HM5: Gen gwo avantaj

ITV: E pou dezavantaj?

HM5: M’te di pat gen dezavantaj

ITV: Ebyen oke pat gen dezavantaj

HM5: Non tann mwen, sanble mwen pat fin ekri yon mo

ITV: Ebyen oke! Ebyen mèsi anpil nan patisipasyon ou nan travay la e nap bezwen lòt lan men’m leu li gen trèz (13) zan li ka pale tou epi pou nou fini fwaye sa.

HM5: Ti gason a poko men’m leve nan kaban li

ITV: Oke!

## Household 1 / member HM6 Male / Round 2

ITV: HM6 maten an se plezi pou nou ansanm, pou nou kontinye seri antretyen yo sou ilitizasyon aparèy emanatè a, pou nou evalye aparèy la. Nan ki nivo ou panse emanatè a pwoteje ou kont marengwen k’ap mòde ou lè ou nan kay la?

HM6: Nan ki nivo?

ITV: Wi.

HM6: M kapab di byen, paske lontan te konn gen anpil moustik, kounya nonb lan diminye anpil m k’ap di nan nivo byen.

ITV: Oke, nan ki nivo ou panse ou li pwoteje ou anndan kay la kont lòt vèmin yo, avèk lòt moun ki anndan kay la, nan ki nivo?

HM6: M panse li te sipoze elimine oubyen repouse tout moustik yo nèt, men toujou gen de twa men pa gen anpil menm jan avèk anvan lè pot ka genyen emanatè a.

ITV: Oke, HM6 nan ki nivo ou panse aparèy la pwoteje ou kont marengwen lè ou deyò nan lakou a?

HM6: Bon, anfèt nan lakou a, m pat janm konn wè marengwen non, se plis anndan marengwen yo konn rete.

ITV: Oke, nan ki nivo ou panse aparèy la pwoteje ak fanmi ou kont marengwen ak lòt vèmin yo lè ou deyò kay la?

HM6: Anfèt m pa konn si se paske pa gen pyebwa deyò a men, lè m deyò a m pa janm kòmsi wè marengwen.

ITV: Oke, èske ou ka site ak dekri pou nou kèk avantaj, enkonvenyan, emanatè a pote pou nou? Avantaj, dezavantaj?

HM6: Bon, kòm avantaj sèke te gen anpil moustik epi lè ou ap dòmi yo te gendwa ap chante nan zòrèy ou, mòde ou, men kounya pa gen sa ankò. Sa ka rive dèfwa ou jwenn on grenn ou de grenn men kòmsi li pa menm jan. Ou ka dòmi alèz, yo prèske pa mòde ou ankò.

ITV: Ki lòt avantaj ou ka site pou nou ankò, ki genyen?

HM6: Aprè sa m pa kwè gen lòt non.

ITV: Oke, si n te ka vini kounya nan dezavantaj? Dezavantaj emanatè a pote?

HM6: Petèt dezavantaj, emanatè a te ka on ti jan pi piti.

ITV: Li te kòmanse?

HM6: Li te ka on tijan pi piti.

ITV: Li pran plas, fò l minyatirize.

HM6: Li pran espas.

ITV: HM6, èske ou ka klase enpòtans avantaj ak dezavantaj, gwo avantaj? Ti dezavantaj? kisa ou wè ou te ka chwazi? Avantaj fèb? trè fèb?

HM6: Gwo avantaj.

ITV: HM6, èske ou ka site oubyen dekri pou nou kèk benefis aparèy sa pote pou nou?

HM6: Benefis? M ka di se menm bagay la, prèske kòmsi nonb de moustik lan li diminye, sa ka rive on lè konsa ou wè on grenn men pa menm jan a oparavan.

ITV: Ki dezavantaj?

HM6: Pa genyen.

ITV: Oke. HM6 si ou te ka bay aparèy la on nòt ant zewo a dis, ki nòt ou te ka ba li?

HM6: M t’ap ba li 8.

ITV: Poukisa ou t’ap ba li 8?

HM6: Uit paske m panse li te sipoze repouse tout moustik yo nèt, pou l te fè moustik pa antre anndan ditou, ditou, men sa ka rive on lè ou jwenn on grenn avèk de grenn, li pa menm jan avèk oparavan,

ITV: Oke, men HM6 avèk le tan, èske ou santi aparèy la toujou efikas toujou? twa mwa apre èske li bay menm randman.

HM6: Bon m ka di lè l te fèk vini nan kay la te toujou gen moustik men apre on mwa anviwon konsa m prèske pa wè moustik ankò.

ITV: Avèk le tan l’ap plis efikas selon oumenm?

HM6: Wi.

ITV: HM6 si ou te ka gen on rekòmandasyon pou moun k’ap dirije pwojè a kisa li t’ap ye? Si ou t’ap pwopoze yo, ba ya kèk konsèy kisa ou t’ap di yo?

HM6: Konsèy m t’ap ba yo se nan emanatè a sèlman, pou yo te fè l on ti jan pi piti pou nou te ka, paske li vrèman pran espas jan li ye a.

ITV: Oke , trè byen, HM6 se te on plezi pou nou te ansanm, nou kontan nou te fè ti pale sa, nou eskize nou tou paske nou pran tan ou,nou di ou on gran mèsi epi nou pran tout sa ou di an kont,nou ba ou garanti, sa ou di yo, y’ap rete konfidansyèl, pèsonn moun p’ap ka idantifye di se HM6 ki te di yo, bòn kontinuite avèk aparèy la, mèsi byen.

HM6: Oke, mèsi.

## Household 2 / member HM2 Male / Round 2

ITV: HM2 nan ki nivo ou panse emanatè a pwoteje ou kont marengwen k’ap mòde ou lè ou nan kay la? Nan ki nivo, si ou te ka fè on ti wonn nan repons ki pi ba yo. Si li pa pwoteje, tou piti ounyen byen, trè byen,pwoteje nèt,nan ki nivo?

HM2: Nan nivo emanatè a se trè byen.

ITV: Se trè byen?

HM2: Wi.

ITV: Poukisa ou ba l trè byen?

HM2: Paske o nivo kay la te tèlman gen anpil marengwen ladan l, nou pa t ka sipòte, paske fòk ou ta achte blakatòks pou ta limen lannwit pou timoun yo e nou tout. E de fwa lè l mòde ou , ou konn jwenn tikrè sou ou , akoz emanatè a ki vin la m pa nan menm frape men nou, menm on grenn ke n pa wè nan kay la ankò la.

ITV: Oke, èske ou panse li pwoteje ou kont tout moustik ki anndan kay la vrèman, ak tout fanmi ou.

HM2: Wi.

ITV: Nan tout kay la nèt? Nan tout sans?

HM2: Na tout kay la.

ITV: Èske ou panse li pwoteje ou kont moustik andeyò kay la?

HM2: Wi, dèfwa lè nou chita,nou pa sèlman rete anndan kay la sèlman, nou gendwa chita deyò a la, nou mete l kote nou. Menm si n ap bay blag li kote nou an, n’ap bay blag epi amanatè a, nou plase l nòmalman.

ITV: Donk na tout deyò a?

HM2: Wi, nan tout deyò a, li pwoteje nou, nou tèlman sèvi avè l, menmsi nou chita deyò a la, marengwen p’ap ka mòde nou.

ITV: Donk ou di li pwoteje ou kont moustik ki andeyò kay la.

HM2: Wi.

ITV: Ebyen nan ki nivo ou panse li pwoteje ou kont moustik sa yo deyò kay la ak fanmi ou?

HM2: Nan nivo li pwoteje lè ou gen pitit , timoun yo konn gen fyèv, ou ap mennen yo lopital tanzantan, men kounya la moustik la pa mòde yo, m konn pèdi konbyen tan depi lè bagay la kòmanse a pou jodya la apèn m kwè m te ale lopital ak youn ki te gen fyèv on fwa, depi lè a sa poko janm fèt.

ITV: Donk li pwoteje ou ak tout fanmi ou?

HM2: Wi.

ITV: HM2, èske ou ka site pou nou kèk avantaj , enkonvenyan aparèy sa bay. Si n te ka chita sou de twa avantaj kisa yo t’ap ye?

HM2: Avantaj m wè l genyen nou pa achte blakatòks gen on pakèt bagay pou ta fè, fò ou ta al lopital trè souvan, akoz yo menm ki vin la, yo pèmèt moustik la pa antre nan kay la, ou vin gen ekonomi ou ki plis,pake tout jounen si n t’ap depanse avèk pou moman kounya, ou pa nan depans sa. Sa se on lòt bagay li vin ye. Sa m ta renmen, tèlman m wè sa bon, pou lakay mwen m wè jan l bon, pou peyi m, peyi m gen anpil maladi ke m ta renmen yo ta fon jan tou pou yo ta voye l na peyi m tou paske lakay mwen tout moun yo malad anpil.

ITV: lakay ou se kibò?

HM2: Pòtapiman, paske si m wè jan l bon la pou mwen, m te ka mande o mwen si yo ta ka banm on chans bò lakay mwen tou.

ITV: Oke, avantaj yo gwo? Yo piti? Kòman ou ka evalye yo?

HM2: Gwo avantaj.

ITV: Oke, HM2, èske ou ka site kèk enkonvenyan aparèy sa bay? Ou ka ba nou de, twa?

Ou te ban ou avantaj, m panse fòk gen enkonvenyan tou, pou ou pat genyen?

HM2: Non, enkonvenyan ki pou te genyen, se moustik la ki ta pou rantre, li bloke l, li fè l pa antre, se sa ki vin byen pou nou, paske lè l pat la on premye fwa la, ou tap ka rete konsa la pou sde pa pye pou ou t’ap bat tanzantan. Men kounyeya la, sa k ta pou fèt la, li vin bloke l kounya. Paske jan kay la vin ye a kounyeya, kay la te pi piti, kay la vin ouvè, li vin pi gwo, la ou ta panse ke moustik la te ka rantre pi fasil, li vin pa jwenn pozisyon pou li rantre, paske a chak moman pou l ta rantre nan on pozisyon, ou plase emanatè yo on fason, lè n’ap dòmi tou, chak plase on fason.

ITV: HM2, èske ou ka klase avantaj aparèy sa bay? Avantaj:? Dezavantaj?

HM2: Avantaj? Non, m ka di li pa gen dezavantaj, li gen avantaj. Paske si ou t’ap depanse, avèk ou vin p’ap depanse, sa fè on lòt bagay, paske se on lòt ekonomi ou vin fè. Se sa ki vin pi bon pou nou.

ITV: Ou pa fokis sou enkonvenyan yo ditou, se sèlman avantaj yo?

HM2: Non, m jwenn avantaj ladan, m di sa m jwenn nan.

ITV: Ant zero a dis, ki nòt ou te ka bay?

HM2: Se pa dis m t’ap ba li sèlman, paske m ta renmen tout peyi a genyen l, nan nivo m wè l ye a.

ITV: Oke, avèk tan èske ou santi li bay menm randman, aprè de mwa, twa mwa, kat mwa randman toujou menm?

HM2: Li banm plis randman kounya, m santi m gen plis randman.

ITV: HM2 si ou ta gen on souwè pou aktivite a, pou pwojè a kisa l t’ap ye? rekòmandasyon?

HM2: Sa m ta renmen, fòk yo ta fè on fason pou yo ta mete l nan tout peyi a nèt, se sa k ta pi bon pou nou tout, pou sa m jwi a, pou yo te ka jwi li tou.

ITV: Ou te renmen ke…

HM2: Pou yo ta elaji l pou tout kote nèt.

ITV: Sou tout peyi a.

HM2: Pou tout moun ta jwen on pozisyon, pou l ta evite moustik mòde kèk moun se sa m ta renmen.

ITV: Ebyen ok HM2, n’ap pran sa an konsiderasyon, se te yon plezi pou nou te prezan la, nou di on gran mèsi, nou ba ou garanti ke sa ou di a konfidansyèl pèsonn p’ap ka di se HM2 ki di tèl bagay. Bon tavay, bòn kontinuite avèk aparèy la.

HM2: M di ou mèsi tou paske gras ak aparè la la, bagay la on tijan chanje pou nou tou pa bò isi, nou pa konn pou lòt kote.

ITV: Dakò HM2, mèsi anpil.

## Household 2 / member HM3 Female / Round 2 (Excluded)

ITV: Vwala sa pral mache byen vit la… vwala… 1 2 ok voila ça marche. Nan ki nivo ou panse emanatè a pwoteje’w kont marengwen kap mòdew lè’w nan kay la? Wa bay esplikasyon nan ki nivo li pwoteje’w, di sa’w obsève, sa’w wè.

HM3: Bon, daprè mwen, mwen wè emanatè a pote on gran amelyorasyon nan kay la, paske nou te gen anpil marengwen, men depi apre, depi lè yo fe nou kado emanatè a, konparativman avèk kantite ke’n te konn genyen, nou pa genyen’l ankò.

ITV: M’konprann, donk sa vle di…

HM3: M’panse emanatè a itil.

OD  Dakò, Ebyen wap fè u.. wap chwazi younn nan repons sa yo, nan ki nivo?, wap fè yon ti wonn nan repons ki pi ba yo, oswa sèke nòt anba, pou’w wè nan ki nivo li pwoteje’w. Eske se pwoteje’w tre byen, eske se pwoteje net, eske se byen, tou piti? eksetera, vlala chwazi … Dakò.

ITV: Kounya nou pwal nan kesyon 2 a. Nan ki nivo pou emanatè a pwoteje’w ak lòt mounn kay la , ak lòt mounn lakay ou kont lòt vèminn yo lè ou nan kay la? nan ki nivo pou’l ta pwoteje’w? e kòmsi se sa’w swete ,wap fè on ti wonn , vwala nan repons sa yo..Èske…

HM3: Pwoteje net

ITV: Dakò

ITV: Twa. Nan ki nivo emanatè a pwoteje’w kont marengwen lè’w deyò nan lakou a? Si pa gen repons wap di pa gen repons, si gen repons wap chwazi repons ki aproprie a. Lè’w nan lakou a, e nan ki nivo li pwoteje’w?, lè’w nan lakou a, èske ou wè’l pwoteje’w lè’w nan lakou a?

HM3: Wi paske a koz de solèy la , solèy la pèmèt transfluthrine nan fonksyone pi byen.

ITV: Trè byen hein.

ITV: Kat. Nan ki nivo emanatè a pwoteje’w ou Jilia* ak fanmi’w ou kont lòt vèminn yo lè ou deyò kay la? Fè yon ti wonn nan youn repons anba… ki pi ba yo.

HM3: Trè byen.

ITV: Ebyen dakò. Poutan nan lòt fwaye yo, yo di li pa pwoteje yo lè yo deyò a, donk sa di yo pa gen menm nivo konpreansyon. Dakò.

HM3: Paske petèt ke yo pa itilize’l deyò, yo ka…, gen mounn ki gen dwa pran emanatè a li jis kite’l, paske gen on jan pou itilize’l, map fè on sèvis la, msispèk la ka gen marengwen m’plase emanatè’m tou prè’m, epi map fonksyone, la moustik paka vinn bò kote’m, si mounn nan jis pran emanatè a li plase’l on kote konsa fiks, li paka konnen si li itil ak si’l pa itil. Paske se nan espas mwen ye a pou’m itilize’l.

ITV: Dakò. Men èske yo, yo pat ba yo enfò…yon fòmasyon sou kòman pou yo itilize’l?

HM3: Wi yo ba yo’l , yo ba yo on fòmasyon , e menm fòmasyon m’pran avèk yo.

ITV: Men yo pa fè’l konsa yo menm.

HM3: Men sè ke yo gengwa…

ITV: Pa fè’l menmjan avè’w.

HM3: … Pa prete atansyon, yo pa fè’l jan pou yo fè’l, yo pa itilize’l a bon esyan.

ITV: Ou menm ou deplase emanatè a kote’w wè’w bezwen’l.

HM3: Wi map sèvi sou galri a m’pran emanatè a m’mete’l bò kote’m sou galri a, lè sa m’gen on kouvèti moustik la paka vinn pike’m.

ITV: Dakò, dakò. Sa se on repons ki enteresan, paske mpa jwenn repons sa kay lòt mounn yo, se on repons ki fyab, ki on trè bòn repons.

ITV: Ok Site ak dekri nenpòt lòt benefis oswa dezavantaj ke emanatè a bay, Ki lot benefis li bay?

HM3: Benefis emanatè a ba ou, li kapab anpe…, li pèmèt ke mouskik ki fè… ki bay zika a paka mòde’w paske li repouse moustik la, sa se on bèl avantaj paske lè ke’w malad ou gen on depans pouw fè, ou gen on tan ou pèdi pou al kay doktè, otomatikman ke’w pa pike kounya ou pa gen ankenn sousi pou’w di ke ou ka fè zika, m’gendwa di ke’m paka fè zika a pati de emanatè a, paske’m m’konnen emanatè a li men’m li repouse moustik la.

ITV: Dakò

HM3: Sa se avantaj la, mpa vrèman wè dezavantaj.

ITV: M’konpwann.

HM3: M’pa wè, O kontrè li plis itil, paske otrefwa, se te aparèy ki konekte nan kouran an ke nou pa menm konnen, ke nou pa gen on etid sou li, se li nou konn itilize. Ou konekte’l ou chaje’l epi wap tuye marengwen avè’l, ou pa konn si li gen efè segondè, ou pa… men avèk emanatè a, pwiske nou gen ide sou emanatè a, epi nou konn kijan, e ki etid ki fèt sou li, la li plis klè ke’m kapab di ke emanatè a valab konparativman avèk lòt yo.

ITV: Lòt pwodwi yo konn itilize yo pou…

HM3: Lòt pwodwi ke yo konn itilize yo, tankou blakatòks la, m’gen tifrè’m nan ki fè asmatik, m’gen pitit gason’m nan tou, li paka respire byen, ki paka pran lafimen, sa li menm li pa gen lafimen ke’l bay. M’panse ke m’pa wè efè segondè ladan’l.

ITV: Blakatòks la bay lafimen vre, li ka fè’w mal pou respire si’w asmatik.

HM3: Nou pa ka itilize blakatòks la.

ITV: Li plis gen avantaj ke dezavantaj.

HM3: Wi li gen plis avantaj ke dezavantaj paske si’m itilize kant… ki kantite lajan’m pwa’l genyen pou’m achte blakatòks? kay la pa piti, pou’m ta di map mete blakatòks nan kat kwen, ckak pyès yo, li ta pwa’l koute’m on pakèt lajan, non sèlman emanatè a mwen jwenn li gratis e pi m’pa gen depans pou’m fè.

ITV: Donk ekri kèk bagay sa yo la rapid la, sitou kòm ou gentan bay avantaj yo, plis ekri… di vwala …vwala...

HM3: M’pral met avantaj, quoi ?

ITV: Ou ekri vit, ou te ka fè on bon sekretè.

HM3: (ri)

ITV: On gwo kote.

HM3: Mwen ekri wi… Map li pou ou: mwen panse ke emanatè a bay on pakèt avantaj, kòm pwoteje mounn kap viv nan kay la kont moustik ki bay zika ak yon paket lòt maladi ke mounn pran nan moustik. Mwen pa vrèmean ka di gen dezavantaj paske mwen pa peye anyen pou emanatè a, lòt pwodwi yo koute pri syèl. Ou pat gen espas ankò, m’tap fenk kare pale.

ITV: Wè, sof si ou vle di yo la, nou tap tou anrejistre si’w vle pale, bon ou gentan di yo déjà m’panse..

HM3: Li gentan koupe.

ITV: Bon ou te ka ekri dèyè.

HM3: Ok

ITV: Ou wè map chache pou ou.

HM3: Ou wè sa map di yo enteresan tou.

ITV: Wi sa’w di yo enteresan vre …Ok li’l pou nou rapidman pou nou ka kontinye.

HM3: Paske avan te chikoungounya ki te ravaje peyi a, epi presizeman blòk 1, blòk 1 an, mwen se on viktim de sa, mwen sigjere leta ayisyen an pran pwojè TEAZ la an chaj, paske sa ka ede kominote tankou , laplèn, gonayv, ilavach ak anpil kote ki bay marengwen pa pakèt, epi ki gen anpil jit ki pwodwi marengwen, epi fè fomasyon pou ayisyen pou yo konnen kòman pou yo detwi jit yo, elimine tout fut , droum a syèl ouvè, e latrye.

ITV: Enteresan, ou tou fè… ou tou fòme kèlke eleman rekòmandasyon nan…pou pwojè a. déjà se sa.

ITV: Ebyen nap kontinye a kesyon yo, mwen kwè nou prèske fini, vwala nou nan kesyon 6 kounya. Klase enpòtans avantaj / dezavantaj sa a pa youn ki nan repons ki anba yo, oswa nenpòt nan bagay sa yo, e ke si pa gen repons wap pran pa gen repons nan avantaj wap chwazi.

HM3: Ok avantaj.

ITV: Si’w wè pa gen avan. dezavataj tou wap di pa genyen.

HM3: klase enpòtans avantaj / dezavantaj sa a pa youn nan repons ki anba yo, oswa nenpòt nan…

ITV: Saw sot di yo la, Èske Si gen gwo avantaj wap chwazi si gen gwo avantaj

HM3: Nan pwojè a?

ITV: Pou bagay emanatè a.

HM3: Wi gen gwo avantaj.

ITV: Ebyen vwala, e yo ou sot di la nòmalman.

HM3: Wi avantaj ekonomik.

ITV: E pa sèlman… ak sanitè tou paske li…e dezavantaj èske gen ti dezavantaj?

HM3: Gwo dezavantaj, pa bon menm…

ITV:…Ti dezavataj, pa twò mal, dezavantaj fèb.

HM3: Map mete dezavantaj fèb.

ITV: M’panse yo ta dwe mete yon kategori pa gen dezavantaj jan bagay sa ye.

HM3: Bon m’gen dwa pa chwazi.

ITV: Wi.

HM3: Pa chwazi vle di ke’w pa satisfè de…

ITV: De sa yo mete a ou byen ou gen dwa ekri pa gen dezavantaj, ou ka ekri’l wi, e sak fè m’pa … kesyonè sa se on bagay tèt chaje paske déjà ou di ke’w pa wè dezavataj, yo pa ka mete pou’w chwazi on bagay ke’w pa wè. Dakò.

Sèt: Site ak dekri nenpòt lòt benefis oswa dezavantaj ke emanatè a bay, a pati de sa’w sot di yo la, ki lòt benefis li bay?

HM3: Lòt benefis li bay?

ITV: Men ou te pale de bagay ekonomik sa’w te vle di pa la?

HM3: Ekonimik paske si map itilize blakatòks, map achte, on pakèt blakatoks pou’m achte.

ITV: Interesan hein, sa fe on lòt bèl avantaj.

HM3: Sa fè anpil blakatòks, si se bakatòks… lòt aparèy la vann trè chè, ou gen ensektisid a kouran an trè chè.

ITV: ouais*

HM3: Ou pa jwenn li ka malere, kote’w pwal jwenn li la?

ITV: Epi gen on lòt aparèy itilize tankou rakèt.

ITV: wi ou griye bagay la.

HM3: Sa gen dezavantaj ladan’l, ouka kite’l , on timounn ki dezòd ka pran kouran ladan’l

ITV: Li boule’l … vwala

HM3: Li ka tuye on timounn paske timounn nan ka pa konnen li konekte’l, epi li al met men’l ladan’l.

ITV: Se vre.

HM3: Ok map reli ankò pou’m kapab ekri’l: site ak dekri nenpòt lòt benefis oswa dezavantaj…

ITV: Ou sot di lòt benefis yo, kilè men … kò’m ou di pa gen dezavantaj, ekri jis lòt benefis yo.

HM3: Oke, lòt benefis ke emanatè a bay … sèke… mwen pa gen pou’m kouri lopital. Arbovirose* se sa ? le mot? abrovirose ? maladi moustik bay la, abrovirose?

ITV: Ekril e m’pa sonjel non, m’pa sonje.

HM3: Wi se abwoviroz…. non mpa… mle fikse…Abwoviroz se on maladi…

ITV: Filarioz ? ou si e pa li ?

HM3: Non kòm si tout… ou pran deng, ou pran filarioz, (pran katab mwen an pou mwen Gabè), ou pran deng, ou pran filarioz, ou pran zika, ou pran chikoungounya, yo tout sa ansanm

ITV: Gen on nom, gen on nom ki dezinye tout pwoblèm sa yo.

HM3: (Pran valiz la pou mwen).Wi *arbovirose li ye, eskize wi,on moman, on moman.

ITV: Bon ou mèt kontinye, apre sa wa ekri?.

HM3: mèsi, on moman.

ITV: ok.

HM3: M’fè anpil rechèch ou konprann sa?

Obraillant Damus: Trè byen trè byen?.

HM3: Oh m’pa ret sou TEAZ non, m’gen on bè’l dokiman la , ok…map ba’w mo a kounya wi ITV: Dakò.

ITV: Ou pa jwenn li?

HM3: *Maladie à transmission vectorielle, men abrovirose , se on maladi li ye , talè…

Obrilant Damus: Ou pa wè’l, ann kontinye, apresa wa chache’l.

HM3: Ok ou kapab di maladi vektoryèl tou, maladi vektoryèl.

ITV: Dakò epi

HM3: M’poko fini non.*Abrovirose se sa wi, sam di a.

ITV: Wi nap ka verifye sa aprè. Se on tèm teknik li ye ou pa finn si si ou byen entegre’l.

HM3: Fini wi

ITV: ok.

HM3: Li pou ou?.

ITV: Wi ou mèt li’l wi, se tap enteresan.

HM3: Lòt benefis ke emanatè a bay sè ke mwen pa gen pou’m kouri lopital pou ka maladi vektoryèl yo, epi mwen vinn konnen kijan pou mwen elimine marengwen.

ITV: Ok trè byen, kounye la nou nan kesyon 8 la. Klase enpòtans avantaj / dezavantaj sa a, sa a, pa youn nan repons ki anba yo, oswa nenpòt nan bagay sa yo… bon euh m’panse se youn nan avanvantaj sa wap pran. E si’w pa jwenn ankenn avantaj wap wè.

HM3: Dezavantaj Fèb, avantaj fèb, ti avantaj, gwo avantaj.

ITV: Kounya nou nan 9 la. Site ak dekri nenpòt lòt benefis oswa dezavantaj, lòt benefis, m’panse ou di yo déjà nòmalman si’w gen lòt, kò’m ou plen ide, si’ gen lòt ide ou ka bay

HM3: Oui m’genyen.

ITV: Ou ka bay lòt benefis ou wè sa bay. Apre sa wa li, wa vèbalize repons la pou nou.

ITV: Ok vwala, vèbalize’l pou nou.

HM3: Lòt benefis ke emanatè a bay sè ke mwen konnen nan ka ke mwen gen on basen dlo ki pwodwi lav, mwen gen pou’m pwòpte’l toutan, epi si mwen vle chita pre espas la, mwen gen pou dote’m de emanatè a.

ITV: Trè byen, ebyen nan dezyèm kesyon an, nou pral fini klase avantaj dezavantaj sa a pa younn nan repons ki anba a …Vwala donk ou déjà gen repons ou déjà, ebyen mèsi anpil HM3, ou ba nou de repons trè ekselan, de bon repons.

HM3: Trè byen.

ITV: Paske ou konprann aparèy la, ou itilize’l vrèman, ou wè valè’l, kounya m’pral fèmen, pou mwen bay mari’w pale paske nan fwaye a se twa mounn pa fwaye fòk li pale tou.

## Household 2 / member HM8 Male / Round 2

ITV: HM8, se on plezi pou nou ansanm jodya pou n fè on ti koze sou aparèy ke ou ap itilize depi kèk tan, emanatè a.sa ap pèmèt nou evalye aparèy la. Nan ki nivo ou panse aparèy la pwoteje ou kont marengwen k’ap mòde ou anndan kay la?

HM8: Pou m kòmanse, mwen salye nou, nou menm ki la, mwen salye staf la ki ansanm avè m nan komite a pandan ke n’ap fè on travay, pou mwen , aparèy la enpòtan anpil nan ki sans li enpòtan sèke gen on pakèt bagay ou pat konnen ou vin fini pa konnen l paske tank ou ap aprann sa son enpòtans, dezyèm bagay sèke marengwen li anmède ou nan tout sans, ou ka chita ou ap fè on bagay, li mòde ou ou pa ka dòmi, on fwa aparèy la vin vini, li fè ke pa gen marengwen ou dòmi alèz, Dèfwa ou ap dòmi, marengwen an konn nui nan zòrèy ou ou vin pa ka dòmi, ou ap pèdi san, li pa bon pou ou donk se on gwo avantaj li ye,nan sans sa m ka di ke emanatè a li bon anpil.

ITV: Alò li pwoteje tou piti? Byen pwoteje? Trè byen pwoteje? Pwoteje nèt? Kisa ou te kapab chwazi?

HM8: Pwoteje nèt, poukisa paske nan kote ou ye a depi ou mete l, ou pa jwenn marengwen ankò. Si ou pa jwenn marengwen li pwoteje nèt. Ou gen de twa espas, ou mete l na de twa espas ou pa jwenn ankò, yo ale paske ki wòl li, wòl li se fè moustik yo yo fui kote a.Donk li jwen wòl li, sa pou l fè a li fè l depi ou mete l y’ale donk li bon, li pwoteje nèt.

ITV: Èske li itil ou avèk moun ki anndan kay la, ak fanmi ou?

HM8: Li pwoteje m paske se mwen avèk manman m k’ap viv anndan kay la, gen de bagay on seri de moun afronte, m pa afronte l, paske gen moun ki gen timoun, mwen m pa gen timoun, on fwa m mete l on kote li p’ap deranje m, paske manman m konn son bagay li ye, li p’ap al ladan l, nan on sans m ka di li pwoteje m nèt mwen menm. Paske bagay moun yo ap viv paske yo gen timoun, timoun nan ka al manyen l sa se bò ap yo, bò kote pa m an mwen menm li regle tout afè m paske se mwen a on granmoun ki anndan pa gen bagay timoun ap vin manyen’peu importe’ kote m mete l, mwen ak granmoun nan manman m nou konn kisa pou n fè. Donk li pwoteje m nèt.

ITV: Lè ou deyò kay la li pwoteje ou?

HM8: Non, paske sa k fè m pa ka di lè m deyò a li pwoteje m, kay la se on kay li fèmen li konn ret anndan, menmsi ou ta rete deyò, ‘une fois’ ke ou pran l ou sòti avè l l’ap pwoteje ou paske lè m anndan , paske bò lakay mwen gen pyebwa , pyebwa lè l frèt li rale marengwen donk li papwoteje ou jan pou l pwoteje ou, men pou l pwoteje ou fò ou sòti avè l’une fois’ ou pa sòti avè l, m’ p ‘ap al di ke li ap pwoteje m, èske ou wè sa m di an paske gen van, van ap pouse merengwen gen on pakèt bagay ki fè marengwen ki vin jwenn ou, lè m deyò pou l pwoteje m fò m sòti avè l.

ITV: HM8, èske ou ka site oubyen dekri pou nou kèk avantaj, dezavantaj aparèy la pote emanatè a bay?

HM8: Pou mwen emanatè a gen on pakèt avantaj, li gen avantak, ki avantaj yo ye, sèke nan pa gen marengwen ankò nou ka dòmi pi alèz, se sa yo. Lè m chita emanatè a la, m’alèz, m pa pè marengwen, m pa pè marengwen ki pote maladi yo donk m p’ap ka malad tout sa yo se avantaj, Ki dezavantaj, m pa ka di dezavantaj, lepoukwa, paske tout oparavan pat gen emanatè m te gen on pakèt bagay ki te konn ap rive m donk si li vin la li fè yo pa rive m sa se on avantaj li ye, m pa ka di dezavanyaj ladan l.

ITV: Oke, bon si n ta klase enpòtans avantaj sa yo , kòman n te ka fè l. Gwo avantaj? ti avantaj? avantaj fèb? Dezavantaj? kisa ou te ka chwazi?

HM8: M t’ap klase l gwo avantaj.

ITV: Ki benefis

HM8: Benefis lan se ‘une fois’ vi ou on koye marengwen ap mòde ou, ou ap toujou bezwen bagay pou ou achte, pwodui pou ou flite sesi pou ou ka alèz, donk ou pa depanse lajan epui li ka ede ou pou sante ou.

ITV: Ki enkonvenyan?

HM8: Bon ki enkonvenyan, enkonvenyan li ka on sèl bagay se sèlman si emanatè a ta gen on efè segondè, m pa konnen , si m pat gen anyen , m pa ka di li gen enkonvenyan. On sèl enkonvenyan se si li ta gen pou l ta bay on maladi, men si l p’ap bay on maladi, pa gen enkonvenyan paske li plis ede nou ke l banm pwoblèm.

ITV: Ki lòt avantaj ou jwenn an itilizasyon aparèy la? Ou ka dekri lòt avantaj?

HM8: Ki avantaj? Avantaj jwenn ladan se lajan ou pa depanse , se sante ou, se on seri de maladi k’ap vini depi se moustik ki bay yo, ou ka di gras a emanatè a ou p’ap genyen yo. Donk sa yo se avantaj yo ye, ou pa depanse lajan achte pwodui paske emanatè a ‘une fois ‘li trete l’ap repouse moustik yo epi ou ka alèz se ki avantaj emanatè a pote pou mwen.

ITV: Ki enkonvenyan?

HM8: M pa gen enkonvenyan, m pa ka di li bay enkonvenyan, enkonvenyan m te di l talè a se si e sèlman si li t’ap gen on efè segondè, depi li pa gen efè segondè donk pa gen enkonvenyan.

ITV: HM8 si ou t’ap bay aparèy la on nòt ant zewo a dis ki nòt ou t’ap ba li?

HM8: Pou mwen m t’ap ba l dis.

ITV: Oke poukisa ou t’ap ba l dis?

HM8: M’ap ba li dis paske kote m ye a pa gen timoun, sak te fè m ba l zewo se si m te gen moun ki pou te gen pwoblèm avè l.On fwa li la, li fè tout travay li te gen pou l fè, se pouse marengwen li pouse l e jan li ye ou ka soti avè l. Epi kote ou ye a la, li fè tout travay pou li fè pa gen moun ki ka blese ladan l, konsènan lakay mwen paske se mwen a manman k’ap viv. Donk m pa gen moun ki pral frape ladan, ki pral gen on pwoblèm ladan l. Donk li fè sa li gen pou l fè a , li pot solisyon pou l pote a pou mwen se dis li ye.

ITV: Oke avèk tan kòman ou wè efikasite a? Li trè efikas? pi efikas? Pa efikas ditou avèk tan de mwa aprè, twa mwa aprè?

HM8: M p’ap janm ka di sa, depi ou met pwodui a ladan l li fò pandan li fò a l’ap fè on travay, tout outan li ap pran tan pwodui ap soti m p’ap al di li efikas menm kan, men li toujou efikas. Èske ou wè sa m di ou la? Lè ou fèk mete l , lè ou depoze ak sant bagay la k’ap monte, sant ki gen ladan l nan lè sa li frape tout bèt yo, bèt yo ale rapid ,men sa pa vle di nan de twa , mwa bèt yo pa toujou ale non, men li pa gen menm pwogresyon an, yo pa kouri menm jan paske pwodui a lè l fèk genyen li pi fò men lè l kòmanse desann li toujou gen menm sal fè a , li ka pa fè l nan menm tan men li fè l kanmenm.

ITV: HM8 si ou te gen kèk rekòmandasyon ak moun ki ap dirije pwojè a kisa l t’ap ye?

HM8: Kisa l ta ye,sèke pwojè sa se on pwojè ki aprann mwen on pakèt bagay.

ITV: Kisa l ye?

HM8: Li aprann mwen on pakèt bagay m pat konnen sou marengwen, gen on pakèt moun nan sit la ki ap konfronte ak menm bagay avèk nou,lakay yo te gen marengwen, mwen lakay mwen te chaje marengwen lè m mete l m pa jwenn ankò. Donk li pouse yo, m ta swete yo toujou ouvri l, yo,kenbe l sa vle di yo agrandi l pou jis pran sit la oubyen nasyonal, tout andeyò m pa bezwen konnen jan y’ap pran l paske jan li ye pou mwen an, li ka menm jan pou lòt yo tou, paske moun yo nan nesesite a epitou li ede nou on fason ekonomik, m wè mwen menm li ede m peye lekòl. Paske sa enpòtan pou mwen paske m’al lekòl.

ITV: Kòman li ede ou peye lekol?

HM8: M lekòl nan inivèsite paske lè ou ap chwadkou, ou konnen se pa sesyon lè kòb sa vin nan men ou, ou ka peye on sesyon dèfwa lè paran ou pa ka bò kòb la jan pou l ba ou l lan, on fwa ou gentan gen tikòb sa ou pran ou peye sesyon, mwen menm li ka ede m peye lekòl invèsite, gen moun li ka peye lekòl klasik,li bay manje ’une fois’ yo agrandi l tout otan gen plis fanmi ladan l moun yo ka jwen kote pou yo viv menm jan mwen menm m’ap viv ladan l.

ITV: Oke, trè byen HM8 se te on plezi pou n te ansanm , sa fè nou plezi e nou ba ou garanti ke sa ou di nou yo ap rete konfidansyèl pa gen moun ki ka di se HM8 ki di tèl bagay done a pral kode, mèsi nou pran tout sa ou di yo an konsiderasyon. Mèsi.

HM8: Se nou pou m di mèsi paske se nou avèk TEAZ ki vini ak pwojè a ki fè m ka benefisye sa m’ap benefisye an la, bon mwen menm m ta swete TEAZ agrandi pou l ka ede lòt moun jan yo ap ede m lan tou, mèsi ak moun ki fini.

## Household 2 / member HM12 Male / Round 2

ITV: HM12 nan ki nivo ou panse aparèy la pwoteje ou kont marengwen k’ap mòde ou anndan kay la?

HM12: Bon nòmalman,kay la se on kay ki an konstriksyon e gen plizyè bagay ki defèt na kay la. Te gen on ti basen ki te fèt nan koub lan la, nòmalman yo kraze l men li konn konsève dlo, te gen anpil moustik, ou konprann sa m vle di ou, kay la te plen moustik, yo anpeche ou dòmi tou sa men depi lè pwojè «  TEAZ » la ateri, bagay yo vin on jan chanje, yo vin ban ou emanatè pou nou konbat kont moustik yo, nòmalman bagay yo vin plizoumwen, gen mwens moustik. Bon, akoz de emanatè a yo vin bay la gen mwens moustik nou vin dòmi pi byen tou paske nòmalman moustik yo konn ap rele nan zòrèy ou, dèfwa chalè a egzajere ou oblije kouvri tout tèt ou. E bèt yo nwizib tou. Akoz emanatè a , bagay yo vin on jan chanje.

ITV: Li pwoteje tou piti? Byen pwoyete? Pwoteje nèt?

HM12: Li byen proteje.

ITV: Nan ki nivo ou panse aparèy la pwoteje ou ak fanmi ou kont moustik ki anndan kay la? Nan ki nivo?

HM12: Nòmalman, moustik yo lè yo mòde ou, li pwodui plizyè maladi puiske emanatè a vin la kounya, vin gen mwens moustik, byenke emanatè a li detui moustik yo vrèman men ap toujou gen ti grenn k’ap vini, men si ke yo vin mwens kounya ap gen mwens moustik k’ap mòde ou puiske vin gen mwens moustik pou kounya, ou vin on jan pwoteje.

ITV: Men nan ki nivo, ou panse emanatè a pwoteje ou kont moustik lè ou deyò kay la? Se pa anndan kay la non,deyò kay la avèk fanmi ou ,èske li pwoteje ou kont moustik?

HM12: Lè n deyò kay la, pa tèlman gen pwoteksyon, paske ou nan la ri ou deyò, si on moustik vini li ka mòde ou a nenpòt moman men si ou anndan kay la nòmalman se on lòt detay.

ITV: Efikasite a pa menm lè lanndan ak lè l deyò?

HM12: Non, anndan kay la ou gen emanatè ou pi byen pwoteje.

ITV: HM12, èske ou ka site pou nou oubyen dekri kèk avantaj, enkonvenyan aparèy la genyen?

HM12: Premye avantaj la , sèke oparavan lè pot ko gen amanatè a m te konn ap fè pafim, m konn pran po chadèk pou n fè pafim, pou n ka elimine marengwen e dè fwa tou nou achte blakatòks kounya bagay yo vin on jan chanje, yo pa tèlman itilize bagay sa yo ankò se youn nan avantaj men dezavantaj lan sèke marengwen akoz emanatè yo, yo vin pa gen avantaj pou yo antre fè sa yo vle ankò moun vin dòmi alèz, yo pa fè bri nan zòrèy nou ankò ou ka dòmi do touni tou sa.

ITV: Ou dòmi alèz? Ou pa achte blakatòks?

HM12: Wi, nou pa fè pafim ankò.

ITV: E si n ta ka pale de enkonvenyan, ki enkonvenyan? Ki dezavantaj aparèy la pote pou nou?

HM12: M sot di ou sa, dezavantaj lan, kòmsi marengwen an vin genyen kounya koz emanatè a, li vin pa gen aksè ankò pou le fè sa li vle, pou l mòde moun.

ITV: M vle anfèt pale de oumenm, kisa ou wè kòm enkonvenyan  ke aparèy la pote, dezavantaj?

HM12: M pa kwè l pote.

ITV: Si ou wè li pa genyen, ba li sèkle pa gen repons.

ITV: Oke, si ou te klase avantaj yo, èske li bay gwo avantaj? Ti avantaj?

HM12: Pou byen di ou, li bay gwo avantaj paske akoz kounya nou vin remanye bagay yo, epi akoz emanatè a tou, epi nou vin on jan pi anpè. Gwo avantaj.

ITV: Oke,men si ou te ka site kèk avantaj ankò aparèy la bay.

HM12: Avantaj li bay,sèke nou dòmi byen, lè ou kouche, li pa fòseman pou kouvri ankò, paske moustik yo konn ap rele nan zòrèy ou. Dèfwa ou konn domine. Ou konprann, ou dòmi byen, ou gendwa ap gade on ti televizyon la, dòmi pran ou san ou pa kouvri, ou dòmi alèz.

IVT: Si ou t’ap bay nòt ant en a dis ki nòt ou t’ap bay aparèy la?

HM12: Pou byen di ou, m t’ap bay nèf sou dis, paske lòt grenn nan, se paske nou mèt fè sa nou vle nòmalman ap toujou genyen lòt ti marengwen k’ap sikile, ou pa konprann sa m vle di ou? Nòmalman se nèf sou dis m t’ap bay. Ou konprann, paske se vre aparèy la egzizte, aparèy lan la, men li p’ap detui tout marengwen k’ap rive sikile a travè zòn an oubyen nan kay lan, gen lòt k’ap pwodui toujou, m t’ap bay nèf sou dis.

ITV: Dakò, avèk tan ou toujou santi li toujou efikas ? Aprè on mwa de mwa, ou santi li bay menm randman? Kòman ou wè sa?

HM12: M twouve bon randman paske dèfwa m chita la, ou patap k’ap chita, ou t’ap jwenn marengwwen k’ap flote nan pye ou. Li bay bon randman.

ITV: Menm aprè de , twa, kat mwa li toujou bay menm randman?

HM12: Menm randman.

ITV: Oke HM12 si ou ta gen on rekòmandasyon, on souwè pou pwojè a kisa li t’ap ye?

HM12: Nòmalman si m ta gen on souwè kisa l t’ap ye? N’ap di tout staf TEAZ lan mèsi pou opòtinite sa, sou travay y’ap fè a sitou , m garanti nou travay la se vre li bon ,li bèl e si m byen raple m lè pwojè sa te fèk vin na zòn nan , premye 8 jou travay nou te fè an kòmsi a travè nou yonte konn ap rele marengwen, marengwen. Yo pat janm konnen ki avantaj TEAZ t’ap chèche, ki maladi li t’ap konbat, kont moustik to tou sa , m di nou mèsi poun pwojè a, mèsi pou opòtinite an, mesi paske yo te panse avèk blòk lan, pa sèlman blòk lan tout lòt blòk ki egziste deja yo, m si mèsi pou sa, se on plezi pou mwen pou m te fè pati gwoup la.

ITV: Oke mèsi HM12 se te on plezi pou n te ansanm, mèsi nou ba ou garanti ke sa ou de nou yo le rete konfidansyèl, pèsonn moun p’ap ka wè se HM12 ki te di nou tèl bagay.

HM12:Oke, mèsi.

## Household 3 / member HM9 Female / Round 2

ITV: HM9 maten an se plezi pou nou ansanm, nan kad seri antretyen yo sou ilitizasyon aparèy emanatè a, pou nou fon ti pale de aparèy la avèk ou konsa sa pèmèt nou evalye aparèy la. Nan ki nivo ou panse emanatè a pwoteje ou kont marengwen k’ap mòde ou lè ou nan kay la?

HM9: Se on plezi pou mwen, pou m patisipe nan pwojè sa, emanatè a se on aparèy ki repouse moustik, li trè efikas e otrefwa nou te konn itilize blakatòks, itilize begond tout lòt bagay k’ap, kounya avèk emanatè a nou repouse moustik san pwoblèm.

ITV: nan ki nivo ou panse ou li pwoteje ou anndan kay la kont moustik, kont lòt vèmin yo, avèk lòt moun ki anndan kay la, nan ki nivo avèk fanmi ou?

HM9: Trè gran, li repouse l anpil.

ITV: Nan ki nivo ou panse ou li pwoteje ou kont moustik andeyò kay la?

HM9: Ou konnen moustik yo se nan espas anndan yo toujou ye, men kounya pa tèlman gen moustik ankò, jan te konn genyen an prèske pa genyen moustik ankò.

ITV: Deyò a tou li pwoteje ou? Nan lakou a? Deyò a?

HM9: Bon, m pa tèlman wè anpil moustik ankò, otrefwa m te konn wè anpil moustik, m pa wè anpil moustik ankò.

ITV: Oke, èske ou ka site ak dekri pou nou kèk avantaj, enkonvenyan emanatè a pote pou nou?

HM9: Avantaj emanatè a pote pou nou, sèke, nou te konn itilize blakatòks, achte blakatòks ou konprann, nou pa itilize sa ankò,emanatè a fè travay la pou nou, dezavantaj nou genyen m’ap redi l ankò, emanatè a okipe twòp espas , fò yo fè on fason pou emanatè a okipe mwens espas , ou konprann ke l te ka akwoche on kote, nan on mi kòmsi kounya alèd de timoun yo,ou konnen gen moun ki gen timoun , timoun yo ou konnen yo dezòd , pou y opa touche l, paske m konnen li gen pwodui timoun pa dwe touche l pou l mete nan bouch li, sèlman sa.

ITV: Oke si nt’ap fè on klase enpòtans avantaj kisa ou te ka bay? Gwo avantaj? Gwo dezavantaj? Ti dezavantaj? dezavantaj fèb?

HM9: Gwo avantaj.

ITV: Ki benefis ou jwenn nan itilizasyon aparèy lan?

HM9: Se prèske menm bagay la m’ap redi ankò, emanatè a, a lèd de emanatè a ou pa depanse anpil kòb ankò nan plede achte blakatòks , begond, ki gen odè dezagreyab , nuizib ou konprann, emanatè a repouse tout moustik yo, li fè travay li.

ITV: Oke, si ou t’ap bay aparèy la on nòt ant zewo a dis, ki nòt ou te ka ba li, aparèy la?

HM9: M t’ap bay aparèy la uit paske li okipe twòp espas , sèlman sa.

ITV: HM9, avèk le tan ,èske ou santi aparèy la toujou efikas toujou? twa mwa apre kòman ou wè aparèy la?

HM9: De mwa aprè, aparèy la toujou gade menm efikasite l, paske odè yo mwen fò. Se te toujou le menm choz, li toujou repouse moustik yo, pa gen anpil moustik ankò

ITV: HM9, si ou gen dè rekòmandasyon pou moun k’ap dirije pwojè a kisa li t’ap ye?

HM9: M t’ap rekòmande pou yo fè emanatè aokipe mwens espas kote ou mòdènize l, ou ka rann li mwens ou konprann , jis pou moun ki gen timoun yo pou yo pa gen pwoblèm alavni

ITV: Oke HM9, se te on plezi pou nou te ansanm, nou kontan nou te fè ti pale sa, nou eskize nou tou paske nou pran tan ou ou te mete ou disponib pou nou,nou di ou on gran mèsi epi ou ba ou garanti, sa ou di yo, y’ap rete konfidansyèl, pèsonn moun p’ap ka idantifye di se Jezila ki te di yo, mèsi byen. Nou pran tout sa ou di yo an konsiderayon , n’ap gade nan ki mezi swivi yo ka fèt.

HM9: Ce fut un plaisir pour moi.

## Household 3 / member HM10 Male / Round 2

ITV: Mèt HM10 se on plezi maten an pou nou ansanm ak ou, nan kad seri antretyen yo, pou nou evalye aparèy la, pou nou jwenn nòt ou de aparèy la, pou nou konnen kijan sa ye bò kote pa ou.

HM10: Avan tou mwen salye komite TEAZ epi gwoup ekip pa m yo tou, lè kolektè ki ansanm avèk mwen, kisa m te kapab di o nivo de emanatè a, emanatè a li vrèman efikas, paske otrefwa lè nou kouche, kote ke marengwen ke konn anpeche nou dòmi, men depi lè emanatè a la bagay sa pa repete ankò.

ITV: Bon li trè byen? Li byen? Li bay tout pwoteksyon? Li pwoteje nèt? Nan ki nivo li pwoteje?

HM10: Bon, o nivo moustik lan li pwoteje men m’ap gade o nivo ou konnen m gen on tibebe paske li gen odè tou ki degaje ladan l, kote ke m ekate l prè timoun yo, m pa vle yo manyen l. Epi nan nivo ke m pa vle yo manyen l otrefwa li te pike ti pitit lan, fè yo te pike l, se la ke m kapab wè li se on dezavantaj men o nivo moustik, li anfòm.

ITV: Li pwoteje nèt? Li pwoteje byen? Trè byen?

HM10: Bon, li pwoteje byen.

ITV: Oke, nan ki nivo ou panse aparèy la pwoteje ou ak lòt moun lakay ou anndan kay la kont moustik ki anndan kay la?

HM10: Li pwoteje nou tout anndan , nan nivo kote l pwoteje nou tankou, mwen men m pa gen pwoblèm moustik ankò, timoun yo pa gen pwoblèm kote lè marengwen ap mòde yo, m pa konnen sa m sot di ou talè a la, sibstans ki ladan l nan genlè ekate marengwen yo menm deyò a dèfwa m konn chita deyò a, lè m konn a bout pantanlon kote maregwen konn ap mòde m, m pran emanatè a m nèk mete l deyò a, m chita, m pa santi, m pa gen nwizaib ankò pou m chita.

ITV: Alò, li pwoteje ou?

HM10: menm anndan, ni deyò.

ITV: Anndan e deyò. Èske ou ka site pou nou, dekri avantaj aparèy sa pote? Avantaj? Dezavantaj?

HM10: Avantaj aparèy la pote pou mwen, lè m kapab di le mwa, m konn depanse prè de senksan goud nan al achte kay abriko dè pwodui kont moustik, kounya m pa fè sa ankò. M konn al achte begonn, m konn flite anndan, nan on mwa m konn achte, prè de twa ou kat begond m pa achte l ankò, epi begonn nan tou lè m mete l se deyò m konn kite timoun yo epi m fèmen pòt la. M konn fè prè de inèdtan edmi avan ke begonn nan degaje apre pou m rantre byen ke begonn nan detui yo men nan demen ankò, fòk mwen flite egal chak swa m’oblije flite. Men avèk emanatè a, m pa bezwen, m reyalize ke m pa al achte begonn ankò, m pa achte dè pwodui ankò nan abriko, men dezavantaj ke l genyen se ke efè segondè an m pa vle li genyen li timoun nan, ou konnen m gen timoun, m gen bebe apèn gen sizan, m pa vle l manyenl lè l manyen l, si l mete men l nan bouch li, m pa konn kisa l’ap fè, li ka fè timoun nan. Epi jan m di ou an, gen de bagay ladan l tou ki ka pike bebe a, timoun yo. Mwen menm kòm granmoun, mwen toujou, m ka eseye jere sa, men timoun nan ou mèt di l non pa fè tèl bagay, se sa li eksite pou l fè. Se dezavantaj sa li genyen.

ITV: Bon si n te ka fon klasman nan gwo avnataj? Fèb avantaj? Ti avantaj? Gwo dezavantaj, kisa ou te ka chwazi?

HM10: M verifye se on gwo avantaj li ye.

ITV: Kisa ou jwenn kòm se pa avantaj non m te ka di, kò m…

HM10: Dezavantaj?

ITV: Pa dezavantaj non, benefis nan aparèy sa, ki benefis ou jwenn ladan?

HM10: Benefis la m sot di li talè a wi, se ekonomi ke m fè a, m te konn al achte begonn, lè pa gen kòb achte blakatòks, lè ou achte blakatòks la ou gendwa sòti ou wè, epi chalè ki soti nna blakatòks la, li nan mayo ou, odè a tou toujou rete e avèk pwodui sa ke n pa achte ankò a , se on avantaj ke l fè. Kifè ke avèk emantè a m pa achte dè pwodui sa yo ankò. M vin fè on ekip ekonimi akoz de emanatè a e m ta swete ke pou TEAZ elaji pou se pa sèl mwen ki pou benefisye de sa, gen lòt moun k’ap viv nan lòt kote, ann di pou TEAZ ta ouvè pwojè sa tou pou moun yo ka wè ki bèl travay TEAZ ap fè. A lèd de emanatè a

ITV: Bon, si ou te ka bay aparèy la on nòt ant zewo a dis, ki nòt ou te ka ba li?

HM10: M t’ap ba l uit sou dis, sak fè m bal uit sou dis m retire de pwen an se lefè ke emanatè a dèfwa li konn degaje on odè tou , odè li degaje an, m pa konn ki efè segondè li ka fè, si lè moun gen timoun piti epi lè y’ap fè l ti fil yo fè yo k’ap pike yo timoun nan se sak fè m redui na pwen an men o nivo de ekate moustik amanatè a li trè bon.

ITV: Oke, avèk le tan ,èske ou santi aparèy la toujou efikas toujou? twa mwa apre èske li toujou efikas? Pa efikas? mwens efikas?

HM10: Li toujou rete efikas men m raple m lè l te fèk trete an odè an te pi fò, men ofieamezi, ant de twa mwa m’ap gade li gentan bwè prè de kat mwa lakay la, odè a pa menm jan ankò. Men jan m di ou sa, lè l fèk trete an odè an pi bon, odè an li fò, e ou jwenn mwens moustik, lè l fèk trete.

ITV: Dakò, si ou ta gen dè rekòmandasyon pou moun k’ap dirije pwojè a kisa li t’ap ye? Si ou t’ap pwopoze yo, ba ya kèk konsèy kisa ou t’ap di yo?

HM10: Bon rekòmandasyon mta genyen jan m sot di ou sa la, tankou m ta renme pou TEAZ elaji li pou plis moun konnen TEAZ, ki bon travay l’ap fè, a lèd de emanatè a.

ITV: HM10 se te on plezi pou nou te ansanm, nou kontan nou te fè ti pale sa, nou eskize nou tou paske nou pran tan ou, nou di ou on gran mèsi epi ou ba ou garanti, sa ou di yo, y’ap rete konfidansyèl, pèsonn moun p’ap ka idantifye di se ou ki te di yo, bòn kontinuite avèk aparèy la, mèsi byen.

HM10: M remèsye TEAZ tou, okontrè, se mwen ki pou remesye TEAZ pou tèt pwojè sa ki rive pa bò kote pan ou se nou ki gen chans jwenn li, epi pou TEAZ elaji espas lan pi laj.

ITV: Dakò, mèsi HM10.

# IDI Block 2

## Household 1 / member HM14 Male / Round 1

ITV: Nou pral kòmanse avèk ou, nou a HM14, nou lakay ou maten an la, nou pral poze ou kèk kesyon sou satisfaksyon ou konsènan emanatè a ou gen lakay ou itilize. Premye kesyon an se nan ki nivo ou panse emanatè a pwoteje ou kont marengwen k’ap mòde ou lè ou nan kay la? Gen plizyè repons la yo pwopoze, ou ap chwazi sa ou wè ki apwopriye. Èske li pa pwoteje ou? Si wi ou ap chwazi sa, si li pwoteje ou tou piti, ou ap chwazi sa, si li byen, si li trè byen, si li pwoteje nèt ou ap chwazi sa, ou ap jis fè on kwa, si pa gen repons tou, ou ap chwazi pa gen repons, ou ap fè on kwa la. Trè byen li pwoteje ou byen.

ITV: Dezyèm kesyon an. Nan ki nivo pou l ta pwoteje ou , nan ki nivo pou emanatè ya ta pwoteje ou ak lot moun lakay ou kont lὸt vèmin yo, lè ou nan kay la? Fè yon ti wonn nan youn nan repons ki pi ba yo, ou mèt toujou fè on kwa jan ou te fè anlè a,di nan ki nivo ou ta renmen pou l ta pwoteje ou.

HM14: Nèt, nèt.

ITV: Ebyen vwala 3-Nan ki nivo emanatè a pwoteje ou kont marengwen lè ou deyὸ nan lakou a? Fè yon ti wonn nan sa ki pi ba yo oswa sèke nὸt ki anba la. Èske pa gen repons? Èske li pa pwoteje ou lè ou deyὸ nan lakou a?

HM14: Non.

ITV: Èske se tou piti, ebyen ou ap chwazi repons la. Avan m poze ou lὸt kesyon kesyon an, èske ou konn mete emanatè a deyὸ?

HM14: Non, m pa konn mete l’ deyὸ.

ITV: Ou kite anndan sèlman?

HM14: Anndan. Pou byen di ou oparavan lè m te konn mete l bὸ kote m dὸmi an m pat konn santi marengwen yo, pou kounya la, se kὸmsi marengwen yo se lavironnaj y’ap fè sou tèt mwen.

ITV: Sa vle di ou gen enpresyon efikasite a diminye avèk tan.

HM14: Wi.

ITV: Men lè ou ap deplase konsa, si ou ap travay on lὸt kote ou pa mete emanatè a bὸ kote ou? Ou jis kite l on sèl kote nan kay la?

HM14: M kite l, okontrè se de m genyen, se de yo ban mwen, m kite youn bὸ kote m nan, m kite youn nan chanm bὸ kote nan chanm kote frè m nan ye a.Li menm m pa konnen kὸman pa l la ye, men pa m nan oparavan li te bon, men kounya la se kòmsi se on bagay ki mande aspèje.

ITV: M konprann,ou gen enpresyon sibstans yo mete ladan l, transkriptin nan lan fini, li ka redui. Katriyèm kesyon an, nan ki nivo emanatè a pwoteje ou ak fanmi ou kont lὸt vèmin yo lè ou deyὸ kay la? Fè on ti wonn nan repons ki pi ba oswa chwazi pa gen repons, lè ou deyὸ kay la an ki nivo li pwoteje ou.

HM14: M ka di kòman, non, paske se deyὸ.

ITV: Ou wè l pa pwoteje ou, se lojik.

5: Site ak dekri nenpὸt lot benefis oswa dezavantaj emanatè a bay. Ki benefis emanatè a bay, ki dazavantaj, ou mèt di yo la an kreyὸl, ki benefis emantè a bay? Kisa ki pa bon ou santi li bay?

HM14: M ka di pou benefis, akoz de marengwen yo, m ka di la nou te konn jwenn anpil marengwen akoz de dlo. M ka di lè l te fèk vini an m pat jwenn marengwen. Men kounya la m ka di nou jwenn marengwen agogo wi.

ITV: Nou itikize plakatὸks?

HM14: Non, m pa itilize bagay sa yo, m pa achte l. Nou pa itilize l, bon bagay sa m p’ap menm itilize l paske m ka pran odè a, depi m pran odè l m gentan anrime.

ITV: Plakatὸks la?

HM14: Wi.

ITV: Ki dezavantaj ou jwenn?

HM14: M pa ka di m jwenn dezavantaj, pa gen dezavantaj pou mwen menm.

ITV: Se sa ou t’ap di kὸm benefis, pa gen lὸt avantaj?

HM14: M panse se tou wi.

ITV: Ebyen n’ap kontinye. Klase enpòtans avantaj, dezavantaj, ou sot di avantaj kounya ou pral klase avantaj sa yo, kὸm ou pat di dezavantaj, m pa bezwen mete dezavantaj. Èske gen avantaj fèb? Èske gen ti avantaj? Èske gen gwo avantaj? Ou ap fè on kwa oubyen on ti wonn. Ou chwazi gwo avantaj.

ITV: 7 la, site ak dekri nenpὸt lὸt benefis oswa dezavantaj ke li bay. Talè a nou te fè benefis, kounya èske gen lὸt benefis ankὸ ou jwenn li bay?

HM14: Lὸt benefis?

ITV: Wi.

HM14: Ou kwè? On kote, kὸm se manman m ki tou prè kote m dὸmi an okontrè se li ki te ka banm on ide sou bagay sa, pa konnen si li pa…

ITV: M pral pale avè l tou talè.

HM14: Se sa k’ fèm di ou paske mwen menm kote m ye a, m mete l bὸ kote m nan de tanzantan m chanje l bὸ m gen espas kote m ka mete l la, m ka mete l’ la. Li menm kote l ye a, li tou prè m wi men m pa konnen si li jwenn marengwen jan pou l’ta jwenn ou byen si li pa jwenn tou, ou wè sa m di ou la.

ITV: Ebyen dakὸ, n’ap vin sou li aprè.

HM14: Pou mwen menm, m ka di gen lὸt avantaj.

ITV: Ebyen, eseye bay yo.

HM14: Lὸt avanataj m ka di genyen, m ka di gen on epὸk marengwen,bon kὸm m ka di lapli pa tonbe, si lapli te tonbe se lè sa m te ka di nou te ka jwenn plis marengwen. Se lè sa tou m te ka di èske emanatè a fè travay li serye ak si li pa fè l tou. M pa konn si nou konprann nan sans mwen ye ya non?

ITV: M wè sa wi.

HM14: Sa vle di pou mwen menm, emanatè a son gwo avantaj li ye pou mwen ki vin nan kay la, son gwo avantaj li ye pou nou, sa k  fè m di sa se paske ouparavan lè l te fè k vini an m pa t tèlman gen marengwen men pou kounyeya m pa di m jwenn li agogo non men gen kèk grenn tankou lè m’ap dὸmi bὸ tèt mwen k’ap siyonnen, okontrè lè konsa m pa konn dὸmi kouvri ,kounyeya m dὸmi kouvri, mwen m se pa yon moun ki renmen kouvri lè m’ap domi.

ITV: Ou kouvri pou moustik pa vin sou ou?

HM14: Wi, m pa renmen kouvri, kounya la, m kouvri agogo. Menm lè marengwen pa tèlman anpil wi, men m kouvri. Men pou m byen di ou genyen avantaj la, li pa tèlman minim non, men li on ti bagay.

ITV: Dakὸ, ou vle ekri sa ou di yo oubyen?

HM14: Pa gen pwoblèm non.

ITV: Ebyen ekri yo, ou mèt pran sa. Nou pral nan uityèm kesyon an, klase enpὸtans avantaj sa pa youn nan repons ki anba sa yo,oswa nenpὸt nan bagay sa yo. Ou ka di pa aplike, ou ka di pa gen repons kὸm ou pat pale de dezavantaj ou jis pale de avataj, ebyen ou ap klase avantaj la.Èske se on avantaj ki fèb? Ti avantaj ou byen gwo avantaj, ou ap chwazi.

ITV: oke nan nèf la, site ak dekri nenpὸt lὸt benefis ak dezavantaj ke emanatè a bay. M kwè sèt la avèk nèf la se menm bagay, men èske ou gen lὸt bagay ou ap di sou avantaj oubyen dezavantaj?

HM14: Non.

ITV: Ebyen dis la, klase enpὸtans avataj, dezavantaj sa yo pa youn nan repons ki anba yo. Oswa nenpὸt nan bagay sa yo, pa aplike, oubyen pa gen okenn repons kisa ou ap chwazi? Kὸman ou ap klase avantaj sa yo? Èske se avantaj ki fèb? Èske se on ti avantaj? Èske se on gwo avantaj? Ebyen mèsi a HM14 pou kolaborasyon ou, kounya nou pral pran on dezyèm moun nan kay la.

## Household 1 / member HM21 Male / Round 1

ITV: Nou pral kὸmanse avèk kesyon yo, premye kesyon an, nan ki nivo ou panse emanatè a pwoteje ou kont marengwen k’ap mὸde ou lè ou nan kay la? Ou ap chwazi youn nan repons sa yo.

2) Dezyèm kesyon, nan ki nivo pou l ta pwoteje ou ak lὸt moun nan kay la?

3) Twazyèm kesyon, nan ki nivo emanatè ya pwoteje ou kont marengwen lè ou deyὸ nan lakou a, èske li pa pwoteje ou?

4) Katriyèm kesyon, nan ki nivo emanatè a pwoteje ou ak fanmi ou kont lὸt vèmin yo, lè ou deyὸ kay la? Èske li pa pwoteje ou lè ou deyὸ kay la?

5) Site ak dekri nenpot lὸt benefis oubyen dezavantaj li bay nan kay la, emanatè a. Ou ka ekri yo, ou ka site tou.

HM21: M pa bezwen ekri yo?

ITV: Ou ka ekri yo, ou ap ekri yo pito? Ou ka site yo tou.

HM21: Se sa m t’ap al di wi, paske nou pa konn ki tip de marengwen k’ap mὸde nou, e chak marengwen ki mὸde nou gen on tip de maladi li ba nou. Alὸ sa se on avantaj li ye pou nou lè nou genyen. Kὸman ou di materyèl la rele ankὸ?

ITV: Emanatè.

HM21: Emanatè a li anpeche marengwen mὸde nou, sa se on avantaj li ye, li pa on dezavantaj. Se on anvantaj li ye, li anpeche marengwen mὸde nou, fè nou pa ka pran maladi.

ITV: Dakὸ, kὸm ou di l, ou pa bezwen ekri l. Ou pale de avantaj.

6) Klase avantaj sa,enpὸtans avantaj la.Èske se on avantaj ki fèb? On ti avantaj? Oubyen on gwo avantaj? Chwazi youn nan repons sa yo.

7) Site ak dekri nenpὸt lὸt benefis oswa dezavantaj ke emanatè a bay.

HM21: Lὸt benefis?

ITV: Wi.

HM21: Se toujou menm bagay yo, benefis la se toujou menm bagay yo se anpeche lὸt marengwem pike nou, dayè m son moun ki nan domèn nan, m travay nan domèn sitou sou afè marengwen an tou. Emanatè a li enpὸtan paske li memn li anpeche lot marengwen mὸde n, sa toujou, se li m’ap toujou kenbe kὸm repons. Chak marengwen ki mὸde nou li gen on tip de maladi li lage nan kὸ nou.

ITV: Wi, filaryoz se yo.

8) Klase enpὸtans avantaj oubyen desavantaj sa ou wè l bay l.Èske se on avantaj ki fèb? Èske se on ti avantaj? Èske son gwo avantaj?

9) Nan nèf la, ou ap site ak dekri nenpὸt lὸt benefis ou wè li bay oubyen dezavantaj ou wè l bay nan kay la, emanatè a.

HM21: Nenpὸt lὸt benefis li bay?

ITV: Wi, ou ka li repons la pou mwen?

HM21: Li pwoteje nou, li rann nou gen on sante plizoumwen.

ITV: Dis la se, klase enpὸtans avantaj sa ou wè li bay la, chwazi youn nan repons anba la. Ebyen dakὸ, mèsi pou kolaborasyon ou.

## Household 1 / member HM22 Female / Round 1

ITV: HM22, nou pral kὸmanse avèk kesyon yo, premye kesyon an, nan ki nivo ou panse emanatè a pwoteje ou kont marengwen k’ap mὸde ou lè ou nan kay la? Èske li pa pwoteje ou? Èske se tou piti? Byen? Trè byen? Pwoteje nèt? Kisa ou ap chwazi nan tout repons sa yo? ÈskeREC se proteje byen? tou piti? Byen? Trè byen? Pwoteje nèt?

HM22: Bon, m pral di, kὸm m pat konnen pou kisa, yo kite l, m pa konnen poukisa, m wè l la, pou byen di ou marengwen, m pa prèske santi, men avan yè swa m te santi youn k’ap pase, alὸ m pa konnen non si se, m pat konnen si se…

ITV: Emanatè a

HM22: M pa konn kouvri, gen on lè m pa t kouche san m pa kouvri, menm sim pa ta kouvri, marengwen fè m kouvri men m pa tèlman santi marengwen pou kounyeya.

ITV: Men èske ou santi li pwoteje ou byen, trè byen oubyen nèt?

HM22: M ka di byen.

ITV: Ebyen m ap chwazi byen an pou ou.

2). Nan ki nivo pou l ta pwoteje ou? Èske ou ta renmen li pwoteje ou nèt?

HM22: Wi.

ITV: Ebyen dakὸ, nan ki nivo emanatè a pwoteje ou kont marengwen lè ou deyὸ nan lakou a, èske li pa pwoteje ou? Èske li pwoteje ou tou piti? Byen, trè byen oubyen nèt lè ou deyὸ nan lakou a?

HM22: Non, li pa pwoteje nèt.

ITV: Ebyen dakὸ, nan ki nivo emanatè a pwoteje ou ak fanmi ou kont lὸt vèmin yo, lè ou deyὸ kay la? Èske li pa pwoteje ou lè ou deyὸ kay la? Èske li pwoteje ou tou piti? Byen? Trè byen? Oubyen èske li pwoteje ou nèt lè ou deyὸ kay la ak fanmi ou? Lè ou deyὸ kay la , èske ou santi emanatè a pwoteje ou?

HM22:Kὸm nan lakou a m pa konn wè tèlman gen lὸt bèt, menm sourit, m pa prèske wè yo tou , m pa konn si se li menm.

ITV: Wi, lὸt vèmin nan , yo ta dwe presize kisa sa vle di,yo ta dwe bay egzanp, kont lὸt ensèk li ka mouch.

HM22: M ka di enpe, li pwoteje enpe.

ITV: Tou piti?

HM22: Wi.

ITV: Dakὸ, èske ou ka bay lὸt benefis oubyen dezavantaj li bay nan kay la, emanatè a. Ki avantaj li bay nan kay la, emanatè a oubyen ki dezavantaj li pote.

HM22: M pa ka di ezavantaj, kὸm m te di ou m pat konnen l, poukisa li la , m ka di li gen on avantaj kanmenm, li gen on ti avantaj.

ITV: Dakὸ. Nou prale nan kesyon 6, ou di gen on ti avantaj. Klase avantaj sa pa youn nan repons ki anba yo. Èske se on avantaj ki fèb? On ti avantaj? Oubyen on gwo avantaj?

HM22:Èske kisa?

ITV:Si se on gwo avantaj oubyen on avantaj fèb?

HM22:M ka di fèb.

ITV: Dakὸ. Site ak dekri nenpὸt lot benefis oswa dezavantaj ke emanatè a bay.

HM22: M kwè m te di ou li, m pa ka di dezavantaj. M pa ka di dezavantaj ditou, si gen on bagay gen on avantaj kanmenm li bay, li pa ka bay dezavantaj m pa wè sa.

ITV: E ki avantaj ou wè li bay?

HM22: Bon toujou menm bagay yo, marengwen, mouch pa tèlman, m pa wè mouch tèlman, gen de lè mouch konn ap bay pwoblèm, ou te gendwa la ou pa ka rete avèk mouch. M pa tèlman wè mouch, sa vle di gen on ti avantaj kanmenm.

ITV: Dakὸ, nou prale nan kesyon 8 la, klase enpὸtans avantaj sa ou wè l bay la. Èske se on avantaj ki fèb? Èske se on ti avantaj? Èske son gwo avantaj?

HM22: Bon, pou mouch yo , m ka si son avantaj menm si li pa tèlman fin nèt gen on avantaj kanmenm.

ITV: Kisa ou ap chwazi nan tout avantaj la? Èske se on avantaj ki fèb? Se on ti oubyen on gwo avantaj? Daprè oumenm.

HM22: M ka di fèb la, m ka di ti.

ITV: Oke, nevyèm kesyon site ak dekri nenpὸt lὸt benefis oubyen dezavantaj ke emanatè a bay?

HM22: Benefis?

ITV: Ki lὸt benefis li bay?

HM22: Benefis? Kὸmsi sou afè marengwen an toujou?

ITV: Wi, oubyen nenpὸt lὸt aspè.

HM22: M pa wè marengwen non , m prèske dὸmi menm kote ak HM14, m ka di ou m ap wè. Petèt kote l kouche a, li ka jwenn ti bagay, men mwen menm m pa jwenn, m dὸmi san kouvri.

ITV: Dis, klase enpὸtans avantaj sa ou wè li bay la, pa egzanp ou di ou dὸmi san kouvri. Èske son avantaj ki fèb? On ti avantaj? Oubyen on gwo avantaj?

HM22: M pa ka di li fin bagay nèt men kanmenm li prèske a san pou san paske marengwen yo te konn anpeche m dὸmi, kounyeya kanmenm m ka di ,m pa prèske wè sa.

ITV: Dakὸ men nan tout avantaj sa yo kisa ou ap chwazi? Èske son avantaj ki fèb? On ti avantaj? Oubyen on gwo avantaj?

HM22: M p’ap fin di li gwo. Bon ou mèt di l gwo.

ITV: Dakὸ, mèsi madam HM22 pou patisipasyon ou, kounya nou pral pran on twazyèm moun nan fwaye a.

HM22: Dakὸ.

## Household 2 / member HM20 / Round 1

ITV: Nou pral kòmanse, malarezman gen de moun ki absan, m pa konn kòman sa fè men ta dwe gen twa moun ki patisipe nan antretyen sa la. Nan ki nivo ou panse emanatè a pwoteje ou kont marengwen k’ap mòde ou lè ou nan kay la? Èske li pa pwoteje ou? Èske li pwoteje ou tou piti? Byen? Trè byen? oubyen pwoteje nèt?Ou ap chwazi youn nan repons sa yo.

HM20: Li pwoteje trè byen.

ITV: Ebyen ou ap chwazi sa la. Ou ap fè on kwa la.

HM20: Anba l ou byen la?

ITV: Sou li. Jan ou vle.

HM20: Anba kat la?

ITV: Depi ou fè on sin sou li. Oke, nan ki nivo pou l ta pwoteje ou  ak lòt moun lakay ou kont lὸt vèmin yo, lè ou nan kay la? Nan ki nivo li ta dwe pwoteje ou? Èske li ta dwe pwoteje ou nèt? Byen? Tou piti oubyen li pa pwoteje ou?

HM20:Wi, li pwoteje m trè byen.

ITV: Non, nan ki nivo li ta dwe pwoteje ou?

HM20: Li te dwe pwoteje m nèt ale.

ITV: Vwala, ebyen ou ap chwazi repons sa. Fè on kwa la.

3- Nan ki nivo emanatè a pwoteje ou kont marengwen lè ou deyὸ nan lakou a? Si pa gen repons  ou ap di pa gen repons. Si li pa pwoteje ou , ou ap di sa. Si li tou piti ou ap di sa. Si se byen ou ap pran sa, si se trè byen ou ap pran sa, si li pwoteje ou nèt tou ou ap di sa.

HM20:Lè m andedan m wè li bagay andedan avèk deyò a.

ITV: Wè, lè ou anndan , kòman sa ye?

HM20: Wi, m ka wè, pou mwen menm li plis pwoteje m pase deyò a, paske andedan li avè m.

ITV: M konprann, men ou pa konn mete emanatè a deyò?

HM20: Non, pwoblèm ki koz la lè yo te vin ban ou travay la avèk lè yo te vin rebanou l ankò, yo pat fè seminè pou yo te di nou fò n mete l.

ITV: Kòman pou itilize l.

HM20: Yo pa t din ou sa, mwen yo te ban mwen l, m te vle pwoteje li, se on materyèl li ye, fò m pwoteje l byen.

ITV: Men yo te di ou poukisa li la sèlman, pou chase moustik?

HM20: Wi.

ITV: Men yo pat di ou kòman pou ou te itilize l?

HM20: Yo pa t di nou kòman pou nou itilize li.

ITV: Yo pa t pase wè ou? Pa t gen sipèvizyon?

HM20: Lè yo vin kite l la?

ITV: Lè yo vin kite l la.

HM20: Non, m pa t jwenn sa.

ITV: Dakò, pou deyò lakou a, èske li pa pwoteje? Èske se tou piti? Èske se byen, trè byen? Pwoteje nèt? Kisa ou ap pran nan repons sa yo la?

HM20: Li pwoteje trè byen?

ITV: Lè ou deyò lakou a?

HM20: wi.

ITV: Ou santi sa?

HM20: Wi

ITV: Donk kat, nan ki nivo emanatè a pwoteje ou ak fanmi ou kont lὸt vèmin yo, lè ou deyὸ kay la? Lè ou deyὸ kay la sou lakou a pa egzanp, èske li pwoteje ou?

HM20: Li pwoteje paske gen lot bagay, si pa egzanp nou t ap achte, gen moun ki itilize plakatòks la paske on sèl grenn vann di goud, imajine, on sèl grenn pa sifi. Si ou t’ap achte l nenpòt kat, nenpòt twa pou on si dola, sa li pwoteje nou se ekonomi nou fè. Li pi bon pou mwen. Kounyeya la m wè l pwoteje m anpil,

ITV: Dakò, men daprè repons yo bay la, èske se tou piti,? Si li pwoteje byen oubyen trè byen?

HM20: Trè byen.

ITV:Ebyen, ok ,kounyeya ou sot pale de avantaj. Klase avantaj sa pa youn nan repons ki anba yo. Èske emanatè a se on gwo avantaj li bay?Èske se on ti avantaj? Èske se on avantaj ki fèb?

HM20: Gwo avantaj.

ITV: Dakò. Site ak dekri nenpot lὸt benefis oswa dezavantaj li bay nan kay la, emanatè a. Ki lòt benefis emanatè a bay?

HM20: Lòt benefis m wè l bay, se toujou menm sa m sot reponn yo. Sa m sot di yo la. Se sèl benefis sa m wè li pote.

ITV: Dakò. Ou pa wè dezavantaj?

HM20: Non, m pa wè dazavanyaj ditou. Se avantaj m wè l pote.

ITV: Dakò. Kòm ou di se benefis oubyen avantaj ou wè li bay, kòman ou t’ap klase avantaj sa yo? Èske se on avantaj ki fèb? On ti avantaj? Oubyen on gwo avantaj?

HM20: On gwo avantaj.

ITV: Site ak dekri nenpot lὸt benefis ou wè emanatè a bay?

HM20: Se benefis sa yo m wè l bay paske sou tout lòt, emanatè a paske li vini men jan avèk travay la li vini ak tout bagay, sou sa , m ka di se on benefis. Paske se li menm ak tout bagay , m wè se de emanatè a y’ap pale paske li bay anpil benefis. Sou menm jan m ta di sou aktivite travay la.

ITV: Benefis, sa vle di pou ou men koman ou santi li pwoteje ou nan kay la pa egzanp?

HM20: Li pwoteje m nan tout sans. Pa egzanp ni sou travay la, menm m kapab di nan kay la tou, nan vwazinaj yo tou li pwoteje, pa egzanp menm lè nou la, menm vwazin yo gendwa mande esplikasyon , ou ba l esplikasyon sou sa. Ou pale avè yo sou sa, kounya yo di se on bon bagay. M wè li pwoteje n avèk vwazinaj yo tou, avèk lòt moun nan anviwònman tou.

ITV:Klase enpòtans avantaj sa.Èske se on avantaj ki fèb? On ti avantaj oubyen on gwo avantaj?

HM20: On gwo avantaj.

ITV: Mèsi mesye… pou patisipasyon ou,m espere lòt de moun yo ap fè on fason pou yo entèvyouwe yo tou paske omwen nou ta ka pran de moun, on sèl moun sou twa li pa sifi paske m wè fòk se twa moun ki pou patisipe.

## Household 3 / member HM17 Female / Round 1

ITV: HM17 nou pral kòmanse avèk kesyon yo, nan ki nivo ou panse emanatè a pwoteje ou kont marengwen k’ap mòde ou lè ou nan kay la? Na ki nivo? men repons yo la, si li pa pwoteje ou, ou ap di sa, si se byen, trè byen, pwoteje nèt ou ap chwazi repons ki bon pou ou a.

HM17: Bon, premye fwa m te mete l la, m ap konn se paske te gen anpil, te gen imidite, m pa t vrèman wè yo, men depi semèn nan kòmsi m wè kèk grenn dè fwa.

ITV: Avan?

HM17: Li te elwaye yo, men pandan semèn sa kòmsi m wè kèk grenn.

ITV: Pandan konbyen tan konsa ou wè l te elwaye moustik yo? Na on semèn oubyen 15 jou?

HM17: Wi.

ITV: Ou ka bay on ide?

HM17: Kòmsi jan yo te konn ap toumante ou, lè ou chita fè pye ou cho. Lè ou ap dom itou, ou konnen se zòrèy yo renmen, y’ap rele nan zòrey ou. Kòmsi lè ou pral dòmi ou mete l, m pa vrèman wè yo.

ITV: Men kounya, ou wè yo fon tounen.

HM17: M wè kèk grenn.

ITV: Kòman ou wè l pwoteje ou? Ou ap chwazi youn nan repons sa yo. Ou ap chwazi youn.

HM17: Li pwoteje m byen.

ITV: Dakò. 2- nan ki nivo pou emanatè a pwoteje ou ak lòt moun nan kay la kont lὸt vèmin yo, lè ou deyὸ kay la? Nan ki nivo pou l ta pwoteje ou? Ou ta renemen l pwoteje ou? Men repons yo.

HM17: Kanmenm li pwoteje m lè m mete l.

ITV: Non sa se kòmsi on bagay ou ta swete. Nan ki nivo ou ta renmen li pwoteje ou?

HM17: Pi byen toujou.

ITV: Ou ap chwazi youn nan repons sa yo.

HM17:Trè byen.

ITV: 3- Nan ki nivo emanatè ya pwoteje ou kont marengwen lè ou deyὸ nan lakou a? Men repons yo, ou ap chwazi sa ki bon pou ou a. Lè ou nan lakou a, èske ou wè emanatè a ba ou pwoteksyon kont moustik?

HM17: M pa konn twò deyò, m pa ka di ou.

ITV: Ou pa konn tèlman deyò?

HM17: Ebyen ou ap chwazi pa gen repons.

ITV: Ou fèmen anndan ou ap.. sa pa na ankèt la men fòk nou ri tou. 4-, nan ki nivo emanatè a pwoteje ou ak fanmi ou kont lὸt vèmin yo, lè ou deyὸ kay la?

HM17:Deyò kay la?

ITV: Wi.

HM17: Ebyen se toujou menm jan wi.

ITV: Dakò. Site ak dekri nenpot lὸt benefis oswa dezavantaj emanatè a bay? Ki lòt benefis?

HM17: Trè byen toujou.

ITV: Ki lòt benefis ou wè l bay?

HM17: Benefis, ka di marengwen pa mòde mwen.

ITV: E dezavantaj?

HM17: Dezavantaj?

ITV: Kisa ki pa mache, kisa emanatè a bay kòm pwoblèm?

HM17: M pa remake gen pwoblèm.

ITV: Menm lè ou pa tèlman detaye sou avantaj emanatè a bay, èske se on avantaj ki fèb? Oubyen on ti avantaj li bay oubyen on gwo avantaj?

HM17: On gwo.

ITV: Ou ap chwazi.

HM17: Gwo avantaj.

ITV: Ebyen fè on kwa.

HM17: Make l?

ITV: Fò ou gen anpwent ou la tou.

HM17: On kwa pou m fè?

ITV: On kwa wi. Site ak dekri nenpot lὸt benefis oswa dezavantaj emanatè a bay.

HM17: Benefis, sèke gen de maladi m p’ap genyen.

ITV: Pa egzanp?

HM17: Kòm malarya, tout maladi ke moustik la pote.

ITV: Dakò. Ki dezavantaj ou wè li bay?

HM17: M pa twouve non.

ITV: Dakò, tout avantaj sa yo, ou sot pale a la, èske se on avantaj ki fèb? Oubyen on ti avantaj? Oubyen on gwo avantaj?

HM17: Gwo.

ITV: Ou mèt chwazi l. Site ak dekri nenpot lὸt benefis emanate a bay nan kay la.

HM17: Benefis?

ITV: Ou pale de benefis, avantaj se menm bagay.

HM17: Ebyen se toujou menm bagay la toujou wi. Se menm bagya yo ankò wi.

ITV: Dakò, e kòman ou ka klase avantaj sa?Èske se on avatanj ki fèb? Oubyen on ti avantaj? On gwo avantaj?

HM17: Gwo.

ITV: Ebyen mèsi HM17 pou patisipasyon ou, kay la se sèlman de moun ki gen ladan, nou pa ka envante, nou pa ka kreye on lòt moun.

## Household 3 / member HM18 Male / Round 1

ITV: Mesye HM18, n ap kòmanse avèk kesyon yo, alò nan ki nivo ou panse emanatè a pwoteje ou kont marengwen k’ap mòde ou lè ou nan kay la? Èske li pa pwoteje ou? Èske li pwoteje ou tou piti? Byen? Trè byen? oubyen pwoteje nèt? Ou ap chwazi youn nan repons sa yo.

HM18: Li pwoteje m byen.

ITV: Ebyen dakò, ou ap chwazi repons twa.

HM18: Wi twa.

ITV: 2- nan ki nivo pou l ta pwoteje ou ak lòt moun yo nan kay la? Èske se tou piti? Byen? Trè byen? Pwoteje nèt?

HM18: Pwoteje nèt.

ITV: Dakò, 3- Nan ki nivo emanatè a pwoteje ou kont marengwen lè ou deyὸ nan lakou a? Nan repons yo la, ou ap mete repons ki apwopriye. Lè ou deyò, na lakou a li pwoteje ou?

HM18: Wi, li pwoteje m byen.

ITV: Dakò, 4- nan ki nivo emanatè a pwoteje ou ak fanmi ou kont lὸt vèmin yo, lè ou deyὸ kay la?

HM18: Li pwoteje m trè byen.

ITV: Lè ou deyò a?

HM18: Lè m deyò a.

ITV: Oke, kounyala ou ap klase enpòtans avantaj sa yo la, sa ou sot di a la. Èske se on avantaj fèb oubyen on ti avantaj? Oubyen on gwo avantaj emanatè a bay nan kay la?

HM18: Li bay on gwo avantaj.

ITV: Èske ou ka esplike m poukisa se on gwo avantaj?

HM18: Paske lè m itilize li, li kapte marengwen yo, yo pa mòde m e m santi m byen.

ITV: Dakò. Site ak dekri nenpὸt lot benefis oswa dezavantaj ke emanatè a bay nan kay la. Ki lòt benefis li bay nan kay la?

HM18: Benefis li ban mwen?

ITV: Wi.

HM18: Li mete m an sekirite, sa vle di m pa pè, m santi m alèz.

ITV: Sa ou pa pè a pa egzanp?

HM18: M pa pè paske marengen pa ka mòde m, ni pot maladi tou. Sa k pou ban m maladi an, li evakye, pa rapò jan yo te ye a li pat konsa.

ITV: Dakò, Klase enpòtans avantaj sa. Èske se on avantaj ki fèb? On ti avantaj oubyen on gwo avantaj?

HM18: On gwo avantaj.

ITV: Dakò, Site ak dekri nenpot lὸt benefis ou wè emanatè a bay? On lòt benefis ou wè l bay nan kay la.

HM18: Lòt benefis m te ka di, sa vle di, pou jan m ta di emanatè a fonksyone, depi m mete li m pa kouvri, sa vle di m pa jwenn marengwen k’ap mòde m, k’ap annwiye m jiskan pou yo mòde m , pou m pè.

ITV: Èske gen dezavantaj ou wè, si ou wè gen dezavantaj ou pa bezwen pè di l.

HM18: M pa genyen, m pa pè di l.

ITV: Ou pa gen dezavantaj? Li pa bay pwoblèm?

HM18: Ki pwoblèm pou li ta bay? Sa vle di m pa wè li bay pwoblèm non.

ITV: Dakò, ou pale de avantaj, èske se on avantaj ki fèb? Èske se on ti avantaj oubyen on gwo avantaj?

HM18: Gwo avantaj.

ITV: Ebyen, mèsi HM18 pou kolaborasyon ou. Ou fè on bon antretyen sitou ou ban ou enfomasyon ki ase orijinal, sitou genyen se premye gwa, tande, ou pale de sekirite, se trè byen.

## Household 1 / member HM16 Female / Round 2

ITV: HM16, li enpòtan pou nou kesyone tout moun, ni moun ki te itilize aparèy la, ni sak pa t itilize l donk fò nou konnen pou ki rezon moun nan pa t’itilize l’.Èske ou ka esplike noupou kin rezon ou pa t itilize aparèy la?

HM16: Bon, mwen itilizason an, se kounya y‘ap fè m konprann kòman itilizayon an ye, yo di m depi l nan kay la m’itilize l kanmenm men, m pa t itilize l paske nòmalman m konnen fòk li ta sipoze nan chanm moun nan men yo di m kèlkeswa kote l plase depi l la, pandan l la m itilize l kanmenm m pa konnen sa rann mwen kont.

ITV: Ou itilize l enkonsyaman.

HM16: Wi.

ITV: Èske ou santi aparèy la pote pou ou yon pwoteksyon? Si ou di ke ou itilize l enkonsyaman? Èske ou panse ke li pwoteje ou? Nan ki nivo ou panse li pwoteje ou? Èske li pwoteje ou trè byen? Èske li pwoteje ou nèt? Li pwoteje ou tou piti?

HM16: M ka di si l la pou pwoteje m, li pwoteje tou piti, paske mwen menm kote m ye a m toujou jwenn marengwen.

ITV: Marengwen yo toujou mòde ou?

HM16: Wi.

ITV: Donk li pa nan chanm ou, marengwen toujou mòde ou?

ITV: Ou pa mete l nan chanm kote ou ye a? Jamè ou pa janm fè sa?

HM16: M pa t konnen.

ITV: Li pa t konnen itilizasyon l. Deyò kay la tou ou pa t konn itilize l jamè deyò kay la?

HM16: Men m pat konn mete l deyò. M pa t konnen vrèman egzakteman paske se kounya ,yo di m ke menm si li la,depi m chita la,m itilize l kanmenm ,bon m pa t konnen itilizazyon an èske se te kèlkeswa kote m pase oubyen se teon bagay pou m te mete ladan l pou m itilize l.

ITV: Men ou pat enfòme de itilite aparèy la,yo pa t di enfòmeou de sa?

HM16:Non, Mwen menm m tande li la,kòmsi m panse se te an tèm travay ,kòmsi yo mete li la, yo soti avè l,yo mete tout bagay yo, tout sa ki genyen pou bagay marengwen yo,m panse se te pou sa, se konsa kòmsi itlizasyon an ye,kòmsi li menm sèlman pou kont li, se on fò m itilizasyon li te ye mwen m te panse fò l te akonpaye de tout lòt aparèy ki sipoze bay yo,mwen pandan l la m pa t konnen kòmsi li menm sèlman pou kont li, se on fò m itilizasyon li te ye.

ITV: Endirèkteman, kisa aparèy la pote pou ou kò m avantaj paske ou pa t itilize l men endirèkteman, li ka pote pou ou kèk avantaj, kisa yo ye si genyen?

HM16: Kèk avantaj, èske m ka di kèk avantaj paske m son moun m pa vrèman chita la sa vle di m pa ka di anyen egzakteman de li vrèman,paske kote l ya kòmsi m pa on moun ki toujou chita ,ki estab kote l ye a. Mwen pou m ta wè li pote on bagay pou mwen vrèman fòk se kote m ta ye a m toujou itilize l mwen menm pou m wè vrèman èske li efikas ou pa,men m pa vrèman, kote l ye a m pa vrèman kòmsi twò rete sou li.

ITV: Oke men èske ou pat enfòme de itilite aparèy la? Ou pa t jwenn okenn enfòmasyon o sijè de itilite aparèy la?

HM16: M byen di ou wi, m pa t konnen vrèman, m tande yo di menm non an m pa t konnen, m pa t konnen si se li yo te rele emanatè a. Men m ta konn tande y’ap di emanatè nan tout sa y’ap di ki la yo. M pa t konnen vrèman, m pat konnen ditou èske se li menm ki pou kont èske li la pou l chase marengwen vrèman. Sa vle di mwen menm tou m pa konnen paske m toujou wè yo te mete l limenm lè l’ap dòmi. Men mwen menm m pa konn anyen m pa konnen vrèman.

ITV: Ebyen, oke mesi, se te on plezi.

## Household 1 / member HM22 Female / Round 2

ITV: Bon, manmi se on plezi pou nou ansanm ankò maten an, nou te rankontre sa gen kèk semèn de sela maten an nou fè on ti pase ankò pou n’deranje ou se o sijè de aparèy sa ka ou ap itilize depi on sèten tan, n’ap fè on ti antretyen ki p’ap dire anpil tan, sa kapab dire dis a kenz minit, pou nou jwenn pwendvi ou sou itilizasyon aparèy sa.

HM22: Dakò.

ITV: Manmi, nan ki nivo ou panse aparèy sa pwoteje ou kont moustik k’ap mòde ou lè ou anndan kay la ? Èske li pwoteje ou tou piti ? Byen ? Trè byen ? Pwoteje nèt ?

HM22: Bon mwen men , kòm mwen te di ou , m pa fin konnen si se travay aparèy la, men m te di ou tou m pa tèlman wè marengwen. Sa k fè m di ou mwen menm m pat konn dòmi san kouvri, m te vin dòmi san kouvri men kèk semèn aprè nou te fin bay li , lè m te fin pale avè ou la, m te gen on doum la, sa m te di deja m te jwenn kèk grenn nan doum, tou avan yè la, m tounen jwenn kèk grenn nan yon doum avèk kèk tigrenn konsa, gen dèfwa ou gendwa chita ou santi on bagay, m ka di aparèy la, si se aparèy la m pa konnen non li, gen on travay ki fèt, on gran, gran, gran amelyorasyon.

ITV: Byen, trè byen ? Tou piti ? Kòman nivo pwoteksyon an ye? Si ou t’ap chwazi.

HM22: Ou kapap di trè byen.

ITV: Aparèy sa ke ou ap itilize il y’a quelque temps, èske se tout moun na kay la ki itilize l oubyen on sèl moun?

HM22: Non, mwen kòm m dòmi prèske menm kote avèk pitit sa li menm, li gen youn kote li, kòm se nou de a ki dòmi nan chanm nan. Apresa m gen yon lòt pitit.

ITV: Oke, pwoteksyon sa se pou tout moun ki anndan kay la?

HM22: Gen kèk moun ki di m ke nan chanm pa yo, yo konn wè men mwen menm m ka di m pa tèlman wè sa ditou.

ITV: Lè ou pa anndan kay la, ou deyò kay la, èske ou panse aparèy la pwoteje ou, nan ki nivo li pwoteje ou deyò kay la?

HM22: Bon, pou byen di ou, se on bagay kòm mwen te di ou tou, lontan lè lapli te konn fin tonbe marengwen te konn bay pwoblèm anpil , men m gen kèk tan la m pa wè marnegwen ditou, ditou.

ITV: Deyò kay la ? Ou pa wè yo?

HM22: Deyò kay la , m pa wè yo konsa ankò, m ka wè kòmsi m ta di ou, m te di ou doum nan m ale nan doum nan , m wè y’ap sote nan doum nan, men tankou konsa la, kòmsi m kanpe deyò a, m pa wè sa.

ITV: Ou pa wè marengwen, donk si ou t’ap chwazi byen, trè byen, pwoteje ou nèt se kisa, deyò kay la ki nivo pwoteksyon an ye?

HM22: M te ka di byen.

ITV: E kounya manmi avèk fanmi ou deyò kay la èske ou panse ke aparèy la pwoteje ou avèk fanmi ou?

HM22: Yo tout fanmi an, yo itilize lakou a, m panse se menm bagay la toujou, si m pa wè yo, yo menm yo pa wè yo tou.

ITV: Oke, manmi èske ou ka site pou nou kèk avantaj aparèy la pote pou ou ? Dezavantaj ak avantaj ? Ou ka dekri ? ou ka site pou nou kèk avantaj?

HM22: Se menm bagay la wi, avantaj marengwen an, m pa wè, m pa konn anyen ankò, m pa konn si gen lòt bagay.

ITV: Oke, ou pa wè marengwen.

HM22: Si marengwen p’ap mòde ou, e bagay sa se on bagay ki nwizib anpil, marengwen pa mòde m, li pot on avantaj.

ITV: Oke, li pa pote pou ou enkonvenyan ? Dezavantaj?

HM22: Non, m pa konn kisa li te fè ou, kòm m pa konnen dezavantaj la kisa l ta ye, men avantaj la.

ITV: Dezavantaj pa egzanp si li ta nwi ou nan on sans, si l nwizib pou ou.

HM22: Non.

ITV: Manmi, si n’ap klase enpòtans avantaj yo ke li pote pou ou pa egzanp avantaj fèb, gwo avantaj kisa ou te ka chwai ladan yo? Ti avantaj? Dezavantaj fèb.

HM22: Gwo avantaj.

ITV: Ebyen kounya si n’ap antre nan kisa aparèy la pote pou ou kòm benefis? Ki benefis aparèy la pote pou ou ?

HM22: Toujou marengwen an, ki pa mòde n, ki pa nwi ou, paske lè marengwen ap rele nan zòrey ou pito l te mòde ou, m pa wè sa.

ITV: E si n ta rantre nan nivo enkonvenyan sa l pote pou ou manmi?

HM22: M pa wè non, m pa konn ki enkonvenyan li te ka pote kòm m pa konnen kisa, enkonvenyan kisa li ta ye, m pa wè okenn enkonvenyan.

ITV: Oke, manmi avèk le tan, pa egzanp apre de mwa twa mwa, kat mwa, senk mwa kòman ou wè efikasite aparèy la ? Èske li toujou efikas ? Pa efikas ? ou li vin mwens efikas ?

HM22: M wè l toujou efikas, se toujou menm bagay yo depi, m pa wè marengwen yo, jan yo te konn ye ditou toujou menm bagay la.

ITV: Oke avèk le tan, li toujou bay menm randman ?

HM22: Wi, depi nan kòmansman lè yo te vin poze m kesyon, se toujou menm bagay yo, jis kounyeya, m pa janm wè yo bay pwoblèm.

ITV: Manmi si ou t’ap bay aparèy la on nòt ant zewo a dis, ki nòt ou te ka ba li ?

HM22: M ka di m ta ba li m pa fin di dis la, men m ta’p ba li nèf edmi.

ITV: Poukisa ou retire demi pwen an ?

HM22: Kòm gen de fwa m wè ti marengwen nan dwoum ki fè m di nèf edmi an pou sa sèlman.

ITV: Oke, manmi si ou ta gen dè rekòmandasyon ou t’ap fè pwojè , moun k’ap dirije pwojè a se kisa ? Ki konsèy? Konsènan aparèy la.

HM22: Konsènan aparèy la, pou yo te kapab ranfòse l plis.

ITV: Ranfòse l nan ki sans lè ou di ranfòse l?

HM22: kòmsi, sonje byen m di ou nèf edmi an kòmsi pou l kapab nan dis la, pou l disparèt tout marengwen nèt.

ITV: Non men pou l ka disparèt yo nèt, kisa ou te ka konseye responsab pwojè yo se ou k’ap itilize li?

HM22: Se toujou aparèy la toujou, amwenske m ta di se te on konpay, si yo ta mennen on konpay, kòmsi bagay sante, anplis kòmsi gen kèk kote ki gen marengwen m pa konnen non si se sa oubyen si se aparèy la. Si se aparèy la ou ap pale, m ka di ranfòse l plis, si l manke on ti bagay, ajoute l, pou l ka fè dis sou dis la.

ITV: Oke manmi se te on plezi pou n te ansanm maten an, nou di ou on gran mèsi difèt ke ou konsantre ti tan sa pou nou, ou kite tout aktivite ou sa fè de fwa ke n kesyone ou, nou pran sa trè an konsiderasyon. Nou ba ou garanti ke tout sa ou sot di nou la yo rete konfidansyèl, nou swete oubon kontinuite ak aparèy la. Mèsi bon jounen.

HM22: Pa gen pwoblèm, ebyen nou menm tou mèsi paske travay la ki fèt la li bon pou mwen tou paske marengwen te konn banm pwoblèm anpil, lè y’ap rele nan zòrèy ou se pa on bagay ki dous non, lè ou fon kouri dèyè youn epi gen ladan yo m pa konnen si gen kèk kote k’ap travay, gen de vwa yo ba ou, se pa nenpòt vwa, gen de ti marengwen piti m gen gwo tou, lè y’ap rele nan zòrèy ou se pa bagay jwèt non li ye, yo nwizin anpil anpil, m di ou lè ou wè lapli fin tonbe gade non se on van ki pou pase wi, ou konn ap priye Bondye pou on van pase pou l ka ale avèk yo, tèlman yo te konn ap bay pwoblèm, m ka di kounya la m pa wè sa. Mèsi anpil, m ta renmen pou bagay sa kontinye lòt kote tou jan m benefisye a, gen on lè m te ale nan yon lanmò laplèn se pa marengwen non ki la ankò, se kòmsi kanaval, wi zòn sa se pa zòn ki dous non e yo pale de gonayiv tou, m ta renmen kòmsi si bagay sa te ka elaji, kòmsi pou tout kote sa yo menm jan m benefisye bagay , pou on lòt ta benefisye l tou.M di nou mèsi, m remèsye Bondye dabò,pou l kontinye avèk nou, nou kapap kenbe travay la, pou li kenbe lavi nou.

ITV: Mèsi manmi anpil se te on gran plezi.

OD: Mèsi se madam ?

HM22: HM22.

HM22: Oke bon jounen.

ITV: Parèy.

## Household 1 / member HM13 Male / Round 2

ITV: HM13 se on plezi pou nou ansanm maten an kò on fwa, m panse nou rankontre sa fè de fwa, pou nou kontinye seri antretyen yo, nan kad aparèy ke ou ap itilize depi on sèten tan kont moustik k’ap mòde ou.Nou prezan maten an pou nou fè on ti antretyen ka’p dire ant dis a kenz minit, deja nou di ou mèsi paske ou rann ou disponib pou nou. Nou pral kòmanse poze ou kesyon yo,ou a di nou kisa ou panse?

HM13: Dakò.

ITV:HM13 nan ki nivo ou panse emanatè a pote pou ou on pwoteksyon kont moustik k’ap mòde ou lè ou anndan kay la? Èske li byen pwoteje ou? Trè byen? Pa pwoteje ou? Oubyen pwoteje ou nèt?

HM13: M ka si trè byen pwoteje.

ITV: Kòman ou ka esplike nou sa?

HM13: Avan pwojè a te vin an zòn nan, nou te ka evalye ke pou n te dòmi fò n te genyen on rakèt elektrik yo vann, fòk nou te gen moustikè, pou n dòmi anba moustikè, men kounyeya san moustikè, san rakèt elektrik la nou dòmi konvenableman, alèz.

ITV: Donk, ou ap viv ansanm avèk fanmi ou pa vrè?

HM13: Wi.

ITV: Èske ou panse aparèy sa, tout moun ki anndan kay la pwoteje yo?

HM13: Bon wi, efektivman.

ITV: Nan ki nivo?

HM13: M ka di pou tout fanmi an nèt m evalye l a uit edemi.

ITV: Uit edmi, se byen, trè byen?

HM13: M ka di li trè byen.

ITV: HM13, lè ou pa anndan kay la, ou deyò kay la sou lakou a ou panse aparèy sa pwoteje ou? Oumenk ak fanmi ou?

HM13: Bon, li pwoteje nou men pa a sanpousa e pa menm fanmi an sèlman, menm vwazinaj la tou li pwoteje, paske nou on sèl fanmi, nan vwazinaj la la, nou konn met aparèy la deyò la tou, m kwè li pwoteje ni noumenm, ni vwazinaj la tou.

ITV: Oke donk si n t’ap chwazi ant byen, trè byen kisa n t’ap pran?

HM13: N’ap mete l byen.

ITV: HM13, èske ou ka dekri pou nou kèk avantaj, enkonvenyan aparèy sa pote pou ou?

HM13: Avantaj li pote pou nou, li ede nou konbat anpil maladi, paske marengwen se bèt ki pote anpil maladi, si tout fwa li napeche marenwegn an mòde nou, sa deja se on avantaj li ye pou nou.

ITV: Ki lòt avantaj ankò ke li pote pou ou?

HM13: Pou mwen kesyon an te ye?

ITV: Wi.

HM13: Avantaj ke li pote pou mwen, li rann mwen viv san enkyetid , lè m di san enkyetid la, dèfwa lè m ap suiv de fòmasyon gen de marengwen lè l mode ou, gen tip maladi, si m pa wè mawengwen marengwen k’ap mode m m viv san enkyetid, se on avantaj li ye.

ITV: Oke, donk ou pa gen enkyetid ke marengwen ap mode ou?

HM13: Non.

ITV: Ou ka di nou ki enkonvenyan li pote pou ou, dezavantaj, itilizasyon an?

HM13: Bon, èske gen dezavantaj, m pa kwè dezavantaj non.

ITV:Oke

HM13: Kòm m pa ka poze kesyon, si m te gen otorizasyon gen on kesyon mm t’ap pze, m ka poze li?

ITV: Oke.

HM13: Poukisa yo pa dakò fi , lè m di patisipe nan pwojè a pou yo mete pye l anndan aparèy la?

ITV: Bon, m vrèman pa ka reponn kesyon sa, m pa konn di dòk Obrillant ta gen on eleman de repons.

ITV: Kisa li di? Ki kesyon?

HM13: Yo di m ke yo pa otorize fi tankou lè ou mete aparèy la pou fi mete pye l anndan l aparèy la, jan gason konn fè l yo pa dakò pou fi fè l. M ta renmen poukisa?

ITV: Petèt se pou si fi a ta ansent paske ou konnen menm timoun pa ka touche m panse se poutèt sa.

ITV: Oke ou di nou pa gen enkonvenyan, dezavnataj?

HM13: Non.

ITV: Men ou sot bay uit edmi sou dis donk si li pa dis sou dis kanmenm gen dezavantaj.

HM13: Non sak fè m te bay uit siu dis la, efektivman nou p’ap janm janm ka derasinen vre marengwen an se sak fè m te bay uit sou dis la.

ITV: HM13, si n’ap klase enpòtans avantaj yo ant gwo avantaj, avantaj fèb, ti avantaj kisa ou t’ap chwazi ladan yo?

HM13: Son gwo avantaj li ye.

ITV: Bon HM13, aparèy sa kisa l pote pou ou kò m benefis? kisa ou benefisye de itilizasyon aparèy sa?

HM13: Pwoteksyon kont marengwen.

ITV: Ki enkonvenyan li pote pou ou?

HM13: M’ap toujou di pa gen ankonvenyan.

ITV: Pa gen enkonvenyan?

HM13: Non.

ITV: Bon HM13, avèk le tan kòman ou wè efikasite aparèy la? De mwa, twa mwa apre , kat mwa apre, èske li toujou bay menm randman, di nou kòman ou ka esplike nou?

HM13: M pa ka di li bay menm randman, dayè, lè aparèy la te fèk vini an vre m pat byen , byen, byen enbi de li , sa vle di na tan pa m m vin konprann li an, kòmsi m wè efikasite a elve , kò m pa konnen kesyon an efikas la èske..

ITV: Marengwen yo toujou la? Èske pa gen marengwen ditou? De mwa apre, twa mwa apre ,kèlke mwa apre?

HM13: Bon, ou ap toujou jwenn men a volim ou te konn jwen li an, ou pa jwenn li.

ITV: Oke.

HM13: Sa vle di, li vin diminye.

ITV: Bon, HM13, si ou t’ap bay aparèy la on nòt ant zewo a dis , ki nòt ou t’ap ba li?

HM13: M t’ap ba li nèf, poukisa m t’ap ba li nèf la, paske jan m te di a , nou p’ap janm ka fin derasinen tout outan gen bagay nan nou menm nou pa kòmanse retire , paske m toujou di sa, ma dlo se bagay ki kale marengwen si tout fwa nou pa ka nou menm antanke lòm nou pa ka retire sa,se pa aparèy la ki pral ede nou derasinen marengwen.

ITV: Oke, HM13 si ou ta gen on konsèy ke ou t’ap bay moun k’ap dirije pwojè yo kisa li t’ap ye? Ki rekòmandasyon? Ki konsèy?

HM13: Bon, m te bay konsèy sa, m’ap rebay li ankò,m ta renmen lè n’ap tounne a pwojè sa ankò, m ta renmen plis lòt kay benefisye l. Kòmsi m wè kantite moun, kantite mezon , fanmi k’ap benefisye la on ti jan minim si tout fwa nou ta ka touche plis fanmi lè sa travay la t’ap pi bon.

ITV: Oke HM13 nou di ou mèsi anpil donk se te on plezi pou n te ansanm, m’ di ou mèsi difèt ke ou konsakre titan sa pou nou maten ou kite aktivite ou pou nou , ou disponib pou nou, donk nou trè kontan, nou swete ou bòn joune, nou espere on jou ankò n’ap fè on ti pale o sijè de aparè la, sa ap pèmèt nou evalye l pi byen.

HM13: Se mwen ki pou ta remèsye nou pou gwo travay sa n’ap fè nan zòn na, m panse ke Bondye ap toujou avè nou nan tout sa n’ap fè. Jan m di a kò m konsèy la, m espere ke konsèy m ban ou an, nou pral chita sou li, pou n ka touche lòt fanmi.

ITV: Dakò, mèsi bokou HM13.

## Household 1 / member HM14 Male / Round 2

ITV: HM14 se on plezi pou nou ansanm maten ankò m kwè nou rankontre sa fè de fwa?

HM14: Wi, wi.

ITV: Nou fè on ti pale deja, nou retounen ankò avèk seri antretyen yo, pou nou wè si gen chanjman, evolisyon o sijè aparèy ke ou ap itilize depi on sèten tan kont moustik k’ap mòde ou. Nou pral fè on ti antretyen ka’p dire anviwon dis a kenz minit, dakò?

HM14: Dakò.

ITV: Premyè keson n’ap poze ou HM14, aparèy sa ke ou ap itilize depi kèk tan, èske ou panse li pwoteje ou kont moustik anndan kay la?

HM14: Anpil, anpil.

ITV: Nan ki nivo, li pwoteje ou?

HM14: Paske pou byen di ou oparavan, pou byen di ou te gen anpil marengwen, men depi lè, emanatè nan kay la se kòmsi m pa tèlman wè sa yo rele marengwen an menm , si ou wè youn, ou pa’p wè de, emanatè a ede nou anpil nan kay la, e se pa sèl kay la, li ede lòt moun nan lokalite a la tou, nan menm lakou an paske gen de lòt kay ankò, paske nòmalman m te sipoze fè sa, se on bagay m te sipoze fè, m gen de emanatè la, m te sipoze , kòm frè m nan gen tibebe la m te sipoze ba l youn met kote l la men, kòm li la, timoun nan pot ko fèt lè emanatè a te la, sak fè m pa bay li, se paske efikasite a vin on tijan diminye , se sak fè m pa ba li, sa vle di, si se on bagay ki, yo pase pwodui ladan si yo p’ap pran li m ta sipoze bay li pou l mete pou pwoteje timoun nan.

ITV: HM14 si n t’ap chwazi ant byen, trè byen, kisa ou t’ap chwazi ladan yo?

HM14: M t’ap chwazi trè byen, si ta gen trè, trè, trè byen m panse se li m t’ap chwazi.

ITV: Gen pwoteje nèt tou kisa ou t’ap chwazi?

HM14: Ou mèt banm nèt la,

ITV: Bon HM14, kesyon nou sot poze ou a la, li te pèsonèl, mentnan n’ap gade oumenm avèk fanmi ou, nan ki nivo aparèy sa pwoteje nou kont moustik k’ap mòde nou anndan kay la?

HM14: Bon, pou byen di ou li pwoteje nou tout nèt, m byen di ou se pa sèlman kay la, li pwoteje tout moun ki nan lokalite an la,pou byen di ou chak lè m pral dòmi m pran l m mete l bò kote m nan, menmsi se atè m dòmi , m met on matla atè a, lè m kouche sou kabann li banm senti fè mal, manman m se bò kote m nan kouche m jis mete youn nan mitan ou an avèk on gran frè m ki la , m kite youn bò kote l la , si se pou marengwen an nou pa jwenn sa yo rele marengwen an menm , m di ou di ou wè youn ou p’ap wè de, m pa konn depi kilè, m wè marengwen nan kay la.

ITV: Oke, si n ta tounen ankò ant byen, trè byen, pwoteje nèt kisa ou t’ap chwazi?

HM14: Pwoteje nèt.

ITV: HM14 kounya nou kite anndan kay la, n’ap pran deyò kay la, lè ou deyò kay la èske ou panse aparèy sa pwoteje ou? Oumenm ak tout fanmi ou?

HM14: Wi li pwoteje paske m konn fè on nonb de tan na la kou a si l bon, tankou nou gen on klèb dèyè a la gen dèfwa se pou emanatè a pat la, ou chita dèyè epi marengwen ap siyone zòrèy ou, ap fè bri, ap mòde ou, men kounya la, m pa wè sa. E klèb la se on kote ki konn chaje marengwen, men kounya m pa wè sa ankò.

ITV: Oke, donk ant byen, trè byen, kisa ou t’ap chwazi? Pwoteje nèt?

HM14: Trè byen, ou met banm trè byen.

ITV: HM14, èske ou ka site pou nou kèk avantaj, enkonvenyan aparèy sa pote pou ou?

HM14: Bon, pou avantaj m pa wè sa m te ka di, pou avantaj la te gen epòk te gen anpil marengwen pou kounya la m pa tèlman wè bagay konsa, m pa ka di gen dezavantaj. Tout sa m ka di se avantaj ki genyen, paske m pa wè dezavantaj.

ITV: Ki lòt avantaj ankò li pote pou?

HM14: Li pwoteje tout moun na kay la. Lè fini li pwoteje tout moun ki nan menm lakou a.On gwo bagay li ye pou mwen,

ITV:HM14, si n’ap klase enpòtans avantaj yo egzanp gwo avantaj, avantaj fèb, ti avantaj kisa ou t’ap chwazi ladan yo?

HM14: Gwo a.

ITV: HM14, èske ou panse aparèy sa pote pou ou kèk benefis, si wi kisa yo ye?

HM14: Wi, yo pote benefis, m pa jwenn marengwen mòde m, yo pa mòde fanmi m, on pakèt benefis li pote pou mwen depi li pa mòde, pa mòde fanmi m pou pote maladi nan kò nou, on gwo avantaj liye, on gwo benefis li ye ou pa dakò?

ITV: Wi. Bon HM14 yo souvan di depi gen avantaj, gen enkonvenyan, ki enkonvenyan aparèy sa pote pou ou?

HM14: M pa ret kwè sa, m pa ret kwè gen enkonvenyan, pa gen sa menm.

ITV: Bon HM14, avèk le tan kòman ou wè efikasite aparèy la? De mwa, twa mwa apre, kat mwa apre, èske li toujou bay menm randman?

HM14: Pou byen di ou kesyon sa, yo pa poze m li m te ka reponn kesyon sa tou, paske lè yon bagay fèk aspèje a fò n ta met nan tèt nou apre on nonb de tan li patap toujou menm jan, oke, pou byen di ou lè li te fèk vini li p’ap menm bagay avèk pou kounya la, sa vle di de tanzantan bagay la se diminye l’ap diminye la, sa vle di se on bagay ki pou ta aspèje, m pa konn sou konyen tan yo konn fè sa men m pa konnen m pa ka di pou n ta aspèje l pou mwenm, m pa konn si n’ap kite l pou mwen nèt ,men pou byen di ou li p’ap janm menm jan.

ITV , HM14, si ou t’ap bay aparèy la on nòt ant zewo a dis, ki nòt ou t’ap ba li?

HM14: O dis.

ITV: Poukisa ou t’ap ba l dis?

HM14: Paske li pwoteje m, se pa sèl mwen menm li pwoteje, li pwoteje tout moun ki nan kay la, se sak fè m t’ap ba l dis lan, bon si te gen plis ke dis m t’ap ba li plis ke dis.

ITV: Oke, HM14 si ou ta gen on konsèy ke ou t’ap bay moun k’ap dirije pwojè yo kisa li t’ap ye? Ki rekòmandasyon? Ki konsèy?

HM14: O sitou, pou doktè Chicoye m sonje m te di Milò sa, se pa sèl doktè a non, m te di m te gen on kado pou m te fè yo, an palan de twa moun m konnen ki te nna pwojè a de sipèvizè yo avèk doktè Chicoye paske mwen menm si pou m byen di ou, se on komèsan m ye, pafen m konn vann , m te di menm lè se on pafen m’ap achte pou m fè doktè Chikoye kado kanmenm avèk se lòt moun yo, sa vle di, pou tèlman travay la bon , li pwoteje nou nan zòn nan. E anplis tou m sonje m te di Milo, m pa konn si Milo te di doktè a sa, paske premye fwa lè yo vin la, apresa lè m jwenn Milo, m di Milo pwochèn fwa k’ap genyen, èske yo p’ap ka pran lòt kay, Milo di m non ki ale, se li blan konnen, se li k’ap toujou travay , m te di Milo menm lè se non nou ki ale, depi yo tounen ankò, m’ap bay non de two moun nan plas mwen an. E mwen fè l, ou wè kòb doktè a ban mwen la, m pral separe l a kat moun , mwen menm k’ap fè kat, m ta bay de lòt moun travay , nan pa m nan.

ITV: 3?

HM14: Twa.

ITV: Oke HM14, se byen se te on plezi pou n te ansanm maten an, ankò, on fwa nou di ou mèsi difèt ke ou konsakre titan sa pou nou , nou sou dezyèm antretyen avèk ou donk se on gran plezi, nou swete ou bòn kontunuite avèk aparèy la. Bòn joune.

HM14: Mèsi, se mwen ki pou ta di nou mèsi tou paske pou bagay sa k’ap fèt la se on bon bagay li ye pou zòn nan e m ta swete pou lè pwojè a tounen nan zòn an ankò, menm lè se non m ki ale, se non m yo konnen , menm lè n ta pran lòt moun travay nan plas mwen, li p’ap deranje m. okontrè si li epapiye li ap bon mwen toujou, l ap pi bon pou zòn nan tou.

ITV: Mèsi anpil HM14, se te on plezi.

HM14: De ryen.

## Household 2 / member HM15 Female / Round 2

ITV: Nan ki nivo ou panse aparèy la pwoteje pou ou on pwoteksyon kont moustik k’ap mòde ou anndan kay la? Nan ki nivo ou panse aparèy la pwoteje pou ou on pwoteksyon kont moustik anndan kay la?

HM15: Bon, kòm m te ka di?

ITV: Èske li pwoteje ou? Trè byen? Pa pwoteje ou? Pwoteje ou totalman?

HM15: Li pwoteje nou.

ITV: Oke.

HM15: Li pwoteje fanmi an alò, emanatè a li enpòtan,a travè de aparèy la,li ede, li fè nou wè, alò pa gen moustik, kòmsi jan moustik yo te ye a li pa konsa nan mezon an.

ITV: Yo diminye. Men si n t’ap chwazi nan ki nivo li pwoteje ou , byen, trè byen, oubyen pwoteje ou nèt nan ki nivo? Kisa ou te ka chwazi?

HM15: M’ap mete byen.

ITV: HM15 nan ki nivo ou panse aparèy sa pwoteje ou avèk fanmi ou kont moustik anndan kay la?

HM15: Padon?

ITV: Nan ki nivo ou panse li pote pou ou on pwoteksyon ou avèk fanmi ou kont moustik anndan kay la? M panse se pa sèl oumen ki itilize l’ se tout moun anndan kay la pa vrè?

HM15: Wi.

ITV: Nan ki nivo ou panse li pwoteje nou?

HM15: Bon, li pwoteje nou. Alò li pèmèt marengwen pa mòde nou sa se on prevansyon, li ede lòt moun kòmsi, alò si n pa itilize moustikè, alò nou kapab dòmi byen, m iwouve aparèy la trè enpòtan.

ITV: Oke, si n ta chwazi bye, trè byen, totalman kisa ou t’ap pran ladan yo?

HM15: Byen.

ITV: Toujou byen, oke, bon HM15 kounya nou kite anndan kay la nou tonbe deyò kay sou galri, èske ou panse lè ou deyò kay la aparèy sa pwoteje ou? Lè ou sou galri a oubyen nan lakou a? Li pwoteje ou?

HM15: Li pa pwoteje m deyò , aparèy la se senpleman pou andedan, alò deyò m toujou remake.

ITV: Ou pa konn itilize l deyò a?

HM15: Non.

ITV: Lè ou deyò li pa pwoteje ou paske ou pa itilize l tou?

HM15: Paske nou pa itilize l deyò , il suffit que m itilize l deyò a menm jan gen on prevansyon ki fèt pou anndan , gen on prevansyon ki ka fèt deyò.

ITV:Bon HM15, deyò kay la avèk fanmi ou, nan ki nivo ou pase li pwoteje ou? Avan se te oumenm poukont ou men kounya avèk fanmi ou nna ki nivo ou panse li pwoteje ou aparèy la, lè ou deyò kay la?

HM15: Men mwen reponn ou kesyon sa paske m te di ou nou pa itlize l deyò.

ITV: Oke, HM15, èske ou ka di nou oubyen dekri nou kèk avantaj aparèy sa pote pou ou? Avantaj, dezavantaj? Ou ka site kèk pou nou.

HM15: Ki avantaj aparèy la pote pou ou? Kisa l pote pou ou kòm pwofi?

ITV: Kisa li fè pou ou?

HM15: Ebyen li fè pou mwen, li pèmèt ke marengwen pa mòde m.

ITV: Ki lòt avantaj li pote pou ou ankò?

HM15: Li fè m dòmi byen.

ITV: HM15 ki enkonvenyan li pote pou ou? Ki dezavantaj?

HM15: Bon, m pa swiv dezavantaj non doktè.

ITV: Ou pa gen dezavantaj?

ITV: Ki pwoblèm li bay nan kay la?

ITV: Li pa bay okenn pwoblèm?

HM15: Non. M pa wè li bay kenn pwoblèm.

ITV: Oke, bon HM15 kounya si n te k’ap klase avantaj yo kòm ou si li pa bay enkonvenyan, si n ta ka klase avantaj yo an tèm enpòtans yo, kòman n te ka klase yo? Fèb? Trè fèb oubyen gwo avantaj li pote pou ou?

HM15: Kòman?

ITV: Avantaj ke l pote pou o yo, èske yo fèb? Èske yo trè fèb ou dimwens li pote pou ou gwo avantaj?

HM15: Li pote gwo avantaj.

ITV: Èske ou ka site ak dekri pou ou kèk, pa nan nivo avantaj , nan nivo pito benefis aparèy sa pote pou ou, kisa ou jwenn ladan l kòm benefis? Ki benefis li pote pou ou? Kisa ou benefisye nan itilizasyon aparèy la?

HM15: M pa sezi kesyon an.

ITV: Ki benefis itilizasyon aparèy la pote pou ou?

HM15: Kòman ki benefis?

ITV: Kisa li fè pou ou , ke ou jwenn ladan depi li rive nan kay la?

ITV: Li ka ekonomik, sosyal, kisa ou benefisye?

ITV: Bon, pa gen repons ou pa konprann?

ITV: Pas de réponses. Bon avantaj yo HM15 te di se enpòtan. Si ou t’ap bay aparèy la on nòt ant zewo a dis ki nòt ou t’ap ba li?

HM15: Ant zewo a dis, m t’ap ba l dis.

ITV: Poukisa ou t’ap ba l dis HM15?

HM15: Premyèman li pwoteje fanmi an kont marengwen.

ITV: Dezyèmman, si gen on premyèman.

HM15: M pa kwè gen gen on dezyèm non.

ITV: Oke, HM15 avèk le tan kijan ou wè efikasite aparèy la? Èske li vin plis efikas, mwens efikas , pa ditou efikas avèk tan?

HM15: Kòman avèk le tan?

ITV: Aprè de mwa, twa mwa , kat mwa kòman ou wè efikasite a?

HM15: Li bon.

ITV: Wi, men, èske li vin mwens efikas, plis efikas?

ITV: Èske li te pi bon avan ke kounya?

HM15: Kòman pi bon avan?

ITV: Lè l te fèk vini nan kay la èske l te repouse moustik pi fasil?

HM15: Bon, pi fasil.

ITV: Avan li te pi fasil?

HM15: Bon wi.

ITV: Oke, avèk le tan sa diminye?

HM15: Wi.

ITV: Bon HM15 si ou ta gen on konsèy ou t’ap bay moun ki responsable pwojè yo, gwo zotobre yo sou aparèy la ki konsèy ou te ka ba yo? Sou fonksyonnman aparèy la.

HM15: Alò, konsèy ke m te ka bay, nou pa vle on sèl aparèy, puiske gen dè kesyon ki poze sou deyò a m panse si gen de aparèy li ap trè enpòtan, sa se konsèy mwen.

ITV: De emanatè?

HM15: Wi paske li ap bon, alò si mwen m gen difikilite pou m reponn kesyon deyò a, m panse on lòt tou kagen difikilite tou pou reponn.

ITV: Ou pa gen lòt konsèy ankò, lòt rekòmandasyon?

ITV: Oke mèsi.

## Household 2 / member HM19 Male / Round 2

ITV: HM19, se on plezi pou nou ansanm maten an donk, pou nou fè ti koze o sijè de aparèy ke ou ap itilize depi kèk tan, nou gen on seri de entretyen ki te fèt deja, ou retounen ankò pou nou gade kòman sa evolye se sak fè nou prezan maten an. Ou konnen m deja oukonn doktè a, ou konn pwojè a plizoumwen byen dokn sa pa vo lapèn pou pran titan pou prezante ou pwojè a donk n’ap vini direkteman ak seri de kesyon yo. Nan ki nivo ou panse emanatè a pwoteje ou kont moustik k’ap mòde ou lè ou anndan kay la ?

HM19: Li byen pwoteje m, li pwoteje m trè byen, sèl sa toutan li gen medikaman yo ladan l’ap pi bon toujou paske toutan li dire plis fò l ta toujou gen bon jan de medikaman pou l ka pi efikas, men si se pou pwoteje, li pwoteje trè byen, paske m swiv sa menm akoz de aparèy la, li bon anpil. Gen mwens moustik bagay sa yo.

ITV: Oke men èske ou ap viv an koup ? famiy?

HM19: Wi m gen on nyès mwen m regrèt li pa la, se bò kiskeya l’ap vann, aprè midi lè l vini li toujou vin bonè annik benyen al lekòl ankò.

ITV: Pwoteksyon an se pou ou ak pou li tou? Li pwoteje l tou ?

HM19: Wi, wi.

ITV:Nan ki nivo ou panse aparèy la pote on pwoteksyon kont moustik andeyò kay la, sou lakou a?

HM19: Wi, li pwoteje m men m’ap gade pou anviwonnman li pwoteje paske moustik yo lè m kenbe yo, paske se vole l’ap vole li ka ale nenpòt kote men pa rapò avèk aparèy la, n’ap itilize a kont moustik la, m wè li efikas li bon anpil menm pou anviwonnman tou li bon anpil, paske lè n pran moustik yo se travay yo di pou n fè a, se pou moustik yo paske nou toujou pran moustik yo e moustik yo k’ap bay plis pwoblèm nou kenbe yo, m wè, bon pou mwen menm se on bon travay li ye.

ITV:Li pwoteje ou byen ? Trè byen ? Pwoteje nèt ?

HM19: Byen, byen, trè byen.

ITV: Oke, li pwoteje ou tou kont moustik k’ap mòde ou ak moun k’ap vivi lakay ou deyò kay la ?

HM19: Wi.

ITV: An famiy li pwoteje ou ?

HM19: M kapab di se pa mwen sèlman, li pwoteje nou, paske se an fami n’ap viv bagay sa yo , li pwoteje nou, li bon.

ITV: Oke nan ki nivo pwoteksyon sa? Piti, byen , trè byen ?

HM19: Trè, trè byen.

ITV: HM19, èske ou ka site oubyen dekri kèk avantaj avantaj aparèy sa pote pou ou ? Avantaj ? Dezavantaj ?

HM19: Non, m pa ka esplike anpil, anpil avantaj li pote.

ITV: Ou ka site kèk pou nou ? si ou di li pot anpil.

HM19: Pa egzanp, moustik yo, nou te gen on jan anpil vin gen aparèy la kounyala gen mwens moustik se sak fè m di li anpil m pa ka menm site menm, m ka site sa yo.

ITV: Ki lòt ?

HM19: Bon li pote anpil bagay, anpi anpil li bon pou ou.

ITV: WI, anpil, anpil men èske nou ka kantifye yo? Pou site youn, de, twa kòm avantaj ke l pote pou ou, moustik yo diminye apresa kisa l pote pou ou ankò ?

HM19: Wi m byen di wi, se sak fè m di yo anpil la wi.

ITV: Èske aparèy sa poye pou ou enkonvenyan ? Si wi ki enkonvenyan.

HM19:Non.

ITV: Li pa pote kenn enkonvenyan ?

HM19: Non li byen pou mwen, byen.

ITV: Oke, HM19 , si n’ap klase enpòtans avantaj ke li pote pou ou yo , nan ki nivo nou ka klase yo ? Avantaj fèb, ti avantaj ? Gwo avantaj ? Dezavantaj fèb ? Gwo dezavantaj.

HM19: Pou mwen menm, Gwo avantaj li pote.

ITV: Oke , gwo avantaj li pote ?

HM19: Wi, gwo avantaj.

ITV: HM19 ki, nan itilizasyon aparèy sa kisa ou jwenn kòm benefis,benefis ke l pote pou ou ?

HM19: M kapab di kòm m te di l deja se pa pou di ou aparèy la sèlman, kòman m kapab di pwojè, m pa konnen kòman m ta di l se pou sa l pote anpil avantaj li pa pote dezavantaj pou mwen non.

ITV: Oke, ou ka site pou nou kèk benefis ke l pote pou ou ke pwojè sa oubyen aparèy sa pote pou nou?

HM19: Si se pou pwojè sa oubyen aparèy la, yo pote, li pote anpil bagay pa egzanp menm pou fanmi, pou nou , pou zanmi pou anviwonnman tou li pote anpil bagay.

ITV: Wi anpil bagay, se kisa konsa ?

HM19: Dòk la m wè m pa ka site non, gen twòp bagay li pote.

ITV: Bon, HM19, si ou t’ap bay aparèy la on nòt ant zewo a dis, ki nòt ou te ka ba li ?

HM19: M patap bay zewo ditou, m t’ap ba l dis.

ITV: Poukisa ou t’ap ba li dis ?

HM19: Se meyè li travay byen nòmalman m ba k dis, si m pat jwenn anyen m t’ap ba l omwen zewo, li byen travay fò m ba l dis.

ITV: Oke, trè byen, HM19 si ou te gen on rekòmandasyon ou t’ap fè pwojè, moun k’ap dirije pwojè a se kisa ? Ki konsèy ? O sijè de aparèy la menm kisa ou te ka di yo.

HM19: Sèl sa m te ka di pwojè a se on bon pwojè ta ka pi aleji li t’ap pi bon, tout anviwonnman men se pou aparèy la si l pa gen medikaman kòm si m ta di ou, paske fò l gen medikaman, si l fè tout tan yo epi l pa jwenn anyen ladan se sèl sa pou pwojè sa se on bon bagay li ye, m ta renmen nòmalman pou l plis laj pou l ta kouvri nan lòt peyi yo tou, tout zòn yo ou pa konprann, pou yo elaji plis.

ITV: Oke, HM19, se te on plezi pou n te ansanm maten an, nou di ou on gran mèsi difèt ke ou te disponib pou nou, nou vin deranje ou. Nou garanti ou ke tout sa ou sot di nou la yo rete konfidansyèl p’ap gen moun ki kapab di nou se HM19 ki te di nou tèl bagay e ke nou swete oubon kontinuite ak aparèy lan, nou swete nou rekwaze ankò.

HM19: Non, nou pa deranje m. Mèsi se on plezi pou men m byen kontan, bon jounen.

ITV: Mèsi, parèy.

## Household 3 / member HM17 Female / Round 2

ITV: Ebyen HM17, se on plezi pou nou ansanm maten an donk, pou nou fè ti koze o sijè de on aparèy ke ou ap itilize depi on sèten tan, emanatè a.Nou pase la maten an pou nou jwenn pwendvi pa ou, pou nou wè kòman, ki randman, ki efikasite aparèy sa anfèt kisa li pote pou ou. Nan ki nivo ou panse emanatè a pwoteje ou kont moustik k’ap mòde ou lè ou anndan kay la ?

HM17: Bon, li pwoteje nou kont on pakèt maladi, paske lè moustik la mòde ou, li ka ba ou on pakèt maladi, ba ou malarya, li ka ba ou tandòt maladi, avèk aparèy la nou santi nou pwoteje.

ITV: Oke, men nan ki nivo? Li tou piti? Li byen pwoteje ou? Li trè byen pwoteje oubyen pwoteje ou nèt ?

HM17: Trè byen.

ITV: Li trè byen pwoteje ou. HM17 nan ki nivo ou panse li pwoteje ou kont marengwen yo lè ou deyò kay la ? Li byen pwoteje ou? Li trè byen pwoteje oubyen pwoteje ou nèt ?

HM17: M pa konn itilize l deyò. M plis itilize l anndan.Paske m pa fasil deyò mwen menm

ITV: Oke, ou ap viv an koup ? An famiy?

HM17: An koup.

ITV: Èske ou panse ke ak mari ou sa pote on gwo proteksyon pou ou aparèy la ?

HM17: Wi.

ITV: E kòman ou ka esplike nou ?

HM17: Li pwoteje nou toujou kont maladi yo toujou , paske depi moustik la mòde ou li transmèt on pakèt maladi nan kò ou.

ITV: Dakò, nivo de proteksyon an se byen , trè byen ?

HM17: Byen.

ITV: Oke, bon HM17, èske ou ka site pou nou kèk avantaj emanatè a pote pou ou ? ou ka dekri kèk avantaj ? Ou ka site kèk avantaj ?

HM17: Nan avantaj dèfwa lè moustik yo egzajere nan kò ou , ou konn pral achte blakatòks , ou konn flite tou , kounya nou vin sanse pa achte yo, noun plis itilize aparèy la.

ITV: Oke, ou ka site kèk enkonvenyan ke li pote, aparèy la, itilizasyon, dezavantaj?

HM17: M pa wè non, m’ pa jwenn okenn dezavantaj non.

ITV: Ou pa jwenn okenn dezavantaj ladan. Oke, HM17 si nou ta klase enpòtans avantaj aparèy la bay, li pote gwo avantaj ? Ti avantaj ?Dezavantaj ? Kòman ou te ka klase sa ?

HM17: On gwo avantaj lè ou ta pral achte bagay e kòman, blakatòks la , kanmenm kò sa ou kite l, ou a fè on lòt bagay avè l. Pase chak jou pou ap achte blakatòks, kòb la ap rete fè on lòt bagay.

ITV: Oke, èske ou ka site pou nou kèk avantaj, kèk benefis aparèy a pote pou ou ? Lòt benefis.

HM17: Bon, se menm kesyon an ankò wi.

OD: HM17 ap swiv wi.

ITV: Atantivman, wi HM17 ki lòt.

HM17: Se menm bagay yo wi, menm benefis yo, kòb la rete ou fè on lòt bagay avè l.

ITV: Kòb la rete oke, HM17 ou di nou pa pote kenn ekonvenyan, dezavantaj ?

HM17: Non.

ITV: HM17, si ou t’ap bay emanatè a on nòt ant zewo a dis, ki nòt ou te ka bay?

HM17: M t’ap ba l nèf.

ITV: Poukisa ou t’ap ba l nèf ?

HM17: Lè ou lekol yo toujou di ou pa bay dis, dis sou dis, m bay nèf la.

ITV: Si se nèf la ou bay, gen on rezon kanmenm.

HM17: M pa gen rezon m jis bay nèf la.

ITV: Oke, ou chwazi bay nèf la, bon HM17 si ou te gen on rekòmandasyon ou t’ap fè pwojè , moun k’ap dirije pwojè a se kisa ? Ki konsèy ? Aparèy ke ou ap itilize depi kèk tan.

HM17: M ta renmen pou pwojè an, avanse pi devan paske ou konnen sitou nan sosyete nou an dèfwa moun nan konn gen volonte vrèman pou l ta rive lwen ak sa ke l’ap fè a men li pa vrèman gen posibilite, li pa fasil jwen yo, lepli souvan .

ITV: Oke , ben HM17, se te on plezi pou n te ansanm maten an, nou di ou on gran mèsi difèt ke ou te disponib pou nou, ou te konsakre titan sa pou ou. Nou rasire ou ke tout sa ou sot di nou la yo rete konfidansyèl p’ap gen moun ki kapab di nou se HM17 ki te di nou tèl bagay e ke nou swete ou on trè bòn jounen.

HM17: Dakò.

ITV: Mèsi byen.

# IDI Block 3

## Household 1 / member HM26 Male / Round 2

ITV: Ou ka kòmanse wi

ITV: Dakò kounye a nou nan fwaye 1 blòk 3 kay HM26, HM26 wap reponn kesyon sa yo pou nou si’l vou plè. Nan ki nivo… kesyon 1 an nan ki nivo ou panse emanatè a pwoteje’w kont marengwen kap mòde’w lè’w nan kay la wap fè yon ti wonn nan repons ki pi ba yo oswa sèke nòt ki anba a?

HM26: E byen ou ka…..

ITV: Li pa pwoteje’w?

HM26: Li pwoteje’m, li pwoteje’m.

ITV: Li pwoteje’w tou piti, byen ou byen trè byen pwoteje nèt w’ap chwazi.

HM26: Byen, li pwoteje byen.

ITV: Li pwoteje’w byen dakò ok, kesyon 2 nan ki nivo pou emanatè a pwoteje’w ak lòt moun nan kay la… ak lòt moun lakay ou kont lòt vèmin yo lè yo nan kay la, wap fè yon ti wonn nan youn nan repons ki pi ba yo oswa sèke nòt ki anba la oswa pa gen repons èske li pa pwoteje’w li pwoteje’w tou piti?

HM26: Tou piti.

ITV: Byen, trè byen.

HM26: Tou piti.

ITV: Donk tou piti ok, kesyon 3 nan ki nivo emanatè a pwoteje’w kont marengwen lè ou deyò nan lakou a wap fè yon ti wonn nan repons ki pi ba yo, oswa sèke nòt ki anba la donk repons yo se li pa pwoteje’w , tou piti , li pwoteje’w byen , li pwoteje’w tre byen , li pwoteje’w nèt kisa w’ap chwazi?

HM26: Bon li pa tèlman pwoteje’w …….. li pa tèlman pwoteje’w

ITV: Li pa tèlman pwoteje’w donk li pwoteje’w……..

ITV: Donk ou ka ekri’l pou li li pa tèlman pwoteje

ITV: Ok kounya nou prale nan kesyon 4 la, nan ki nivo emanatè a pwoteje’w ak fanmi’w kont lòt vèmin yo lè ou deyò kay la, wap fè yon ti wonn nan youn nan repons ki pi ba yo oswa sèke nòt ki anba la donk se li pa pwoteje’w tou piti , byen , trè byen pwoteje nèt?

HM26: Tou piti.

ITV: Tou piti dakò 5 site ak dekri nenpòt lòt benefis oswa dezavantaj ke emanatè a bay

HM26: Bon tankou lè’m mete’l nan chan’m lan m’wè li li, marengwen yo gen mwens marengwen.

ITV: Lew mete emanate an nan chanm ou ou li gen mwens marengwen

HM26: Li gen mwens marengwen.

ITV: Li kap ekri ou byen?

ITV: Ou pa oblije ek…. ou ka ekri wi men ou pa oblije.

ITV: Ok dakò donk se sèl benefis sa li ba ou.

ITV: e pou dezavantaj?

HM26: Bon de par dezavantaj sèke lè pye’m pran ladan’l li fè’m wè li kon eseye blese’m

ITV: li kon blese’w pafwa?

HM26: Wi.

ITV: Lè’w mal kenbe’l?

HM26: Wi lè’m mal kenbe’l li konn eseye li konn blese pye’m …

ITV: Eben ban’m wè pye’w ( rire ).

ITV: Ok dakò kesyon 6, klase enpòtans avantaj, dezavantaj sa pa younn nan repons ki anba yo oswa nenpòt nan bagay sa yo ou ka pa aplike oswa pa gen repons. Donk men repons yo: gwo dezavantaj oswa pabon menm, ti dezavantaj pa trò mal, dezavantaj fèb, avantaj fèb, ti avantaj, gwo avantaj.

HM26: Avantaj fèb

ITV: Avantaj fèb dakò.

ITV: Avantaj fèb se kounya ou byen avan?

HM26: Bon avan’l avan’l avan’l te chak tan li vin pwoteje’m ……bon se pa marengwen ki vin abitye avè’l men avan li te li te pi efikas.

ITV: Avan pat gen dezavantaj?

HM26: Wi.

ITV: Avan te gen gwo avantaj?

HM26; Wi te gen gwo avantaj vrèman.

ITV: Ekri gwo avantaj avan.

ITV: Gwo avantaj avan

ITV: Oswa gwo avantaj, avan vwala e ti dezavantaj se kounya

HM26: Wi.

ITV: Ti dezavantaj kounye a …ti dezavantaj sa se avantaj donk deza….ti dezavantaj se la , isi.

ITV: O ti avantaj

ITV: o siprime sa

ITV: Ti dezavantaj

ITV: Wè la.

ITV: M’mete.

ITV: Ti dezavantaj se avan , avan.

ITV: O u di gwo avantaj se avan.

ITV: An gwo avantaj e avan ti dezavantaj se ( maintenant)

ITV: Ok donk nou nan kesyon 7 la kounya site ak dekri nenpòt lòt benefis oswa dezavantaj ke emanatè a bay donk ki lòt benefis emanatè ba ou oswa ki lòt dezavantaj ke ou jwenn?

HM26: Bon sèke …ni po tèt men’m lè ou mete’l nan chanm lan ou chita ou mete’l devan chèz la marengwen an pa vin sou ou se avantaj sa.

ITV: Ok entèresan dakò e kesyon 8 lan kounye a , klase enpòtans avantaj , dezavantaj sa pa youn nan repons ki anba yo, oswa nenpòt nan bagay sa yo, donk e ka pa aplike oswa pa gen repons donk men repons ki anba yo se: gwo dezavantaj pa bon menm , ti dezavantaj pa twò mal, dezavantaj fèb, avantaj fèb, ti avantaj, gwo avantaj?

ITV: toujou presize’l avan ou byen kounya.

HM26: Avan te avantaj la ton jan t gwo avantaj avan kounya o fi e a mezi gon ti retrans yo vin met ladan’l marengwen yo vinn plis.

ITV: sa vle di li vin fèb kounya?

HM26: Li vin feb

ITV: E pou dezavantaj te gen gwo dezavantaj avan ou byen ti dezavantaj avan

HM26: Li pa ban’m li pa le li pa ban’m dezavantaj e sèl sof sa m’blese ladan’l

ITV: Li te blese’w nan mèt la

HM26: Li te blese’m lè m’ap pran’l

ITV: Ok donk nou pral nan kesyon 9 la kounye a , site ak dekri nenpòt anm… lòt benefis ke emanatè a bay?

ITV: Oswa dezavantaj benefis ou byen dezavantaj?

ITV: Oswa dezavantaj ke emantè a bay?

HM26: Li pa bay dezavantaj avantaj , li pa bay dezavantaj se se sof ke lè’l la li diminye marengwen yo marengwen yo te vin mwens.

ITV: Hum ok

HM26: Li redwi li redwi pousantaj marengwen yo avan te konn gen plis marengwen kounya la gen mwens.

ITV: Son bon avantaj dakò e kesyon 10 la kounya , se klase enpòtans avantaj dezavantaj sa pa youn nan kesyon ki anba yo oswa nenpòt nan bagay sa yo ou ka pa aplike oswa pa gen repons ou gen repons yo la se gwo dezavantaj pa bon menm , ti dezavantaj pa twò mal dezavantaj fèb , avantaj fèb , ti avantaj , gwo avantaj?

HM26: Ok m’ap pran ti avantaj

ITV: pou kounya.

HM26: wi Pou kounya

ITV: Presize wè e avan avan te gen.

ITV: Pou kisa wap pran ti avantaj la?

HM26 ; Bon avan te gen….avan te gen gwo avantaj

ITV:Gwo avantaj e pou dezavantaj?

HM26: Pou p’am mwen pa tèlman jwenn gwo dezavantaj paske’m pa janm pa vrèman antre nan kay la. pa tèlman antrenan …

ITV: Ebyen se bon mèsi pou tout presizyon sa yo ou bay n’ap pran yon lòt moun la n’ap…

## Household 1 / member HM30 Male / Round 2

ITV: Ok Bon la mwen nan fwaye 3 e pa sa?

HM30: Egzakteman.

ITV: An non m’nan blockk 3.

HM30: Block 3 fwaye 1.

ITV: Block 3 fwaye 1, kay HM30 la nou pwal pale sou emanatè a, alò nan ki nivo ou panse emanatè an pwoteje’w kont marengwen kap mòde’w lè’w nan kay la?

HM30: Bon nan nivo… 2.

ITV: Èske li pwoteje’w byen, tou piti, trè byen, pwoteje nèt ou byen li pa pwoteje’w lè’w nan kay la…emanatè a?

HM30: Tou piti, 2.

ITV: Tou piti… sa’k fè sa?

HM30: Bon m’pa kon si se emanatè a ki pa byen trete, paske jan’m t mete’l la m’te mete’l nan bon pozisyon, te gen anpil… te gen marengwen se vre, lè’m mete emanatè a m’wè vinn gen plis marengwen. So li pa fè gwo efè efè pou’l ta fè a, li pa fè’l.

ITV: Dakò men avan kòman sa te ye lè emanatè a te fenk vinn nan kay la?

HM30: Avan sa li te bon.

ITV: Men pandan konbyen tan li te bon konsa?

HM30: Dizon paske’m te mete’l pou yon jou aprè sa demen si Dye ve mwen al gade’l li pa ban’m bon satisfaksyon menm jan avè premye jou a.

ITV: Dakò, donk de jou an jou efikasite a ta sanble diminye.

HM30: Diminye, efè a.

ITV: Dakò, bon kesyon 2, nan ki nivo pou emanatè a pwoteje’w ak lòt moun nan kay ou kont lot vèminn lè yo nan kay la? nan ki nivo emanatè a ta pwoteje’w ak lòt moun nan kay ou kont lòt vèmin yo?

HM30: Toujou menm bagay la, tou piti.

ITV: Men nan ki nivo ou ta renmen’l pwoteje’w? èske ou ta renmen’l pwotejew nèt?

HM30: Wi m’ta renmen’l pwoteje’m trè byen.

ITV: Trè byen dakò, kesyon 3 nan ki nivo emanatè a pwoteje’w kont marengwen lè’w deyò nan lakou a?

HM30: Li pwoteje’m byen, lè’m deyò a, m’pa jwenn marengwen.

ITV: Dakò èske ou konn mete emantè a sou galri a tou?

HM30: Wi m’konn mete’l sou galri a.

ITV: Koman sa ye lè’w mete’l?

HM30: Bon m’pa konnen si pa rapò avèk lè a lè’m mete’l sou galri m’pa jwenn marengwen.

ITV: Ou pa jwenn moustik, wè dakò, e sou lakou a men’m lè ou pa mete’l ou pa jwenn moustik?

HM30: M’pa jwen moustik sou lakou a.

ITV: Kesyon 4 nan ki nivo emanatè an pwoteje’w ak fanmi’w kont lòt vèmin yo lè’w deyò kay la?

HM30: Lè’m deyò kay la li pwoteje’m, li pwoteje’m byen, wi lè’m deyò a, li pwoteje’m byen paske m’konn ap di… m’kon ap di li fè efè, apre sa tou lè’m antre m’konn chanje’l pozisyon, m’al gade si’m wè sa’m wè a si se sa pafwa jan’w wè m’sot di’w talè a, efè a konn diminye m’pa konnen sa se si se nan emanatè a li ye ou byen m’pa konnen si se apò de lè a ki pase, gen dwa pa gen marengwen tou, konsa ou konpwann?.

ITV: Donk ki sa ou tap chwazi la? ou santi lè’w deyò kay la, ou santi li pwoteje’w byen? tou piti ou byen trè byen?

HM30: Lè’m deyò kay la li pwoteje’m byen… nimewo 3.

ITV: Li pwoteje’w byen ou tap pran. 3…site ak dekri nenpòt lòt benefis oswa dezavantaj ke emanatè a bay. Ki benefis emanatè a jwenn… ou jwenn ladan’l? ki benefis emanatè a… ki sa emanatè a fè pou ou? sak benefis la?

HM30: Benefis pou’m ta jwenn…pou emanatè a ta fè pou mwen an, depi lè’l te la se premye jou a li te ban’m on bon satisfaksyon, men o bu de dezyèm jou a, tout lòt jou yo m’pat jwenn a travè anyen menm de li. So si’m ta vle pou’m ta jwenn on satisfaksyon avèl fòk m’ta jwenn on emanatè ki byen trete epi konsa tou pou travay la ka mache.

ITV: Dakò, e dezavantaj, kisa emanatè a fè’w santi ki pa bon? esplike pwoblèm li bay.

HM30: Ah, pwoblèm li bay se men’m jan ak tou talè a, se marengwen li pa repouse a, kounya li vinn fè gen plis marengwen lè konsa m’pa itilize’l menm kote a ankò, m’mete’l on lòt kote.

ITV: ALò klase avantaj ou jwenn nan emanatè a, èske li ba’w gwo avantaj ou byen ti avantaj ou byen avantaj fèb?

HM30: Avantaj fèb.

ITV: Dakò e pou dezavantaj?

HM30: Dezavantaj la li trè fèb.

ITV: Ok ,ok di ki sa emanatè sa fè pou ou pou benefis, kisa emanatè a fè pou ou nan kay la, depi li rive nan kay la kisa ou jwenn kòm benefis ?

HM30: Bon kòm benefis m’jwenn… m’te wè te gen plis marengwen, pa fin genyen’l ankò men o fi e a mezi jan’m sot di’w tou ta lè a, m’ap toujou di’l, lè’l te la a, lè’l te fenk vini a li te bon. Lè m’plase’l lòt kote so pa gen bon bagay, vinn gen plis marengwen paske youn nan dezavantaj ki fèb lakay emanatè a.

ITV: Konsta sa èske ou ka di èske premye semèn li pi bon dezyèm semèn li mwens bon?

HM30: Wi, premye semèn nan li te bon, dezyèm semèn la li pat bon.

ITV: E pou dezavantaj ou ka di ki sa … ki pwoblèm emanatè a bay sa pou dezavantaj?

HM30: Dezavantaj li bay, dezavantaj emanatè a ban mwen se trete li pa trete a, li pa ban’m bon satisfaksyon.

ITV: Ok m’konpwan, ok wityèm kesyon se menm jan avèk sa a, ou pral klase avantaj emanatè a bay.

HM30: Nimewo 3 toujou, dezavantaj fèb.

ITV: Dezavantaj feb,E pou avantaj.

HM30: Avantaj …

ITV: Avantaj fèb?

HM30: Yèp

ITV: La’w te pwan avantaj fèb, dezavantaj fèb, dakò, donk avantaj fèb sa se avan?

HM30: Avan.

ITV: Sa se aprè?

HM30: Non aprè.

ITV: Wè aprè, e avan kòman sa te ye avan? ou di’l te bon?

HM30: Li te bon.

ITV: Avan te gen gwo avantaj an kèlke sòt?

HM30: Wi te gen yon bon ti avantaj.

ITV: E pou dezavantaj la avan, kòman sa te ye avan? Avan prèske pat genyen?

HM30: Prèske pat genyen.

ITV: Avan se sa.

HM30: Pa twò nòmal.

ITV: E aprè dezavantaj fèb?

HM30: Fèb, e pou sa’m make dezavantaj.

ITV: E pou avantaj? sa se pou dezavantaj. Avan te gen ti dezavantaj, avan pat gen dezavantaj se apre ki gen ti dezavantaj fèb an kèlke sòt. Ok, e pou benefis ankò, ou pa raple’w ki benefis ou jwenn nan emanatè a?

HM30: Premye jou a m’jwenn, premye jou a te gen bon benefis nan emanatè a, m’ka di si son semèn m’pran 2 jou ladan’l, m’paka bay yon jou fix paske m’pa kite’l plase on sèl kote.

ITV: Li te travay nan 2 premye jou se sa?

HM30: Hum.

ITV: Ou di Li trè efikas nan 2 premye jou?

HM30: Wi.

ITV: E pou 10 la e kisa wap pran pou avan ki avantaj èske avan te gen gwo avantaj?

HM30: Wi te gen avantaj.

ITV: Avan gwo avantaj

HM30: Hum hum.

ITV: E aprè?

HM30: Avantaj fèb.

ITV: Ok.

HM30: Ok.

ITV: E pou kounya èske gen pou an tèm de dezavantaj ki sak gen kounya èske gwo dezavantaj ou byen ti dezavantaj?

HM30: Ti dezavantaj.

ITV: Ok e ben mèsi anpil paske nou fin pale.

HM30: Mèsi tou.

ITV: Nou pral pase a yon lòt moun.

## Household 2 / member HM27 Male / Round 2

ITV: Wi HM27, nan ki nivo ou panse aparèy la pwoteje’w kont marengwen kap mòde’w nan kay la, Èske’l pa pwoteje’w ? Èske’l tou piti pwoteje’w ? Li byen pwoteje’w ? Li trè byen pwoteje’w ou byen’l pwoteje’w nèt ?

HM27: Bon premye esè emanatè yo te ban nou pounn mete lakay nou a, m’pap di nou te jwenn satisfaksyon a 100 %, men a 50 % li te kouri dèyè moustik pandan 2 jou 3 jou, men apre sa rès semenn yo *la même chose. Dezyèm esè a m’ka di 100 % paseke li pwoteje’m vrèman, e m’gen menm vwazinn mwen m’pataje emanatè a avè’l tou.

ITV: Men nan ki nivo ? Byen, trè byen pwoteje nèt ? Si’w t’ap chwazi.

HM27: M’ka di trè byen.

ITV: Trè byen, ok, trè byen, *maintenant HM27… , anndan… lè’w pa anndan kay la, deyò kay la, èske ou panse aparèy sa pwoteje’w kont marengwen yo le’w sou galri ou byen lë’w sou lakou a ?

HM27: Wi paske lè’m sou galri a map etidye , m’ap repase dè nòt, m’mete’l devan’m nan li pwoteje’m.

ITV: Nan ki nivo ? byen, trè byen ou pwoteje nèt ?

HM27: Byen

ITV: Byen ok. Bon HM27 se toujou nan lakou a, ou di li pwoteje’w byen.

HM27: Wi.

ITV: Bon HM27 ou itilize li avèk mounn anndan lakay ou? Ak fanmi’w pa vre? Aparèy la?

HM27: Wi m’itilize’l ak manman’m, m’itilize’l avèk on bò frè’m tou, apre sa m’itilize’l ak on vwazinn mwen.

ITV: Nan ki nivo li pwoteje’w ak fanmi’w?

HM27: M’ka di pwoteje nèt paske yo pa plenyen menm jan yo te konn ap plenyen pou marengwen lontan yo.

ITV: Li pwoteje’w nèt ?

HM27: Wi.

ITV: Ok.

ITV: 5, HM27 èske’w ka site ou byen dekri pou nou kèlke avantaj aparèy sa pote pou ou ?

HM27: Avantaj sè ke avan emanatè a m’te gen moustik lakay mwen, kounye a se pa *la même chose. Li pa gen oken dezvantaj tou non.

ITV: Ou pa …,li pa gen kèk dezavantaj ke’l pote pou ou?

HM27: Non, li pa nwi’m.

ITV:Ok pa gen dezavantaj , HM27, si nou tap klase avataj sa yo li pote pou ou a, nan ki nivo nou te ka klase yo? avantaj fèb , gwo ou ti avantaj? , sinn ta ka mete yo nan on echèl?

HM27: Gwo avantaj.

ITV: Li pote pou ou gwo avantaj… kisa’l pote pou ou HM27 kòm benefis? emanatè a.

HM27: Kòm benefis sè ke m’dòmi byen.

ITV: Ou dòmi byen. Ki lòt benefis li pote pou ou ankò?

HM27: E m’dòmi an sekirite tou paske m’konnen emanatè a la, m’pa gen moustik k’ap mòde’m.

ITV: Dòmi byen, dòmi an sekirite ok. Ki lòt li pote ankò, pa genyen? Dezavantaj kisa’l pote pou ou kòm enkonvenyan?

HM27: Li pa pote enkonvenyan.

ITV: Li pa pote ankenn enkonvenyan pou ou ok…. Ou te di’l pote pou ou gwo avantaj.

HM27: Uh huh.

ITV: HM27, si nou t’ap bay aparèy la on nòt ant zero a dis, ki nòt n’ap ba li? Ki nòt ou t’ap ba li

HM27: M’tap ba’l 9 sou 10.

ITV: Ok poukisa HM27?

HM27: Paseke m’trouve fò nou ta mete plis bagay tankou…pou ta kouri dèyè moustik. Plis pwodwi pou ta kouri dèyè moustik menmsi ke m’pa bezwen’l…tankou si’l nan salon an la , m’mete emanatè a nan salon an menmsi m’sou galri a la pou moustik yo ta on ti jan pè, pou yo ta kouri.

ITV: Ok ou t’ap ba’l 9 sou 10 pou sa ok. Avèk le tan HM27 kijan’w wè efikasite aparèy la, avèk le tan li vinn pi bon? li vinn mwen bon? sa’w ka di nou?

HM27: Li vinn pi bon.

ITV: Ou ka esplike nou sa?

HM27: Sak fè’l vinn pi bon, paske premye pase a li pat bon menm, dezyem fwa a m’jwenn emanatè a se pa menm bagay la tou.

ITV: Ok avèk le tan lap vinn pi bon pou ou, bon Claudeson, si’w ta gen kèk konsèy w’ap bay mounn kap dirije pwojè yo la? Gwo zotobre yo, ki konsèy ke’w ta ka ba yo ?

HM27: Tankou doktè Chikòy?

ITV: Wi gwo zotobre yo ki la, tankou doktè Chikòy, doktè Obrillant, ki konsèy ke’w te ka ba yo pou rann aparèy la pi efikas, ou m’pa konnen ki sa’w te ka di yo?

HM27: Bon sa’m tap di sèke nan tout sa wap fè nan lavi a gen on defisi. Menm si dè fwa ke gen kote’w konn al travay emanatè kraze, pyèj kraze , gan pèdi , mta *just…pou yo toujou pa dekouraje nan sa y’ap fè a paske tout sa y’ap fè gen on rezilta nan lavi, a menm si ou pa peye’l sou tè a w’ap peye’l anlè a kanmenm, *just kenbe la. E sa m’tap di.

ITV: Men si nou…Si’w t’ap bay on konsèy pou efikasite aparèy la.

HM27: Emanatè a ?

ITV: Wi ki sa’w t’ap di?

HM27: Efikasite a sa’l ye, se met plis…, kijan yo rele bagay sa?

ITV: Poud?

HM27: Poud ladan’l, pwodwi yo met ladan’l la.

ITV: Transfluthrine.

HM27: Transfluthrine la pou’l te ka pi byen toujou.

ITV: E byen ok HM27, se te on plezi.

## Household 3 / member HM29 Male / Round 2

ITV: Donk nou pwal pale avèk HM29 nan block 3 fwaye 2, sou emanatè a la maten an, alò nan ki nivo ou panse emanatè a pwoteje’w kont marengwen kap mòde’w lè’w nan kay la?

HM29: Tou piti.

ITV: Tou piti, èske ou ka bay esplikasyon pou ki sa se tou piti?

HM29: Paske nòmalman nan premye moman yo m’wè’l te pli zou mwen efikas, avan anviwon yon semèn m’wè’l totalman redwi.

ITV: Dakò 2 nan ki nivo pou emanatè a pwoteje’w ak lòt moun lakay ou kont lòt vèmin yo lèw nan kay la? Nan ki nivo pou’l ta pwoteje’w?

HM29: Si’l te ka pwoteje nou nèt li tap bon.

ITV: Ben vwala, pwoteje nèt, ok, 3 nan ki nivo pou emanatè a pwoteje’w kont marengwen lè’w deyò nan lakou a?

HM29: li pa pwoteje’m deyò a.

ITV: Ou wè’l pa pwoteje’w lè’w nan lakou a, moustik mòde’w lè’w nan lakou a.

HM29: Uh huh

ITV: Men èske’w konn deplase’l tou met nan lakou a?

HM29: Wi m’konn mete’l deyò.

ITV: Lè’w mete’l sa sa fè? .

HM29: Lè’m mete’l, lè sa li redwi nan kantite marengwen ki tap vinn bò kote’m nan.

ITV: Dakò, donk se byen, men lè’w nan lakou a ou di’l pat pwoteje’w, li pa pwoteje’w ditou si’w pa mete emanatè a deyò…si’w pa mete emanatè a deyò li pa pwoteje’w ditou.

HM29: Non li pa pwoteje’m.

ITV: 4, nan ki nivo emanatè a pwoteje’w ak fanmi’w kont lot vèmin yo, lè’w deyò kay la? Eske li pa pwoteje’w? Eske pa gen repons?

HM29: Li pa pwoteje’m, m’pa gen repons pou sa, se anndan an tou piti sèlman.

ITV: Alò bay , di tout sa emanatè a fè pou ou di sa’k rele benefis, kisa’w jwenn nan emanatè sa?

HM29: Bon sèl sa’m wè’l bay kòm benefis nan jan’m te di’w, oparavan nan anpil moman ke se pwoteje’w kont moustik, lè’w mete’l swa anndan ou byen deyò a, nan premye semèn nan ou alèz bèt pap mòde’w, men aprè sa li pat vinn bay menm efikasite a, pa gen efikasite a ankò.

ITV: Ok sa se pou benefis, e pou dezavantaj ki pwoblèm emanatè a bay?

HM29: Bon pwoblèm li bay, sèke sèvis, sètadi nou te prevwa pou’l te bay, randman pou’l te bay la, li pa bay li pwiske se nan on sèl semèn sèlman li konsa , apre sa lòt semèn nan li pa vinn efikas, sa vinn pèmèt ke moustik yo toujou…preske rete nan men’m nivo a.

ITV: Dakò nou pra’l nan kesyon 6 nap kontinye nan avantaj nou jwenn nan emanatè avan èske te gen gwo avantaj ou byen ti dezavantaj?

HM29: M’ka di nan premye semèn la li te gen yon gwo avantaj paske li te totalman redwi e..kantite moustik ki te a *lenteryè mezoson* an.

ITV: E kounye a ki avantaj? Eske kounye a gen ti avantaj ou byen gen avantaj fèb ou byen ti dezavantaj?

HM29: Avantaj totalman fèb.

ITV: Ok e pou dezavantaj èske avan te gen gwo dezavantaj ou byen ti dezavantaj fèb?

HM29: Avan te gen ti dezavantaj fèb.

ITV: Ok e kounye a èske gen dezavantaj fèb ou byen gwo dezavantaj?

HM29: Gwo dezavantaj kounye a.

ITV: Ok, e pou benefis ki sa ou jwenn nan emanatè a, ki sa emanatè a fè pou ou lè’l rive nan kay la, sa’k rele benefis? kisa’w jwenn ladan’l? kisa’w tire ladan’l?

HM29: Bon an tèm de benefis jan’m te di’w avan, nan premye moman yo m’ka di’w li te totalman redwi kantite moustik ki tap mòde mwen, epi nan fen tankou 2, 3 semèn li totalman redwi.

ITV: E pou dezavantaj ki sa’w tap? di globalman an tèm de dezavantaj?

HM29: Bon pou dezavantaj li pa efikas, li toujou pemèt, li pemèt moustik yo toujou rete a menm volim la.

ITV: Dakò, sa se men’m kesyon an, pou avan te genyen…

HM29: Gwo avantaj aprè ti avantaj.

ITV: …E *maintenant, kounye a se avantaj fèb?

HM29: uh huh.

ITV: E avan ou te gen ti dezavantaj fèb, se sa’w te di talè a la, avan nan premye itilizasyon an.

HM29: Wi avan an te gen dezavantaj fèb.

ITV: E aprè, kounye a gen gwo dezavantaj?

HM29: Ya.

ITV: E pou benefis ki sa’w tap di ankò? men’m lè se enpe repetisyon, men ou ka panse lòt bagay ou ka di an tèm de benefis?

HM29: Wi an tèm de benefis m’ka di nan on moman done, nan premye moman, dinn pa *(d’une part) li te eseye pwoteje nou kont moustik, e pi, bon m’ka di kont lòt bagay ki te ka nwi nou nan kay lan, sa te parèt avantaj pou nou paske nan moman sa, yo te kap dòmi san’w pat bezwen kouvri, ou byen limen blakatòks. Men apre, bon li redwi, li pa gen men’m efikasite a ankò, li pa bay menm sèvis yo, m’panse se sa an gro.

ITV: Dakò sa se dezyèm kesyon an, se mènm bagay la ki la, ou te di avan gwo avantaj?

HM29: Aprè ti avantaj.

ITV: E aprè ou byen *maintenant* ti avantaj, e an tèm dezavantaj kounye a ou di se gwo dezavantaj, e byen mèsi frè pa’m mwen pou tout sa ou ban mwen, epi n’ap pase a on lòt moun la

HM29: Dakò.

## Household 3 / member HM28 Female / Round 2

Date: 27/12/18

ITV: HM28, nou fè yon pase maten an nan kad seri antretyen men nou genyen nan kad pwojè a, donk n’ap fon ti tan, kòm ou di nou tan’w pa anpil nap pran on 5 a 10 minit pou’n fè on ti kesyone ou, si sa pap deranje’w. HM28, nan ki nivo ou panse aparèy la pwoteje ou kont, kont moustik kap…kap pike’w andann kay la? Nan ki nivo emanatè a pwoteje’w? Èskel pa pwoteje’w? Ou di mwens tou piti , ou di mwens byen pwoteje’w , trè byen pwoteje’w , pwoteje’w nèt?

HM28: O kòmansman.

ITV: O kòmansman li te li te kòman? Li te byen pwoteje’w?

HM28: Wi.

ITV: Ok.

HM28: Men a la long

ITV: A la long, sa’k vinn genyen kòm pwoblèm? kisa’w konstate kòm pwoblèm?

HM28: Kòm si li mwens pwoteje’w

ITV: Ok li vinn mwens pwoteje’w. Ok lè’w deyò kay la. Èske’w panse’l pwoteje’w kont moustik yo, lè ou pa andann kay la? Lè ou deyò kay la, nan ki nivo’l pwoteje’w?

HM28: Kòm si lè’m nan lakou an epi tou aparèy lan anndan an?

ITV: Non lè, lè’w deyò a epi’l deyò a tou avèk ou, aparèy lan deyò a èske’w panse li pwoteje’w?

HM28: Bon li konn ede’w kòmsi li diminye.

ITV: Li diminye moustik yo.

HM28: E toujou o kòmansman men e apre sa’l diminye.

ITV: Ok apre l’ap di…lap diminye ok, ok donk lè ou nan lakou a ou di nou li pwoteje ou

HM28: Pa fè sa, pa fè foto non, bou nou pap fè sa

ITV: Ok trè byen, bon ou itilize li avèk mounn anndan lakay ou? HM28 ak fanmi ou? aparèy la?

HM28: Wi yo pe itilize li

ITV: Nan ki nivo ou panse li pwoteje’w ak fanmi’w? nan ki nivo’l pwoteje’w?

HM28: Men’m bagay la o kòmnansman epi tou a la fen…

ITV: O kòmansman li te pi bon e pwi a pre sa li diminye?

HM28: Li diminye.

ITV: Li diminye ok, HM28 èske ou ka site pou nou kèk avantaj aparèy sa ofri’w? kèk benefis dezavantaj? Ou ka dekri, ou ka site pou nou si’l vou plè?

HM28: Kòm si, ki sa’l fè?

ITV: Uh huh.

HM28: Ki sa’l fè?

ITV: Sa’l pote pou ou kòm avantaj kòm benefis , emanatè a?

HM28: Li chase moustik yo, m’wè li chase yo o kòmansman.

ITV: Ki lòt avantaj li pote pou ou ankò?

HM28: Li pa mòde…. Marengwen pa…..Kòm si li ede’w, marengwen pa mòde’w ou pa santi…ou pap fè tout la jounen…

ITV: Uh huh, ok bon si aparèy sa pote pou ou yon enkonvenyan ki sa’l ye? Dezavantaj?

HM28: Anyen m’pa wè anyen.

ITV: Li pa pote ankenn dezavantaj pou ou Ok.

HM28: Li pa deranje’m.

ITV: HM28, si nou tap klase yo pa òd avantaj ke’l pote pou ou , kòman nou te ka klase yo? gwo , piti , fèb ou kòman’w te ka klase avantaj yo?

HM28: Gwo, piti, fèb kòman?

ITV: Gwo avantaj, ti avantaj, avantaj fèb.

HM28: O kòmansman li gwo a la fen li kòmanse desann.

ITV: Uh huh.

HM28: Kòman’m ta mete la? fèb.

ITV: Li vinn fèb ok.

HM28: Li vinn fèb

ITV: Ok HM28 kounya nou nan nivo benefis, dimansyon benefis ki sa aparèy sa pote pou ou kòm benefis? Saw benefisye de aparèy sa? Emanatè a?

HM28: O Li te chase moustik, li te ede’m a moustik yo anpil.

ITV: Uh huh, e enkonvenyan li pa pote okenn enkonvenyan pou ou?

HM28: Non li pa fè nou anyen.

ITV: Ok ti dezavantaj fèb. HM28 si’w t’ap bay aparèy la nòt antre 0 a 10, ki nòt ke’w tap bay li si’w tap ba li on nòt?

HM28: Konbyen’m ta bali la li? paske o kòmansman li te travay byen, se a la fen li pra’l diminye, map mete’l 7 , 6 yo konsa.

ITV: Wap mete’l 7 , 6?

HM28: O wi paske li te travay ( ri) m’paka…….

ITV: Li travay byen apre sa li kòmanse ap diminye.

HM28: Hein?

ITV: O kòmansman an li tap fè 10?

HM28: Li t’ap fè 10 men apre li diminye, nòt la diminye sou li, konbyen’l 7, 7 w’pa wè

ITV: HM28 si’w tap bay mounn ki responsab pwojè a yon konsèy de aparèy la, sou aparèy la, ki konsèy li tap ye? ki sa’w tap ka rekòmande yo pou aparèy sa fonksyone pi byen?

HM28: Kòm si… m’pa konnen si se kantite bagay ki ladan’l nan pou nou ta… pou’n ta ogmante’l, pou moustik, pou’l ta ka fè plis tan e sa’m ta di, e pa sa?

ITV: Ok ou ta renmen yo ogmante pwodwi yo pou’l ta fè plis tan.

HM28: Si sa pa gen dezavantaj ladan’l tou lè’w ogmante pwodwi a èske li pap yon pwoblèm pou ,pou kay la?

ITV: Ok. E byen ok HM28, se te yon plezi pou’n te ansanm, nou di’w mèsi paske nou fè on ti pale…

## Household 3 / member HM25 Male / Round 2

ITV: Ou ka kòmanse

ITV: Ok, la nou nan fwaye 3, blòk 3, kay HM25 donk nou pral kòmanse nan kesyon 1 an wap reponn pou nou , nan ki nivo ou panse emanatè a pwoteje’w kont marengwen ka’p mòde’w lè ou nan kay la?

HM25: Tou piti

ITV: Fè yon ti wonn nan repons ki pi ba yo oswa sèke sa ki anba a

ITV: non e la.

HM25: An ok

ITV: Li pa pwoteje’w, li pwoteje’w tou piti an ok tou piti dakò. Kesyon 2, nan ki nivo pou emanatè a pwoteje ou ak lòt moun lakay ou kont lòt vèmin yo lè ou nan kay la wap fè yon ti wonn nan younn nan repons ki ba yo , tre byen ok donk li pwoteje’w trè byen? Donk nou pral nan kesyon 3 a , nan ki nivo emanatè a pwoteje’w kont marengwen lè ou deyò nan lakou a wap fè yon ti wonn nan repons ki pi ba yo donk li pa pwoteje’w? Tou piti ok. Li pa pwoteje’w lè ou deyò a? Dakò. Kesyon 4 , nan ki nivo emanatè a pwoteje ou ak fanmi’w kont lòt vèmin yo lè ou deyò lakay… la nan kay la , fè yon ti wonn nan youn nan repons ki pi ba yo oswa sèke nòt ki anba a? Donk li pa pwoteje li tou piti, byen, trè byen pwoteje nèt? donk kisa wap pran pa gen okenn repons? Dakò. 5 Site ak dekri nenpòt lòt benefis oswa dezavantaj ke emanatè a bay?

HM25: Bon kòm benefis m’te ka di se jis kòm pwoteksyon tankou pou sante ( c'est-à-dire que ) kòm li pa gen ankenn sibstans ou byen fime li degaje ki te ka nwi òganis moun la m’ka konsidere ke se yon benefis men enkonvenyan se ke o debi li , li fè on bon travay men o fir e a mezi lè mo jou ap pase sou li li diminye , nan diminye li diminye de fwa’l konn , de fwa’l konn pa fonksyone ditou e kòm si li la li pa la li pa itil anyen

ITV: An ok donk e kesyon 6 lan kounya

ITV: Sa se benfis ok e pou an, an, an dezavantaj?

HM25: M’pa gen ni dezavantaj dan le sans ke lè li kòm benefis premyeman li pa nwi sante’m mwen respire nòmalman e mwen ka, mwen ka fè tout sa’m vle anndan kay la sè ta di men, m manje anndan an san ke m’pa gen choz kap nwi mwen men kòm enkonvenyan se le tan, le tan sè ta di ke si li sèvi de jou apre res jou yo li diminye diminye jiskaske efè a disparèt nèt moustik yo repran abitid nòmal yo, jan yo konn mòde avèk valè yo konn ye

ITV: Ok kantite ok

ITV: Dakò anfèt nap kontinye kesyon 6 , klase enpòtans avantaj dezavantaj sa pa younn nan repons ki anba yo oswa nenpòt nan bagay sa yo ka pa aplike oswa pa gen repons: 1 gwo dezavantaj pa bon menm , 2 ti dezavantaj pa twò mal , 3 dezavantaj fèb , 4 avantaj fèb , 5 ti avantaj , 6 gwo avantaj?

ITV: E presize nan ki moman avan ou byen kounye a kòman sa te ye

HM25: Bon m’……..

ITV: avan te gen gwo avantaj ou byen ti avantaj?

HM25: Avan te gen gwo avantaj sè ta di ke e ou te kòm si li pase wi ou santi moustik lan la ou santi’l la men ou ta di’l poze sou ou pou’l mòde’w non o’w pa li pa ou santi li jis pase sè ta di ke li pa gen entansyon an paske li pa kapab pa konn si se sibstans ki nan emanatè an ki kòz sa men li pa kapab men aprè 2 jou 3 jou se ou kòmanse santi ke li diminye avantaj la diminye e pwi rive jiska 5 , 6 jou li disparèt konplètman li mouye yo mòde’m e lè sou premye dezyèm twazyèm jou yo mwen dòmi nòmalman men sou katriyèm , senkyèm , sizyèm jou yo moustik la nwi somèy mwen paske emanatè a pa fè travay li te konn fè a

ITV: Uh huh

ITV: Avan ou te jwenn gwo avantaj

HM25: Avan mwen te jwen gwo avantaj men aprè

ITV: Donk aprè avantaj la vin fèb?

HM25: Li Vin fèb.

ITV: Èske avan tou te gen dezavantaj prèske pat genyen?

HM25: Avan pat gen dezavantaj paske sèl dezavantaj ki pou ta genyen se pou… sè ke li ta anpeche’m fè yon bagay ki te yon woutinn jan manje , dòmi epòk an pran egzanp ke ou pran repilsif nòmal jan blakatòks ou byen begonn lè’m fè’l lò’m aplike’l anndan chanm nan ou byen kay la konplètman li kòz ou oblije sòti pou kite’l reyaji lè’l fin reyaji pou rantre men konparativman ak emanatè a ou mete’l la lap fè efè a pandan ou menm ou nan kay la ou ka kouche , chita , manje gad tele fè sa’w vle anndan an sa se avantaj ki ladan’l lan sitou sè ta di ke pat gen dezavantaj avan , avan 3 , 4 jou

ITV: Uh huh trè byen.

ITV: Donk kesyon 7 la site ak dekri nenpòt lòt benefis oswa dezavantaj ke emanatè a bay?

HM25: Bon e benefis , benefis pou’l ta ban’m an plis de sa m’sot site yo sè ke li ta , m’pa di endetèrmine men li ta gen yon efè dirab sè ta di pa 2 , 3 jou men ou te ka di yon mwa 2 mwa jiskaske li renouvle jiskaske e gen yon jan yo pase avè’l nan laboratwa yo renouvle’l men si e sè’l sa m’te ka konsidere kòm benefis anplis de sa li pote pou mwen deja.

ITV: E dezavantaj pa genyen?

HM25: Dezavantaj bon dezavantaj , li pa twò gen dezavantaj a par tan a par tan e tan defè sèlman sa li gen kòm dezavantaj aprè sa li pa gen ankò

ITV: Ok dakò , donk e kesyon 8 lan , klase enpòtans avantaj , dezavantaj sa pa younn nan repons ki anba yo oswa nepòt nan bagay sa yo donk wap wè gen gwo dezavantaj pa bon menm , ti dezavantaj pa twò mal , dezavantaj fèb , avantaj fèb , ti avantaj , gwo avantaj avantaj ki sa ou tap chwazi nan tout sa yo dezavantaj fèb?

ITV: pou kounya ou byen aprè , avan sa?

HM25: Apre sa paske avan sa pat gen dezavantaj ditou apre sa’l te fonksyone nòmalman

ITV: e pou avantaj , avantaj , gwo dezavantaj , ti dezavantaj pou kounya saw tap chwazi?

HM25: Pou kounya apre apre nomb de tan m,site jan 3 jou an 4 jou avantaj la fèb

ITV: avantaj fèb apre ….. ok kontinye

ITV: Ok kounya kesyon 9 la site ak dekri nenpòt lòt benefis oswa dezavantaj ke emanatè a bay?

HM25: Benefis e gwo avantaj , benefis e dezavantaj , benfis se jis benefis m’te ka di an plis li pa pran twòp espas ou jis depoze’l li pa twò lou a transpòte li pa tankou nan sans ke li ka blese,w bon sof si ou mal sèvi avèl paske li gen tij metalik ladan’l men a pa sa ou jis ka transpòte’l ou jis depoze’l yon kote e kòm si fonn ni nan anvironnman an ou jis depoze’l on kote on moun ka parèt li ka panse se jis on dekoratif se yon materyèl dekoratif li ye dezavantaj li genyen se jis m’ap toujou kontinye di sè ke tan , tan defikasite a ki jis dezavantaj emanatè a

ITV: Dakò kounya kesyon 10 la klase enpòtans avantaj , dezavantaj sa pa youn nan repons ki anba yo oswa nenpòt nan bagay sa yo ou ka pa aplike oswa pa gen repons donk gen gwo dezavantaj pa bon menm , ti dezavantaj pa twò mal , dezavantaj fèb , avantaj fèb , ti avantaj , gwo avantaj?

HM25: Nan sans ke pou’m ta chwazi , si pou’m ta chwazi se jan m’ka chwazi gwo avantaj dan le sans ke li tanporè sè ta di ke gwo avantaj ki ta sipoze ki sipoze nan bon pou mwen se dire a sè ta di ke dire an li gen nomb de tan li dire de twa kat jou maximòm men de gwo dezavantaj sèke se jis poul pa ta nosiv pou la sante a pa sa gwo avantaj se on dire de tan de twa a kat jou apre sa li jis parèt menm jan’m te di a li pa gen menm efè a li jis parèt tankou materyèl dekoratif sèlman sa

ITV: Ebyen se bon , epi’w te pale de dezavantaj tou?

HM25: Dezavantaj , dezavantaj mwen m’ka toujou di sè ke ou ka jis pase avèl laboratwa pou’l ta jis ke ….m’pa kon si on mwayen nou te ka jwenn pou’n ta fèl plis dire fèl dire plis epi siprime dezavantaj ki vini apre nomb de jou mwen site a sèlman sa kòm dezavantaj apre sa se yon bon inisyativ ki te ki a ta sipoze jounèlman m,pa di rantre tout repilsif ki gen aktyèlman sou le mache men ki ta jis sevi paske gen maladi kip a bon pou fime gen fime kip a bon pou seten maladi klostwòfobi ou byen asm ou byen ou te ka di emanate a te ka kouvri tout sa yo paske emanatè a pa degaje oken odè li pa deranje oken fime nan jan oksijèn e tou li pa polye oksijèn ou ou bezwen lè ou andan kay la

ITV: Ok e byen mèsi.

# IDI Block 4

## Household 1 / member HM31 Female / Round 2

ITV: Block 4, foyer 1, kay madam HM31, bonjou madam nou pwal fè on ti antretyen la, sou emanatè a, alò nan ki nivo ou panse emanatè a pwoteje’w kont marengwen kap mòde’w lè’w na kay la? èske li pwoteje’w tou piti, èske se byen, èske se trè byen, èske se pwoteje nèt ou byen èske li pa pwoteje’w?

HM31: O kòmansman li te pwoteje byen, byen byen, men kounya li vinn bese.

ITV: Li vinn bese?

HM31: Wi

ITV: E bon…sè ta di kounya , ou tap di li pwoteje’w byen ou byen trè byen?

HM31: Pa trè byen paske m’achte blakatòks tou, mwen oblije vinn mete blakatòks

ITV: Kounye a?

HM31: Wi.

ITV: Tandiske avan ou pat bezwen sa.

HM31: Uh uh ou pat bezwen sa.

ITV: Donk se byen nòmalman.

HM31: Wi wi.

ITV: Èske blakatòks sa yo se on pakèt lajan sa koute’w lè’w achte yo konsa?

HM31: Wi, ou gen dwa achte blakatòks la, bwat blakatoks la, paske lajan w’ap achte 2 blakatòks pou… a 10 goud.

ITV: Dakò men ou achte’l chak jou, souvan ou byen?

HM31: Non mwen pa itilize’l souvan, tankou si m’achte li m’limen li. Kounye a lè yo vinn bay kouran li vinn bay bagay la fòs, m’oblije m’etènn li pou’m jwenn rès la pou demen si Dye vle avan ke…

ITV: M’konpwann , men w’achte li pou’w kapab… sa ede ou chase plis moustik.menm lè emanatè a la ou oblije met… achte blakatòks paske emanatè a vinn…, efikasite’l vinn redwi.

HM31: Li vinn redwi, sak fè li… tankou’m ta di’w nan redwi li vinn redwi a, marengwen fè vironn ou men’l pa pwoche sou ou non a vrè di.

ITV: M’konprann.

HM31: Yo gen dwa ap rele nan zorèy ou.

ITV: Li ap eseye atake men’l pa rive sou ou vre. Mwen konpwann, donk lè li annd an ou pat tande moustik ditou.

HM31: Non non pat gen bagay konsa.

ITV: Ok alò nan ki nivo pou emantè an pwoteje’w ak lòt moun lakay ou kont lot vèminn yo yo lè ou nan kay la? e èske li ta dwe pwoteje’w byen ,trè byen ou byen tou piti ou byen li… vwala, lè ou nan kay la, nan ki nivo pou’l ta pwoteje’w?

HM31: li pwoteje’w trè byen.

ITV: E nan nivo sa ou ta swete poul ta pwoteje’w?

HM31: wi

ITV: Nan ki nivo emanatè an pwoteje’w kont marengwen lè ou deyò nan lakou a?

HM31: Lè’m deyò a mwen mete’l kote mwen, li pwoteje mwen… marengwen an ap rete on ti jan lwen, men’l pa pwoche sou ou men. Otrefwa, ou pat konn… Lè’w mete’l deyò an kote’w ou pat santi marengwen menm.

ITV: Menm menm, donk lè ou deyò nan lakou an, kounya li pwoteje’w byen tou piti ou byen trè byen ?

HM31: Tou piti.

ITV: Dakò e nan ki nivo emanatè an pwoteje’w ak fanmi’w kont lot vèmin yo lè ou deyò kay la? èske li pa pwoteje’w lè’w ou deyò kay la? Èske li pwoteje’w tou piti? Èske li pwoteje’w byen? Èske li pwoteje’w trè byen ou byen èske li pwoteje’w nèt lè ou deyò kay la ?

HM31: Li pwoteje.

ITV: Li pwoteje, men bay yon ti nivo, èske se byen, trè byen ou byen tou piti?

HM31: Kont lòt vèminn?.

ITV: Wi.

HM31: E byen Li pwoteje’m, mwen pa konn wè vèminn bò isi.

ITV: Sa’w tap chwazi? byen ou byen trè byen ou byen tou piti ?

HM31: Byen.

ITV: Ok, kounye la w’ap bay benefis oswa dezavantaj ke emanatè a bay ki benefis avèk emanatè sa ki sa li fè pou ou?.

HM31: Li fè pou mwen, m’pa wè… marengwen pa mòde’m, mwen dòmi, marengwen pa mòde mwen, mwen dòmi li fè.

ITV: E ki dezavantaj sètadi ki sa ki pa mache avèk emanatè an?

HM31: Fèb li fèb sèlman.

ITV: Alò, klase enpòtans avantaj ou jwenn yo, èske se gwo avantaj, ou byen ti avantaj, ou byen avantaj fèb?

HM31: Avataj la sanse fèb si emanatè a fèb, paske avantaj pou’l ta bay la li vinn…

ITV: Kounya avantaj li vinn bay la vin fèb, men avan…

HM31: Wi avan se te bon bagay nèt.

ITV: Ok, avan gwo avantaj, e kounye a ti avantaj?

HM31: Wi.

ITV: Alò, map reprann sa ankò, ki benefis ou jwenn ak emanatè an?

HM31: M’jwenn benefis, mpap achte blakatòks, yo te plizyè.

ITV: Sa se avan, avan w’tap achete blakatòks e ki benefis kounya ou jwenn?

HM31: E byen m’jwenn benefis, malgre li pa fò marengwen fè viwonn li pa rive sou mwen, dayè se yon benefis li ye, paske marengwen an te gen dwa mòde’w ou pa konn ki maladi li te ka lage sou ou.

ITV: Ok, e pou dezavantaj ki sa’k pa mache ak emanatè… ki sa, ki pwoblèm li bay?

HM31: Sè’l sa’w wè’m di’w la.

ITV: Ok, alò n’ap klase avantaj, dezavantaj yo, èske se avan? ki avantaj li te ye avan? èske te gen gwo avantaj, ti avantaj ou byen avantaj fèb?

HM31: Avan li te gen gwo avantaj.

ITV: E kounye a?

HM31: Avantaj lan fèb kounye a?

ITV: Ok. Lè ou di gwo avantaj, ou ka bay esplikasyon? ou ka esplike?

HM31: Gwo avantaj la?

ITV: Wi.

HM31: Gwo avan li te bay avan ke ou pat santi marengwen menm.

ITV: Wè.

HM31: Ou pat santi marengwen menm, ou pa nan limen blakatòks, chak fwa ou ouvè vantilatè e pou tèt chalè, apre sa tout bagay byen.

ITV: E kounye a, ou di ti avantaj, ou ka bay esplikasyon?

HM31: Ti avantaj kounya dèske ou santi marengwen ap chante, marengwen ap fè viwonn ou lap chante.

ITV: Vwala, donk ti avantaj sa … se on dezavantaj fèb… fèb an kelke sòt?

HM31: Wi.

ITV: Alò nap reprann benefis, ki benefis li bay, ki sa’w jwenn avèk bagay sa depi li vinn nan lakou a? kisa li fè pou ou depi’l vinn nan kay la ?

HM31: Li fè pou mwen, li fè pou mwen, m’pat ka dòmi ak marengwen, m’pat ka dòmi, li fè pou mwen.

ITV: E pou dezavantaj?

HM31: Sèl dezavantaj m’di’w e fèb li fèb.

ITV: Dakò, nap presize paske li vinn fèb, paske avan li te fò. Ok pou nou fini, klase enpòtans avantaj dezavantaj sa yo, e kòm kounye a ou viv emanatè an nan yon premye tan, kounya ou wè…ou di li vinn bay ti avantaj, nap di kounye a sa vle di *maintenant ki sa ou jwenn kòm avantaj? Èske son’w gwo avantaj? ou byen ti avantaj, ou byen avantaj fèb pou kounye a la ?

HM31: Avantaj fèb

ITV: Ok e avan, avan koman sa te ye?

HM31: Trè byen.

ITV: Ebyen gwo avantaj ?

HM31: Gwo Avantaj

ITV: Mèsi!

## Household 1 / member HM34 Male / Round 2

ITV: Nou nan blòk 4 fwaye 1 kay HM34, bonjou nou pral fè on ti antretyen sou emanatè a, nan ki nivo ou panse emanatè a pwoteje ou kont marengwen k’ap mòde ou lè ou nan kay la

HM34: Li pwoteje m ase byen, lè l te fèk vini li te pwoteje m trè, trè byen.

ITV: E kounya, kòman sa ye?

HM34: Kounya m pa ka pale menm jan , lè l te fèk vini na m pat konn nan achte blakatòks ditou, kounya ou vin nan obligasyon achte blakatòks ou byen veye tann lè yo bay kouran.

ITV: Ebyen kounya ou panse li pwoteje ou byen? Trè byen? Oubten pwoteje nèt?

HM34: Avan li te byen. Avan li te trè byen ou konprann?

ITV: E kounya?

HM34: Kounya , li pasab.

ITV: Nan ki nivo pou emanatè a pwoteje ou ak lòt moun lakay ou kont marengwen ak lòt vèmin yo , lè ou nan kay la? Nan ki nivo pou l ta pwoteje ou? Èske ou ta swete li pwoteje ou byen? Trè byen? Pwoteje nèt?

HM34: Trè byen.

ITV: Nan ki nivo emanatè a pwoteje ou kont marengwen lè ou de yò nan lakou a?

HM34: Bon lè m deyò nan lakou a, m pa itilize li vrèman, paske se plis galri a m itilize l avèk anndan m itilize l , m pa itilize l deyò ditou, si gen on espas m ka konsidere kòm deyò se galri a.

ITV: Nan lakou a ou pa mete l?

HM34: Non,paske jeneralman m pa chita nan lakou a tou?

ITV: Ou pa konn mete l sou galri a?

HM34: M mete l sou galri a toutan , sitou lè apremidi.

ITV: Lè ou mete l sou galri a kòman sa ye?

HM34: Li trè byen. Lè l fèk vini.

ITV: Wi.

HM34: Lè l fèk vini sou galri a nou nik mete l epi manman m oubyen matant mwen mèt chita sou galri a pa gen pwoblèm.

ITV: E kounya lè ou mete l sou galri a kòman sa ye?

HM34: Bon kounya la, li pasab, ou oblije mete l, si m chita la matant mwen oblije chita tou pre m nan, otrefwa li pat konsa,lè ou te fèk genyen l ou annik mete l sou galri a la epi tout bagay ,

ITV: Donk kounya oun t’ap di tou piti? Nan ki nivo pou emanatè a pwoteje ouak fanmi ou kont lòt vèmin yo lè ou deyò kay la ?Èske lè ou deyò kay la  pa egzanp sou galri a, èske li pwoteje ou? Galri a fè pati de kay la, se pa deyò kay la. Ou di ou pa konn mete deyò kay la.

HM34: Non, m pa konn mete l, sa vle di deyò kay la fò ou ta chita nan lakou a la.

ITV: Men yo pat di ou mete l deyò kay la oubyen oumenm ki pa mete l?

HM34: Non, paske deyò a ka , sa vle di pou m ta mete deyò a, se ta dèyè a le moman moun lakay mwen pa vrèman sòti nan lakou a, ou wè sa m vle di ou, nou jis itilize lakou a pou nou pran dlo alò emanatè a pa ka sèvi dlo , kote ki gen dlo, ou konprann sa m vle di ou an? Se sa k fè ke nou pa vle kite l pou ba l pwoblèm.

ITV: Site ak dekri nenpòt lὸt benefis oswa dezavantaj emanatè a bay? Ki benefis li bay?

HM34: Benefis li bay, li bay ke lè n gen emanatè a, pi gwo benefis li fè pou mwen m pa achte blakatòks , m pa oblije veye EDH pou yo bay kouran pou m gen , pou m alèz, avantaj sa li bay.

ITV: E dezavantaj?

HM34: Dezavantaj la se lè pa egzanp, lè li kòmanse , sa vle di pou kounya la, li vrèman , vrèman trè piti, sa vle di kounya ou oblije achte blakatòks, epi se on depans ki konn ap fèt anplis , ki pat sipoze te fèt, si m te gen emanatè, m patap fè depans blakatòks la.

ITV: M konprann, ,klase enpòtans avantaj emanatè a bay, kòm kounya avan , èske te gen gwo avantaj oubyen ti avantaj?

HM34: Avan, lè l te fèk vini?

ITV: Wi.

HM34: Te gen gwo avantaj.

ITV: E kounya?

HM34: Se on ti avantaj li bay.

ITV: Ti avantaj li deja endike la se trè byen, oke alò site epi bay lòt benefis emanatè a bay.

HM34: Youn nan benefis emanatè a bay, sèke avèk transfritrin ke yo mete ladan l lan vin fè ke m dòmi mye, lè m te fèk genyen l,sa vle di m konnen ke m’ap byen dòmi, m p’ap dòmi avèk on kè sote kote ke ou oblije plase on blakatòks nan kay la,k’ap ba ou pwoblèm respiratwa ki konn deranje m mwen men pèsonèlman , se pi gwo avantaj ke m panse m ka jwenn nan emanatè a.

ITV: Dakò, e pou dezavantaj kisa ou ka di?

HM34: Pou dezavantaj lè pa egzanp apre on nonb de tan li vin pa gen menm efikasite a ankò. Si nou te ka, m pa konnen si se chak de mwwa y’ap chan je l pou yo te ka ban ou pwodui pou nou enjekte ladan pou li kapab reprann menm efikasite a m panse li t’ap pi bon.

ITV: Oke, bon sa se on kesyoon 6, 8 la yo menm sanble,,klase enpòtans avantaj emanatè a bay pou kounya ki avantaj ki genyen?

HM34: Kounya, ki avantaj li genyen, sa m’obljie, avantaj li genyen an li piti.

ITV: Donk se on ti avantaj an kèlke sòt.

HM34: Ti avantaj,

ITV: E avan?

HM34: Avan se te on gwo avantaj.

ITV: E pou benefis lan kisa ou ap di?

HM34: Bon benefis ke li bay, li pèmèt ke,ou viv byen kont tout vye ensèk sa yo.

ITV: E pou dezavantaj?

HM34: Dezavantaj lè ou pa gen emanatè a, ou oblije itilize blakatòks , itilize lafimen , lòt pwodui pou ou kapab chase marengwen.

ITV: Sa se si ou genyen l, lè m di dezavantaj, se ki dezavantaj ki soti nan emanatè a.

HM34: Nan emanatè a dezavantaj ke l pwodui jan m di l lan se efikasite a ki redui.

ITV: Oke, nou pral pase na dènye kesyon, èske emanatè a bay gwo avantaj? Ti avantaj? avantaj fèb?

HM34: Lè l fè k vini li bay gwo avantaj.

ITV: Avan e kounya?

HM34: Ti avantaj.

ITV: E pou dezavantaj avan kòman sa te ye?

HM34: Avan?

ITV: Wi.

HM34: Avan m pat ko wè.

ITV: Ebyen n’ap mete okenn repons , e pou kounya èske gen on ti dezavantaj oubyen genn on gwo dezavantaj?

HM34: Gwo dezavantaj paske li pa genyen menm efikasite a.

ITV: Ebyen, mèsi frè pa mwen pou antretyen sa ou te akspete akòde nou, n’ap swete ou bon jounen.

HM34: Dakò.

## Household 2 / member HM32 Male / Round 2

Date: 20/12/18

ITV: Nou nan block 4 e mwen pwal fe on antretyen la avek HM32 nan foyer 2. E byen vwala HM32 nou pwal pozew kek kesyon sou aparey la sou komanw viv si gen bagay ki negatif di yo si gen gen bagay ki pozitif tou dil. Ddonk pa mare pa santiw pouw di bagay ki negatif o kontre sa ape de nou chanje bagay yo apre si nou konnen yo. Alò nan ki nivo ou panse emanate a pwotejew kont marengwen kap modew leuw nan kay la eske li pa pwotejew eskel pwotejew tou piti eske se byen eske se tre byen eske se pwoteje net.

HM32: Premye moman lè’l fenk vini li pwoteje’m anpil.

ITV: Wi.

HM32: Li pwoteje’m anpil.

HM32: Wi premye moman lè’l fenk vini, moustik pa nan kay la, e lwen’m moustik pati. Men kounya li diminye, li manyè diminye paske’l ta merite swa chanje… ou konpwan?

ITV: Donk li pwoteje’w kounya tou piti ou byen?

HM32: Tou piti Li pwoteje’m tou piti, men jan’l te pwoteje’m nan li pa pwoteje’m konsa ankò.

ITV: Dakò.

HM32: Li manyè fèb.

ITV: Ok, Nan ki nivo pou emanatè a pwoteje’w ak lòt mounn nan kay nan, kay ou kont lòt vèminn yo lè’w an kay la?

HM32: Lè’m nan kay la moustik pa vinn bè kote’m menm, e lwen’m yo pase , tankou mennm ravèt, ou konpwann ?

ITV: Wi.

HM32: Menm ravèt.

ITV: Nan ki nivo ou ta renmen pou’l ta pwoteje’w èske se ta pwoteje’w tou piti ou byen byen , trè byen ou pwoteje nèt?

HM32: Non li pa pwoteje’m, m’santi pou’l ta pwoteje’m pi byenn, pi byen ou konprann?

ITV: Dakò.

HM32: Men kounya li manyè fèb ou konpwann?

ITV: Ok nan ki nivo, emanatè a pwoteje’w kont marengwen lè’w deyò nan lakou?

HM32: Lè’w nan deyò nan lakou a yo deyò, a men anndan kay mwen’l pa rive nan chanm mwen, li pa rive ditou menm si yo ta parèt yo ale, li pa touye yo non men’l kwape yo.

ITV: Donk deyò a li pwoteje’w, nan ki nivo? se tou piti, byen, trè byen?

HM32: Tou piti deyò a, men anndan an li pwoteje’m anpil.

ITV: Ok 4, nan ki nivo emanatè a pwoteje’w ak fanmi’w kont lòt vèminn, lè yo deyò kay la?

HM32: Lè yo deyò kay la, yo deyò, men anndan kay mwen yo pa antre, yo antre tou piti nan ti kwen, men bò kote pam yo pa rive.

ITV: Dakò donk lè’w deyò a ou santi’w pwoteje nèt , ou byen tou piti, ou byen byen?

HM32: Non tou piti.

ITV: Dakò.

HM32: Tou piti.

ITV: Ebyen ok site epi dekri nenpòt lòt benefis oswa dezavantaj emanatè a bay.

HM32: Ebyen li bay anpil.. mwen pa malad, moustik pa mòde’m, bon si moustik mòde’m m’ap malad men sil pa mòde’m mpap malad e m’byen dòmi, ou konpwann?

ITV: Sa se avantaj.

HM32: Wi se on avantaj.

ITV: E pou dezavantaj kisa’w jwenn kòm dezavaj?

HM32: Avataj paske’m pa malad, paske’m pa malad, m’jwenn avantaj. Lèfini mwen byen dòmi, bèt pa rive sou mwen.

ITV: Men èske emanatè a pa bay pwoblèm tou?

HM32: Non , ki pwoblèm, li pa ban’m pwoblèm ditou, ankò mèm li pwoteje’m.

ITV: Dakò. donk, klase enpòtans avantaj/dezavantaj sa yo sa a, younn nan repons ki anba yo, oswa nenpòt nan bagay sa yo. Èske… èske se gwo avantaj? Èske se ti avantaj? pou avantaj Èske’l gen…li bay gwo avantaj?

HM32: Wi li ban’m gwo avantaj.

ITV: Avantaj fèb, ti avantaj piti, oswa ti avantaj?

HM32: Gwo avantaj.

ITV: Dakò.

HM32: wi wi.

ITV: E pou dezavantaj, èske pa gen? Éske dezavataj la li fèb ?

HM32: Non, kounye a li vinn fèb , li fèb.

ITV: Èske gwo dezavataj? Èske pa bon menm ditou? Èske se on bagay… Èske emanatè a pa bon ditou?

HM32: Non li pa bon, li manyè mye, manyè fèb.

ITV: Lè’m di dezavantaj sèta di pwoblèm li rankontre yo, pwoblèm ou rankontre emanatè a bay. Èske se on pwoblèm ki piti ou byen se on gwo pwoblèm?

HM32: Non pwoblèm nan piti menm, pwoblèm nan pitit paske li febli, paske premyeman lè’m te fenk jwenn li an moustik pat rive kote’m menm, men kounye a yo pa rive sou mwen non, yo nan on kwen.

ITV: M’konpwann.

X: Se… se, ou konnen pwodwi ki nan trasfluthrine nan gen on nonb de tan li fè.

ITV: Donk se dezavantaj…, on dezavataj ki fèb ou tap pwan?

X: Feblès pwodwi a nan…nan…nan transfluthrine nan, nan emanatè a, e sèl sa wi, li pa dirab, sèl dezavantaj li pa dirab, li diminye avèk le tan.

ITV: Donk ki benefis ou jwenn menm?

HM32: benefis mwen jwenn moustik pa mòde’m, m’byen dòmi, m’byen reHM32ire, men depi’w ap dòmi moustik pase sou ou faw faw ou pantan, men kounye a m’pa pantan m’jwenn bon avantaj, m’byen dòmi m’byen respire.

ITV: E pou dezavantaj kisa’w tap di pou dezavantaj? sa ki pa mache?

HM32: Non kounye a li manyè febli, li febli, li pa rive sou mwen menm.

X: Se dirabilite e…e…emana…e transfluthrine nan emanatè a ki dezavantaj la, sèl sa sèlman.

ITV: Wap viv menm kote avè’l?

X: Wi.

ITV: Klase enpòtans avantaj dezavantaj, bon sa se on kesyon ki bagay déjà , donk pou avantaj èske se gwo avantaj ou byen ti avantaj?

HM32: Gwo avantaj.

ITV: Ok men pou kesyon dezavantaj, èske se on dezavantaj ki fèb? èske se on ti dezavantaj tou piti

HM32: Tou.. li fèb …on dezavantaj tou piti, tou piti, li fèb paske yo pa rive sou mwen, men yo lwen.

X: paske a la minit yo flite transflitrin nan bagay la se on bagay ki…

ITV: Nap repwann benefis ankò, ki benefis ou jwenn lè’w itilize emanatè a?

HM32: En ben mpa malad, moustik pa mòde’m ankò, m’byen dòmi, m’byen respire.

ITV: E pou dezavantaj ou pa jwenn?

HM32: Dezavantaj, li febli, li febli.

ITV: E si w’ap klase avantaj yo, èske wap pran ti avantaj, avantaj fèb ou byen gwo avantaj?

HM32: Gwo avantaj mta renmen.

ITV: E pou dezanvantaj? wap pwan ti dezavantaj ou byen gwo dezanvantaj?

HM32: Gwo dezavantaj mta renmen, li tap bon pou mwen.

ITV: Bon’w di dezavantaj…

X: Dezavantaj wi…

ITV: lè’w di dezavantaj, se tèm nan ki mal tradwi, lè’w di dezavantaj se sa ou te atann ke emanatè an ta ba ou ou pa jwenn li, e pwoblèm li bay, èske li pa baw sèvis?

HM32: li ban’m sèvis men li ban’m sèvis fèb.

ITV: E byen wap di yon ti dezavantaj tou piti, e byen mèsi, mèsi pou pawòl ou a site pou mwen, ok vwala bòn jounen.

HM32: Ok dakò mèsi.

## Household 2 / member HM35 Male / Round 2

Date: 20/12/18

ITV: Blòk 4 fwaye 2 kay HM35, nou pral fè on ti pale sou emanatè a, nan ki nivo ou panse emanatè a pwoteje ou kont marengwen k’ap mòde ou lè ou nan kay la? Èske li pwoteje ou byen, trè byen, pwoteje nèt oubyen li pa pwoteje ou?

HM35: Li pwoteje m nèt, paske lè ou gen emanatè ou kouche bò kote l, depi l nan zòn ou marengwen pa pase kote ou ditou, donk li pwoteje nèt.

ITV: Dakò, nan ki nivo pou emanatè a pwoteje ou ak lòt moun lakay ou kont marengwen ak lòt vèmin yo , lè ou nan kay la?

HM35: A la minit ke ou gen emanatè a bò kote ou , ou te mèt te avèk timoun bò kote ou , ke ou t’ap regle on aktivite , ke ou t’ap fè manje, ke ou t’ap kwizine, ou ap gad lapotòp pa egzanp on seri, bèt pa apwoche ou ditou.

ITV: Donk ou panse se trè byen.

HM35: Trè byen.

ITV: Nan ki nivo pou emanatè a pwoteje ou kont marengwen lè ou deyò a nan lakou a?

HM35: Nan lakou a se otre choz paske emanatè a pa avè ou , se anndan li ye si li deyò a ansanm avè ou l’ap pwoteje ou men si se deyò li pa la, pwoteksyon an ap fèb.

ITV: Se on presizyon ou te dwe pote dè depa donk lè ou mete emanatè a avè ou kòman sa ye?

HM35: O li mye , sitou nan boutik papa m nan kòmsi gen marengwen , dè fwa m konn mete l anndan nan boutik lan , lè m kanpe m’ap sèvi, lè sa marengwen pa atake m ankò, sitou m toujou a bout pantalon mwen menm.

ITV: Wè, men si ou pa gen emanatè a bò kote o..

HM35: Ou chofe.

ITV: Donk kisa out’ap chwazi la na repons yo sijere la, lè ou deyò lakou a.

HM35: Avèk amanatè, trè byen san emanatè pa gen pwoteksyon,

ITV: Oke nan ki nivo emanatè a pwoteje ou ak fanmi ou kont lὸt vèmin yo, lè ou deyὸ kay la?

HM35: Lè ou deyò kay la, timoun yo jeneralman, m gen ti neve n ki toujou ap jwe m konn, youn nan foto yo, n’ap remake sa , m kite timoun yo ap jwe emanatè a bò kote yo a. Y’ap jwe li pwoteje yo san pwoblèm.

ITV: Dakò, èske se byen? Trè byen?

HM35: Trè byen.

ITV: Site benefis oswa dezavantaj emanatè a bay? benefis dabò.

HM35: Avantaj emanatè a bay leswa ou pa bezwen achte blakatòks tankou si gen kouran ou bezwen vantilatè, emanatè a kòmsi ke ou genyen li fè ofis de tout bagay sa yo nèt, sa se pou avantaj la, pou dezavantaj la , inik dezavantaj ki gen nan emanatè a sèke dirabilite , li gen on nonb de tan, li gen on nonb de tan defikasite , apre on onb de tan, efikasite amwendri se sèl sa ki genyen kòm dezavantaj

ITV: Dakò, klase enpòtans avantaj , dezavantaj sa yo nan repons ki pi ba,èske se gwo avantaj?

HM35: Gwo avantaj.

ITV: Pou kounya oubyen avan?

HM35: Gwo avantaj pandan emanatè a, pandan ou fèk genyen l lan , li ak plèn kapasite l gwo avantaj totalman , apre on nonb de tan, kòmsi ou gen anviwon plis on mwa depi m genyen l m’ap sèvi avè l la, sa vle di e li …

ITV: Donk pou dezavantaj, èske se on gwo dezavantaj  ki genyen oubyen ti dezavantaj? Dezavantaj fèb.

HM35: Ti dezavantaj , paske pou rezoud li osi senp se jis transfritin nan pou enjekte.

ITV: Ti dezavantaj sa yo se pou kounya e avan sa te gen avantaj , oubyen pat gen dezavantaj? Pou kounya la ou di gen on ti dezavantaj ou jis mete transfritrin nan e avan èske te gen ti dezavantaj oubyen gwo dezavantaj.

HM35: Pat gen dezavantaj ditou , se ti dezavantaj nèt.

ITV: Avan?

HM35: Wi avan.

ITV: Ti dezavantaj nèt kounya oubyen avan te genyen ti dezavantaj oubyen?

HM35: Pat gen dezavantaj menm ti dezavantaj emanatè a te genyen , tout dezavantaj se anfen de peryòd.

ITV: Ki benefis emanatè a bay?

HM35: Emanatè a jan m sot di ou talè a la, li ranplase plizyè bagay tèlke blakatòks, kòmsi ou pa bezwen chak swa pou al nan achte plizyè blakatòks epitou answit si gen kouran , pa gen kouran ou toujou ap frape, ou toujou pwoteje kont moustik, se benefis sa yo li rapòte.

ITV: Pou dezavantaj?

HM35: Pou dezavantaj la, m pa wè gen dezavantaj paske emanatè kote ou mete l li bon , alaminit ke ou pa kòmsi fèmen l on kote , li bon tout kote ou mete l li bon.

ITV: Klase enpòtans avantaj, dezavantaj vwala se menm kesyon 6 la, pou avantaj èske se avantaj fèb, oubyen ti avantaj oubyen gwo avantaj?

HM35: Gwo avantaj, men sitou li pa toksik , li pa ba ou pwoblèm, li pa afekte sante ou, se on gwo avantaj emanatè a ye.

ITV: E pou dezavantaj, èske se on dezavantaj ki fèb? Ti dezavantaj pa twò mal oubyen on gwo dezavantaj?

HM35: On ti dezavantaj tou fèb.

ITV: Oke , ann reprann kesyon sa avan, e pou benefis li bay, kisa emanatè a bay nan kay la? Ki benefis? Kisa ou wè? Kisa ou tire de emanate a?

HM35: Bon, emanatè a lefè ke ou gen timoun nan kay la e ke ou gen emanatè a la, li pwoteje ou kont marengwen ki ka ba ou vye maladi sitou timoun nan bazaj, emanatè a li vin enpòtan pou li anpil , marengwen p’ap mòde l, li pwoteje totalman.

ITV: Wè, pou dezavantaj pou timoun, èske gen dezavantaj pou timoun?

HM35: Pa gen dezavantaj, sitou ke li pa toksik , li pa ba yo pwoblèm sante , pa gen dezavantaj ditou avèk emanatè a.

ITV: Oke dènye kesyon, klase enpòtans dezavantaj, avantaj say o, pa youn na repons ki anba yo, nenpòt nan bagay sa yo, pou avantaj.

HM35: Gwo avantaj.

ITV: Pou dezavantaj, èske se gwo dezavantaj, pa two mal, dezavantaj fèb? Avantaj fèb? Gwo dezavantaj?

HM35: Ti dezavantaj.

ITV: Dakò ebyen mèsi Gregory pou antreteyn sa ou aksepte ban mwen.

HM35: Dakò.

## Household 3 / member HM33 Male / Round 2

Date: 20/12/18

ITV: Mwen nan blòk 4 fwaye 3 kay HM33 ,bonjou nou pral fè on ti pale sou emanatè a, nan ki nivo ou panse emanatè a pwoteje ou kont marengwen k’ap mòde ou lè ou nan kay la? Èske li pwotete ou byen, trè byen, pwoteje nèt oubyen tou piti, oubyen li pa pwoteje ou?

HM33: Trè byen.

ITV: Trè byen, li pwoteje ou trè byen kounya oubyen avan?

HM33: Trè byen avan.

ITV: E kounya?

HM33: Kounya m ka di tou piti.

ITV: Dakò, kounya se tou piti. Oke nan ki nivo pou emanatè a pwoteje ou ak lòt moun nan kay la kont lòt vèmin yo , lè ou nan kay la? Nan ki nivo pou l pwoteje ou? Èske li dwe pwoteje ou nèt? Trè byen? oubyen tou piti ?

HM33: Tankou lè ou di kont lòt vèmin se lòt bagay?

ITV: Lòt bèt, ensèk yo e vwala.

HM33: M ka di li piti.

ITV: Tou piti? Sa se nan nivo li pwoteje ou kont yo menm. Men nan ki nivo ou ta renmen l pwoteje ou?

HM33: M ta renmen l pwoteje m trè byen tou,.

ITV: Oke nan ki nivo pou emanatè a pwoteje ou kont marengwen lè ou deyò nan lakou a?

HM33: Byen.

ITV: Lè ou di byen, èske ou konn mete l deyò a?

HM33: Wi, m konn mete l ,kote m m’ap deplase nan lakou a m toujou ansanm avèk li.

ITV: Dakò, Oke nan ki nivo emanatè a pwoteje ou ak fanmi ou kont lòt vèmin yo , lè ou deyò lakou a, deyò kay la?

HM33: M ka di tou piti.

ITV: Dakò, Ki benefis emanate sa bay?

HM33: Li pèmèt ke m sev kòm, m t’ap plede achte lòt bagay.

ITV: Sev sa vle di fè ekomoni?

HM33: Ekonomi, wi m t’a plede achte lòt bagay pou pouse marengwen yo sa vle di.

ITV: Ki dezavantaj li bay? Ki pwoblèm li bay?

HM33: Sèl pwoblèm li bay , pou mwen se gwosè a, paske li pran on ti plas, men se pa on bagay kip ran anpil plas non , se gwosè a sèlman ki pou mwen li dezavantaj , apresa m pa wè kenn dezavantaj ankò.

ITV: Klase enpòtans avantaj emanatè a bay,n’ap di pou kounya èske gen gwo avantaj oubyen ti avantaj? Èske gen ti avantaj fèb?

HM33: Pou kounya se on avantaj fèb.

ITV: E avan ki avantaj ou te jwenn?

HM33: Avan se te gwo avantaj.

ITV: Dakò, site benefis ou jwenn nan emanatè a? Ki bebefis li bay? Kisa ki pozitif?

HM33: Sa ki pozitif li bay sèke moustik yo mode m mwens, pa gen moustik depi lè m te gen emanatè a na men mwen epi li fasil pou transpòte.

ITV: E pou menm dezavantaj yo?

HM33: Se toujou menm bagay la m ka di, sèke li on tijan pran on ti espas anplis

ITV: Klase enpòtans avantaj emanatè a bay,n’ap di pou kounya ki avantaj li bay, èske gen gwo avantaj oubyen ti avantaj? Èske gen ti avantaj fèb?

HM33: Ti avantaj.’

ITV: Pou kounya.

HM33: wi.

ITV: E avan, kòman sa te ye?

HM33: Se te on gwo avantaj.

ITV: Ki sa emanatè sa fè pou ou nan lavi ou?

HM33: Li anpeche m atrape maladi ke moustik la te ka pote ban mwen epi li fasilite kòmsi m ta di pou repouse moustik yo kòmsi m ta di, li pa gen on odè ki nui m lè m’ap dòmi epi setou.

ITV: E pou dezavantaj menm kisa ou ap di?

HM33: M pa wè dezavantaj non.

ITV: E pou kounya pou dènye pwen an, si n’ap pran gwo dezavantaj pabon menm, ti dezavantaj ,pa two mal , dezavantaj fèb, avantaj fèb kisa ou kapab pran nan tout bagay sa yo?

HM33 ; Repran l pou mwen ankò.

ITV: Gwo dezavantaj pa bon menm, ti dezavantaj pa two mal , dezavantaj fèb, avantaj fèb kisa ou kapab pran nan tout bagay sa yo?

HM33: M t’ap pran dezavantaj fèb.

ITV: Dezavantaj fèb, e pou avantaj menm?

HM33: Gwo avantaj.

ITV: Men avan ou te di gwo avantaj se te avan.

HM33: Wi avan, ou ap pale pou kounya?

ITV: Wi.

HM33: Ti avantaj pou kounya.

ITV: E avan se te?

HM33: Gwo avantaj.

ITV:: Ebyen, mèsi frè pa m nou fini.

HM33: Oke.

# IDI Block 5

## Household 1 / member HM36 Female / Round 3

ITV: Nan ki nivo ou panse aparèy la pwoteje pou ou on pwoteksyon kont moustik k’ap mòde ou anndan kay la?

HM36: Emanatè a li se on aparèy ki ekstrèmeman enpòtan se vre, men kèk fwa, sa pa anpeche kèk ti grenn mòde ou. Ou konprann sa m di ou la? Men li repouse marengwen yo totalman.Men gen kèk ti grenn ki konn enfiltre ki mòde ou kanmenm.

ITV: Èske li pwoteje ou tou piti? Trè byen? Pa pwoteje ou? Pwoteje nèt?

HM36: Li pwoteje m tou piti.

ITV: Alò, nan ki nivo pou emanatè a pwoteje ou ak lòt moun nan kay la kont lòt vèmin yo, lè ou nan kay la?

HM36: Nòmalman emanatè a, li pwoteje nou kont vèmin, nòmalman moun k’ap viv bò lakay yo, sitou ou konnen nou gen de emanatè,sa vle di nou mete chak nan on chanm kote moun yo ye a, ou konnen l’ap pwoteje yo nòmalman men ka gen kèk grenn ki enfiltre ki vin mòde ou.

ITV: M konprann.

HM36: Men se on trè bon aparèy.

ITV: Bay benefis emanatè a bay?

HM36: Emanatè a, benefis li bay,sa yo te chwazi pou l te fè nòmalman, li fè l menmsi li pa fè l asan pou san men kanmenm, li reponn. E mwen menm tou ki benefisye a, m satisfè de li paske dè fwa m konn ap jwe la avèk on patenè m la,anlè a m konn ap jwe sou do kay la,nou mete l bò kote nou an,e li evite tou twòp marengwen vin pike nou, se nòmal.

ITV: Èske ou wè li bay dezavantaj?

HM36: Bon avantaj li bay se paske nòmalman li kouri dèyè mawengwen.

ITV: Oke, èske li bay dezavanataj?

HM36: Dezavantaj li ka bay, petèt m ka pa two konn twòp dezavantaj li,e m pa konnen nan ju k’ap vini yo,men pou moman aktyèl la,sof m di ou gen kèk ti enfiltrasyon,marengwen yo konn fè on ti parèt dè fwa,lè ou mete l a kote ou la, mwen menm ak patnè a antre nou,nòmalman si gen kote pou marengen an pase pa lòt bò, li pase, men m wè l plis bay avantaj ke dezavantaj.

ITV: Dakò.Kounya nou pral klase enpòtans avantaj yo.Èske se avantaj fèb? ti avantaj? Gwo avantaj? Oubyen èske se dezavantaj fèb, ou ka gade la pou gen on ide.

HM36: li bay on avantaj fèb kanmenm.

ITV: E pou dezavantaj? Èske se dezavantaj fèb? Ki pa two mal?

HM36: Li bay on avantaj ki pa two mal.

ITV: Ki lòt benefis emanatè a bay, lòt benefis, sa vle di lòt avantaj.

HM36: Si l bay on lòt avantaj mwen pa konnen ki lòt avantaj li bay egzat, men sof m konnen emanate a la,pou li anpeche, pou kouri dèyè marengwen yo,pou l anpeche yo mode nou,se pi gwo avnatj ke nu te ka genyen.Menm lè m mete avantaj fèb men se kanmenm paske gen kèk enfiltrasyon, paske m panse lè emanatè a on kote patap gen moustik ditou men lefè ke m jwenn kèk ti moustik ki pou mode mwen, se sak fè m mete l nan sans sa, men kanmenm li anpeche tankou jan kip ou ta gen kantite marengwen ki konn souk ay la, ki konn ap mòde nou,li anpeche yo vini sou nu pa gouf.

ITV: E pou dezavantaj kisa ou ap di? Ki lòt dezavantaj ou wè li bay si genyen?

HM36: Si ta gen dezavantaj li ta bay, li mwens jan ou wè nu te di a la,ti dezavantaj pa two mal.Se sa sèlman m ka di wi,

ITV: Ebyen se bon nou rive la, mèsi.

## Household 2 / member HM37 Male / Round 3

ITV: HM37, mwen kontan avèk ou la maten an, 26 avril nou pral pale de eksperyans ou fè avèk emanatè a.Pou kesyon 1 an men sa li di, nan ki nivo ou panse aparèy la pwoteje pou ou on pwoteksyon kont moustik k’ap mòde ou anndan kay la?

HM37: Mwen men m, nòmalman m dòmi nan chanm mwen, lè m pot ko genyen marengwen te konn anmède m anndan anpil,men m vin reyalize ke depi lè li nan kay la, m pa di ke li fè tout marengwen nan kay la ale men,li ede m anpil ak marengwen yo, paske lè m nan chanm mwen m mete l bò kote m dòmi m pa santi marengwen menm.

ITV: Dakò, kòman ou ka mezire fason li pwoteje ou,èske li pa pwoteje ou ditou?Èske li pwoteje ou on tijan? Byen? Trè byen? Oubyen? Ou ap chwazi youn nan repons sa yo.

HM37: Li pwoteje m byen.

ITV: Dakò,kesyon 2 a, nan ki mezi emanatè a pwoteje ou avèk fanmi ou kont lòt vèmin yo, lè ou nan kay la?

HM37: Bon li pa pwotele tout moun anndan kay la nòmalman, paske m kwè ke,se nan chanm mwen an m mete l, chanm mwen an pa gen marengwen men lòt kote yo gen marengwen.

ITV: Èske li pwoteje ou kont lòt bèt? Lòt vèmin?

HM37: Wi,m te konn wè annipye pafwa nan chanm nan,avèk petèt ravèt, m reyalize m pa wè sa yo ankò,yo disparèt, m pa wè to ankò.

ITV: Kòman ou santi li pwoteje ou? Ou santi li pwoteje ou kont bèt sa yo? On ti jan? Trè byen?

HM37: On ti jan.

ITV: Kesyon twa, nan ki nivo emanatè a pwoteje ou kont mawengwen, lè ou deyò nan lakou a?

HM37: On ti jan.

ITV: Si ou vle bay espliksyon ou ka pale, lè ou deyò kay la, kòman li pwoteje ou?

HM37: Lè m deyò kay la m toujou santi marengwen an,men se lè m antre anndan chanm nan m pa santi l ankò, menm lè pa gen kouran. Lontan lè m pat genyen kouran se te on pwoblèm, m pat ka dòmi. M te konn dòmi nan salon, lè m genyen l kit pa gen kouran,m anndan paske nòmalman,gen on travay ke li ap fè, li bon, pwodui ke nou genyen an.

ITV: Oke, Kijan emanatè a pwoteje ou oumenm ak lòt moun nan kay la kont lòt vèmin yo, lè ou deyò kay la?

HM37: Li pwoteje m ase byen.

ITV: Bay avantaj ou jwenn nan emanatè a? Ki benefis ou jwenn ladan?

HM37: Kèk avantaj nou jwenn nan bagay la, li ede m avèk moustik avan moustik te konn ap plede mòde m sou kò m,m’ap plede grate, menm moman li vini nan kay la,sa yo pa rive m ankò.

ITV: M konprann. E pou dezavantaj? Èske gen enkonvenyan sa bay emanatè a?

HM37: Li pa gen dezavantaj ke li bay non. Li pa aji sou sante nou,lè ke m pot ko genyen l, m te anfòm.Men mwen vin genyen l kounya m toujou anfòm, m pa gen okenn dedomajman, m trè anfòm.

ITV: Nou prale nan sizyèm kesyon an, nou te pale de avantaj, dezavantaj. Pou avantaj èske se avantaj ki fèb? On ti avantaj oubyen on avantaj ki enpòtan?

HM37: Avantaj sa li enpòtan anpil menmsi li pa voye tout marengwen ki nan kay la ale.

ITV: Dakò, e pou dezavantaj,èske se on dezavantaj ki fèb? ti dezavantaj? Gwo dezavantaj?

HM37: On ti dezavantaj, trè fèb.

ITV: Esplike sa dezavantaj fèb.

HM37: Li pa repouse tout marengwen,pou l te ban ou on avantaj ki plen, ki t’ap pi bon,li t’ ap ansante tout marengwen anndan kay la,kenbe l nan kay la, nou toujou jwenn marengwen nan lòt chanm yo,li pa bay on avantaj a san pou san.

ITV: Ki tout avantaj ou jwenn nan emanatè a?

HM37: Tout avantaj, lè ou pale de tout avantaj,lè l vin nan kay la sa li fè pou mwen premyeman pa gen ravèt nan chanm mwen ankò,pa gen marengwen nan chanm mwen nòmalman, chanm pa m nan m ka di pa gen marengwen m dòmi alèz,e lòt avantaj li bay ankò, m te gen blakatòks m te konn achte m pa sèvi a sa ankò paske m genyen l, blakatòks fini pou mwen.

ITV: Se trè byen, e pou enkonvenyan, dezavantaj?

HM37: M pa wè anyen m te ka di ankò pou li,se on bon travay.

ITV: Kounya nou prale nna 8èm kesyon an, pou avantaj sa yo, èske se avantaj ki enpòtan anpil,oubyen modere, oubyen ti avantaj,

HM37: Se on avantaj ki enpòtan anpil pou mwen.

ITV: Sa k fè sa?

HM37: Rezon an si m te knn nan chanm mwen m’ap plde depanse kòb achte blakatòks chak jou,10 goud, pafwa m te konn achte de m mete, si kounya la,kòb sa ret nan pòch mwen, m regle lòt bagay avè l, m pa achte blakatòks, avantaj sa li bon anpil.

ITV: Oke, e pou dezavantaj? Èske son dezavantaj ki fèb? Oubyen on ti dezavantaj? Gwo dezavantaj?

HM37: Li pa on gwo dezavantaj, fèb.

ITV: Ebyen dakò.Èske ou gen lòt avantaj ankò ou ka bay sou emanatè a?

HM37: Lòt avantaj nan ki sans?

ITV: Ou di pa egzanp li ede ou pa achte blakatòks. Èske ou gen lòt avantaj ankò ou jwenn nan emanatè a?

HM37: Emanatè a gen anpil lòt avantaj, li repouse moustik.

ITV: Dakò, n’ap tounen nan kesyon 10 la,èske se on avnataj enpòtan modere?

HM37: Avantaj la toujou enpòtan, li ede ak marengwen.

ITV: Oke, e pou dezavantaj? On dezavantaj ki fèb? Gwo dezavantaj? Ti dezavantaj?

HM37: Fèb?

ITV: Sa k fè ou di li fèb?

HM37: M te di ou sa deja, li pa repouse tout marengwen yo pou mwen,m t’ap kontan si n te vini ak youn ki toujou marengwen, sa ke m pa konprann kòman fè li repouse marengwen yo li pa touye yo? M ta renmen nou vini avèk youn ki kapte marengwen yo, ki tiye marengwen yo.

ITV: M konprann, mèsi HM37.

## Household 3 / member HM38 Female / Round 3

ITV: Bonjou HM38, nou kontan avèk ou maten an,nou nan fwaye de enpas rèn kanapevè,nou pral poze ou kèk ti kesyon sou fason ou itilize emanatè ae sou eksperyans ou fè avèk li,premye kesyon an nna ki mezi emanatè a pwoteje ou kont moustik lè ou anndan kay la?

HM38: Li trè ede m paske te gen anpil moustik anndan, li kouri dèyè yo,..

ITV: Ou mèt pale wi,èske ou santi li pwoteje ou trè byen, malman oubyen byen, on ti jan?

HM38: Trè byen.

ITV: Oke, nou prale nna kesyon 2, nan ki mezi emanatè a pwoteje ou avèk fanmi ou kont lòt vèmin yo, lè ou nan kay la?

HM38: Sèl kont moustik li ede m.

ITV: Dakò, ou pa wè li pwoteje ou kont ravèt?

HM38: Ditou.

ITV: Dakò, twa, nan ki nivo emanatè a pwoteje ou kont mawengwen, lè ou deyò nan lakou a?

HM38: Lè m sou lakou a, m mete l bò kote m,si m’ap fè on lòt bagya m mete l bò kote m tankou lè m’ap lave, oswa lè m kanpe nan kay la.

ITV: E kòman ou wè sa, èske ou li pwoteje ou?

HM38: Li pwoteje m.

ITV: Èske ou li pwoteje ou byen, trè byen, ou on ti jan oubyen pa ditou?

HM38: Li pwoteje m byen.

ITV: Oke,nou prale nan kesyon kat la,nan ki emanatè a pwoteje ou ak fanmi ou kont lòt vèmin yo, lè ou deyò kay la?

HM38: Li pa ede m kont lòt, sof ke kont moustik.

ITV: Dakò,n’ap avanse avèk kesyon yo, ki avantaj ou jwenn nan emanatè a depi ou ap itilize l? Ki benefis asa ba ou?

HM38: Bon avantaj lan sè ke moustik pa vin pike m, li pwoteje m.

ITV: Oke,pa gen lòt avantaj ankò ou wè?

HM38: Non.

ITV: Nou prèske fini ou mèt on ti pasyante, èske gen enkonvenyan ou jwenn nan emanatè a, kisa ou jwenn kòm dezavantaj?

HM38: M pa jwenn anyen non.

ITV: Alò pou avantaj,n’ap eseye klase avantaj sa yo, èske se on avantaj ki enpòtan? Ti avantaj? Oubyen on avantaj fèb?

HM38: Enpòtan.

ITV: E pou dezavantaj, èske ou wè gen dezavantaj?

HM38: Non, m pa wè sa.

ITV: E pou avantaj èske gen bagay ou ka di sou avantaj ou jwenn nan emanatè a?

HM38: Wi, li pwoteje m kont moustik ou konnen ke m gen on bebe tou,li pwoteje l pou mwen tou pou moustik pa pike l.

ITV: E pou enkonvenyan, dezavantaj?

HM38: M pa jwenn.

ITV: Nan avantaj ou jwenn yo, si ou ap klase yo, èske ou ap di se on avantaj ki enpòtan oubyen on ti avantaj, on avantaj ki fèb?

HM38: Enpòtan.

ITV: E pou dezavantaj?

HM38: Ditou, m pa jwenn.

ITV: Talè a ou te di avantaj, ou di li pwoteje ou ak tibebe a tou, èske gen lòt avantaj ankò pèsonèlman ou jwenn nan emanatè?

HM38: Li ede m avèk bebe a,pèsonèlman lie de m mwen menm.

ITV: Avan emanatè a te la, kòman sa te ye moustik yo?

HM38: Te gen anpil, yo te nwizib.

ITV: Wè, te gen anpil, e kounya ou di?

HM38: Yo diminye,pa genyen ditou paske m pa wè yo.

Dakò: E pou dazavantaj?

HM38: M pa jwenn.

ITV: Oke,n’ap klase yo, ou te deja di avantaj se avantaj enpòtan epi kisa ou ta renmen di ankò sou emanatè a?Ki remak ou genyen?

HM38: Remak ke m genyen, emanatè a trè itil epi, m wè l trè bon, on moun ta sipoze posede l.

ITV: Dakò, mèsi.

## Household 4 / member HM39 Female / Round 3

ITV: Bonjou HM39, nou kontan avèk ou maten an, nou nan fwaye twa enpas rèn kanapevè,nou pral poze ou kèk ti kesyon sou fason ou itilize emanatè a sou eksperyans ou fè avèk li,premye kesyon an,nan ki mezi emanatè a pwoteje ou kont moustik lè ou anndan kay la?

HM39: Bon, pwoteje li pwoteje m,ou konnen lè marengwen an mòde ou gen on seri de mikwòb li ka pote pou ou, men pa rapò de mete nou mete l, lè n’ap dòmi nou mete l kote nou,pa tèlman gen marengwen.

ITV: Èske ou santi li pwoteje ou totalman, trè byen, byen, on tijan, oubyen pa ditou?

HM39: Bon, mwen sa k fè m pa ka fin di li trè byen nèt,li pa nan epòk marengwen epi mwen pa estab la,se plis travay de nwi m fè, ou konprann? Se timoun mwen yo ki la, men oparavan li te byen nòmal paske m te la lè sa,

ITV: E lè sa te gen anpil moustik?

HM39: Te gen anpil marengwen.

ITV: Lè sa ou te ka di se te byen.

HM39: Wi, trè byen.

ITV: Trè byen avan e kounya?

HM39: Pou kounya la, m pa konn kòman.

ITV: Oke, kesyon de, nan ki mezi emanatè a pwoteje ou avèk fanmi ou timoun oubyen mari kont lòt vèmin yo, lè ou nan kay la? Èske li pwoteje ou kont ravèt, mouch etsetera.

HM39:M pa wè ravèt, te konn genyen, se ti sourit m konn wè.

ITV:Èske ou pasne se emanatè a ki fè yo pa vini?

HM39: M pa swiv byen non,sak fè m pa swiv, m konn genyen l vrèman.

ITV: Men lè emanatè a rive nan kay la,èske avan emanatè a te vin nan kay la te gen ravèt?

HM39: Wi te genyen.

ITV: E lè emanatè a vin nan kay la?

HM39: M pa wè yo.

ITV: Ou panse se emanatè a ki vin nan kay la?

HM39: Limenm pou l ye paske se pandan li anndan m pa wè ravèt.

ITV: Dakò, ou santi se yon pwoteksyon total? Trè byen?

HM39: On pwoteksyon trè byen.

ITV: Nan ki nivo emanatè a pwoteje ou kont mawengwen, lè ou deyò nan lakou a?

HM39: M gendwa ap lave la, m pran l m mete l kote m nan, mouch yo gendwa ap pike m mete l kote n nan m pa wè mouch, li pouse mouch a marengwen anpil.

ITV: Kijan ou t’ap klase repons lan ou t’ap di, totalman, trè byen, byen, on ti jan?

HM39: Byen.

ITV: Kesyon 4 la, nan ki mezi emanatè a pwoteje ou ak fanmi ou kont lòt vèmin yo, lè ou deyò kay la? tankou mouch ounyen ravèt etsetera, lè ou deyò kay la.

HM39: Wi, li pwoteje m.

ITV: Li pwoteje ou men nan ki sans? Èske se byen, trè byen? Totalman?

HM39: Totalman li pwoteje m.

ITV: Oke, gen on lè pat gen emanate la, gen on lè vin genyen, depi emanate a rive nan kay la ki avantaj li bay?

HM39: Pou benefis mwen jwenn nan, m plis pa wè marengwen avèk moustik, ravèt tou, ki te konn on jan vini, bon m pa wè yo ankò,marengwen, te gen anpil marengwen vrèman,men m pa wï yo ankò. Si se pou pwoteksyon marengwen an. Li bay napil pwoteksyon marengwen.

ITV: E pou enkonvenyan, ki dezavantaj ou jwenn ladan, èske ou jwenn enkonvenyan nan emanatè a?

HM39: Non, m pa gen okenn enkonvenyan m jwenn non, a tout sa, se avantaj m jwenn.

ITV: Oke, ann gade enpòtans, no pale de avantaj, dezavanataj, men avantaj sa yo, èske se on avantaj ki enpòtan, on ti avantaj, oubyen on avantaj ki fèb?

HM39: Trè enpòtan.

ITV: Oke, e pou dezavantaj? kisa ki pa ale avèk emanatè a? Ki bay pwoblèm?

HM39: Non, li pa banm pwoblèm non, se timoun yo ki te di m on lè konsa ke y opa ka pran sant li, m di bon nou konnen li gen yon medikaman ladan l, sa pa pwoblèm, medikaman ki ladan l lan, se pou marengwen an. Li pa bay okenn pwblèm.

ITV: Yo pat ka pran sant li, kisa yo te di lè sa?

HM39: Yo di yo pat ka pran, men apresa yo vin alèz avè l. Se yo menm ki plis itilize l, mwen menm m’al travay.

ITV: Èske oumen tou, ou te gen pwoblèm avèk sant yo te di li genyen an?

HM39: Non,nenpòt kote m kouche li mèt lajounen m toujou pran l mete kote m,dakò.

ITV: Donk pou dezavantaj, èske pa genyen? Li tou piti, li fèb? Enpòtan?

HM39: Non, avantaj la enpòtan.

ITV: E pou dezavantaj?

HM39:M pa gen oken dezavantaj.

ITV: Oke, e pou avantaj, kòman, ki lòt avantaj ou sonje emanatè a poye pou ou nan kay la?

HM39: Bon avantaj li pote se kont marengwen,kont ravèt, se avantaj sa paske gen de lè m konn ap dòmi, dra m konn kovri tèt mwen paske marengwen tèlman rele nan zòrèy mwen,, plakatòks la konn pa ka pran sant li ankò,men limenm akoz de limen, m pa tande marengwen ankò. Se on avantaj sa ye kanmenm.

ITV: Ou te di se te yon gwo avantaj e pou dezavantaj ou te di ou pa wè dezavantaj. Kisa ou genyen pou ta di ankò sou avantaj emanatè a bay.

HM39: Li banm anpil avanataj paske marengwen te konn genyen anpil, anpil.Menm la ou konn wè m oonn chita la,marengwen yo konn ap tèlman mòde pye ou,yo fè pye m cho, epi m’ap lave la, m mete l kote m nan, pandan m’ap lave a.

ITV: Se sa nou wè la.

HM39: M itilize l anpil, m di genlè m’ap pran youn m’al nna travay la ansanm avè l.Doktè a m t’ap tann vini pou m mande l, èske m pa ka deplase l? Paske se djòb sekirite m fè, tankou demen lè m fè nwit.

ITV: Ki kote ou ap travay?

HM39: M’ap travay boudon.

ITV: Gen anpil moustik la.

HM39: Wi, anpil moustik.

ITV: Ou di se on avantaj ki enpòtan, m note sa pou ou, e pou dezavantaj ou di ou pa wè dezavantaj.

HM39: M ka poze ou kesyon?

ITV: Wi, èske se pouse li pouse marengwen an, oubyen li touye l?

ITV: Li pa touye l, li pouse l.

HM39: Si l te touye l ou t’ap wè l, paske se li menm ki la avèk de ti mesye m yo.

ITV: Mèsi anpil, ou bay anpil enfòmasyon ki enpòtan.

# IDI Block 6

## Household 1 / member HM43 / Round 3

ITV: Nan ki nivo ou panse emanatè a pwoteje ou kont marengwen k’ap mòde ou lè ou nan kay la? Èske li pwoteje ou tou piti? Byen? trè byen? Pwoteje nèt? Oubyen li pa pwoteje ou? ou ap chwazi youn nan repons sa yo.

HM43: M’ap mete tou piti, paske trè byen an fò ou ta kapab chita epi ou disponib, ou mete l kote ou,pou wè ki vrèman efè ke li fè vrèman.

ITV: Donk ou pa tèlman itilize l? Ou pa tèlman disponib?

HM43: Mwen pa tèlman itilize l, m pa tèlman chita, m toujou nan lari, m pa nèg ki tèlman chita nan kay,men tankou leswa lè m mete l la kanmenm, kantite marenwgen ki konn genyen an pa gen kantite marengwen an la, men lajounen m poko janm chita la pou m swiv,m te di banm mete l bò kote m nan la.

ITV: Ou pa disponib, ou ap vann oubyen ou ap?

HM43: Non, m p’ap vann non, m toujou gen lòt aktivite, apresa m toujou nan lari.

ITV: Ou ap brase?

HM43: M’ap brase.

ITV: Oke nan ki nivo pou emanatè a pwoteje ou ak lòt moun nan kay la kont lòt vèmin yo, lè ou nan kay la? Tankou mouch pa egzanp.

HM43: Mwen m ta renmen kòmsi, m pa konn si pwodui ke n mete ladan yo a pou ogmante l plis paske lè kòmsi ou gen timoun,mwen gen de timoun, plis madanm mwen e lakay la se on zòn ki plen marengwen li ye, m ta renmen yo mete plis pwodui ladan epi pou lè yo kouche la, pou yo pa di ke marengwen ap nwi yo, pou m pa leve m jwenn mak sou yo, paske gen anpil marengwen, si se pou marengwen genyen yo lakay la vre.

ITV: Wi, paske la genyen.

HM43: Sa ka rive li diminye yo, men genyen, toujou gen marengwen.

ITV: Sitou la.

HM43: Ravin yo, epi gen dlo bò lakay la k’ap koule, menm lè ke se pa on kanal men pa janm pa gen marengwen lakay la m ta renmen pou l ta vin plis efikas toujou, m pa konn si se pwodui pou yo ta ogmante.

ITV: Dakò, nou pral nan kesyon twa, nan ki nivo emanatè a pwoteje ou kont mawengwen, lè ou deyò nan lakou a? Kò m ou di lajounen ou pa la, èske le swa.

HM43: Leswa li pwoteje nou on ti jan paske marengwen yo te konn fè plis efè paske lè moun kouche tankou lè pa gen kouran konsa ou konn ap tande bri yo nan zòrèy ou anpeche ou dòmi, ou pa tande bri yo menm jan ankò, ou konprann.Menm lè de twa grenn mòde ou kanmenm men kantite ki te konn genyen an pa genyen l ankò. Se sa k fè m mande pou ta gen plis pwodui nan bagay yo, pou m pata menm tande yon grenn menm

ITV: Ok.Nou pral nan kesyon 4 la, nan ki nivo emanatè a pwoteje ou ak fanmi ou kont lòt vèmin yo, lè ou sou lakou a, deyò kay la?

HM43: Sou lakou a, sa m ta k’ap di, kòm mwen pa chita, lè m pa la tou m panike, paske m gen on timoun ki tou piti ki avè m,li gen dezan on mwa,m pa ka mete deyò a,san m pa la paske li menm l’ap toujou gen tandans pran li pou l jwe,fòk gen on gramoun la ki pou ap siveye l, tankou m te gendwa chita la,m mete l bò kote m nan la pou m wè ki efè li fè paske se lè sa m kapab di, pwoteksyon li pral fè plis la ankò,se pa rapò ou gen timoun ou konnen gen maladi malarya ki deyò, tout efè,m vin jwenn on pwoteksyon pou fanmi an tou,paske avèk maladi ki deyò,malarya tout kalite maladi, vye bèt sa yo ap vin mòde lòt moun apresa pou yo vin mòde ou,se maladi y’ap pote.

ITV: Oke, emanatè a nan kay la, ki benefis li pote pou nou?

HM43: Li pote on soulajman pou mwen paske,’à nos jours’, ou pa gen on bagay efikas vrèman ki pou kouri dèyè marengwen, e marengwen an li menm se maladi li vin mete nan kò ou. Men avèk emanatè a ki la, son pwoteksyon ke li ye pou fanmi an tou,se sak fè ke li pote on solisyon pou mwen kanmenm.

ITV: Ki dezavantaj ou wè emanatè a bay? Èske li gen dezavantaj?

HM43: M poko kabap di li bay dezavantaj paske, jan mwen di ou, fason ke m ta renmen itilize l la,m pa jwenn li, se pa kouri sèlman pou l t’ap kouri dèyè marengwen m te vle depi marengwen an paske kote l, li detui l.

ITV: Oke, talè a nou te pale de avantaj, dezavantaj,pou avantaj, èske se gwo avantaj li bay, oubyen ti avantaj, avnataj fèb.

HM43: Avantaj fèb.

ITV: E pou dezavantaj,èske se on dezavantaj fèb oubyen ti dezavantaj sa vle li pa two mal,oubyen on gwo dezavantaj,sa vle di li pa bon ditou.

HM43: Non, m pa di li pa bon non,m pa ka di li pa bon non.

ITV: Kisa ou t’ap pran?

HM43: M te ka toujou pran dezavantaj fèb.

ITV: Oke pou nou fini,ki lòt benefis emanatè a bay?

HM43: Se toujou menm bagay yo toujou, paske se jan m te reponn ou la, avantaj fèb la, se paske m ta vle li plis touye moustik yo ke li chase yo. Lè sa se on gwo avantaj li t’ap ye pou mwen.

ITV: E pou dezavantaj ki lòt dezavantaj ou wè?

HM43: M pa wè.

ITV: Mèsi anpil pou tan sa ou ban mwen.

HM43: M te fè nou tann mwen, eskize m pou reta, m kontan pou pasyans nou, m apresye sa.

ITV: Pa gen pwoblèm.

## Household 2 / member HM41 Male / Round 3

OD: Dakò HM41, nou kontan avèk ou maten an, nou pral poze ou kèk ti kesyon sou fason ou itilize emanatè, sou eksperyans ou fè avèk li, sa ki pozitif ou negatif, si ou di on bagay ki negatif sa ape de nou amelyore l pi devan, premye kesyon an, nan ki mezi emanatè a pwoteje ou kont moustik lè ou anndan kay la?

HM41: Lè yo te ban mwen l premye fwa, J3, m te mete li m te wè li te efikas, m pat gen moustik ak marengwen ankò, men an aprè on de semèn, doktè a te vin pote on lòt pou mwen ankò kite J6, m twouve sa vrèman efikas toujou, m t’ap mande si pa genyen ki J 7 oswa 9, oswa 12 paske toutan nimewo a pi wo, genlè li plis efikas pou mouch avèk marengwen.

OD: Pou pwoteksyon sa yo, èske li pwoteje ou trè byen?totalman oubyen byen, on ti jan, oubyen pa ditou?

HM41: Li pwoteje m totalman.

OD: Oke, kesyon de, nan ki mezi emanatè a pwoteje ou avèk fanmi ou kont lòt vèmin yo, lè ou nan kay la? Èske li pwoteje ou kont ravèt, mouch etsetera.

HM41: Mwen sèl sa m ka mande, èske nou pa ta ka mete a lavni pou kont mouch pou mwen tou, paske mwen menm m wè mouch men lè m mete l, li pouse l, men li pa vrèman pou pouse l san pou san.

OD: Donk ou santi li pwteje ou, on ti jan oubyen byen oubyen trè byen?

HM41: Pou mouch lan li pa fin san pou san li ka on swasanndi pou san, paske jan li fè efikas pou marengwen ak moustik lan, li pa fè konsa pou mouch.

OD: Nou t’ap di li pwoteje ou byen, trè byen on ti jan kont mouch lan?

HM41: On ti jan. Li pa fin trè byen nèt.

OD: Kesyon twa,nan ki mezi emanatè a pwoteje ou kont moustik lè ou deyò nan lakou a? Èske li pwoteje ou kont moustik emanatè a?

HM41: Wi, paske depi m chita deyò a, m gen anpil zanmi m lè m chita, m mete l la,m pa gen moustik a marengwen.

OD: Ou santi ou pwoteje l totalman oubyen trè byen?

HM41: Totalman.

OD: Kesyon kat, nan ki mezi emanatè a pwoteje ou kont lòt vèmin lè ou deyò nan lakou a? Èske li pwoteje ou kont mouch oubyen ravèt, lòt bèt ki nwizib emanatè a?

HM41: Non, m poko wè,m poko eseye l nan ravèt, nan mouch lan m eseye l, m’ap esye l nan ravèt epi m’ap ba ou rès pwosesis yo.

OD: E pou mouch lan kòman ou wè sa ye?

HM41: Pou mouch lan li amelyore, li pa san pou san men li amelyore.

OD: Ki avantaj ou jwenn depi lè ou ap itilize emanatè a? Kisa sa pote pou ou kòm benefis?

HM41: Depi lè m’ap itilize l, li vrèman pote on benefis san pou san pou mwen paske m te chaje marengwen, m te chaje poustik, tout devan lakay la toujo gen dlo ke m pa jwenn marengwen ankò ak moustik

OD: E ki lòt avantaj ou jwenn apa sa?

HM41: Li pèmèt lè m’ap dòmi m pa jwenn moustik k’ap nwi m, m dòmi ase byen, menm lajounen m gendwa kouche epi m mete l bò kote m, m viv alèz.

OD: M konprann obyen dòmi, e ki lòt avantaj ankò?

HM41: M jwenn tout avantaj, si m pat jwenn on avnataj m t’ap di ke m pa jwenn.

OD: E pou dezavnatj, sa vle di sa ki pa ale, ki pwoblèm li bay, èske li bay pwoblèm emanatè a?

HM41: Non, m pa jwenn.

OD: E pou avantaj sa, èske se on ti avntaj, on avantaj ki enpòtan, on avantaj ki fèb?

HM41: On gran, gran avantaj.

OD: E pou dezavantaj ou di ou pa wè.

HM41: Non.

OD: Kisa ou ka di ankò sou avantaj ak dezavantaj?

HM41: Avantaj pwodui a vrèman efikas. M pa jwenn okenn rapò ki negatif de pwodui a, men sa mwen menm m vle a lavni de pwodui a se pou mouch lan, se pou yo mete yon pwodui ki plis menm jan avèk marengwen a moustik, pou l konsa pou l ka evakye mouch yo san pou san.

OD: Dakò, si ou ap klase avantaj sa yo, se on avnatj ki enpòtan, modere oubyen fèb?

HM41: Avantaj ki enpòtan.

OD: Pou dezavantaj ou pa jwenn anyen.

HM41: M pa jwenn anyen ladan.An giz pou m ta jwenn, m gen zanmi lè m mete l bò kote yo, yo di m ba yo l, m di yo se avè l pou m fè pwosesis la, avè l pou m fè alaji administrasyon an, m oblije pa ka ba yo l, m oblije ap mande nan biwo a, m rapòte yo ki valè moun k’ap mande, pou doktè a jwenn avè yo, pou yo ka bay non, pou m fè yo ba pou pwodui a mache, pou yo ka vin konprann pwdui a sou le mache.

OD: Tout moun ta renmen genyen l daprè sa ou di?

HM41: Wi, tout moun ta renmen genyen l, paske gen moun ki chita, k’ap enjòy yo, de twa byè, m mete l bò kote yo, pouse moustik, pouse marengwen, kounya pa genyen, yo mande m men m pa ka ba yo pa m nan paske se avè l pou m fè pwosesis yo, pou m fè elaji konpayi an.

OD: Dakò, kisa ou ta gen ankò pou ta di sou avantaj ak dezavantaj?

HM41: Pou avantaj m ta konsidere, pou doktè a ta fè lòt pwodui pou lpote pou mwen, pou m ka bay moun k’ap mande m yo.

OD: Lòt pwodui sa vle lòt emanatè?

HM41: Wi, paske yo wè l vrèman efikas, yo bezwen pou yo antre nan pwojè a.

OD: Mèsi anpil pou enfòmasyon sa yo, m’ap di yo bòn jounen, bon retablisman paske ou te tonbe.

## Household 3 / member HM40 Female / Round 3

ITV: Bonjou, nou kontan avèk ou maten an HM40, nou nan fwaye twa, nou pral poze ou kèk ti kesyon sou fason ou itilize emanatè, sou eksperyans ou fè avèk li,premye kesyon an,nan ki mezi emanatè a pwoteje ou kont moustik lè ou anndan kay la?

HM40: Bon, li pwoteje m anpil, malgre m wè genyen bon, se pa tèlman lè yo tou, men yo te konn fatige nou anpil, men li pa two fatige n kounya.

ITV: Moustik yo?

HM40: Wi.

ITV: Bon, kòman ou te ka di li pwoteje ou? Èske se byen, totalman? trè byen?

HM40: Trè byen.

ITV: Oke, kesyon de, nan ki mezi emanatè a pwoteje ou avèk fanmi ou timoun oubyen mari kont lòt vèmin yo, lè ou nan kay la? Èske li pwoteje ou kont ravèt, mouch etsetera.

HM40: M’ap gade plis, men y opa tèlman, men plis pwoblèm nou se plis foumi,ki konn plis fatige nou.

ITV: Ou pa wè li chase foumi?

HM40: Wi, m wè foumi yo fon ti mwens.

ITV: Yo bese

HM40: Wi.

ITV: E pou tankou mouch ki bay pwoblèm, ravèt?

HM40: M te mande doktè a sa si l pa ka pote pou nou pou mouch tou, li di li t’ap gade sa pou nou.

M wè yo kòmsi plizoumwen.

ITV: Men pou li menm ou pa wè li chase mouch ak lòt bèt yo?

HM40: Wi, li chase yo wi.

ITV: Kòman ou wè l chase yo? Èske se byen, trè byen ou totalman? On ti jan?

HM40: M’ap mete li byen.

ITV: Ok ann mete l byen,, lè ou deyò kounya,: nan ki nivo emanatè a pwoteje ou kont mawengwen, èske se byen, trè byen, totalman, on ti jan, pa ditou?

HM40: Bon, lè nou deyò, nou konn mete l nan lakou,li plizoumwen pwoteje nou.

ITV: Tankou la ou deyò a la,èske ou santi li pwoteje ou?

HM40: Wi, li pwoteje nou.

ITV: Èske se byen, trè byen, on ti jan?

HM40: M’ap mete byen.

ITV: Oke,nan ki mezi emanatè a pwoteje ou ak fanmi ou kont lòt vèmin yo, lè ou deyò kay la? tankou mouch ounyen ravèt etsetera, lè ou deyò kay la.

HM40: Li plizoumwen pwoteje nou, men n’ap swiv plis.

ITV: Pwoteksyon ou jwenn nan,èske se pwoteksyon ki total, trè byen, byen, on ti jan oubyen pa ditou?

HM40: Bon, totalman.

ITV: Ou santi li pwoteje ou totalman lè ou deyò a?

HM40: Wi.

ITV: Kont lòt vèmin sa vle di mouch, paske fòk ou bay enfòmasyon,sou bagay pu viv tout bon vre, e fòk ou pa ezite di bagay ki negatif, ann bay tout avantaj ou jwenn nan emanatè a, ki avantaj ou jwenn?

HM40: Nou jwenn on bon avantaj,malgre n’ap swiv plis toujou,nou pa jwenn ove avantaj, nou jwenn bon avantaj.

ITV: Ki avantaj, ou ka bay esplikashyon? Ki avantaj ou jwenn?

HM40: Lè amanatè a nan kay la,tankou pitit sa li konn pa ka dòmi avèk moustik, kòm m pa konnen se pa sezon yo,paske lè se sezon yo, n’ap swiv plis.M pa tèlman wè ditou.

ITV: E pou enkonvenyan, ki dezavantaj ou jwenn emanatè a bay?

HM40: Nou poko jwenn dezavantaj paske ou konnen nou fèk genyen l, nou genyen, nou dezwen swiv li plis.

ITV: Depi kilè ou genyen l la?

HM40: Nou poko gen mwa edmi.

ITV: E pou avantaj ou jwenn èske se on avantaj ki enpòtan, on ti avantaj oubyen on avantaj ki fèb?

HM40: M’ap mete on avantaj kip a two fèb.

ITV: Ou t’ap di modere?

HM40: Wi, modere.

ITV: E pou dezavantaj, èske ou jwenn nan emanatè a?

HM40: Nou poko jwenn dezavantaj.

ITV: Oke, si t’ap dekri avantaj emanatè a genyen,kisa ou t’ap di sou sa?

HM40: M t’ap di n’ap gade plis ke sa toujou, m’ap mete kontwòl toujou, men jiska prezan m pa ka di mal de li.

ITV: Nou pa jwenn dezavantaj?

HM40: Nou poko jwenn dezavantaj.

ITV: Si ou wè dezavantaj, fòk ou pa kache sa.

HM40: Ebyen se sa m si ou wi, n’ap swiv plis toujou, ofieamezi ou ap vini n’ap ba ou plis.

ITV: Pou avantaj, kòman ou te klase avantaj yo, èske se on avantaj enpòtan,modere oubyen ki fèb?

HM40: M’ap toujou mete l modere.

ITV: Dakò.Ou di o pa wè dezavabtaj, dezavantaj sa vle di sa ki pa mache ladan,ki pwoblèm li bay se sa ki dezavantaj.

HM40: Se toujou swiv n’ap sziv li plis ke sa.

ITV: Pou la moman se oke,kisa ou t’ap ankò sou avantaj avèk enkonvenyan ou jwenn nan emanatè a?

HM40: en konvenyan pou nou ta jwenn, nou fèk genyen, gen eksperyans nu pa fè ak li.

ITV: E pou avantaj,èske se on avnatj ki enpòtan, ti avnatj, avantaj fèb?

HM40: Avantaj enpòtan.

ITV: E pou dezavabtaj ou di?

HM40: M pa jwenn.

ITV: Ou pa gen on remak pèsonèl ou ta renmen fè sou emanatè a?

HM40: Kòmantè pèsonèl, la m pa wè anyen ki mal ladan, n’ap swiv li ou konn m fèk genyen l.Pwoblèm nou plis genyen se foumi ak mouch, m te di mesye a sa.On kesyon m’ap poze ou èske li pa ogmante tout sa yo foumi, mouch, moustik?

ITV: Ogmante nan ki sans?

Ef: Èske li ka mwens yo? paske se foumi an nou plis genyen.

ITV:Ou wè li pa fè anyen sou foumi?

HM40: Li on ti jan mwens ke jan l te ye a.

ITV: Ou pasne se emanatè a ki ka?

HM40: Wi, m pasne se li.

ITV: Dakò, mèsi.

## Household 4 / member HM42 Male / Round 3

ITV: Mwen lakay ou HM42 nan Bourdon, nou pral fè on ti pale sou fason ou itilize emanatè a, nan ki nivo ou panse emanatè a pwoteje ou kont marengwen k’ap mòde ou lè ou nan kay la? Ou mèt pale.

HM42: Lè m nan kay la, lè m mete l nan espas kote m chita, moustik la konn gen tandans vin anvayi nu, men sitou se nna pye ou oubyen sou tèt ou lè ou chita,men lè ou pran emanatè a, ke ou depze bò pye ou, oubyen sou tab la tou nou konn mete l,sèke li kouri dèyè do yo,pafwa genyen ki konn gen tandans ap kouri avèk on vitès men kou l rive nan espas ankò, ou wè li tounen.

ITV: Oke nan ki nivo pou emanatè a pwoteje ou ak lòt moun nan kay la kont lòt vèmin yo, lè ou nan kay la?

HM42: M ta renmen ke emanate a pou l ta fè efè sou mouch,kòm m pa fè eksperayns lan dabò,lè m itilize l pou m wè èske li fè efè sou mouch,oubyen li pa fè, m pral gade pou m wè a pati de jodya eksperyans mwen, si m mete èske li fè kèlke efè sou mouch oubyen li pa fè, m’ap fè nou konn sa men,nòmalman m ta renmen pou emanatè a pote yon rezilta byen sou afè mouch paske nou konnen mouch la avèk moustik lan se de, m ka di se konfrè yo ye,youn pa mache kite lòt.

ITV: Dakò, nou pral nan kesyon twa, nan ki nivo emanatè a pwoteje ou kont mawengwen, lè ou deyò nan lakou a?

HM42: Lè pou m deyò nan lakou an, sèke,m ka di se toujou menm bagay la men ou konnen lakou a on tijan pi vas, men ti espas lè m chita nan lakou a,ke m toujoun itilize, ke m toujou jwenn ke li pwoteje byen. Pa egzanp, li toujou kouri dèyè do moustik lan,lè o chita yo gen tandans vin anvayi ou,pa egzanp pose sou ou san presizyon men ‘il suffit’ ke aparèy la bò kote ou,ou wè yo on tijan fou, yo fou, youn ka poze fap apre sa li kouri li ale,y opa rete, lè sa tou,ou tiye yo pi fasil.

ITV: Oke, kesyon kat, Oke nan ki nivo emanatè a pwoteje ou ak fanmi ou kont lòt vèmin yo, lè ou deyò lakou a, deyò kay la?

HM42: M poko fè remak pou lòt vèmin yo,m pral swiv sa epi sa mwen wè, m’a pote presizyon.

ITV: Dakò.Alò nou pral nan kesyon 5. Site epi dekri nenpòt lòt benefis ou jwenn emanatè a bay? Dabò n’ap pale de benefis, ki benefis emanate a bay?

HM42: Bon,benefis li bay, sitou moustik nan peyi sa, li pote anpil maladi,si tout fwa nou ta gen espasyalis k’ap panse sou sa e ki ka fè aparèy, pandan ou chita, oubyen ou kouche,ki te ka kouri dèyè do bèt sa yo vrèman, li t’ap vrèman efikas se sak fè tou, m te enterese nan pwojè sa,pou nou mete plis efikasite, plis pwodui pa rapò nan sa m’ap viv pou le moman epi mete plis efikasite jis pou nou ka benefisye, se pa mwen sèlman, tout moun ki sèvi avèk aparèy sap ou yo ka jwenn on chans avèk bèt ki rele moustik sa.

ITV: Ki dezavantaj emanate a bay? Èske ou wè li bay dezavantaj?

HM42: Bon, m pa fin di li bay dezavantaj, paske yo te di ke litiye moustik, li pa vyolan a lasante, m te pze kesyon avan m te pran l, paske m gen de timoun ki asmatik, e m te tyen ke li p’ap segondè, jiskaprezan m wè sa va, paske timon yo respire byen, mwen menm tou gen de lè m pwoche bò kote l, m pa pran kenn odè ki pa sa.

ITV: Dakò, nou pral pran kesyon 6 la, klase enpòtans avantaj emanatè a bay, n’ap di pou kounya èske gen gwo avantaj oubyen ti avantaj? Èske gen ti avantaj fèb? Ou ap chwazi youn nan repons yo.

HM42:M ta renmen chwazi gwo avantaj la wi.

ITV: Ebyen make l, ou ta renmen chwazi l oubyen ou ap chwazi l?

HM42: Annatandan banm ba ou ti avantaj la jis pou nou ka pote koreksyon pou s ak’ap vin dèyè yo ka vin plis nòmal.

ITV: Trè byen. E pou dezavantaj, èske se dezavantaj fèb oubyen ti dezavantaj, pa two mal, oubyen gwo dezavantaj pa bon menm?

HM42: M pa ka di li pa bon menm. Dezavantaj fèb, pou yo pote ti koreksyon.

ITV: Ebyen, oke. Kounya ou pral site epi dekri nenpòt lòt benefis oswa dezavantaj emanatè a bay. Ou mèt pale, ou pa oblije ekri yo.

HM42: Kòm m di sa déjà, m swete ke teknisyen yo,kòm n’ap viv nan on payi ki ase sal, alò ki trè sal,yo aparèy ki gen on pousantaj pa egzanp a 80 pou san nou nan yon peti ki pwòp, konnen si l vin an ayiti l’ap gen on dezavantaj, menm pwodui ou te sèvi avè l la,pa egzanp pou pou peyi ki pwòp la,ou sèvi avè l an ayiti, avantaj li te bay 90 la, m panse an ayiti l’ap bay 50. Paske pwodui an, lè l, lanse li pa jwenn ensèk la dirèkteman nan pakou l, m panse ke li jwenn lòt enkonvenyan, salte, lafimen, ou konn son peyi ki toujou gen lafimen, fatra k’ap boule. Sa vin fè tou, mwen menm m panse ke, yo ka toujou panse yon etid sou sa, sèke nou te fè on eksperyans avè l pa egzanp sen domeng, nou te mete 70 pou san ke n te jwenn efikasite an ayiti eseye mete 80, 90 pou n wè si n’ap jwenn yon efikasite ki plis.

ITV: Nou prale nan kesyon 8 la, klase enpòtans avanataj, dezavantaj sa y opa youn nan repons ki anba yo,oswa nenpòt nan bagay sa yo,ou avnatj èske ou ap pran avantaj fèb, avantaj modere avantaj enpòtan?

HM42: Avantaj modere.

ITV: E pou dezavnatj ou ap pran dezavantaj fèb, dezavantaj modere, dezavantaj enpòtan?

HM42: Dezavantaj fèb, si mbyen konprann sa ki dezavantaj la, paske li pa fè m mal.

ITV: Kesyon 7 ak 6 la se menm bagay.Kesyon 6 ak 8 la se menm bagay Kòmsi yo mete 6 la, yo tradi l an fransè,se pa grav, nan 9,site ak dekri nenpòt lòt benefis oswa dezavantaj ke emanatè a bay?

HM42: Prèske m wè menm jan avèk 8 la ankò.

ITV: Èske ou gen on bagay pou t’ap ajoute? Sa plis sanble ak 7 la.

HM42: Pa vrèman paske em di sa deja nan 7 la si m gen on bagay ke m t’ap ajoute sèke m di,ke peyi a trè sal si gen on pwodui ke sen domeng te itilize a 70 pou san,an ayiti mete l a 80 pou san, aparèy la pa vize sèlman ensèk la,li jwenn lòt bagay nan wout li, lafimen m di ou pousyè, se sa m di ou.

ITV: Klase enpòtans avantaj ak dezavantaj sa yo, nan repons ki anba yo,oubyen nenpòt nan bagay sa yo,se menm bagay la ankò ki poze.

HM42: Pou avantaj, m toujou pran modere.

ITV: E pou dezavantaj?

HM42: Dezavantaj fèb.

ITV: Mèsi anpil pou ti pale sa, ou bay anpil enfòmasyon ki enpòtan.
