# Supplementary material for: Community end user perceptions of hessian fabric transfluthrin vapour emanators for protecting against mosquitoes under conditions of routine use in Port-au-Prince, Haiti
Source: PLoS One. 2024 Jul 10;19(7):e0300368. doi: 10.1371/journal.pone.0300368 (PMC11236173; doi:10.1371/journal.pone.0300368)
Supplement: S3 Data — (DOCX) [file pone.0300368.s005.docx]

TEAZ Project Haiti – Social Science Data

Transcripts of Focus Groups Discussions (FGDs) and Photovoice Focus Group Discussions (PV-FGDs)

[FGD Men Blocks 1-4 2](#_Toc155781459)

[FGD Women Blocks 1-4 21](#_Toc155781460)

[FGD Men Blocks 5-6 33](#_Toc155781461)

[FGD Women Blocks 5-6 47](#_Toc155781462)

[PV-FGD Men Blocks 5-6 56](#_Toc155781463)

# FGD Men Blocks 1-4

Date: 08/01/19

ITV: Obrillant Damus, principal social sciences investigator

ITV: Alò, m byen kontan avèk nou la maten an la, nou pral kòmanse avèk « Focus group » la maten an, son reyinyon li ye sou itilizasyon emanatè a, alò se pa vrèman kesyon ki te prevwa men se la ansanm n’ap poze kesyon yo, ki pwoblèm nou jwenn nan moustik, ki pwoblèm moustik bay?

HM35: Mwen se HM35 nan blòk 4, moustik se on ensèk ki bay anpil maladi jeneralman pa vwa sangin se konsa nou kontakte li nou menm, men emanatè a jan nou konnen l se on pwodui ki ede konbat kont li, ki se on sibtiti de blakatòks li ye, men sèl pwoblèm ke mwen ka twouve avè l se nan fason li fèt la, sa vle di tuil ansanm avèk jan li ye a, li difisil pou ka mete l an sispansyon pa egzanp depandaman fason ou kouche pou l te ka repann plis nan pyès la, nan pyès kote ou kouche a,se sèl pwoblèm sa mwen menm pèsonèlmamn m twouve avè l paske,nan fòm emanatè a ye kounya si kote ou dòmi an pa rapò avèk kote l ye a, li pi wo ke li donk li difisil pou jwi plènman de avantaj ke li bay la, menm si li repouse moustik yo sèt, men le fè ke pa rapò a kote l ye a, pa rapò ak emanatè a komsi li pa itilize efikasman se sèl sa m twouve kòm enkonvenyan nan emanatè a.

ITV: Li pran twòp plas?

HM35: Non, sou on kabann ou ye, emanatè a ou pa ka mete l an sispansyon, se atè li ye, lè konsa li elwanye moustik yo se vre, men le fè ke li pa paralèlman elve pa rapò avèk kabann nan li pa sèvi jan pou li ta sèvi a.

ITV: An ok, fò l ta menm otè?

HM35: Egzakteman.

ITV: M konprann, ou byen fò ta petèt pi piti pou l te ka mete l on kote bò tèt ou?

HM35: Egzakteman, se sa sèlman.

ITV: Kilès moun ankò k’ap entèveni?

HM1: Bonjou tout moun, mwen se HM1, mwen menm eksperyans ke m fè ak emanatè a paske mwen menm ki m te ka di gen on tèren alèjik avèk pwodui ensektisid yo, menm pafen ke m’ap itilize m toujou trè pridan avèk ki tip de pafen ke m’ap itilize,lè ke pwojè a te vin nan zòn nan blòk 1 epi yo t’ap pale m de pwodui ki ap repouse marengwen,mwen te trè pèplèks pou m pa di ostil,avèk ide a paske m konnen ke ki konsekans ke sa te ka genyen sou sante m pèsonèlman ki asmatik epi lè m kòmanse swiv evolisyon an kijan emanatè a li evolye nan mezon an,premye obsèvasyon ke m te fè sè ke nòmalman depi on odè bagay ki sifokant ta deranje, li konn deklanche alèji m,swa fè nen m brile m, gòj mwen grate m, m pa ka respire, m’santi m’anvi toufe e ot, m pase on jounen avèk emanatè a tout nan enviwònman imedya mwen m pat gen okenn pwoblèm ditou, se premye konsta ke m fè m te ankò gen dout sou esplikasyon ke ou te ban mwen ke l’ap repouse moustik yo, epi mwen suiv dezyèm nivo a, e efektivman, alalong mwen wè ke pat gen moustik ditou ankò nan kay la, eksperyans ke n te fè ki ekstraòdinè, se sa ki pi make m, te gen on basen dlo nan kizin nou, basen dlo sa li te konn favorize anpil moustik nan kizin nan kote l te ye a,e nan tout kay la, epi nou depoze emanatè a efektivman nan espas kote ke basen an te ye a, kote ki te gen anpil marengwen, e nan on tan vrèman rekò nou te wè ke marengwen yo pat la ditou, se obsèvasyon sa ke m fè, premye bagay li pa deranje moun ki gen pwoblèm de sante ki ta ka akoz de pwodui a se t’ap pi gwo pwoblèm ki te ka genyen, si se te on bagay ki te ka deranje moun. moun ki sansib, ki alèji bagay sa yo se pi eksplwa ke m wè ke pwodui a fè sè ke li pa deranje moun o nivo de sante.

ITV: Trè byen, se on bon temwayaj, se byen l’ap pale de eksperyans li fè ak emanatè a men nou poko rive nan faz sa la, paske gen on lòd nan kesyon yo, alò premye kesyon m te poze a sa ki pwoblèm moustik bay, ou reponn, ki pwoblèm nou jwenn nan moustik, ki pwoblèm moustik bay? Sa vle di avan emanatè a vin lakay nou, ki pwoblèm moustik te konn ba nou? Ou bay deja kèk temwayaj, kiyès moun ankò k’ap pale?

HM10: M se HM10 m sòti nan blòk 1, m se yon kolektè nan blòk 1, blòk 1 ki presizeman a debisi mwen di tout zanmi yo bonjou presizeman avèk doktè a ki la, avan ki pwoblèm moustik te konn bay?moustik lan, lè moustik la mòde ou ou konnen, tout moun konnen ki maladi moustik bay e ki pi pwoblèm lè moustik lan vini se chikounkounya, men se toujou menm jan, se toujou menm pwoblèm yo, toujou maladi, malaria, se toujou konsa lè moustik lan mòde ou ki maladi li bay.

ITV: Oke, èske gen benefis ou ka jwenn nan moustik? Èske moustik se on bèt ki bay avantaj oubyen benefis?

HM10: Bon li pa bay ni avantaj ni benefis.

ITV: Oke, avan emanatè a te rive lakay nou ki pwoblèm nou te konn jwenn nan moustik? Ki pwoblèm moustik te konn bay? Kiyès k’ap reponn?

HM14: Bonjou tout moun mwen se HM14, mwen m kwè m soti nan blòk 2, pou byen di nou, moustik la te konn bay anpil maladi,apre dekouvèt yo vin fè, pou byen di ou avan dekouvèt m te konnen moustik la, m pat konnen ki tip de maladi li te konn bay non,menm m konnen moustik la depi li mòde on moun l’ap voye ou lopital kanmenm e se lè ou al lopital doktè ap di ou men tèl maladi ou fè e se tèl bèt ki pike ou, ki ba ou maladi sa, aprè tout etid yo fin fè,yo vin wè ke moustik la li bay filaryoz,li bay chikounkounya, li bay deng, li bay zika, se sak fè m ka di moustik la pa gen okenn benefis ladan l ditou si se pou mwen.

ITV: Alò avan emanatè a te rive lakay nou kisa nou te konn fè pou pwoteje tèt nou kont marengwen? Sa se HM13 nimewo 2, fò ou bay tout non ou.

HM13: Bonjou tout moun, non pa m se HM13, mwen vle reponn avèk premye kesyon an,mwen menm mwen ka di, tout sa Bondye kreye, li gen enpòtans li, il est vrai que lèzòm gendwa poko konn enpòtans li marengwen an, sou plan pozitif la, men sou plan negatif la m konnen l, nou tout konnne l,marengwen son bèt ki bay anpil maladi,mwen menm mwen viktim de li deja, gen anpil maladi m pran deja se marengwen ki ban mwen yo,alò, enpòtans lan, m ta renmen yo fè rechèch la, paske pa gen anyen Bondye kreye sou tè a ki pa gen enpòtans li,se sa m te ka di angwo.

ITV: Ou sonje ki maladi moustik te ba ou? Ou ka bay non yo?

HM13: Chikoungounya, m ba ou youn.

ITV: Chikoungounya se te on epidemi, jiska mentnan e sa rete on menas pandye sou tèt moun,alò ki pwoblèm moustik te konn bay, ki pwoblèm nou jwenn nan moustik e kisa nou te konn fè pou nou konbat moustik ak avan nou te gen emanatè sa rive lakay nou? Avan kisa ou te konn fè pou pwoteje tèt ou kont moustik, ki pwoblèm moustik te konn bay? Kisa ou te konn fè pou rezoud pwoblèm sa yo avan?

HM20b: M sam salye tout moun, m salye doktè a espesyalman.

ITV: Se HM20b pa vre?

HM20b: Wi, se HM20b, blòk 3 je crois, marengwen te konn bay anpil pwoblèm jis si n te kon gen dlo sal, bagay ki konn kenbe dlo sal,on seri de kote bagay sa yo nou sipoze trete yo on seri de lòt jan poutèt pou l pa gen twòp marengwen bò kote nou rete a, m pa pale an tèt mwen, tèt pa m pèsonèlman, m pale an jeneral pou tout moun,donk mwen menm m te konn gen anpil marengwen bò lakay mwen, kote m’ap viv la,men m bat nòmalman tanzantan pou ou ap wè kò m mwen te di bagay ki kenbe moustik yo,nou k’ap bat pou n pa kenbe yo,lè m vin jwenn bagay sa tankou yo vin gen emanatè a, sèl sa m kapab di mwen menm, emanatè a li bon,men se pou tretman pou yo ta met ladan li plis,pou l kapab pi efikas paske moustik yo, nòmalman si n genyen li se pou l pouse moustik men fò l kapab genyen plis efikas tretman e sa m te kapab di pou n kapab plis pouse marengwen yo.

ITV: Dakò, men avan emanatè a te rive lakay ou kisa ou te konn fè pou kori dèyè moustik sa yo, oubyen pou touye yo?

HM20b: Wi pa egzanp lè genyen yo,moun yo konn gen, gen moun ki gen moustikè,menm si moun ki pa genyen, yo fè yon jan kanmenm menm si se pa san pou san yo bat pou marengwen pa pike yo,se sa m te ka di.

HM30: Non pa m se HM30 nan blòk 3 pou m fè suit avèk sa mesye a t’ap di a pou n ta pwoteje tèt nou kont marengwen yo, nou te itilize blakatòks avèk begond pou n ka pwoteje tèt nou avèk marengwen se sa m t’ap di.

ITV: Èske blakatòks la ak begond konn bay ti pwoblèm sou sante nou?

HM30: Menm si li pa byen efikas men pou ou byen dòmi ou sipoze itilize l menm si li pa bon ou fè l kanmenm, mèsi.

HM26: Mwen non m se HM26 blòk 3, nou te konn itilize balakatòks avèk moustikè, epi m te kon pase losyon sou po m tou.

ITV: Losyon wè.

HM26: Men on sèl bagay, losyon an konn fè boul sou po m.

ITV: Ou alèji avè l?

HM26: M alèji avè l, apre lè emanatè a m ta swete yo mete plis pwodui ladan pou l pi efikas.

ITV: Pou l pi efikas?

HM26: Wi.

ITV: Trè byen. Mako vle pale men n’ap pran mpoun ki poko pale ditou, n’ap pran mesye sa ki deja, ki te ka papa m.

X: Premye fwa m te konn sèvi ak blakatòks, depi m vin genyen l lan, m pa sèvi ak blakatòks ankò, m sèvi avè l, avan tou lè m pat ko genyen l moustik mòde m tout kote nan kò m, ou konprann men depi m genyen l lan, moustik pa mòde m ankò, leswa lè m’ap dòmi m’ap bay figi m kalòt, men kounya m pa bay figi m kalòt ankò. Men sèlman m wè l on ti jan vin fèb.

ITV: Wè efikasite a redui, oke sa se HM11?

HM6: Bonswa m se HM6 nan blòk 1, sa m te konn itilize se blakatòks mwen menm pouvi ke,m pa t ka pran odè balaktòks la, lè fini tou nou konn boule po zoranj, nou konn met po zoranj boule pou n ka repouse marengwen yo, men li pat kòmsi osi efikas ke emanatè a.

ITV: Se yon bagay natirèl, po zoranj, men li pa vrèman efikas.

HM6: Wi. Oke mèsi.

ITV: Kiyès ankò ki ap pale paske pawòl la se pou nou li ye, fòk nou di l tout moun gen dwa la pou pale, se HM18?

HM18: Non pa m se HM18, mwen menm lè ke m te konn itilize balakatòks sa vle di li fè on ti kouri dèyè l men pa vrèman pou n te ka itilize l men emanatè a tankou pa egzanp kounya m’ap gade l sou twazyèm etap ki genyen an m wè l pa kouri dèyè marengwen yo vre, twazyèm etap sa.

ITV: Efikasite a redui?

HM18: Twazyèm etap sa kòmsi marengwen yo toujou ap fè..

ITV: Aprè konbyen tan ou konsatate sa? Aprè de mwa, on mwa?

HM18: Aprè on mwa,paske se twazyèm etap la nou pran la wi.

ITV: Efikasite a redui?

HM18: Se sa sèlman wi.

ITV: Bon, chèf sa poko pale non, èske nou gen on bavay n’ap di? Avan ou te gen emanatè a lakay ou ki pwoblèm ou te konn jwenn nan moustik? Ki pwoblèm moustik te konn bay e kisa ou konn fè pou rezoud pwoblèm sa yo, pou konbat moustik avan ou te gen emanatè a lakay ou?

HM44: Mwen se HM44, avan emanatè a te lakay mwen, se te jan blakatòks sèlman paske begond lan li on ti jan plis, paske ou bezwen itilize yon pwodui pou ka rete nan milye a,sètadi si ou gen on chanm ou flite on begond ladan l, lojikman ou p’ap ka ret anndan paske you toujou tyen aske ou fèmen pòt la, ou soti pou l ka fè on efè,men evantyèlman blakatòks la yo konn itilize l nan sans ke ou chita sou on chèz epi li ka distans soulye dam nan epi,men pwoblèm mwen menm m gen avè l, m klostwofòb anplis mwen asmatik sètadi ke lafimen sa m pa sipoze pase prè l paske m vin pa ka respire byen nòmalman, m vin pa itilize l ditou,m oblije tolere marengwen ap mòde m’ maten, midi, swa, kounya vin koz lè enanatè a vini nou vin itilize l men, pwoblèm mwen gen avè l se maj defikasite a li redui entèval de jou, aprè de jou, twa jou li kòmanse diminye, limit 5 jou kòmsi li te jis yon, li jis gen on aspè dekoratif, ryen de plis.

ITV: M konprann, ou te di sa deja nan on antretyen.

HM44: Sètadi ke sa m ta swete yo plis fè se,menm jan avèk yon kòlèg te di talè a, premyèman ou ka kouche se vre,men lè l kòmanse diminye konsa, ou oblije avanse l pi prè ou,men m pa ta gen pwoblèm ke li diminye maj la vin bese men ou ou vin pa ka apwoche l, imajine ou mouche soub on kabann epi li atè a, li twò gwo ou pa ka di m ou ka mete l tankou si kabann nan gen on ti, kèlkeswa bagay ou te ka mete l la paske gen li twò go, lojikman si n ta bezwen kite l gwosè sa,nou ta redui ladan l epi mete plis pwodui epi pou maj defikasite a ka plis elve.

ITV: Minyatireze l, m konprann,on bèl temwayaj wi ou bay la, epi ou pat anvi pale, kilès ankò n’ap pran zanmi sa ki sa mekanisyen.

HM34: M se HM34 blòk 4, kamarad yo site plizyè bagay ke yo konn itilize pou yo kapab fè prevansyon kont marengwen men genyen, pafwa gen moun tou ki konn pa egzanp lakay mwenm, m te konn itlize vantilatè tou kont marengwen yo,m te konn itilize on seri de pwodui ke yo vann nan sipèmache, nan makèt ke ou kapab itilize kont marengwen an, men mwen menm mwen te fè, m gen pwoblèm avèk, sitou blakatòks la,paske m pa kapab respite li byen, lè emanatè a te vin lakay mwen pèsonèlman li te banm bon rezilta pandan on semèn, ou pa bezwen an manyen emanatè a ditou ou jis mete l anndan chanm nan sèlman epi ou gen lapè,men aprè on semèn ou oblije de tanzantan ap avanse l plis vè oumenm, transflitrin an ke yo te enjekte ladan l nan li ta sipoze gen on dire plis ke sa,mwen pa knnen si se kantite transflitrin yo mete a ki pa sifizan, mwen pa konnen tou si se an tèm de emanatè a ki on tijan twò gwo ki pran twòp espas tou ki vin fè ke pa egzanp si ou gen kabann ki wo li bay pwoblèm,si ou gen on kabann ki pi ba li pa deranje.

ITV: M konprann.

HM34: Ou wè sa m di a, sa vle di si m gen on kabann ki pi ba, m mete emanatè a tou pre l lan, lè sa pa gen maregwen k’ap pase bò zòrèy ou,men lè pa egzanp emanatè gen fèb entansite si ou gon on kabann ki pi wo, kòmsi ou pat bezwen emanatè a.

ITV: Avèk tan, plis li pi pre ou, li pi bon,plis li lwen ou li mwens efikas.

HM34: Wi, sa vle di ke fò n reflechi sou sa,m pa konnen kòman n’ap wè,kòman n’ap kapab fè li, epi lòt bagay ke m toujou panse tou, m panse ke moun k’ap itilize emanatè yo tou ta sipoze gen aksè a transflitrin nan tou pou yo te ka,de manyè ke pou yo te ka enjekte l ladan l ankò.

ITV: Poukont yo?

HM34: Wi, imajine aprè on semèn li bay rezilta epi lòt semèn, li bese nan entansite l,sa vle di ke m te kapab rebagay li pou l kapab banm plis efikasite.

ITV: Trè byen, sa vle di ede moun yo trete emanatè a poukont yo.

HM34: Natirèlman.

ITV: Bèl temwanyaj, kiyèk ankò k’ap pale pou n ka pase nan lòt faz kesyon, èske ou gen emanatè lakay ou?

ITV: Ebyen, n’ap pase a on lòt kesyon, bon se kanmenm pa mal paske pwodui ki gen kounya yo, menm lè yo di ou l’ap dire uitèdtan se manti, se katrèdtan, yo double l,si ou gen on bagay deja pou yon semèn ki plis efikas m panse se trè, trè bon, m panse ke gen mwayen ou ranfòse efikasite l, epi sa ap vini. Nan sa nou te konn itilize deja yo, m konnen gen moun ki gentan reponn, ki limit produi antimoustik, ki limit pwodui nou konn itilize kont marengwen yo genyen? An tèm defikasite sètadi èske genyen ki pi bon ke lòt, genyen ki mwens bon etsetera, bay limit mwayen sa yo nou te konn itilize pou pwoteje tèt nou kont marengwen, si se ventilatè, si se blakatòks, gen moun la ki reponn deja, moun ki poko pale ditou, m ta renmen yo pale pou yo bay limit oubyen move kote teknik oubyen mwayen nou te konn itilize avan pou n pwoteje tèt nou kont moustik avan emanatè a te rive lakay nou.

HM10: Avan emanatè a te rive lakay la, nou te konn itilize begond avèk balakatòks.

ITV: Ki limit bagay sa yo?

HM10: Limit blakatòks la, sa k pa bon ladan ki se toujou odè, odè an ki pa bon, sitou mwen menm ki te gen on tibebe a lepòk odè an konn pa bon pou li, de menm ke begond lan tou, men lè ke nou vin gen emanatè an, m sonje lè emantè a premye semèn emanatè an te vini li te pi trete, odè an te pi frape, paske m sonje madan m nan te di HM10 kisa, yo pat konn sak emanatè an paske aprè kesyon li vin poze yo, m pale l’, m pral di l men sa k la, men sak emanatè an.

ITV: Wi.

HM10: Li di HM10 kisa ki santi fò la, m di sak santi fò, m pa wè sa non, m’ap chache m wè se emanatè a, jan m te esplike ou talè an, lè l te fèk vini, men kounya la, li pa vin banm menm odè sa, e li te pi efikas lè sa.

ITV2: Odè emanatè a te bay pa t deranje ou?

HM10: Non, odè a pa t deranje m, men pa rapò avèk bebe a m pat vle li twò pre bebe a, paske madanm mwen te di m retire l pre timoun nan, m nèk ekate l.

ITV: E blakatòks la, ou pa t ka pran sant li, èske li te konn chase moustik vre?

HM10: Bon, m p’ap mete l a san pou san non,paske m knn ap gade balaktòks la la ap degaje chalè, epi moustik ap vole.

ITV2: A konbyen pou san ou ka mete l?

HM10: M ka mete l’a on swasant pou san.

ITV: E begond lan?

HM10: Begond lan konn efikas vrèman men se odè a, lè n flite begond lan se soti nou oblije soti nan kay la,se bay kay la, se apre nou fini, nou ouvè pòt kite lè pase epi nou rantre.

ITV: On lòt kesyon limit efikasite,oubyen pwoblèm mwayen nou te konn itilize avan pou pwoteje tèt nou kont moustik, di ki pwoblèm, ki limit pwodui sa yo te konn prezante pou nou?

HM1: Se HM1 blòk 1, anfèt ansanm de bagay nou konn itilize kont moustik yo, si n ta pran le ka de vantilatè a limit sè ke fòk gen kouran pou itilize l e n’ap viv nan yon peyi kote ki pa vrèman gen elektrisite,ou ap veye kilè yo bay kouran pou kapab limen ventilatè pou rantre nan chanm ou pou dòmi, sa se premye limit lan, dezyèm limit lan ke ventilatè a genyen lè ou ap dòmi ak on ventilatè sou oumenm li ka ba ou pwoblèm de sante, sa vle pwoblèm nan poumon, ki gripe, li ka ba ou pwoblèm bwonch sa vle di lip am toujou rekòmande pou dòmi avèk on ventilatè sou ou,gen moun ki dèyè astis ki pa fikse l sou yo menm lè sa ankò, on ventilatè pa rekòmande, pa rapò avèk blakatòks la dire l trè kout, met blakatòks an on chanm on kadè ou wè li tounen sann, li pa de long dire vrèman,e anplis de sa, ou pa ka vrèman mete l prè ou, paske ou pa ka pran sant li, m p’ap menm pran le ka pou on moun ki asmatik men odè li pa twò agreyab, m konn itilize fimigasyon,kòmsi pwodui natirèl pou chase marengwen, sè ke lè ou mte l pa egzank se nan on rech dife moun nan pran li mete swa po zoranj, oubyen grenn maskreti, lè ou mete li se bay espas la,se fèmen pòt pandan on bon bout tan e aprè, lè marengwen yo soti pou fèmen pòt la ankò,paske si ou reouvri l lè lafimen an ale,si marengwen rantre ankò ou gen menm pwoblèm nan ankò,sa vle chase marengwen nan espas la fèmen pòt la,pa reouvri l ankò, pou marengwen pa reantre, si ou reouvri pòt la ankò se kòmsi fimigasyon ou te fè a pa gen okenn itilite,sa se limitasyon l, e dire tou on fimigasyon konsa dire li pa long, alòske emanatè a li ale pi long dire, li fè plis tan,m panse ke li trè efikas nan sans sa.

ITV: Mèsi HM1, a chak fwa pa bliye bay non nou lè n’ap pale.

HM20b: M ta renmen konnen emanatè a pwodui yo mete ladan li an, kisa li ka fè sou sante ou, si pou mete l pi lwen ou pi pre?

ITV: Pwodui yo mete ladan l lan, se on pwodui yo ap itilize depi ‘des dizaines d’années’, yo wè li pa bay okenn pwoblèm sou sante moun, se a yo ekri nan pwotokòl rechèch la.

HM20b: Paske jan nou tout knnen li, pou l pouse moustik la,ou apwoche li kabannn nan oubyen pi, pi ba, paske oumenm se solisyon n’ap chèche, si pa egzanp ou gen emanatè a ou byen ou ap apwoche l bò tèt ou,paske se sa m’ te bezwen konnen pa rapò a pwodui n’ap mande a.

ITV: Se manyen pou ou pa manyen l, se sak fè yo di ou, gen on kote pou manyen l.

HM20b: Dakò, se kesyon sa m te genyen.

HM13: M gen on kesyon, wòl emanatè a, pwodui yo mete ladan l lan, èske se pou pouse marengwen oubyen pou atire marengwen yo ye?

ITV: Se sa k fè yo di ou, amanatè a, li gen on propriyete repilzif, li la pou repouse,chase marengwen, pou fè l pati pou l pa vin mòde ou,pou l chase anvayisè a, moustik la pou l pa vini, li fè l rete lwen, se sa li genyen, li pa touye moustik la, men li fè moustik la pa vin mòde ou.

HM13: Kesyon an te gen on swivi dèyè l, nan moman n’ap fè kapti moustik yo, emanatè a toujou la, m wè nou toujou pran moustik, alò, èske li pouyse yo a san pou san, oubyen gen on to ke l repouse marengwen yo?

ITV: Ebyen se on bagay eksperimantal, li pa gen efikasite,maksimal a san pou san, li repouse men se pa a san pou san, se sa k fè li bezwen pou yo amalyore l.

HM44: Se HM44 de blòk twa, lè ou pale de dire, si pa egzanp m pran blakatòks la, premyèman li chase marengwen yo se vre, men pou m byen di ou, m pa konn dire l, imajine ou limen li kounya la, nan dis a kenz minit aprè m etenn li, paske m pa ka respire men mwen fè on konsta, sè ke otomatiklman ou etenn li, se otomatikman marengwen yo pase ankò, ou etenn li pou 1, ou gen on avyon ki pase bò zòrèy ou ap fwing, dezyèm choz lè ou pran ka de begond lan, begond lan,egzanp ou pran n chanm ki èmetik.

ITV: Ki fèmen?

HM44: Wi, ou flite begond lan ladan l,la li pa gen okenn espès ‘d’air’ eksteryè ki vini pou ayere anndan,l’ap toujou rete anndan,men otomatikman ou gen on fenèt..

ITV: Lè ou rete anndan li p’ap bon pou ou.

HM44: Li p’ap bon pou ou men, se ou menm li repouse ou,li repuse moustik la tou, tandike kay la se pou ou li ye.

ITV: Li pa konvivyal.

HM44: Non, sètadi ke, lè ou pran begond lan, lè ou ouvè on fenèt, oubyen gen vantilatè, ou konekte vantilatè ou, li repouse pwodui, li repuse efè li te ka fè a, otamatikman tout begond lan fin ale, ou tande ou gen kolokatè anndan kay la avè ou.

ITV: Bèl mo, kolokatè paske fòk yo bò kote ou, fòk yo viv avè ou,men balakatòks la limenm li repouse moustik la men anmenm tan li repuse ou tou,paske ou anndan on lafimen anndan kay la,li repouse ou, ou vin pa ka pran sant lan, ou gen de chwa swa ou etenn li epi ou kite moustik la mòde ou, swa ou kite l anndan epi ou soti ou al mache epi lè ou tounen,ou p’ap jwenn kèk grenn moustik apre on sèten nonb de tan.

ITV: Mesye ap byen panse wi, bravo. HM3 vle pale men HM3 ap gen demen se sak fè m p ata renmen li pale kounya paske se gason yo k’ap pale.

HM3: Se pa rapò ak kesyon HM13 te poze talè a, ou reponn li men li manke on ti diplis, li te poze kesyon kijan fè lè y’ap fè kapti moustik yo, kolektè yo toujoun pran moustik?

ITV: Wi, se sa m pa konn sa byen, petèt oumenm mi sipèvizè u ka reponn pi byen pase m.

HM3: Wi, lè kolektè a limenm l’ap fè kapti li jwenn moustik, enanatè ki sou teren yo, se pa limenm ki nan fwaye yo,emanatè ke yo remèt fwaye yo, se emanatè ki deja trete,ò sou teren nou fè etid sou emanatè yo tou,noun gen de tip emanatè sou teren, nou gen emanatè de kontwol, nou gen emanatè ki trete tou.

ITV: Dakò,trè byen.

HM3: Li nòmal, on kolektè ka jwenn moustik, li ka pa jwenn tou,depandaman de ki amanatè ki nan men li.

ITV: Dakò si li pa trete …

HM3: L’ap jwenn moustik.

ITV: Si li pa trete se moustik ki ap vini.

HM3: Wi, se sa.

ITV: Mèsi pou presizyon sa, ebyen m ka kontinye ak kesyon yo, si nou sonje pwodui nou te konn itilize avan yo,avan emanatè a te lakay nou, bay non pwodui sa yo, epi di limit yo, bay limit yo,bay avantaj yo, si genyen ankò,nou sonje, nou bay pou begond blakatòks etsetera.

HM35: Se HM35 blòk kat, nan bagay nou konn abitye itilize pou n kouri dèyè moustik, gen on distenksyon ke mwen menm m pa two fè, tankou blakatòks la kouri dèyè moustik la, tandiske begond lan li gen on karaktè definitif li konn touye l.

ITV: Wi, se vre.

HM35: Sa vle di se diferans sa m wè ant plizyè kategri se sa n itilize yo, gen bagay nou trete nou kouri dèyè moustik la nou jis elwaye l,ansuit lè bagay yo fini pou l tounen, tandiske begond lan li plis gen on aspè definitif li touye l nèt.

ITV: Blakatòks la plis repilsif, begond lan alafwa son ensektisid li touye epi li repilsif tou.

HM35: Ekzakteman.

HM1: Se Mako, blòk 1, se vre ke begond lan gen pou objektif touye moustik yo, men konsta ke nou fè dènye ane yo, pwodui sa yo sè ke yo mwens efikas ke yo te ye avan, begond yo.

ITV: Moustik yo vin adapte yo.

HM1: Egzakteman, nou gen on jenerasyon moustik m te ka di ki vin rezistan pa rapò a pwodui sa yo, ki fè ke menm lè ou gen begond lan, li plis deranje oumenm ki sansib a pwodui a ke moustik la limenm ki gentan adapte li a pwodui sa yo ‘au fil des années’

ITV: Li fè ou plis mal ke limenm.

HM1: Wi, pase moustik la paske yo menm yo plis rezistan a pwodui sa yo.

ITV: On bèl temwanyaj wi.

HM34: Gen rakèt yo.

ITV: Rakèt elektrik?

HM34: Wi, sa vle di yo fè kaptaj moustik yo, yo touye yo tou, yo menm gen moun ki itilize yo, genyen yon sòt se ti aparèy ki fnksyone sou fòm de kouran, ou konekte l nan chanm ou, epi ki atire moustik lan, ki touye l tou, sa vle di ke yo vann sa yo nan makèt la, m konn wè yo.

ITV: Ou itilize yo deja?

HM34: Bon, mwen gen on moun mwen ki itilize l, se pa mwen dirèkteman, paske moun nan gen on anviwònman moustik, sa vle lè m’al lakay li, yo te konn bal pwoblèm, li achte l pou l kapab fonksyone limenm, sa vle di li bay rezilta tou.

ITV: Rakèt elektrik wi.

HM34: Men kounya an tèm de jan y’ap poze kesyon, depi ou gen kouran, ou pa gen pwoblèm, menm jan avèk ventilatè a tou, depi ou gen kouran ou pa gen pwoblèm.

HM1: Rakèt la trè efikas, li boule yo men sof ke, imajine on moun ki sot travay on jounen fatigan, pou on rakèt nan men ou, ou ap fè espò.

HM30: Lè ou touye on marengwen se 20 ki vini wi, nan rakèt la, se Sowèl sou blòk twa, se menm jan ak emanatè a lè l fèk vini li pouse marengwen yo, apresa marengwen yo anvayi. On moun pa ka bay tèt li manti, pou l ta knnen konten tan blakatòks la dire.

ITV: Fò n ta evalye l.

HM30: Li p’ap kapab, paske se on bagay yo fè, se dòmi ou bezwen dòmi anpè, ou pa vle tande bri marengwen an, ou gendwa flite l apresa ou sot nan chanm nan apresa ou vin dòmi, tan ou ap dòmi an ou konn ki marengwen ou ap rele?

HM44: Se HM44 blòk twa, ann pran pa egzanp vantilatè an, ou ap jwenn gen kote vantilatè a fò ou ta fikse l sou ou paske,gen kote ki tèlman gen moustik otomatkman vantilatè a vire ou santi gen youn ki pase bò kote ou,lè l tounen sou ou ankò, ou pa jwenn li, ann pran egzanp ou fikse l sou ou menm, men moun nna ou pa sipoze fikse l sou ou, li ka koz kèlkeswa maladi ki lye avèk twòp imidite, sa vle di ke li sipoze ayere tout chanm nan, men valè moustik mi genyen an, vantilatè a gendwa pa ka fè anyen pou li e kesyon m ta renmen poze paske jan on mesye te di l talè an, ou konn limen n blakatòks yo pa mòde ou, ou pas anti l se vre men ou konn chita,pa kiryozite epi pou wè youn k’ap pase m ta renmen konnen èske blakatòks la se tiye li tiye oubyen li repouse l’, oubyen se anpeche li anpeche li mòde ou?

ITV: M panse se repose li repouse l.Si li repouse l, sa vle di li anpeche li mòde ou. M’ap poze ou on kesyon, èske pwodui anti moustik yo, èske lè nou pral achte youn, èske nou met nan tèt nou, sa pral ede nou dòmi oubyen viv pi byen, èske nou itilize l pou pwoteje tèt nou kont maladi, èske nou toujou konsyan ke moustik bay maladi e ke li enpètan pou nou itilize pwodui anti moustik?

HM1: M panse ke, premye reflèks anpil fwa ou bezwen dòmi, ou fatige bèt la ap ba ou on chan sonèt li nwizib ou bezwen trankilite,men apresa lè ou ap vin aprann maladi ke li bay, ou ap tande ke pa egzanp moun ki malad, ki gen zika, mi gen deng ou tande ke se akoz moustik lè sa ou vin gen on lòt reflèks de pwoteksyon ou sante men premye reflèks oumenm ki ap itilize pwodui sa yo, se paske ou bezwen on trankilite, ou bezwen lapè ou avèk bèt la, paske li vrèman nuizib,apresa lòt nivo a, men gen bagay ki enpòtan m te vle ajoute se konsènan efikasite emanatè a, si ou ap itlize emanatè a pa egzanp, epi ou pa fè dot prevanasyon, dot aranjman nan anviwònman,paske m te esplike talè a m te gen on basen nan kizin lakay mwen ki t’ap fè ke marengwen yo egziste plis, otomatikman tou, m’ap itilize emanatè a, m vin krazde basen sa tou, premye bagay se ke sa k’ap pwodui marengwen an li pa egziste ankò,e si bò lakay ou pa egzanp gen rigòl,gen fatra se asire ou avan ke anvowònman ou pwòp,epi lè ou depoze emanatè a lè sa ou mye evalye rezilta ke li bay e sa, si anviwònman gentan pa otòp,menm si ou ap itilize emanatè a ou ka pa ka byen evalye efikasite a tou fò ou asire ou ke tout sa ke ou dwe fè nan anviwònman ou fè li epi kounyeya ou ap itilize emanatè a tou.

ITV: Se yon bèl kòmantè, sa vle di lit chimik lan, pa sifi, men fòk ou chanje konpòteman ou tou, sitou anvè anviwònman pa lage dlo nenpòt kote, eseye dekouvri epi elimine kote moustik yo ap repwodui yo.

HM1: Li pa obligatwa pou m di fò m kite gwo basen k’ap banm marengwen an, e pou m di ke piske m gen emanatè pou m gad si emanatè a bon tout bon,m’ap kite on basen k’ap banm moustik se lè sa m’ap konnen èske li bon.

ITV: Fòk nou chanje konpòteman nou tou.

HM1: Egzakteman.

ITV: Nan on sans ede emanatè a, bon kòmantè.Nou prale nan kesyon 2 a, se 3 kesyon ki genyen, se kesyon nou reponn deja nou antisipe, ki avantaj emanatè a bay? Benefis nou jwenn ladan depi nou itilize l?

HM34: HM34 blòk 4, premye avantaj emanatè a se avantaj ekonomik la, lè ou gen emanatè a lakay ou gen on seri de depans ou p’ap al fè, ou pa nan achte balakatòks, ou p’ap veye kouran, gen on seri de bagay ou pa bezwen fè, gen avantaj sa, men lòt avantaj ke li bay tou se emanatè a bay posibilite pou ou kapab mouv, pa egzanp, lè ou pran begond lan nan on sal a manje ou fin flite begond lan ou pa ka manje apresa, sa vle di ke ou oblije jwenn on espas de tan pou òganize sal a manje ou, alòske lè ou gen emanatè ou konnen moustik nan sal a manje ou, ou jis depoze emanatè a epi sava, ou sou galri lakay ou, ou nan lakou lakay ou, kèlkeswa espas ke ou ap itlize a ou konn genyen moustik nan zòn nan epi ou itilize emanatè a, jan kamarad la sot di a se vre li pa sifi sèlman, emanatè a p’ap ede ou sèlman fòk ou fè on lòt travay tou nan anviwònman ou, paske lè ou ede anviwònman ou evolye avèk emanatè a k’ap ede ou li pi bon pou ou, lè sa ou pwoteje vwazen ou lan se pa pwoteje tèt ou sèlman ou pwoteje vwazen ou lan tou.

ITV: Mèsi HM34, ki avantaj, ki benefis emanatè a bay, HM34 byen reponn kiyès moun ankò ki ap konplete ou byen ki ap di lòt bagay, youn nan moun ki plis pale la, ki di anpil bagay se HM1.

HM1: M vrèman apresye repons HM34 la, aspè ekonomik lan li gen plizyè pòte, non sèlman ou pa depanse kòb pou ou al achte pwodui kont moustik yo, men tou anpil fwa nu gen reflèks an ayiti tou pou, olye ke nou envesti nan lasante nou envesti nan lamaladi, gen moun ki depoze tèt yo kay doktè, li toujou malad, l’ap depanse kòb kay doktè.

ITV: Se sak fè yo di prevni vo mye ke geri.

HM1: Egzakteman. Olye ke ou al depanse kòb la lopital, lè sa ou gentan konnen ou p’ap al malad, pwoblèm moustik la ka bay tankou malarya, deng, ou al depanse kòb kay doktè, mwen m gentan fè prevansyon pou m pa malad, sa se on avantaj, ki vrèman ekstraòdinè.

ITV: Emanatè a se on outl de prevansyon.

HM10: M se Jodee HM10 se toujou blòk 1, ki avantaj emanatè a bay, emanatè a fè m fè on pakèt ekonomi, paske m konn ap desann ka briko al achte dè pwodui sa nou sot pale yo, m pa fè sa ankò.

ITV: Kay briko?

HM10: Kay abriko, se la m konn plis desann al achte pwodui, m vin bliye zòn sa akoz de amanatè a.

ITV: Pandan n’ap bay avantaj, nou ka fè on antitèz tou, nou bay dezavantaj, enkonvenyan. Ki enkonvenyan Mako ou wè emanatè a bay?

HM1: Banm reflechi touju.

HM10: Dezavantaj li genyen, jan m sot di tale a, ou konnen timoun dezòd, dèfwa li konn pike l, Ou konnen se on fè li ye.

ITV: Ou te di sa deja.

HM10: M toujou ap evite poun timoun nan avèk emanatè a, gen on lè li te grafonyen li menm, m toujou ape vite pou li pa two kole sou emanatè a.

ITV: Dakò.

HM11: M se HM11 nan blòk 1, apre pike li konn piki a tou, li gen on lòt dezavantaj, sèke,li twò gwo, li okipe twòp espas, si l te ka on ti jan pi piti li t’ap pi bon.

ITV: Minyatirize l?

HM11: Wi.

HM1: Nou plis itilize l nan salon, m panse si l te plis estetik li t’ap plis enteresan, lè ou mete li kòmsi an on espas, ou ap itilize li ou ap resevwa moun, plis li bèl, epi lè ou ap pale moun nan de byenfè li, epi se on bagay ki bèl, lè sa ou ka mete l kèlkeswa kote, ou pa bezwen kache l dèyè on chèz, ou mete l on kote, si l te on bagay dekoratif epi ou mete l, non sèlman l’ap ba ou rezilta, m panse fò nou ta travay sou sa tou.

ITV: Mèsi HM1, avantaj, enkonvenyan, toujou prezante nou avan nou pale.

HM13b: Non pa m se HM13b, m’ap fè on ti entèvansyon sou sa kòlèg la te fè a, sou aspè ekonomik lan, pou mwen menm m p’ap kouri di aspè ekonomik lan paske, dayè pwodui yo mete nan emanatè a nou pa konn, mwen menm m pa konn non l, m pa konn konbyen li vann, pou le moman nou kapab di ke aspè ekonmik lan li bon, paske nou jwenn li gratis, nou pa depanse pou li, la nou kapab di sa, men si nou ta vin jwenn ke pwodui sa vann egzanp wi, ven mil dola ameriken, sa vle di kounya, aspè ekonomik lan li bon kounya? Pou mwen menm non, avnatj m ka di sè ke, gen de maladi emanatè a anpeche nou jwenn li, a travè moustik yo, se pi gwo avantaj li ye, pou mwenm menm.

HM1: Nan kad pwojè, m pa al pran l nan sans jeneral si m dwe achte pwodui a, men an kad pwojè a, li gen on aspè ekonomik pou mwen paske m pa depanse on gous mwen menm.

ITV: E sa y’ap itilize ldan l lan se non bagay ki koute zewo kenz santim konsa.

HM1: M pa depanse zewo kenz santim nan mwen menm, li ekonomik pou mwen.

HM35: Youn nan kesyon m ta renmen poze, m pa janm satisfè de repons yo ban mwen deja yo, se transflitrin nan kisa li ye?

ITV: Se on ensektisid, yon pwodui pou touye oubyen pou chase moustik.

HM35: Wi, men, se paske moun ki chwazi transflitrin nan pou kisa se li li chwazi pa rapò a lòt ki avantaj ke li genyen pa rapò a lòt yo?

ITV: Ebyen lòt yo mwen sefikas, genyen ki make pa egzanp, uitè defikasite poutan se pa vre, li bay dezè ou twazèdtan apresa li pa fè anyen, epi gen lòt tou ki bay pwoblèm sante sa limenm y’ap itilize l depi ‘des dizaines d’années’e yo di li pa gen okenn pwoblèm su sante, gen kontwòl ki fèt.

HM35: Oke.

HM13: Non pa m se HM13, ajan nan blòk 1, m ta renmen pwodui sa nou aspèje emanatè emanatè a, èske li p’ap pwodui on lòt maladi pou nou aprè, se vre dèyè marengwen pou nou, men èske li p’ap pwodui on lòt maladi sou nou?

ITV: Sa se on bèl kesyon, jan m di l ankò, gen on pwotokòl rechèch entènasyonal gen kiskeya, gen inivèsite tanzani, angletè, etazini, pwodui yo met ladan li an, se on pwodui y’ap itilize depi ‘des dizaines d’années e yo di li pa gen kenn enpak sou sante nou, se sa mwen wè nan pwotokòl la, ou pa gen okenn enkyetid, se efikasite y’ap chèche.

HM13 1: Oke.

ITV: Avantaj, dezavantaj emanatè a, toujou, sinon n’ap pran on ti poz, pandan poz la nou ka gen ide n’ap vin disklite yo la.

[POZ]

ITV: Nou pral kontinye nan kesyon 3, èske emanatè a pwoteje nou kont piki moustik? Anpeche moustik mòde nou, nan ki mezi nou wè sa? Èske gen on moman moustik pat mòde nou ditou, èske moustik vin mòde nou apre, eseye di nan ki mezi nou wè emanatè a efikas, e si gen lòt avantaj nou konnen emanatè a bay, pa egzanp nou te bay avantaj ekonomik se trè byen men ka gen lòt avantaj, nou konnen, donk eseye bay yo,lòt avantaj,èske emenatè a pwoteje nou kont moustik toutbon? Ki limit paske emanatè a pa pwoteje nou a san pou san, gen on limit nan pwoteksyon an, bay limit pwoteksyon emanatè, e pou enkonvenyan nou bay enkonvenyan men èske pa gen lòt nou sonje? Eseye bay yo, m’ap tande.

ITV: Dakò ki konsta nou fè ankò? Limit pwoteksyon emanatè a, ki lòt pwoblèm li bay, avantaj li bay, sa nou sonje.

HM1: Pa rapò ak kesyon limitasyon emanatè ak pwdi ki ladann lan,gen mon ki di konsta ke yo fè otan ke jou ap pase, yo wè ke pwodui a, yo wè ke efikasite a li diminye men kesyon ke m genyen,ou pral oblije ogamante kantite pwodui a pou l ka gen on pi long dire,èske li pa pral koze on lòt pwoblèm o nivo de sante,paske se si ke tout pwodui swa ou ap inale, ap konsome, li gen on doz ki dwe, sa pral pale de ovèdoz si ou al oblije mete plis pwodui pou on dire ki pi long, èske kounya kantite yo pral mete a, pa pral gen pwoblèm sou sante moun?

ITV: Fò n ta panse lè efikasite a redui nèt,pou ta retrete l ankò, pou n pa mete twòp depase.

HM1: Fòk ta gen on limit.

HM10: Se HM10, se toujou blòk 1, kolektè nan zòn 1, ou te pale de efikasite emanatè a, sa m remake ak emanatè a dè fwa lè m pran, m konn sòti deyò galri a, m konn al chita kote moustik konn anpeche m chita,tanznatan map plap,plap nan pye m, lè m vin itilize emanatè a, lè m mete emanatè a pa gen moustik ki vin mòde m deyò a ankò, m wè emanatè a vin banm anpil bakòp.

ITV: Èske nou gen abitid mete l deyò emanatè a, èske lè nou mete l deyò a li anpeche marengwen mòde nou?

HM10: Wi, se sa m sot di ou.

ITV: Ou sot di sa men èske gen lòt moun k’ap temwaye sou sa?

HM13: Non pa m se HM13 blòk 2, sitou pou mwen mwen menm m fè refrijerasyon, klimatizasyon, tankou m travay lakay mwen, lè on kliyan banm aparèy pou m ranje, m gendwa ap ranje on frijidè la, m met aparèy la bò kote m, m gendwa fè nenpòt inèdtan ap ranje frijidè a la m pa wè marengwen, men apre on ti moman m kite l deyò a m fè on antre anndan lakay mwen, se kòmsi yo kite deyò a, yo al anndan kay la.

ITV: Yo swiv ou?

HM13: Wi, kounya lè m mete emanatè a anndan, yo kite kay la,men pou byen di ou, emanatè bagay pou on nonb de tan, nou konn sa tou wi, men on kesyon m te gen pou m poze,èske pwojè a kontinye toujou oubyen si li pral nna yon lòt zòn oubyen menm si li pa ta rete nan zòn mwen an, si l pral nna on lòt zòn kòman y’ap fè pou aspèje emanatè yo pou nou stp?

ITV: Nou gen on pwen sou sa, n’ap rive nan pwen an. Kilès moun ankò ki ap pale?

HM30: Non pa m se HM30, blòk 3, avantaj emanatè a bay pandan titan sa yo menm si l pa fè tan m ta vle, men m’ap bay li a 60 pou san, m konn mete l deyò sou galri a lakay la, m pa konn jwenn marengwen, li repouse l se vre, men lè n anndan sa ka rive nou jwenn marengwen yo pa vini jan pou yo ta vini an, men a 65 pou san li bon pandan de jou sa yo.

HM34: Se HM34 blòk 4, sak pase lè ou fèk gen emanatè a,tankou jan HM45 sot ap esplike a se vre, lè ou fèk gen emanatè a,kèlkeswa pozisyon ou mete l, nan espas ke ou ap evolye a,pa egzanp ou pran sal sa,ou mete emanatè lè ou fèk genyen l ou pa bezwen plede manyen l, li jis trete espas la menm konsa m konsidere l kèlkeswa kote ou chita ou p’ap jwenn,kèlkeswa sa ou jwenn pou fè nan espas la,ou p’ap jwenn moustik men alalong paske l’ap diminye nan efikasite l, vin fè ke alalong ou vin mete l nan sitiyasyon pou vin apwoche l plis de oumenm, se ki fè ke lè n’ap pale de transflitrin nan, de dozaj lan tankou jan kamarad la sot di la,madam HM3 te sot pran on egzanp ke m te renmen, se vre, lè ou gen emanatè, kòmsi lè se lakay ou k’ ap resevwa materyèl, k’ap fè stokaj materyèl yo, lè ou epapiye emanatè yo, ou jwenn plis efikasite nan kay la,ou jwenn plis efikasite nna espas ou ap evolye a, e tout moun nna kay la tou,men lè ou pa genyen tout emanatè yo,se de grenn emanatè ou genyen, mpa gen menm efè a.

ITV: M panse te genyen de seri emanatè, gen moun yo te bay de emanatè.

X: Yo te bay de emanatè pa fwaye.

ITV: Gen moun yo te bay ankò?

HM34: Pou lakay mwen se de sèlman yo te bay, m kwè se menm ka pou HM35 tou.

ITV: Men èske de sa yo, yo te trete yo pou nou aprè?

HM34: Yo pa janm trete yo.

HM3: Yo te banm de premye emanatè ki te trete kounya otandefwa pwojè a ap avanse yo te vin rekipere emanatè a, ansuit yo rebannou yo ki retrete, yo te bann de emanatè, yo reprann, y’ale yo fè etid sou li, yo retrete l, yo ban mwen l ankò.

HM34: Sa se nan ka pa ou la, men nan pa m nan yo pa chanje l, m byen di wi, nan ka pa m nan ak an pa HM35 a,yo fin ba nou emanatè a,yo pa janm retounen sou plas ankò.

ITV: Bon, èske gen lòt repons ankò? Ebyen menmsi m’ap avanse ak kesyon yo, si on moun gen on bagay l’ap di, li ka di l nenpòt lè ou vle, Kesyon twa nan ki fason nou mwenm nou itilize emanatè a, kijan nou itilize l li pi efikas? Gen plizyè fason nou ka itiliz el, n’ap di mwen ki fason ki pi bon, sa ou konstate, ki fason ou itilize l tou ki pa bon? movèz itilizasyon, bòn itilizasyon.

HM35 ; Mwen se HM35 blòk 4, nan koze bòn e movèv itilizasyon emanatè a, se an fonksyon de kote ou mete l la, pa egzanp si se anndan on chanm ou mete de emanatè yo, se nòmal l’ap bay pi bon rezilta ke si se an deyò ke ou ye, pa egzanp bò lakay la genyen l, gen domino mesye yo te konn jwe, si n mete emanatè yo bò domino an,l’ap repouse l men li p’ap menm jan avèk si se anndan kay la ap ye, ke m emanatè a se transflitrin yo mete ladan lè l nan on espas konfine, li evakye plis,men kò m lè se deyò li ye, m pa kwè ap gen menm efikasite a, se sèl sa m ka di kòm diferans ki genyen.

ITV: Sa depan de kote ou mete l.

HM35: Lè ou nan espas konfine, efikasite a plis bon.

ITV: Men èske pafwa tou no konn itlize l on fason nou wè li pa byen mache? Èske nou panse nou konn mal itilize l pafwa, si gen eksperyans ke n te fè di sa, pa genyen, pa gen pwoblèm. Kilè emanatè a nou panse nou byen itilize l? Kijan nou itilize l nou panse ki bon, li pi efikas lè sa?

HM44: Ki fason ki t’ap mye pou itilize emanatè a, se mete l bò kote ou oubyen mete l nan on pwen ki estratejik?

ITV: Sa se kesyon sa ke n poze a men kisa ou fè oumenm?

HM44: Lojik, lè li fèk vini, ou te mèt mete l nenpòt ki kote ou ye, l’ap fonksyone men ofieamzi l’ap desannm kwè ou te meèt mete l bò kote ou, swa l te ka nui ou, m pa konnen, kèlkeswa pwoblèm ki te ka genyen an, èske lè ou mete l pre ou li gen on pwoblèm li ba ou? Paske lè l fenk vini, ou chita la, ou ka mete l distans mete l bò twazyèm chèz la, l’ap fonksyne nòmalman men de, twa, kat, senk jou, ou oblije rapwoche l, ou ka mete l la bò kote m, pou apreprè santi, se kòmsi ke pwodui a trasflitrin ki ladan l,lan li fèb, li vin fèb, li la wi men li fèb.

ITV: Lè li fèb fò ou mete l pi pre ou?

HM44: Lè l fèb, fò m rapwoche l.

ITV: Donk sa se on bon pratik itilizasyon, si l fèb tou ou mete l lwen se on movèz pratik itlizasyon.

HM1: Bon, m panse tou, si ou ap itilize emanatè a, li gen on pwodui ladan, l’ap degaje on odè, m panse ke ekspoze li ‘à l’air libre ‘li ka jwe sou dire pwodui a ta ka fè, plis ou ekspoze li ‘à l’air libre ‘m panse ke si li nan on espas èmetikman fème li ka ba ou plis tan m panse si ou pran malen plezi, se a leksteryè ou ekspoze li, m panse ke l’ap mwen efikas, on lòt lè ou ka vle itilize l, nan tan ke li ta dwe ba ou la, ou ka mete l a lenteryè dire ki te prevwa pou l te ba ou a, li pa ba ou l.

ITV: Ou panse lè a aji sou li, plis li ekspoze deyò, plis efikasite a ap redui, sa ou di a lojik.

HM1: M panse ke menm lè ou itilize on begond si li a lenteryè si li konfine nan on espas, l’ap pran plis tan, li plis efikas, men si m flite on begond ‘a l’air libre ‘se si ou ap wè nna on tan rekò l’ap evapore, epi se on pwodui k’ap degaje ‘a l’air libre’ l’ap evapore pi byen, ke si li an on espas ki fèmen.

ITV: Mèsi Mako pou komantè a, ki limit nou konstate nan itilizasyon an, eseye di fason nou itilize l.

HM20b: Se HM20b, èske gen on limit sa depan de kote ou rete a, devan kabann ou ap itilize li, èske gen on kote jis pou ta mete li pou pouse marengwen an? M gen enpresyon mwen menm si ou mete l la, mwen menm m te gen de, lè yo t’ap pran konya m rete on sèl,men lè m te gen de a sa depan de sa ou ap fè a, ou rapwoche li bò kote ou,men lè m te gen de a m te wè li gen plis efikas, men kounya vin gen on sèl pou mwen li manke, gen kote ki pa genyen, nou pataje, m vin ret a on sèl. Donk èske gen on kote ou ka fikse emanatè a?

ITV: Jan yo di nou, emanatè a, ou pa bezwen kite l on sèl kote, ou dwe itiliz el on fason kreyativ, o ka deplase avè l ou mete l on kote, HM3 konn sa byen, sa HM3 fè, se pa plake l on kote, ou trankil, ou ka nan douch pa egzanp ou mete l la.

HM20b: Pa egzanp si on sèl ou te genyen oubyen de, sa depan de kote ou rete a.

ITV: Wi, jan ou di si ou gen plizyè li pi bon.

HM20b: Imajine sa depan de kote ou rete a, si se on sèl li ye oubyen de, pa egzanp si nou twa nou chita la epi gen espas ki rete bò isi a la, m gendwa kouche,chita m’ap fè televizyon oubyen on lòt bagay, pa rapò a kote m rete a, marengwen an nòmalman m tande y’ap pase, pa egzanp dèyè m, bò pye ou la, sou mwen m pa plis santi yo, se sak fè nòmalman si ta gen plis bagay, si yo ta ban ou plis, e jan sipèvizè a t’ap di a si fè l pi piti bon m pa konnen.

ITV: Ebyen sa se kesyon nou poze, nou pa gen repons kounya pou nou bay, m kontan nou di sa paske se nou ki itilize l, nou gendwa pou nou poze kesyon sou sa ki enpòtan.

HM1: Lòt bagay ankò, se itilize emanatè a avèk entelijans, pa egzanp nan ka lakay la, gen de chanm ke nou genyen, paske gen de chanm ki sitiye a lès, ki gen fenèt ki gran,ki byen ayere chanm sa ou p’ap janm gen moustik fasil nan kay la paske zòn sa li fè frèt,e gen anpil solèy ki penetre lejou e lanui li fè trè frè, pa gen anpil moustik nan zòn sa yo, men gen de chanm ki sitiye a lwès, se dè chanm ki trè imid, non sèlman yo imid ki fè cho, se nan chanm sa yo marengwen yo gen plis gen tandans rantre e rete plis, si ou gentan wè se la fwaye yo ye, itilize entèlijans mete emanatè a nan zòn sa, ou ap wè ke l’ap repouse yo, marengwen an p’ap rete kote ki fè frèt la, nan chanm kote ki byen ayere yo pa prale ladan, se kote ki cho a yo prale, ou ka mete l nan peryòd li fè cho a,nou mete nan chanm sa, epi leswa lè n nan salon, nou mete l nan salon an,leswa nou mete l nan chanm nou, dè fwa nou mete l an on chanm, nou mete l antre de chanm epi l bay rezilta, itilize entèlijan pou wè kibò pou itilize li, e nan ki moman.

ITV: Klima.

HM1: klima tou jwe on wòl, tanperati, pou ou mete l nan tèl chanm.

ITV: Mèsi HM1 pou kòmantè a. Gen moun ankò ki ap pale? Si pa gen moun ankò ki ap pale, n’ap kontinye avèk kesyon yo, nòmalman nou konnen bagay yo global, nou reponn deja, nou antisipe, n’ap jis retounen, sou sa noun te di deja, bay ide sou sa pou n ta fè, pou n ta amalyore emanatè a? Gen moun ki fè sa deja la. Kòm kesyon an vin poze kounya,eseye reponn.Bay ide pou nou amelyore emanatè a? Piske se nou ki itilize l.

HM20b: Se toujou HM20b ide m genyen, se plis pwodui pou n ta paske toutan emanatè a,jan m ta di ou, si ou gen anpil, l’ap itilize plis, jan ou fonksyone avè li, l’ap vin bese, l’ap redui, si n pa mete kenn pwodui ladan l, m byen kwè nòmalman jan nou t’ap bezwen li efikas la nou p’ap jwenn li, se sa m t’ap di.

ITV: Kisa pou n fè pou nou amelyore emanatè a? Kiyès moun ki genyen repons? Nou gen HM6.

HM6: Bonswa, m se HM6 blòk 1, se menm jan nou te di l talè a, se gwo emanatè a on ti jan twò gwo,li pran twòp espas,pou n ta fè on jan pou n ta minyatirize l, avèk estetisite a tou paske li on tijan manke bèl, si yo te ka fè l on jan pou l pi bagay, pou l pi estetik, ou ka mete l nan salon, pou si ou gen moun ki vin lakay ou.

ITV: Ki lòt moun k’ap pale ankò, kisa pou n fè pou n amelyore emanatè a? Nou envoke kesyon aspè materyèl la, pou l bèl, sipò pwoteksyon an pou l ka, men ou gendwa bay ide tou sou efikasite chimik.

HM45: De nouvo se mwen menm HM45, Sa n kapab fè, gen kesyon ki poze sou dirabilite, m tande anpil kolektè tou, yo remake aprè on dire 6 jou on bagay konsa,men jan l te konn repouse moustik la, li pa repouse, m panse pou yo ta pèmèt pou l vin pi efikas,se ta yon renouvèlman pou yo fè, m ta di a la uitèn, chak 8 jou konsa ebyen, yo remete ankò transflitrin nan.

ITV: Pou yo retrete l?

HM45: M panse on mezi konsa t’ap bon, li t’ap toujou rete efikas.

ITV: On bèl entèvansyon, kesyon an toujou pandant,imajine tout san ou ta dwe fè pou emanatè a kapab pi bon,.

HM35: Se HM35 blòk 4, jan kamarad la sot di sa la,kesyon estetisite a li pou bokou,paske y ka mete l sou fòm pou l ka sispandi, pa egzanp li akwoche nan mi,si gen on mwayen pou n ta eseye palye on seri de pwoblèm ki genyen avèk fòm li genyen aktyèlman, fòm li ye aktyèlman la depandaman de kote ou ap kouche a, ou ka pa ka jwi l plènnman, donk se sou kesynn estetisite a mwen te baze plis, pou yo fè chanjman ladan.

ITV: Kiyès ankò?

HM1: M pa t vle antre nan aspè kòmsi teknik, chimik bagay yo paske se on pwodui li ye,m pa gen okenn ekspètiz ladan, mp’ap al di fò yo ta ogmante pwodui pou l ka pi efikas paske lè ou ogmante pwodui a se si ke li ke gen dè konsekans sou sante, sa vle si ke, le fè ke ou bezwen on rezilta, ou ka gen on rezilta dan limedya ou pa wè marengwen mentnan ka gen dot konsekans sou sante,m panse ke se jis retrete l nan tan ke yo mete a.

ITV: Vwala.

HM1: Renouvle li peryodikman, m vrèman konk kesyon pou yo ta mete plis pwodui san pa ta gen on etid ki montre vwala, men risk.

ITV: Wè.

HM44: Se HM44 de blòk 3, pwodui a, transflitrin nan ki nan emanatè a, li velab pou 90 jou.

ITV: Se sa yo te prevwa.

HM44: Se sa yo te di, men lojik si li valab pou 90 jou, li bann senk jou, ogmantasyon an, jan mesye a di l la,m pa gen pwoblèm non, e ke,ou ta pase nan labratwa pou wè premyèman si gen ogmanstasyon ki fèt, èskem p’ap gen on repèkisyon sou mon nan k’ap itlize l la men di ke mwen kont ogmantasyon an,m pa two al nan san sans sa paske se paske li fèb ki fè li fini avan 90 jou,se 85 jou an mwens wi, li la pou 90 se 5 li fè, lojik la se si on pwoblèm, si se on mank chimik li gen ladan l, po yo al nan laboratwa pou yo gade kisa ki fè li pa fè nonb de jou li ta sipoze fè yo, e si a lye pou yo ogmante l, se senp wi.

ITV: Oke.

HM1: M pa konnen, petèt ke eksperyans ke kamarad la fè a li gendwa diferan nan ka pa m nan, m apèn mande gran sè m HM3, konbyen fwa li te trete, yo te trete l de fwa, lajounen plis fwa m pa le men m konnen sa ke li bay, li di m se de fwa sèlman,e pandan peryòd ke pwojè a nan blòk 1 an,si li trete de fwa e pwojè a la lontan, m wè senk jou a m panse ke li trè pe pa rapò a rezilta ale odela de 5 jou pou zòn 1, m pa konnen si se itilizasyon ki mal fèt de emanatè kit a ka redui nan efikasite l,paske tale a m te di sa, nou konn mete deyò, nou mete l deyò ‘à l’air libre” se si ke pwodui a ka evapore pi rapidman.

ITV: Se on bèl kesyon, èske itilizasyon, èske enpak itilazyon l abizif ke ou fè.

HM1: Se si ke ou fè on movèz itizasyon de li, li ka pa ba ou randman ke ou ta dwe espere a,li mye ke ou itilize li, rezilta ap rete sa l dwe ye a,e talè a m te denonse on bagay ki te enpòtan,se konsènan lòt akonpanyaman anvirònmantal ke ou kapab, zòn kote ou abite a, ki aranjman ke ou fè,ede emanatè a pou l bay plis rezilta lit a dwe bay.

HM35: Se HM35 blòk 4, pou fè swit a sa kamarad la sot di a, reyalite pa l la ak reyalite pa nou an yo diferan petèt menm pou lòt kamarad la paske emanatè pa l la li trete de fwa pa nou an, blòk 4 la, yo annik ba nou li e jiska prezan li pa janm retrete, donk nonb de tan li ba nou sèvis la,l’ap toujou diferan avèk pa kamarad la ki retrete deja se sak diferans lan ki fè,pa nou an se pn sèl fwa, depi lè yo pote l ban nou an tou trete yo pa janm pran li, nou toujou avè l, li pa efikas ditou nan moman sa n’ap pale a.

ITV: M konprann. Men èske ou panse se on pwoblèm regilarite ki fè genyen gen kote yo retrete l, gen kote li pa retrete?

HM45: M raple m,mwen menm toujou m regle afè lojistik gen de blòk nou ale,lè nou ap kite blòk la,avèk emanatè a nou vini yo, nou pran ansyen yo, nou bay nouvo pa egzanp si n’ap on premye pase nan non premye blòk, ebyen nou kite emanatè a,nou gen on dezyèm pase pou nou fè ankò, kounya avèk emanatè nou vin fè dezyèm pase a,lè nou prale n’ap kite l, nou pran premye nna men u na, men m raple m lè nou te su bòk 4,yo genlè te poze on kesyon avèk moun k’ap itilize emanatè yo,li te di ke jiska prezan pa l la ap ba l bon sèvis, se sou nòt sa yo te oblije kite l, poukisa, on ne change pas une equipe qui gagne.

ITV: Si l’ap bay bon rezilta yo pat bezwen chanje l? Se nan blòk 4 la yo te fè konsta sa?

HM45: Wi, gen youn pami moun yo ki te di ke, ebyen pa l la ba l bon rezilta, yo te di pa lapèn pou yo chanje l epi yo te ale avè l, m panse se rezon sa ki fè yo pat gentan chanje yo paske gen blòk sa fèt, blòk 1 yo di sa te fèt, blòk 2 sa te fèt.

ITV: Li retrete?

HM45: Wi, paske jan m te di chak rawonn yo, yo vin ak on lòt emanatè, yo pran ansyen an nan men yo epi yo kite.

ITV: Yo bay lòt yo?

HM45: Wi, m kwè tou li te fèt pou blòk 3.

ITV: Moun yo gendwa pa fè atansyon, yo gendwa chanje l, yo pa fè atansyon si yo chanje l, bon chèf ou p’ap pale?

HM44: Bon, se ankò, HM44 blòk 4, m ta renmen konnen paske lè pwojè a te fèk balanse nan blòk pa m nan antouka, yo te di ke, lè ou mete l an n milye anbyan, yon miliye ki varyab, ki gen solèy, choz sa yo, m sonje ke mesye HM45, lè l t’ap vini, yo te bezwen, m sonje doktè Cyrille, yo te di ke, li te di ke li bezwen yon kote ki imid, m ta renmen konnen èske gen kote, li bay plis daprè sa itilizatè a di gen kote li bay plis efikasite, li pi efikas, m ta renme konnen kijan moun sa itilize l,èske li mete l on kote li gen lè ki pran ladan,li tankou li gen limyè egzanp solèy, ounyen,li mete on kote ki sèk.

ITV: Wè.

HM44: M pa renmen konnen paske mwen m lè m’ap itilize pa m nan, m plis rete nan chanm, dè fwa m konn ap deplase, m fè tout mwayen posib pou l pa mouye paske e materyèl itilize yo sitou choz ki landan l lan, m pa ta renmen dlo tonbe ladan oubyen pou l gen kèlkeswa pwoblèm, se sak fè m itilize l, on kote ki sèk,m pa konn si se akoz de sa ki koz li pa ban m efè ke majorite moun ki itlize l yo, ki di ke li fè on bon titan ap ba oyo bon travay, choz sa yo, paske mwen menm lojikman li banm de jou, sak fè se mwen li bay de jou epi gen moun li bay plis ke mwen, mwen menm li degrade,premye jou li bon, m ka di li san pou san,dezyèm jou, l’ap degrade tou piti ou kòmanse wè yo ap pase, twazyèm jou li rive a 60, katriyèm jou l’ap desann, sekyèm jou li la m’ap gade l l’ap gade l.

ITV: Ki lòt moun ankò ki gen ide pou yo ta amelyore emanatè a an tèm defikasite ou genyen gendwa tou prezantasyon materyèl, jan li dwe ye pou l ta pi bèl, pi konvivyal, m pasne on bagay pou alèz avè l lakay ou, si pa gen moun ki gen kide m pral pase nan dènye kesyon an, dènye kesyon kise ide pou emanatè a ta vin yon pwodui ki vin an tout nan fwaye an ayiti, pou vin on pwodui komèsyal, on pwodui petèt leta ka finanse, m pa konne, pou tout moun ta gen aksè a li menm, ide pou distribisyon emanatè a.

HM45: Se HM45, nan kad fokis goup la nou gen jodya, ansanm kesyon ki poze yo,yo t’ap eseye tou evalye pwodi ki egzsite deja ki kapab repouse moustik, mwen panse ke si emantè a li fè diferans pa rapò avèk sa ki egziste deja yo, ebyen li t’ap byen vin sou si ou teren an, paske nou wè pou sante li preferab, nou wè tou pou aspè finansye a, ekonimik lan li menm tou li preferab m panse ke pa rapò avèk tout pwen sa yo,yon pwodui konsa li t’ap byen vini sou teren, m p’ap di sou mache men sou tèren.

ITV: Trè byen, ki lòt moun ankò ki gen ide pou emanatè a ta vini yon pwodui domestik sou tèren an, demen ou a reponn HM3 paske se pa moman pa ou la,kiyès k’ap reponn?

HM35: Se HM35 blòk 4 bon,pou emanatè a ta yon pwodui komèsyal, fòk moun k’ap itilize l yo non sèlman genyen enstriman pou yo mete transflitrin nan, men tou fò transflitrin nan fòk li disponib li menm tou,paske sa se on eleman enkontounab pou emanatè a fonksyone donk fòk gen disponiblite transflitrin nan ansuit pou n pale de sa,se sa mwen panse.

ITV: Wi se vre, alò ide nou genyen pou nou jere emanatè a tou,pou nou jere l, pou nou antreteni l, bay ide sou sa, ide pou l antreteni, ide pou l distribye tout kote nan peyi a.

HM45: Doktè, pou antretyen an, m te fè remak tou li t’ ap trè bon anvan menm nou bay moun yo emanatè a, pou gen on travay edikatif ki fèt,pouke moun, pou yo kapab konnen kòman pou yo itilize l paske gen anpil moun li te lakay yo,yo pat konn non l, yo pat konn kòman pou te itilize l,yo pa menm m ka di atantif ak efè ke li pote sepandan, si emanatè a vin lakay ou, ou konne men poukisa li vini, ou ap gade pou wè evolisyon l,lè sa ou ap kapab wè rezilta avan travay sa ta fèt,se pou yo ta fè on kanpay de sansibilizasyon ou denfòmasyon pou di tout moun men sa ki emanatè a,men kòman pou nou itilize l,e men kisa nou kapab atann de li kòm rezilta, fòk nou ta gen travay konsa ki ta fèt avan.

ITV: Men, èske pat gen yon moun ki pou te fè travay sa nan blòk yo, yo pat mete on moun?

HM3: Nan blòk pa m an, li te fèt.

ITV: Yo te fè l nan blòk pa ou la?

HM3: Wi.

ITV: Gen blòk pa fè l.

HM3: M pa konnen, m pa ka di sa paske.

HM34: Kisa ki pa fèt la?

ITV: Li di gen moun ki gen emanatè a lakay li ki pa konnen poukisa li la, kisa pou l fè avè l paske pat gen on travay edikatif ki te fèt alabaz, an amon.

HM34: M pa konnen, pou blòk pa m nan li fèt.

ITV: Bon sa ka rive, m wè sa deja nan on kay gen on moun ki pa janm la,epi lè y’ap fè fòmasyon li pat janm la,epi li pa konn sak la, men tout moun ki itilize emenatè a konnen poukisa li la.

HM35: Tankou, na ka pa m nan, a la minit yo ban mwen de emanatè yo mwen enfòme papa m kite la ansanm avè nou la,avèk frèm an ke emanatè a son enstriman ki la ki pou repouse moustik donk a la minit ke ou ap dòmi, depi ou mete tou pre ou men detanzantan kòm l’ap fini, li rale plis vè limenm men m te gentan fè kanpay otou de moun lakay mwen, pou m di yo men poukisa li la, men kijan pou n sèvi avè l,pa kite timoun sèvi avè l,pa kite timoun manyen l, paske se pou repouse moustik li la,donk men kòman pou n sèvi avè l.

ITV: Se sa responsab pwojè a te di ou, epi ou transmèt enfòmasyon an?

HM35: Wi, sipèvizè nou an te fè travay la.

ITV: Se wòl sipèvizè yo, mèsi HM35.

HM35: Wi.

HM1: Nan zòn pa nou an, sè ke chak moun ki vin lakay nou,nou pale se pwojè a, ki se pwòch nou gentan gen demand deja pou emanatè, yo di ou gen on bagay konsa lakay ou epi ou pa janm di sa, sa vle di ke lè ou di moun nan men ki rezilta ou jwenn, men ki byenfè li, li di o,kijan ou ap fè m ka jwenn youn, sa vle di gentan gen on demand pou li,mentnan se ta fè on edikasyon jan ke HM45 di a, e grand echèl,on kanpay de sansibilizasyon, pou se pa sèlman zòn ki touche pa pwojè pilòt la ki okouran de li, sè ke zòn tijo, kanapevè, se pa tout tijo ak kanapevè,ki enfòme de pwojè a, se prezante pwojè a agrand echèl,itilize medya yo,rezo sosyo yo, pou ke tut moun enfòme de on bagay konsa, e gen enfòmasyon osi pou moun konnen gen on pwodui sou mache a ki ap bay rezilta pa rapò a kesyon marengwen an,e se ta kòmanse ap panse a kondisyon a grand echèl, fimansman epi pwodiksyon a grand echèl.

ITV: Trè byen, kiyès k’ap pale ankò?

HM10: kòman kesyon an te di ankò.

ITV: Pou nou ta distribye emanatè a nan tout fwaye, pou tout moun gen aksè a limenm. Bay ide tou pou nou jere, pou antreteni emanatè a.

HM10: Mwen se HM10, on kolektè nan blòk 1, mwen te gen frè m ki te vin lakay la, epi lè m mete emanatè a, li remake l, li di sa sa ye, epi mwen esplike l,men efikasite emanatè, misye te menm mande m pou m prete l li, m di non monchè, m di li pa ka deplase, m esplike l men men kòman li ye, se pa pou mwen li ye, men òganizasyon ki mete l la, nou pa ka deplase, epi li te mande m, li te poze m kesyon sa, li di m konsa men sak fè nou pa elaji pwojè a, nou pa fè dè espòt tankou pou moun te ka achte l, li di ki kote m’ap jwenn achte l,pou n te ka achte l, kisa n te ka fè, m di non se on etid y’ap fè sou, yo poko ka elaji espas la men m konne ke ofieamazi y’ap fin konnen l, gen spòt k’ap bay sou li vrèman,l’ap ka jwenn achte l.bò kote pa ou m panse nou ka fè dè espòt pou tout moun konnen li epi ou t’ap jwenn moun t’ap mande pou pwodui a, paske li trè efikas.

ITV: Bon ide, kiyès moun ankò, gen moun ki pa renmen pale e lè ou vini la, se pou ou pale,kiyès ki ap pale sitou moun kip a pale anpil yo, m ta renmen yo pale, bay ide pou emanatè a distrinye nna tout fwaye, kòman nou ka jere l? HM34 pale anpil men kòm HM34 vle pale n’ap ba l pale,men si gen on lòt moun k’ap pale li mèt pale, allez-y HM34.

HM34: Alò youn nan bagay kòmsi na blòk 4 la nou,ke m te fè mwen menm pèsonèlman, sa vle di,ou konnen akoz de kamera yo te bay pou nou te fè foto a.Mwen te pran de twa fwaye, nou te itilize emanatè a lakay yo, m te kite emanatè a lakay yo pandna lontan, epi apresa m fè foto avè yo nan kay la pou kapab montre efikasite emanatè a,paske sase on kanpay de maketing ke m te fè dirèkteman po emanatè a,alò youn nan bagay ki genyen nan kesyon vilgarite on pwodui se konnen pwodui a dabò,ansuit se efikasite pwodui a, lòt bagay ki genyen sè ke moun k’ap vann pwodui an to fò ke li kapab genyen leplen pouvwa sou pwodui a,pa egzanp nou menm k’ap jwi eksperyans lan de emanatè a talè a jan konfrè a t’ ap di di, nou kapad di kounya pou ekonomik lan, nou fè pati de pwojè a, ekonomikman emanatè a bon pou nou, sa vle ke fò ke moun k’ap fè eksperyans vilgarize l la, fò nou konne ke, jan kamarad yo sot di l talè a, èske en tèm estetik pou n’ap gade kòman n’ap amelyore paske pou mwen menm li on tijan gwosomodo, paske li kapab amelyore paske nou kapab fè li bokou pi bèl, pi atreyan pou n kapab,pou pwodui a kapab pi rantab.

ITV: Trè byen, kiyès ki ap pale ankò? Paske se nan dènye pwen an nou ye,e gen bagay avan nou pat di nou ka di l,san pwoblèm.

HM1: Bon se on kesyon plis ke nan fokis gwoup yo,gen diferan zòn yo gentan enonse deja kesyon efikasite tout moun wè ke pwodui a bon,piske se te on pwojè pilòt,se kan mentman se m ta ka di la majorite de popilasyon an kapab benefisye de on tèl pwojè aske nou wè ke li bay rezilta nan zòn ke n kòmanse a,li endenyab ke nou wè ke li bay rezilta,kilè n’ap lanse a grand echèl.

ITV: Pou nou etann li.

HM10: Pou n tounen avèk on lòt kesyon ankò, efikaskite m wè emanatè a lepòk ke m ap dòmi gen chalè, m bezwen di lè m’ap dòmi gen chalè,kon gen plis marengwen, m remake ke kounya la gen m dòmi pi byen, pa gen marengwen avèk amanatè a.

ITV: Oke, kiyès moun k’ap di on lòt bagay ankò.

HM35: Pou fini avèk kesyon efikasite emanatè a,wi emanatè a bon tout moun dakò alinanimite ke se on pwodui, men anvan pou nou vilgarize l se pou n gentan met tèt nou ansanm tout pwoblèm kòman pou n rezoud renouvèlman transflitrin nan, nan emanatè a,paske sa pou bokou,se vre emanatè a lè ou genyen l li bon, fò n konn nan konbten tan reyèlman l’ap bezwen renouvle l, paske o depa ou di 90 jou, depandaman de blòk yo, li aji plis long pa rapò a lòt,kounya pou nou estatiye l pou konnen nan konten tan pou nou fè renouvèlman transflitrin an emanatè a pou nou rezous pwoblèm sa dabò et ansuit pou n pase a kad distribisyon o nivo nasyonal o nivo global.

ITV: Mèsi se te on bèl kesyon, èske tanperati oubyen klima gen on rapò sou efikasite emanatè a paske gen kote li mache pi byen ke lòt kote.

HM1: Mwen menm m te toujou trè pwòch pwojè a, menm lè m ke m’al travay m swiv de prè, m te enplike a on nivo,m te vle jis konprann, youn nan bagay mwen wè, yo etidye kesyon imidite pou pwojè a, sa vle di, se si ke si yo fè etid sou imidite, se si ke se pa pou ryen yo fè etid la, gen on rezon pou sa, se si ke li gen pou wè bokou a kesyon sa.

HM1: Menm nan zòn detid kifèt Debisi, si n’ap fè n rapwochman ant Debisi avèk kanapevè, se si rezilta p’ap janm menm.

ITV: Debisi pi frèt.

HM1: Wi, li pi woe pi gade nan zòn kte m rete a dèyè do m nan gen on sous dlo ki pase dèyè.

ITV: Kote k pi fret li t’ap pi efikas?

HM1: Se si.

ITV: Ebyen mèsi, kiyès k’ap di on lòt bagay ankò?

HM34: An Maketing yo di sa, tout pwodui ke ou ap teste ou fè etid demache pou li,donk m pa konnen lè pa egzanp n’ap pale de vilgarize kèkeswa tip de pwopagand ke n kapab fè pou emanatè a,m panse ke neyanmwen gen travay kominotè pou n ta fè dan le sans ke, gade espas yo, kòman, si m pran egzanp zòn tijo kanapevè,zòn sa yo bokou pli sen ke si ou pran zòn site solèy ki gen plis fatra, on seri de dlo k’ap dòmi,on seri de bagay, kòmsi ke zòn sa yo ta mande plis emanatè tou,donk sa vle di ke, gen on kanpay de sansibilizasyon,pou yo ta fè an tèm de pwoprete jan konfrè a t’ap di an se vrè, menm si ke moun nan ta gen emanatè a, men fò yo ta netwaye espas yo a tou, lè ou pran egzanp « comme il faut » li di ou sigarèt li ba ou filing men tou yo di ou konsa ke sigarèt nui a sante ou,sa vle di menm si n’ap bay emanatè a, nou kapab di emanatè a bon,men l’ap pi efikas toujou si nou netwaye espas kote nou ye a.

ITV: Sa vle di se pou nou ensite moun yo a chanje de konpòteman, ebyen mèsi anpil.

HM13: Se pa entèvansyon n’ap fè nn, m sonje m poze ou on kesyon ou te di te di m ou ap vin sou li m wè ou pa..

ITV: Ki kesyon li te ye ankò?

HM13: M te di ou konsa èske pwojè a ap kontinye nan blòk mwen, nan tout blòk 4 la, si l p’ap kontinye kòman y’ ap fè yo aspèje emanatè yo?

ITV: On bèl kesyon, mwen, wi pwojè a paral kontinye nan lòt blòk, m pa konnen si se nan blòk pa ou la, men y’ap kontinye pwojè a.

HM13: Nou pa gen pwoblèm pou l kontinye nan lòt blòk, men kòman y’ap fè pou yo aspèje emanatè yo?

ITV: M poko ka reponn kesyon sa pou kounya la, m pa gen repons, m panse kòm se yon pwodui eksperimantal puiske nou wè li efikas, efektivman nou wè li pi efikas pase saki sou tèren an, nou pral fè l tounen on pwodui endistriyèl, ki pa’ chè pou moun ka genyen l.

ITV: Mèsi pou tout bèt entèvansyon sa yo.

# FGD Women Blocks 1-4

Date: 20/01/19

ITV: Bonjou tout moun, nou pral kòmanse ak fòkòs gwoup feminen an maten an, m deja esplike nou deja nan sa nou pral fè, m pa bezwen repran l ankò, epi tout moun ap pale bon kreyòl an bon kreyòl, m pa bezwen anyen an fransè, si ap gen bagay fransè, se mwen men ki ap konnen, m’ap tradui yo soti nan kreyòl rive nan fransè, pale bon kreyòl, pa mare, pale byen, bon no pral kòmanse, premye kesyon an se avan pwojè a te rive nan blòk nou an, nou gen blòk 1 blòk 2 la, kisa nou te konn fè pou pwoteje nou kont moustik? Bay non nou.

HM31: Se HM31. Nou te konn itilize plagatòks avèk begond pou marengwen.

ITV: Dakò.

HM15: Mwen se HM15, avan ke pwojè an pot ko antre nan zòn lakay mwen, m te plis itilize moustikè.

HM48: Avan pwojè a te rive nan zòn nou, lakay nou ki se debisi, mwen te konn itilize blakatòks anpil, m te konn itilize moustikè, m te konn itilize begond tou.

ITV: Èske pwodui sa yo te gen on enpak sou sante nou? Kisa yo te konn fè nou pwodui sa yo? Èske nou santi nou byen lè n itilize yo? Èske yo te ban nou pwoblèm sante? Si on moun ap reponn, reponn, epi bay non ou avan ou reponn.

HM31: Lè ou itilize pakatòks la, li pa fin twò bon pou sante, begond lan tou, m son moun ki asmatik, bagay sa yo lè ou wè itilize yo ou oblije sot deyò, ou pa ka respire byen.

ITV: Pwoblèm repirasyon, kiyès k’ap reponn ankò?

HM49: Mwen se HM49, avan pwojè a m te konn itilize blakatòks, ‘il est vrai que’ blakatòks lan odè an konn nui mwen, men ‘à vrai dire ‘ m pa konnen si l ni sante moun, m byen kontan ke pwojè sa vin rive, emanatè a pa vrèman nui nou, tout kote nou ye ansanm a emanatè a, nan tout aktivite nou ye nan kay la nou senpleman mete l bò kote nou, epi li repouse moustik yo, m twouve sa son bèl pwojè.

ITV: Kiyès moun ankò k’ap di on bagay avan n ale pi lwen? Ki pwoblèm moustik bay menm? Paske nou gen anpil moustik lakay nou, nou rankontre moustik on pakèt kote, nan chanm, nan twalèt, nan ‘salle de séjour, galri’ki pwoblèm moustik bay?

HM5: Mwen se HM5, moustik aji sou sante ou, li bay maladi, divès maladi.

ITV2: Egzanp ki maladi li bay?

HM5: Egzanp chikoungounya.

ITV2: Ou te gen chikougounya?

HM5: Wi.

ITV: Lè ou te genyen l kisa ou te fè pou sa?

HM5: Kòman?

ITV: Lè ou te genyen l ou te pran medikaman?

HM5: Wi, m te pran medikaman epi m te eseye itilize blakatòks alepòk.

ITV: Se lè ou fini, sa vle di ou antre nan on prevansyon tèsyè.

HM31: Lè marengwen an mòde m, li fè m grate kò m, ou wè?li vin ban m on sòt de gratèl, depi ou wè marengwen mòde m se konsa li fè, depi bèt la mòde m epi m grate li, l fè boul konsa.

HM15: Pou mwen menm marengwen an li pa bon, paske lè li mòde li pa fè m byen, alò li fè boul sou mwen e anplis tou gen on maladi ki te frape alò, lafyèv zika, yo fè m konnen se marengwen lè l mòde ou ki ba ou l, alò mwen menm pwojè sa li menm byen anpil paske a lèd de emanatè an m’ap itilize l sitou lakay mwen, mwen remake pa gen marengwen, sa li vrèman enpòtan.

ITV2: Ou te gen Zika?

HM15: Wi.

ITV: Kisa ou te fè pou ou te swaye zika?

HM15: Alò m t’ale konsilte, yo te banm medikaman apresa mwen vin nòmal.

ITV: Men aprè o te fin gen zika ki konpòtman ou te pran pou te pwoteje ou kont moustik?

HM15: M te toujou ap itilize moustikè.

ITV2: Ou kontinye itilize l?

HM15: Non, depi pwojè an parèt nan zòn nan, mwen pa itilize moustikè.

ITV: Dakò, sa se bon temwayaj n’ap bay la,bon kisa moustik la ye?Kòman nou te ka defini l? kisa li ye tibèt sa? Fò m ta bay on definisyon, m di tibèt bay plis nou menm, èske se on bon bèt li ye? Èske se on bèt, èske nou ka asosye benefis a limenm, èske nou ka tire benefis nan bèt sa?

HM49: Nòmalaman moustik la se on ensèk nuizib li ye.

ITV: Ou ka bay plis detay?

HM49: Lè m di nou nuizib, paske li aji sou sante on pakèt moun nan popilasyon an.

ITV: On ajan vektoryèl.

HM49: Wi,se on ensèk ki nuizib, si tout fwa yo ta rive elimine l,nan mond lan, m p’ap di ayiti sèlman.

HM5: Si moustik se on bèt ki bay maladi donk mwen pa panse li fè pati de animal, m pa konnen ensèk ki bon pou lòm, ki ka pouse nou vanse.

ITV: Nan gwoup maskilen an, gen on moun ki te di konsa, tout sa Bondye kreye bon, nou gendwa pa konnen bon kote moustik la, san ka fè avè l, men li di, Bondye pa kreye l pou anyen, se sa li di nou, sèl limenm ki di sa.

HM5: M pa pataje avi sa.

HM15: Menm jan nou sot di la doktè,e tout sa Dye kreye li bon, donk, moustik la gendwa gen on kote ki bon, nou poko ka dekouvri kote ki bon an men mwen menm m’ap pale de kote ki movè an, alò sitou, dèfwa ou fatige nan on ti moman ou ta pran, kòmsi pou ou ta al fè on ti kouche, kay la tèlman gen moustik, li anpeche ou dòmi,e sitou lè y’ap ounou ounou nan zòrèy ou, ou vin pa ka dòmi alò,mwen menm si pwojè sa, ta elimine moustik lan, li t’ap enpòtan pou nou, anpil moun t’ap satisfè avèk pwojè sa.

ITV: Èske gen moun ankò?

HM48: Mwen ka di moustik se on vektè de maladi li ye,paske li transmèt, li ka ba ou zika,li ka ba ou malarya, li ka ba ou lafyèv deng, m’ap gade m te ale nan on lokalite jeremi,tèlman gen moustik ou pa ka dòmi, o oblije ap flite, flite, flite,m flite nenpòt twa begong pandan ti espas di jou mwen fè a,m pa ka dòmi, boul plen kò m,m ta renmen pwojè arrive lwen konsa tankou kote moun ki pa kapab yo,pou ale jeremi, pou al wè kòman moun yo ap viv, yo pa ka viv vrèman avèk marengwen, moustik sa.

ITV: Gason yo di sa tou, yo swete pwojè a vin on pwojè nasyonal, on pwojè ki aksesib ak tout kouch sosyal. Èske gen moun ankò ki ap di on bagay?

HM31: M apiye dam nan pou ale patou e tou, la nan lilavwa marengwen bay anpil pwoblèm, aktyèlman m gen pitit mwen ki abite nan zòn nan, li menm li gen on bebe 5 mwa ki fè malarya se doktè ki di se marengwen ki fè l sa, li obljie tout jounen, tout nwit, li oblije mete l anba moustikè si tèlman gen marengwen nan zòn nan.

ITV: Lilavwa se zòn laplèn yo?

HM31: Wi.

ITV: Se chan moustik ki la wi.

HM31: bon mwen, m te laplèn yèswa, m pa janm dòmi a marengwen akoz pa gen kouran, m pa ka respire blakatòks la ankò, m pa janm dòmi.

ITV: Avan pwojè a te rive nan zòn nan kisa nou te panse de moustik? Bon nou reponn deja, kisa nou te konn fè pou pwoteje tèt nou, nou di moustikè, begond, etsetera, nou di ankò, moun ki asmatik yo bagay sa yo pa bon pou yo, si n’ap konpare ak pwodui sa yo èske emanatè a gen pwoblèm sou sante nou? Èske li anpeche nou respire? Kisa li fè nou? Èske li afekte sante nou emanatè a?

HM49: Non, emanatè a li pa afekte sante nou,tout sa m swete pou chanjman ki pou fèt nan emanatè a,on sèl ti pwoblèm emanatè an bay, li enpe two gwo,si yo te ka redui li paske lè n’ap deplase ansanm avè li,deplasman li t’ap plis fasilite nou pou n deplase ansanm avèk li.

ITV: Kiyès ankò ki ap pale?

HM22: HM22, m remèsye Bondye dabò, lè pwojè a pot ko vin lakay nou tankou lòt moun yo di yo te konn itilize, mwen menm tou se menm bagay la,m te konn itilize plakatòks epi begond ki pa bon pou sante ou,avèk moustikè m te konn itilize, men pa rapò a pwojè a ki vini an,m ka di, kounya la m ka di ou, mwen menm m te toujou di,mwen menm m pa konnen pou lòt moun lakay mwen non, mwen menm nan chanm m dòmi an m pat ka dòmi san kouvri, men m vin gen on moman, mwen kouche, mwen dòmi san kouvri,m pa jwenn marengwen k’ap nui m, ni deyò tou, m wè, epòk lapli ap tonbe konsa marengwen konn ap ba ou pwoblèm, dè fwa ou ap priye Bondye ki lè pou on van pase pou l ale avèk enpe,m ka di pandan pwojè a la, m pat wè sa ditou men gen on bagay nou konn di ki fè m vin wè, sak fè lè n t’ap poze kesyon m te di toujou di, dènye moman, m bay nèf edmi, kounya m ka di m’ap di m’ap bay dis, daprè sa nou te di m, emanatè a li gen on bagay ladan, on moman li kapab fini, nan moman avan m vin la,m ka di m wè kèk tigrenn k ‘ap retounen sanble ke se sa ki landan emanatè a ki kapab fini, ki fè m wè on grenn, semèn pase a m kouchem m tande youn k’ap soti,m pa konn, m pa konnen kote sa,,ki sa li ye nan gouvènman, pou vwa l’ap vini an,si m pran presyon, m te ka kouri pou li,se on kalòt m pase m pa konn si li pran l, a si li pa pran l kounya m pa janm tande l, dè fwa, kounya la, emanatè a gendwa vin,sa ki ladan gendwa fin fèb, m wè kèk tigrenn k’ap pase,paske daprè sa nu te di, li gen on bagay ladan l, si l fini, m kapab wè tigrenn k’ap parèt, se sak fè kounya mwen di, m ba l dis paskem wè vrèman sa nou di, m pa wè yo, kounya m vin wè yo, m wè sa nou yo, se pouse li pouse yo, se pa tiye li tiye l,men mwen menm si n te ka tiye yo nèt pou m pa tande egzistans yo ditou paske bèt sa li nwizib anpil. L’ap vin fè on kou sou ou,li fè ou konnen l’ap vini,ou konprann lè ou pral fè n kou sou on moun ou ap parèt byen dousman, sa menm li fè ou konnen ke li ap vini, sa vle di.

ITV: Li briyan, se pa on atakan silansye.

HM22: egzakteman, sa vle di yo santi yo genyen bwa dèyè banann yo,m ta bezwen kòmsi pou yo ta elimine l nèt.

ITV: Ebyen bagay yo kòmanse dinamik wi echanj yo, mèsi se on bèl entèvansyon, nou te konn itilize plakatòks, on banm lòt bagay tankou begong, ensektisid, ki limit bagay sa yo? Èske bagay sa yo tout bon vre yo chase moustik? Tankou begond, sèpanten oubyen sa yo rele plakatòks la? Èske yo kouri dèyè moustik vre bagay sa yo? Daprè eksperyans ke nou fè, èske li efikas vre?

HM31: Lè ke ou flite, ou gendwa flite epi ou pa tande yo, swa yo gentan mouri, oswa tou yo gentan ekate yo soti, menm blakatòks la, lè l fini yo tounen pi rèd, depi blakatòks la fini, m pa konn si se on kote yo kache,yo tounen.

ITV: li dire kèk segond.

HM31: Wi.

ITV: Kiyès ankò k’ap pale?

HM49: Nòmalman blakatòks la, ni begond nan, yo pa tiye marengwen an, yo sèlman elwaye l pou on nonb de tan apresa yo retounen. De mèm tou pou emanatè an, mwen mande konpayi an pou yo ajoute plis dòz nan emanate an paske li efikas lè nou fèk genyen l, aprè on nonb de tan efikasite a kòmanse vin diminye.

ITV2: Aprè konbyen tan konsa?

HM49: M remanke li kòmanse diminye aprè 22 a 25 jou dòz la kòmanse diminye.

ITV: Kiyès moun ankò ki gen entèvansyon pou fè paske tout moun lib pou pale, di tout sa yo panse sou emanatè a,paske nou nan kad eksperyans, nou itilize emanatè lakay nou.

HM31: Akyèlman, kounya, emanatè a pa remèt anyen, li pa remèt anyen menm, oblije reziye a marengwen an paske mwen menm m pa ka pran blakatòks la, ni begond nan ankò.

ITV: Efikasite?

HM31: Li fini, efiksaite an fini kounya.

ITV: Ebyen se pa on efikasite absoli, etènèl, nou konnen l’ap fini avèk letan,e pa gen kenn pwodui kounya ki ka fè tout tan sa,menm lè yo di ou de jou se manti.

HM49: Mwen menm sa mwen pwopoze a mwen ke yo ta fè emanatè a tankou blakatòks yo, yo diminye yo fè l.

ITV: Yo minyatirize l.

HM49: Wi, egzaktaman tankou on blakatòks a chak fwa li renouvle, m panse li p’ap la pou le tan e letènite men fò pwodui an ta fini, fò konpayi an panse pou yo te ka ban nou l tankou se blakatòks,obyen yon lòt pwodui.

ITV: On bagay ki plis konvivyal.

HM49: Egzakteman.

ITV: E se menm komantè sa yo gason yo fè tou, youn nan dezavantaj emanatè an, yo di li pa estetik, li pa bèl, li pran twòp espas.

HM5: Se vre blakatòks y opa gen on dire ase long pou pwoteje nou kont moustik sitou leswa,lè blakatòks la fini, moustik tounen anvayi nou ankò, men moustikè a fè cho,se sak fè m wè, youn nan bèl avantaj ke emanatè a te genyen sè ke li pa kòmsi kouvri, byen ke yo di li ankonbran, li pa estetik men kòmsi nan somèy nou, li pa kòmsi fè nou cho byen, kòmsi li pwoteje nou pou on dire ki pi long, ke lòt pwodui ki te gen avan, m twouve sa se on bèl avantaj.

ITV: Bèl kòmantè HM5.

HM15: Emanatè a, li vrèman enpòtan, mwen menm m’ap swiv li lakay lan, m pa remake marengwen non, men mwen gen on kesyon m ta renmen poze ou doktè, èske yon kay ki gen plizyè chanm nou pa ta ka pèmèt gen plizyè emanatè.

ITV: Se on kesyon, se pa sèlman ou menm ki poze l, gen anpil moun ki poze l, yo ta renmen pou yo ta gen plis emanatè nan kay la, bon sa mande yon kou, fò l trete etsetera men mwen panse youn nan finalite pwojè a se ede moun gen pwodui sa lakay yo a yon pri ki aksesib, li gendwa se 5 goud ayisyen epi ou ap jwenn òganizasyon finanse l, peye l pou ou, se sa m panse yo pral fè.

HM15: Paske mwen menm m konn ap swiv sitou nan lakou lakay lan e lè m deyò a m wè m remake marengwen, lè m anndan m pa remake l, m panse si tout fwa ta gen omwen de, lè m’ap fè aktivite deyò m t’ap itilize, m pa konn si ou konprann non, youn deyò a.

ITV: On sèl ou gen lakay ou?

HM15: Wi, on sèl.

ITV: Se pa t de yo te remèt ou?

HM15: On sèl.

ITV: On sèl yo te ba ou.

HM15: Wi.

ITV2: Ou pa konn sèvi avè l deyò?

HM15: M pa konnen non, men on sèl m wè nan chanm nan.

ITV: On sèl ou wè nan kay la, men nòmalman se de.

HM15: M pa itilize l deyò HM20b pat di m ke m ka itilize l deyò.

ITV: M kwè se li ki pran lòt la bay on moun, men se de yo te bay, m sonje sa wi.

HM15: M pa konnen,li te gendwa vle on lòt moun patisipe avèk emanatè a, men mwen menm lè m’ap itilize l lè m anndan m pa remake marengwen, men lè m deyò nan lakou a.

ITV: Ou pa janm mete emanatè a deyò?

HM15: Non,m pat resevwa lòd pou m te mete l deyò.

ITV: E poutan yo te bay lòd pou mete l deyò, kote ou ye, ou ka deplase avè l, donk se enspektè a ki pa t fè mesaj la pase.

HM31: Emanatè a, lè ou chita deyò ou mete li chase marengwen paske nou konn deplase l, kòm pwoblèm ki genyen li twò gwo pou m apiye dam nan, m apiye demwazèl lan paske lè ou chita nan salon an,epi pou ou ap deplase on gwo bagay sa pou met kote l, li pa efikas.

ITV: Wè, pwoblèm konvivyalie e se on pwoblèm…

HM22: M gen on kesyon tou, tankou lakay la m gen de,gen yon pitit mwen ki dòmi nan yon chanm, li gen youn nan chanm li men m pa kwè, m pa konnen si l ka ba l anyen paske se on moun ki dòmi avèk vantilatè, depi l antre se vantilatè li mete pou jiskan li jou si gen kouran, alò m pa konnen si sa ka fè on efè sou emanatè sa,paske gen lòt pitit mwen an ki dòmi nan chanm nan, ki di li konn wè marengwen, m ta renmen konnen lòt pitit mwen an ki toujou itilize vantilatè a, èske li pa fè on efè, èske m ka travèse l nan chanm lòt tidam nan,èske l’ap toujou efikas?

ITV: Wi depi l anndan kay la, l’ap toujou mache.

HM22: Non, se paske kote l ye, m di ou moun nan li sèvi ak vantilatè limenm, se nwit li fè avèk vantilatè pandan ke li nan chanm li an.

ITV: Li pa gen emanatè ladan l?

HM22: Wi,li nan chanm nan.

ITV: Li gen emanatè avèk ventilatè?

HM22: Wi, li pa konn anyen nan afè emanatè a limenm.

ITV: Se HM16?

HM22: Non, youn ki rele Doudou. Se HM16 ki pa gen nan chanm li an, m t’ap travèse l nan chanm pa l lan.

ITV: A wi, li ka fè sa,li pa bezwen gen vantilatè avèk emanatè a an menm tan, li ka prete lòt moun yo li.

HM22: Dakò.

ITV: Èske gen moun ankò ki gen repons oubyen n’ap kontinye paske gen anpil bagay la pou n di, gen anpil moun ki di emanatè a gen odè, èske emanatè a bay odè vre?

HM49: Non, emanatè a pa bay odè.

HM5: Non. M pa wè li bay odè.

HM31: M pa wè odè.

HM22: M pa wè sa ditou ditou.

HM15: M pa remake sa.

HM48: Wi, li gen odè.

ITV: Kòman ou wè odè a?

HM48: Kòmsi li pa tèlman, kòmsi lè li twò prè ou, ou santi li on ti jan fò, men m pa konnen aprè, lè li kòmanse ap fè plis jou, ou wè li ale.

ITV2: Li pa dezagreyab odè sa?

HM48: Li pa trè dezagreyab, mwen menm m se on moun ki alèjik, nenpòt bagay fè m ap etènye, etènye sa rete.

ITV2: Èske emanatè a fè ou sa?

HM48: Bon, nenpòt bagay fè m sa, m pran on pafen m respire konsa etènye.

ITV2: Donk emanatè a konn fè ou sa tou? Li konn fè ou etènye?

HM48: Wi.

ITV: Dakò, se ka se pa emanatè a, paske daprè temwayaj mwen jwenn yo di, gen Mako pa egzanp, nan ‘focus group’ maskilen an li di emanatè a pa fè l anyen, li pa deklanche.

ITV2: Kòm ou di tout bagay fè ou sa, kounya ou pa konn egzakteman kisa ki koz.

HM49: Sou, banm gade 6 on sèl moun ki di deranje li, sa vle di gen on lòr pwoblèm, se pa emanatè an.

ITV: E daprè rankont mwen te genyen yè sou sa avèk moun yo, emanatè a pa bay odè se sant sak kat fil la,twal dejit la se li menm, ou konn pran sant twal sa?

HM48: Wi.

ITV: Transflitrin nan pa gen odè, se on bagay ki inodò, mwen menm tou m te panse li te bay odè,se lè m aprann, yo di li pa gen odè, se sant sak la ou pran, ou konnen gen moun se sa yo pè ki fè yo kwè bagay la bay odè,se on odè fiktiv, se pa on odè reyèl,se on odè imajinè, men se pa egziste.

HMX: Petèt m fè reyasyon alèjik ak sak la.

ITV: Wè, alò, ki avantaj emanatè a bay? N’ap bay avantaj yo, ansuit n’ap bay enkonvenyan yo. Ki pwoblèm emanatè a bay? Men dabò n’ap pale de avantaj avèk.

HM49: Nou pale de dezavantaj emanatè a deja, nou swete ke pou òganizasyon an, redui nan gwosè an,epi ansuit l’ap fasilite tach nou lè deplasman yo.

ITV: Oke la ou bay on dezavantaj paske li twò gwo, e pou avantaj kisa ou t’ap di?

HM49: Avantaj, li repouse marengwen yo,e ke li pwoteje nou kont viris ensèk yo ka ban nou.

ITV: Kont viris moustik ematofaj, paske se bwè san, se sa pou yo fè, jan eyedès eyejipti, jan albipiktis,se yo menm ki bay aboviroz yo, maladi, chikoungunya, zika, deng, menm lè isi yo poko jwenn ka,men yo jwenn ka ki sispè,ka avere, ka sispè chikougounya e zika,pou avantaj, kiyès moun ankò k’ap pale de avantaj emanatè a?

HM5: Avantaj emanatè a,’du fait que’ li repouse moustik, m te toujou di li pèmèt nou dòmi byen, li anpeche n malad,avèk piki moustik yo, men dezavantaj emanatè an,pandan echanj sa jodya, m te ka te konn di li wi, m toujou ap Mayiki retire bagay ou a la,paske dè fwa li nwi m li ankonbre m,men m panse ke menm jan a Metayer si nou te ka redui ladan m p’ap di menm jan avèk dimansyon blakatòks non,men on ti jan pi piti, m panse l t’ap trè enteresan.

ITV: Bèl kòmantè, kiyès moun ankò k’ap pale de avantaj avèk enkonvenyan? Avantaj ak dezavantaj emanatè a bay?

HM31: Emanatè a bay anpil avantaj paske ou konn kouvri pou marengwen, malgre ou limen blakatòks,men kounya lè ou gen emanatè a bò kote ou ou pa bezwen kouvri, li bay avantaj men dezavnataj li genyen se gwo li twò gwo a.

ITV: Wè, kiyès moun ankò ki ap pale?

HM15: Avantaj li bay nou dòmi alèz depi emanatè a anndan kay la,dezavantaj ke li bay, se lè li kòmanse fini nou retwouve marengwen yo, m wè sa, li pa nòmal.

ITV: Se enpak tan sou efikasite a.

HM15: Wi.

ITV: Ki lòt moun ankò k’ap pale?

HM48: Avantaj emanatè a, li ekonomik paske nou te konn achte on pil blakatòks, nou te konn ap achte begond, kounya nou pa bezwen tout bagay sa yo, li repouse moustik, epi nou dòmi byen, dezavantaj li men m te toujou di l depi avan li okipe twòp espas, si yo te ka fè on fason, li te ka plake nan on mi, paske gen kote ki gen timoun, estrikti jan li fèt la, li kapan blese on timoun,ou konprann? Epi m pa konnen avèk pwodi yo trete lan, pou timoun pa manyen l, yo toujou di pran prekosyon pou timoun pa manyen l.M ta renmen pou li ka izole on kote konsa, pou okipe mwens espas.

HM22: Emanatè a bay anpil avantaj, avantaj li bay, marengwen ap mòde ou, lè ou touye on marengwen vale san, ou kouche la ou pa konn ki valè ki souse ou lanwit la tout malere, malerèz nna peyi a prèske anemi, y’ap fin mete ou anemi nèt, youn, lèfini, yo p’ap kite ou dòmi, ou p’ap dòmi y’ap mòde ou. Sa vle di tout avantaj sa emanatè a bay la, li bay on avantaj enòm, pou afè dezavantaj, m pa di li bay okenn dezavantaj paske tout bagay ki kòmanse li gen on fen,sa vle di kounya li se ka pa sa ki ladan l lan pou lakay mwen se sa ki ladan l lan ki kòmanse fèb, ki fè mwen menm mwen di ou mwen wè gen kèk tigrenn k’ap retounen,sa vle di depi yo rekòmanse avèk li ankò, marengwen yo ap chèche on kote, men m di ou se fini m ta renmen yo fini avèk yo nèt paske marengwen, si l gen on bagay li pote, se Bondye ki kreye l m pa ta renmen pou m te fini ak lavi l, men marengwen an anmède moun anpil anpil, si yo te ka fini avè l nèt, m gen kat bèt m ta renmen yo elimine nèt.

ITV: Premye a se?

HM22: Marengwen.

ITV: Dezyèm nan?

HM22: Dezyèm nan se rat.

ITV: Twazyèm nan?

HM22: Twazyèm nan sourit.

ITV: Katriyèm nan?

HM22: Katriyèm nan ravèt. Lè m di sa, marengwen mòde m,men m annafè ak rat, m anafè a sourit,sourit maje 700 dola pou mwen, Kòb la te 720 dola, yo manje 700 dola, Bondye 20 dola tonbe yo pa jwenn 20 dola, yo fè m pèdi 700 dla, lè m voyen chanje l, pa gen okenn kote, yo pa gen nimewo pou m ta chanje l. M te achte ti pou l mwen, rat antre nan on bwat li manje l,si m pa pè rat la m tiye l wi, paske li pa wè m, li sou tibèt la,paske se plizyè ki te la, y’ap rele, lè m pran on wòch m mete sou do l m’ap peze l, m pè l, m retire kò m,epi gen on bwat la, m te ka pran l m peze l m tiye l wi, men m tèlman sezi, m egare m pa tiye l, men m pa kite l fè menm twa jou ankò,m mete on bagay pou li, kote li manje l la, la li bagay,li pa la fè on lòt moun sa.

ITV: Èske nou konstate emanatè a li chase ravèt tou oubyen lòt vèmin yo no konnen tankou ravèt, èske nou wè emanatè a li aji sou yo tou wi ou non?

HM22: Emanatè a, m pa ka di li rive la paske lakay mwen ravèt la m prèske pa wè l, paske m gen on poud nou itilize poud sa pa vle sa k rele ravèt la, li pa vle wè l menm, e se pa on bagay m wè fasil.

ITV2: Ou konn non poud sa?

HM22: Ti poud, bon m pa konn okipe gade l non,sachè li ye a, li vèt, li pa vle wè ravèt.

ITV: N’ap kontinye paske lè a ap avanse, fòk nou bay moun yo on poz kanmenm, kisa ki fè nou itilize emanatè a? E kisa ki ta anpeche nou itilize l? N’ap kòmanse ak premye kesyon an.

HM49: Nou itilize l pou repouse marengwen.

HM5: Au départ ‘ m itilize emanatè a paske yo ban mwen l pou m itilize, avèk pwojè TEAZ ka, yo te di ke y’ap chwazi kèk moun etsetera,nan kad on etid, pou yo itilize emanatè a ansuit n’ap di kisa nou wè, nou itilize l, nou wè ke li repouse moustik.

ITV: M sonje an out lè mwen entèvyouve ou, nan kad antretyen endividyèl, ou te enpe pè, ou te di ou pa vle pou li gen enpak sou sante ou, ou pat vle Mayiki mete bò kote ou men èske ou toujou gen aprè sansasyon sa? Ou toujou gen santiman ke ou ka atenn pa emanatè a?

HM5: Non, aprè echanj ke nou te genyen an,ou te mete klè ke, emanatè a li p’ap gen okenn enpak sou sante m donk, m pa gen oken pwoblèm pou m itilize l.

ITV: Mèsi HM5, men ki rezon ki te fè ou te pè?

HM5: Paske yo di « au départ » m pa konnen si se moun nan ki te mal enfòme m, yo te di ke aparèy sa yo gen pwodui pou repouse moustik, pou sesi pou sela,donk m panse ke pwodui ki te ka nan emanatè a, te ka gen enpak sou sante m.

ITV: Wi efektivman, gen on bann pwodui yo itilize ensektisid yo gen enpak sou sante moun, men sa li menm li gen plizyè deseni y’ap itilize l, yo pa wè li gen okenn enpak sou sante moun. M’ap gad foto yo fè, on tibebe atè, epi emanatè sou kote l, k’ap dòmi trankilman san moustikè.

HM5: Paske an jeneral, blakatòks,m pa renmen odè l, m pa ka pran sant donk, m panse emanatè a nan menm fason an, li t’ap aji sou sante m petèt pa ‘à court terme’ men ‘à long terme’ se sam te panse.

ITV: Men ki, èske gen on rezon ki ta fè nou pa itilize l?

HM5: Non, m pa wè, bon li repouse moustik, petèt paske li on tijan pran espas men meseye adapte m avè l.

ITV: Nou pa gen on lòt moun la ki wè ta gen on rezon ki ta anpeche yo itilize emanatè a, pa gen rezon?

HM22: Non.

ITV: N’ap pran poz dejene,epi aprè n’ap kontinye.

[POZ]

ITV: Bon, nou rekòmanse, kijan nou itilize emanatè ki bon? Daprè nou menm nan ki fason nou itilize l nou wè li bon? Nan metòd nou itilize l nou wè sa mache pi bon, ke yon lòt fason.

HM5: Lakay la m itilize emanatè a nan chanm nan,paske chanm na gen plis gen moustik, m pa konn si se paske, li mwens ayere ke lòt espas yo,donk nou itilize emanatè a la sèlman, an jeneral m pa konn vrèman deplase l,men si m bezwen sèvi avèk espas kote li ye an, m ka deplase l, men se plis kote ki mwens ayere yo, m itilize emanatè a.

ITV: Trè byen, sa se on bòn itilizasyon, le fè de emanatè a, itilize kote ki mwens ayere, kote ki gen plis moustik, pa gen lòt moun ankò k’ap temwaye?

HM31: M itilize emanatè a nan chanm epi pafwa lè m chita sou galri a tou m itilize li, lè m chita sou galri a, lè moun yo al ranmase marengwen yo pa jwenn li, lè swa menm deyò a, se li nou itilize pou n ka chita ak marengwen, anpil marengwen lèswa.

ITV: Kijan nou itilize emanatè a ki bon? Lè n itilize l nan tèl kote nou wè li pi bon? Li chase moustik pi byen etsetera donk, bon pratik, move pratik nou fè de emanatè a, bon pratik yo dabò, move pratik ansuit.

HM22: Mwen menm se pa de sa m’ap bagay paske kote m itilize l la se la m toujou kite l, se sou sa ou te di talè a, ou te mande poukisa nou itilize emanatè a? Sè ke nou pat konnen, alò, se sa k fè m’ap remèsye sèvo sa yo k’ap fè rechèch pou yo jwenn bagay ki kapab itil, m’ap priye Bondye pou Bndye kapab toujou ranfòse yo, anrichi konesans sa yo, pou yo kapab lè yo vini avèk on bagay ki kapab itil moun, alò nou pa t konnen si on moun itilize plakatòks menmsi ou pa met sigarèt nan bouch ou, ou fimen, sa vle di ou gendwa fimen si ou mete on plakatòks lakay ou,ou fimen de pòch sigarèt, san ke ou pa menm konnen, sa vle di, m’ap di Bondye pou lm anrichi moun sa yo k’ap fè rechèch, pou l toujou anrichi konesans yo,paske mwen menm m pa konn anyen, sa vle di m’ap priye Bondye, menm si m pa konnen m ka priye, m’ap priye pou moun sa yo k’ap fè rechèch sa yo, pou yo kapab vini ak lòt bagay, pou yo dekouvri lòt bagay toujou, po yo kapab itil mond lan, m pa ka di peyi a, se tout mond lan, m ayisyen, lòt yo se moun yo ye tou. Pou kesyon sa ou poze kounya la, lakay la, m ka di kote m te toujou itilize emanatè a se la li toujou ye, kounya pandan sa la, m pral fè chanjman kò m te di ou talè a, mwen pral fè chanjman pou mete l nan chanm HM16 nan, pou m wè kisa…

ITV: Kisa l’ap bay.

HM22: Paske li di li wè marengwen, li gen plafon an, ti tounen on bagay nan kò marengwen yo,se plwaye on twal, frape l nan yo, depi li wè yo on kote kounya la nou pral esye mete li nan chanm li an pou n wè.

ITV: Dakò,mèsi HM22, kijan nou panse nou mal itilize emanatè a?Ki move itilizasyon nou konn fè ak emanatè a?Ki itilizasyon nou konn fè ki bon? Kisa nou konn fè tou nou wè ki pa mache? Pa gen ekspeyans?

HM15: Non.

ITV: Alò ki limit itilizasyon emanatè a, sa vle di li chase moustik men èske li gen limit li?

HM22: Bon, nou pa t konnen afè limit la non,men mwen menm m vin wè limit lan kounya la, se sa k fè m di ou sa talè a, m te bay nèf edmi men kounya m vin bay dis daprè sa nou te di m, se pouse li pouse marengwen an, sa vle di kounya sa k ladan l lan, se fini li fini kounya ki fè mwen wè kèk tigrenn k’ap retounen, se sa k fè m mande si tout fwa, sa k te ladan l lan, yo ka ranfòse l pou mwen paske m kòmanse wè yo ap retounen.

ITV: Dakò, kiyès moun ankò k’ap pale sou pratik itilizasyon emanatè a? Bon pratik? Move pratik? Ak sou limit emanatè a?

HM5: M sonje emanatè sa te distribye a pati jiyè, out,m ka di debi novanm m te gentan remake aparisyon kèk moustik ki pa t anpil konpare a kounya men deja debi novanm m te kòmanse wè kèk grenn moustik.

ITV: Dakò, kiyès moun ankò k’ap temwaye? Ebyen menm lè on kesyon poze, nou ka reponn aprè paske nou nan on antretyen, on bagay ‘Focus Group ‘.

HM48: Lè emanatè a te apèn vini, li te repouse moustik yo nòmalman men kounya m kòmanse remake moustik ankò men ou konnen li la lontan, m wè moustik ankò, men lè yo trete l,n’ap wè mwens moustik, nou p’ap wè moustik ankò, paske.

ITV: Oke, n’ap bay tout ide nou genyen pou emanatè a ta vin pi bon, pou yo ta amlyore l,fè l vin pi efikas, kiyès moun k’ap bay ide pou emanatè ka vin pi bon.

HM31: Pou emanatè a ta vin pi bon, m wè ke fòk yo ta vin avèk on sòt se esprey, lè yo ban nou li, lè l vin diminye, kounya nou te ka flite li pou l ka remonte, paske aktyèlman letan li vin pa bay anyen la, se on fatra li ye, paske li pa repouse marengwen men si nou gen sprey nou flite li, se sa nèt.

ITV: Dakò. Ki lòt moun ankò k’ap pale? Se byen li di bagay klèman, Kiyès k ‘ap pale?

HM15: Sa ke m’ap di pou emanatè a plis avantaje pou nou menm jan madam nan sot di an, m ta renmen nou itilize yon pwodui a chak fwa emanatè an an bès a lèd de pwodui sa, on begond kèlkonk pou nou kapab flite pou li retounen jan ke li te ye a, paske lè n’ap remake pou kounya li vin an bès, kantite marengwen li te konn repouse a piske li vin an bès, ou ap toujou remake marengwen ap nan kay la, m ta renmen ke nou vini avèk on pwodui ki ka ede emanatè toujou.

ITV: Pou ranfòse, pou l retounen jan li te ye avan.

HM15: Wi.

HM48: Mwen ta renmen ke yo komèsyalize emanatè a, pou moun gen aksè avè l, lè l an bès, paske fò konpayi an fè kòb tou, pou nou al achte pou nou retounen ou konprann? epi pou li dire plis se sa m panse.

ITV: Dakò. kiyès moun ankò ki gen ide pout a amelyore emanatè a?

HM5: M ta renmen ke yo redui nan dimansyon an emanatè a m p’ap di pi estetik non yo gendwa a lèd de sa y’ap mete ladan li gemdwa pa bèl, agreyab, itil, pa egzanp on ventilatè, ou wè li estetik emanatè a gendwa poko ka rive nan dimansyon sa men ke li mwens anbarasan, mwens ankonbran.

ITV: Oke, sa se pou antreteni dispozitif la.

HM15: Èske yo pa ta ka itilize, menm jan m’ap gade vantilatè sa,èske yo pa ta ka fè l yo itilize l menm jan? Yo mete l nan on mi, nou wè, nou remake gen anpil moun k’ap plenyen de longè oubyen lajè espas ke li pran, èske yo pa ta ka itilize l, pou yo plake l nan on mi?

ITV: Se on ide, wi.

HM15: Se on ide m bay menm jan ak ventilatè an nenpòt kote ou ye, ou ap jwenn van, espas ke l pran, li on jan deranjan, si ke ou te vini ak pwosesis sa, m wè ke li t’ap vrèman enpòtan.

ITV: Ebyen n’ap kontinye pale,lè nou di amelyore dispozitif la, sa vle di pou l ta vin pi efikas, pi bon, nou ka rele sa konsa efikasite entrensèk, pou pwodui yo mete ladan l lan ta petèt vin pi bon, pou l ka fè travay la, nou bay ide deja, nou gen ide ankò n’ap bay? Ki ide nou genyen pou yo te distribye emanatè a? Bay ide pou l ta distribye. Ide pou l distribye epi ide pou yo pran swen l? Sa vle di pou to swaye l, antreteni l byen, pou l ka toujou efikas, kiyès moun k’ap bay on ide? Sa mande reflechi.

HM15: M pa sezi kesyon on byen non doktè?

ITV: Bay ide pou emanatè a vin nan tout fwaye.

HM15: Gwosè li ye, m wè majorite moun ki ta dwe itilize l p’ap ka itilize l, paske gen on moun ki ka gen on sèl chanm kay, chanm kay la tèlman ankonbre, espas pou l ta jwenn pou l mete l la, li p’ap jwenn li, m wè si yo ta fè l pi ba, mwen panse ke ka gen plizyè moun ki kapab itilize emanatè a, pou mwen se sa ke mwen t’ap di.

ITV: Bèl kòmantè, mèsi, se on kòmantè, ki ka figire nan bagay la, ki ide nou ka genyen pou jere emanatè a? Pou yo pran swen l? Èske ou gen ide?

HM22: Aspèje li, sa pa sa?

ITV: Wi.

HM22: Pou yo ta aspèje li, pou l kapab toujou efikas jan li te konn ye,men pou kounya, kòm m te di tout bagay ki gen on kòmansman li gen on fen,kounya genyen pwodui ke yo mete ladan l lan ki pou toujou la, m pa konnen kisa yo te fè ladan l, kounya pou l toujou ka fè travay li konn ap fè a.

ITV: Wi, se on bon kòmantè ou fè.Èske gen bagay ankò nou t’ap di avan m pase nan dènye kesyon an? Kisa n’ap di ankò sou emanatè a? Sou eksperyans nou fè ak emanatè a? Nou menm pèsonèlman? Lakay nou? Avan m pase nan dènye kesyon an.

HM15: Emanatè an pou mwen li vrèman enpòtan, e si nan zòn kote m ye a, si ta gen plis, si tout moun nan zòn nan ta itilize emanatè a li t’ap vrèman enpòtan paske lakay mwen gendwa pa gen marengwen men gen on lòt moun nan zòn nan, ki ap rele pou marengwen, si antèt pwogram sa, ta ka fè tout moun nan zòn nan itilize emanatè sa m panse li t’ap vrèman efikas pou mwen, paske mwen menm m pa gen marengwen, piske li kòmanse diminye, n’ap toujou remake kèk ti grenn marengwen men alò tout fwa rezistans lan toujou la, m p’ap jwenn marengwenm m panse si nou ta fè nou kado plis emanatè, chanm, galri, deyò, m panse li t’ap vrèman enpòtan.

ITV: Oke, avan m ale pi lwen, èske gen on moun k’ap di on bagay? M pral poze on kesyon la ki fasil, men kesyon an, si n t’ap bay emanatè a on nòt sou echèl en rive nan dis, kòman nou t’ap evalye emanatè a? Sa vle di n’ap bay on nòt zewo jiska dis, n’ap ba l on nòt, ba l on nòt lè li te fèk vini an e ba li on nòt kounya. Lè l te fèk vini, lè nou te fèk kòmanse itilize l, nan premye mwa yo, ki nòt nou t’ap ba li?

HM22: M ba l dis pou lè l te vini an, mwen menm m te di ou m te ba li nèf senkant, men kounya la lè m vin wè sa nou di yo se sa,mwen vin wè kounya pandan ke li an bès la, m kòmanse wè marenmwen yo, m wè se li menm kite fè m pat jwenn marengwen yo, sa vle di kounya mwen menm m ba l dis, men pou kounya nan moman sa kounya la, m wè marengwen yo kòmanse tounen m ka di m’ap ba l twa.

ITV: Oke HM22, ou sensè, kiyès moun ankò k’ap pale?

HM5: ’Au début” m te fè, on bèl eksperyans avè l, pat gen moustik ditou, m panse li te merite dis la, jiska prezan, m p’ap desann twò ba nan nòt lan, paske se pa emanatè a, se jan yo fè l la, pwodui an fini donk,m kwè ke li ka toujou rete nan tòp 7 a 10,m pa konnen, a mezi ke,yo renouvle pwodui ki te fini ladan l lan,emanatè a toujou efikas.

ITV: Emanatè a li amelyorab, li ka amelyore,kiyès moun ankò k’ap pale? Nòt nou te bat emanatè avan? E ki nòt nou ba li kounya?

HM48: Avan m te toujou si, akoz de twòp espas emanatè a okipe mwen te ba l 8, jiska prezan m ba l 8 paske m konnen se trete pou yo trete l, depi yo trete l, emanatè a ap vin emanatè toujou, l’ap toujou repouse marengwen.

HM15: Èske nou gen repran emanatè an pou nou al retretre l?

ITV: Wi, yo te fè sa deja,on lè yo te bay de apresa yo te bay de ankò, men se menm emanatè a yo pran nan men ou, yo retrete l,pou yo wè, m pa konnen èske yo pral ban ou, m panse lòt faz ki pral rive se pwodui a ki ap disponib pou nou gen aksè a limenm?

HM22: Mwen menm lè m bay dis la, kounya m bay twa, m vle pou nou eseye konprann, se pa emanatè a ki pwoblèm nan, emanatè a fò yo pote manje ba li ou gendwa gen on bèt la ou gade l, se manje ou ba li ki bèt la, men si ou mare l on kote, li pa jwenn manje ou ap gade zo ak po k’ap rete gade ou, mwen menm emanatè a mwen bay twa kounya se pa emanatè a m bay twa non, pwodui a fini ladan l pou demontre pwodui a fini ladan kounya, sa vle di depi li rekòmanse ankò, l’ap toujou pran nòt li te genyen nan, ou konprann men pou kounya la, m di ou m kòmanse wè marengwen yo ap vin ;poze m kesyon, sa vle di kounya la, mwen di mwen ba l twa men se pwodui a k’ap vini pou l vin fè valè l ankò, se pa emanatè a ki pwoblèm nan, se pwodui a.

ITV: Mèsi HM22 pou bèl kòmantè sa, pa gen moun k’ap di anyen ankò, paske m wè tout moun pale, ebyen m pa wè anyen ankò pou mwen di, nou reponn a tout kesyon ki genyen, mèsi pou atansyon nou te ban mwen.

HM31: M ta renmen ke menm lè yo ta bay pwodui pou emanatè a premyèman,aprè pito yo te mete l sou mache moun ta achte l, se pat outan y’ap jwenn pou yo pote l ban nou, men a on pri nou te ka achte l tou, pou nou flite li nan plas plakatòks begond bagay sa yo, l’ap pi fasil pou nou si n di se bay y’ap ban nou toutan, nou gendwa pa jwenn li, gen on lè nou ka achte l a on ti mwayen ke nou ka.

ITV: Sa se on bèl kòmantè wi, kiyès moun ankò k’ap di on bagay?

HM22: Sa m rete pou m di kounya se mèsi, a moun ki te vin ak inisyativ la, moun ki fè rechèck ki te dekouvri bagay yo, avèk tout moun ki te mete touch yo, kèlkeswa moun nan doktè Chikoye, oumenm kòm doktè, lòt moun ki te konn vini lakay la, ki poze kesyon, tout moun ki te patisipe yo, m di Bondye, m di mèsi e m’ap priye Bondye tou, pou Bondye konsève lavi nou, mache avè nou, kouvri nou avè pisans li pou Bondye lonje lavi nou.

ITV: Mèsi, pa gen moun k’ap di on bagay? Paske on moun ka toujou pale wi.

HM15: Mwen remèsye nou anpil pou inisyativ sa, sèl sa m’ap mande nou m ta renmen gen plis emanatè nan zòn nan, fè m kado plis pou lòt moun mwen yo, vwazen yo, paske m pa ka ap benefisye on bagay poun pwochen bò kote m nan pa benefisye l.

ITV: Bèl koze. Pa gen moun k’ap di anyen ankò?

HM22: M apiye sè HM15, lè li di li pa gen marengwen lakay li a, sa vle di tout marengwen yo repouse yo, sa k te genyen lakay yo deja y’ap ranfòse moun sa la,sa vle di lè l mande pou tout lòt mount e ka genyen sa se on moun k’ap pataje, m dakò ak sa, lè l gen nan men l se on moun ki renmen pataje avèk lòt moun, kòm m te di sa deja m te di nou gonayiv yo di, gen kote ki rele laplèn, kote sa marengwen ap manje moun gwo midi, sa vle di kote sa yo, si yo te kapab antre kote sa yo,yo ta ede moun yo, daprè mwen menm, moun nan zòn sa yo, podyab. Anfen, mwen menm m te ale nan on lanmò nan zòn sa yo, on sèl nwit m te pase la, m pa kwè m t’ap ka fè on jounen ankò, moun yo gen pwoblèm, nou menm nou gen ti pwblèm tou wi, bò isi a nou wè sa moustik ap fè nou, men mloun a yo se nan chan marenwgen wi, marengwen plis ke moun ki an zòn an wi, se kanaval wi, sa vle di, nou remèsye moun yo ki vini avèk pwogram sa.

ITV: Mèsi.

# FGD Men Blocks 5-6

Date: 30/05/19

ITV: Nou pral kòmanse, jodya se 30 me 2019, nou pral òganize on ‘focus group masculin’, n ap fè on gwoup ‘photo voice’ avèk gason tou jodya, demen n’ap fè on gwoup diskisyon avèk fanm yo, demen gason pa ladan l, nou pral poze premye kesyon an la,ki konesans nou te gen sou moustik avan pwojè a te rive nan katye nou? Kisa nou te panse de moustik?

HM37: Sa mwen te panse de moustik,lè pwojè a pot ko rive,moustik te konn ban ou anpil pwoblèm, pafwa yo fè boul leve sou mwen, lè pwojè a vin rive kounya, bagay moustik la, m pa wè l, sitou pou lè m’ap dòmi, nan chanm nan m pa wè moustik men, emanantè a, li pa sèvi nan tout kay la, nan chanm nan sèlman,se sa m panse.

ITV: Dakò. Kisa ou te konn fè pou konbat moustik? Pou anpeche moustik la mòde ou avan pwojè a te rive lakay ou.

HM37: Pafwa, m te konn achte blakatòks, m te konn sèvi a begond tou, m te konn flite l, lè m pral dòmi.

ITV: Kiyès nou ankò k’ap pale paske m ta renmen tou moun pale, paske se on gwoup diskisyon li ye, pale bay non ou.

HM41: Oparavan devan lakay mwen te gen dlo,men lè bagay moustik la, pa swa m te konn boule 4 a 5 plagatòks, paske poutèt dlo a ki dòmi deyò a, li poze, li vin bay moustik avèk marengwen, men pa le moman, emanatè an vin se pandan m te parèt konsa, kounya dòk la ta pral bay on lòt moun, m di l m vle antre nan pwojè a tou, li te tou ban mwen l, men m wè depi lè m vin mete l la, li te vrèman travay byen, sa m te vle si, èske yo pa ka mete plis dòz ladan l ki pou tankou lè mouch, lòt bagay sa yo, m ta panse si n te ka fè l konsa paske gen moun mwen bay eseye l, jiskaprezan yo nan men yo, yo poko ban mwen l, gen moun k’ap mande emanatè a.

ITV: Tèlman yo bezwen l?

HM41: Wi.

ITV: Ebyen, kiyès k’ap pale ankò?

HM43: Mwen menm oparavan kisa m te genyen, moustik yo te bann anpil pwoblèm, ou konnen gen dlo, gen kanal dlo,lè ou lage dlo a, epi li sitire marengwen, lè marengwen yo kale, sitou mwen gen de timoun,e timoun yo bousòl konn leve sou yo, e premye tigason m nan te rive gen malarya, pa rapò de marengwen ki t’ap mòode li, men lè n vin rive,li te lè li te tan, lè n resi vin jwenn avèk emanatè a, aparèy sa ki ede n anpil, sitou ankò se nan chanm mwen m itilize l,paske lè m itilize l nan chanm mwen m mete l bò kabann nan, pa rapò kote tigason m nan dòmi an,li pouse moustik yo, sa vle di misye pa prèske gen boul, pafwa lè l leve le maten nou konn jwenn bousòl sou li,pou kounya la, pa gen jan de bagay sa yo ankò, li diminye moustik yo ou konprann,e m konn tankou vwazinaj mwen yo itilize l, dè fwa lè y’a dòmi sitou dam nan gen tibebe, li di m ke moustik yo diminye,men oparavan mwen te konn itilize vantilatè oubyen lè pa gen kouran se blakatòks m te konn itilize, de menm pou vwazinaj la,men kounya la, lè m prete l li, li pa itilize blakatòks,paske ou konnen plagatòks la li pa bon pou sante tibebe a,m plis itilize l, m gendwa fè n de jou avè l, epi m ba li l fon twa jou, men li di m ke li efikas, lie de l, men sèlman jan kamarad la di an,m ta renmen li pi efikas, si pa egzanp ta gen mouch lè yo vini pou l touye yo.

ITV: Se byen.

HM52: Avan pwojè a te rive nan zòn nan, nou te toujou konn itilize blakatòks, nou gen moustikè tou nan kay la, kont viris sa yo,lè pwojè a vin ateri nan zòn nan,li vrèman bon, nou menm nan zòn nan sa k fè m te ankouraje doktè a tou, gen plis demand paske nou menm,nou itilize l lakay la, nan chanm mwen,nan chanm timoun yo, ou konn se de emanatè li ye, nou itilize li,men sa m remake plis, li pa tiye marengwen yo, mank efikasite ladan l, men li se on bon aparèy paske li repoze marengwen yo vrèman,men li manke efikasite paske li pa tiye marengwen yo,m ta renmen, kòm doktè a te di yo pral retounen yo nan laboratwa pou yo mete plis pwodui ladan yo, pou marengwen depli l tonbe ladan pou l mouri m ta renmen paske se on bèl pwojè li ye, epi gen plis moun lakay la ki mande l tou, nonb lan plizyè moun ki enterese avèk sa, ou ap gade nan priz yo tou, m fè l diferan pozisyon, de menm ke pou konfrè m nan, se konsa sitou nou travay de konsè,se sa li ye angwo.

ITV: Dakò, chak moun la gen emanatè lakay yo? Konbyen emanatè nou genyen?

HM37: M gen on sèl.

Les autres: De.

ITV: se k te ou te ofri youn nan? On sèl yo te ba ou HM37?

HM37: Wi, on sèl.

ITV: Alò, kisa moustik lan ye pou nou menm? Lè yo pale de moustik ki bèt sa? kòman nou konprann bèt sa?

HM52: Kòman m konprann bèt sa, se on bèt ki nuizib,se on bèt ki pote anpil maladi, gen de, maladi ou ap vin dekouvri lakay ou marengwen an bay anpil maladi, ou gendwa pike ou pandan ou ap dòmi konsa, oswa ou gen bebe, li vin pike li sa vle di li vin lage on viris anndan timoun nan nenpòt tip de moun nan, ke marengwen an se on bèt ki nuizib an menm tan se pou yo detui, se pa on bèt nou ka amize avè l, jan ou wè emanatè a, ou wè pwojè a vini n’asepte l, nou renmen li, paske li kouri dèyè moustik la, se pa kouri sèlman pou li kouri dèyè l, fò l tiye l tou,paske moustik la li nuizib, li pa bon pou sante moun ditou, ditou, ditou, mèsi.

ITV: Èske gen youn la ankò ki ap di bagay sou moustik, kisa moustik la ye? Kisa li panse de li? L’ap bay non l apresa pou l pale.

HM37: Pa gen anpil moun m ka di, jan konpatriyòt la di nou, moustik se on bèt, depi li mòde ou kote li mòde ou la leve on gwo boul, e li vini anpil maladi pou nou, m sonje lè m te piti moustik te fè m malad.

ITV2: Ki maladi ou te genyen?

HM37: M te gen anpil boul sou mwen, li grate m se lopital yo te mennen m, paske moustik te konn mòde m anpil.

ITV: Ki laj ou te ka genyen konsa?

HM37: Onz, dizan.

ITV: Kisa ou ap di HM53?

HM41: Mwen sa m te vle di,tout emèjensi m vle di fòk yo banm plis emanatè pou moun ki nan blòk yo, paske pa m nan,depi lè yo te vin pran l, pou al pase nan laboratwa depi menm jou a doktè a ba li lakay la m poko janm eseye l,pou m konn si l ogmante kounya, se moun ki di m li pouse marengwen yo plis ke lè l te la deja, m gen emanatè j3, m gen j 6 la,m vin gen de men jiskaprezan m poko janm sèvi avè l,de pi li sot nan labaratwa.

ITV: Moun ou prete l?

HM41: Wi, m bay moun yo, jodya li dòmi kay moun sa, demen li kay on lòt, m poko fè eksperyans lan, men pito mwen menm m pa genyen l, ke yon moun deyò paske mwen m te kòmanse eseye l deja, se sa k fè m mande pou yo bay plis emanatè pou m te ka bay moun sa yo,pa m nan pou m ka itilize l.

ITV: Oke, men avan ou te konn itilize l?

HM41: Wi m te konn itilize l.

ITV: Apresa, moun prete l, yo pa vle ba ou l.

HM41: Non.

ITV: Bon, gen on moun la ki fèk parèt m ta renmen l pale,kisa ou te konn fè pou pwoteje tèt ou kont moustik avan emanatè a te rive lakay ou?

HM42: Bonjou dabò, avan m pat konn fè anyen pou moustik paske lakay mwen an, menm lè mostik lan te konn pwopaje anndan se vre,men li pa konn gen aksè pou li mòde m, sitou leswa, men lajounen m konn pa gen tan tou,men m te konn achte rakèt pou nou pete yo men avèk..

ITV2: Sa nou fè a rakèt la?

HM42: Nou pete moustik lan.

ITV: Rakèt elektrik?

HM42: Wi. Men la ou konnen toutotan bèt la ap pran nesans,e sa ou ap itilizease achte ou ap achte l, e de jou an jou l’ap epuize, sa vin fè tou, gen delè ou santi ou dekouraje, de acha ou ap fè toutan se sa k fè m pasne si gen moun k’ap panse vrèman, lasyans se sa li fè, se reflechi sou on seri de ka pou pote solisyon a yo, m panse ke, nan lin ke nou pran ak jan de aparèy sa yo ke n pa.., l’ap rann popilasyn an on gwo sèvis, e m panse ke popilasyon an ap rive satisfè avèk chemen sa ke nou pran.

ITV: Dakò, kisa moustik lan ye pou ou? Kòman ou konprann bèt sa? kòman ou defini l?

HM42: Moustik lan, se on bèt nwizib,m konnen ke li pote maladi, malarya, alò gen on maladi m toujou pa renmen repete l wi, filya.

Itv & ITV2: Filaryoz.

HM42: Se menm jan avèk satan,yo di si ou ap lapriyè, ou lapriyè nondye, ou pa bezwen lonmen non satan,paske si m rele Jak, si ou ap pale ou pa janm di Jak, a Jak ap fè travay li, li p’ap janm gade men imedyatman ke ou di Jak, li konnen li rele Jak l’ap gade,se sa k fè m toujou swete on moun lè ou ap lapriyè ou pa bezwen site non satan,rele Bondye sèlman.

ITV: Ou pè site non maladi a paske ou gen enpresyon l’ap vin sou, si ou site non l?

HM42: Wi, m panse li ka nan zòn nan, paske m pa vle wè l vre,se on bèt vrèman ki nuizib,e moustik’ces jours-ci’ m pa ka konprann yo menm non,kounya yo vin pran konprime, lontan ou te konn teke moustik la li tonbe, kounya, yo gen fòs,e yo swiv ou menm jan avèk menm on bèt yo enstri, eksperyans ke m fè avèk yo, si ou ap fè on travay la y deplase, yo deplase an kolòn,ou tounen yo avè o la, sè ke fòk ou ta toujou gen on bagay pou ou ap repouse yo.

ITV: Èske nou ka jwenn benefis nan moustik? èske bagay sa ou ka jwenn on avantaj ladan l moustik? Tout moun ka reponn wi.

HM43: M pa jwen oken avantaj, olye de avantaj se detui l’ap detui moun.

HM52: Pa gen avantaj nan sa, se maladi l’ap vin pwopaje.

HM41: Si l soti nan on dlo ki sal, l’ap mòde ou se maladi li ba ou, l’ap mòde on bebe li ka bal maladi, li gendwa lafyèv malarya, fyèv tifoyid, se maladi li ba ou, se deranje li deranje ou, sa pa ka gen avantaj ladan l, se dezavantaj li ye.

ITV: Tout moun di pa gen avantaj nan moustik se sèlman pwoblèm moustik bay. Ki mezi an jeneral, kisa nou te konn fè pou pwoteje tèt nou kont piki moustik? Pou nou touye yo toua van pwojè a te rive nan katye nou?

HM42: Nou pat konnen vrèman kisa pou n, sè ke aparèy nou te konn itilize a se aparèy elektronik lan sèlman, epi depi m piti tou, m konn tande yo di, si ou sanble dife lafimen an konn evakye yo, men yo konn di ke lafimen an pa bon pou sante, fò ou pa fè li, se pa on bèt m wè ki gen on solizyon, ou ka chita ou reflechi epi ou di o pote on solisyon, men kòman m’ap pran on solizyon pou m evakye yo oubyen pou m tiye yo, sof pla men ou, pafwa lè bèt la nui ou..

HM52: Oparavan, jan ou wè m te di ou pou talè a, m te toujou konn itilize tankou lakay mwen nou itilize moustikè, dè fwa nou bagay, itilize blakatòks, men kanmenm tank n’ap detui moustik, moustik p’ap janm sispann,men moustik ap toujou la, men avèk emanatè a, tank nou jwenn plis efikasite ladan l, m kwè matrengwen yo, sa vle di y’ap kanpe, ni distans yo p’ap ka penetre nan chnam kote ou ye a paske otomatikman li gen plis pwodui, sa vle di depi marengwen an vini otomatikman l’ap tiye li, sa se nòmalman l’ap evakye yo,depi pwodui a, sant lan tou,sa vle di odè a, depi marengwen an pran odè a,pwodui ki nan emanatè a l’ap evakye l, l’ap fè l ale lòtbò li p’ap ka jwenn kote pou li pike ou, pike moun lakay ou. De tout fason, se sa.

ITV: Kilès moun ankò k’ap pale?

HM42: M poko bay sijesyon m sou emanatè a.

ITV: N’ap rive sou kesyon an.

HM41: Tankou lontan, paske mwen m pa moun pòtoprens, se moun andeyò m ye, bò lakay mwen lè marengwen yo, wè yo pren fèy ave, yo pran po pistach, ou wè yo sanble dife yo mete ladan, gen on bagay ke yo mete twalèt bèf tou yo mete, vin fè lafimen, ki pouse marengwen yo, fè mostik yo pa rete men se kòmsi se lave men byen nèt siye l atè, ou fè l aswè a, men demen byen bonè yo tounen pi rèd.

ITV: Men lè ou fè l la, yo ale vre?

HM41: Li evakye yo, men apre yo tounen.

ITV: Yo jis ka pouse l, men yo pa ka tiye.

HM41: Wi, fòk nou jwenn on rezoud nan emanatè a,ki pou rale marengwen an moustik lan pou l tiye l, se tiye pou l tiye l.

ITV: Mèsi anpil pou mezi nou te konn pran pou chase moustik, bagay la poko fini, si gen moun ki vle ajoute.

HM43: Plis pwodui pou yo ajoute sou li.

HM52: Se plis pwodui pou yo ajoute nan emanatè a, e pa sèlman pou l evakye yo, fòk li tiye yo.

ITV: N’ap rive sou emanatè a.

HM43: Banm di on dènye pawòl, m sonje lontan, lè m te pi piti gen n ti aparèy ki te konn genyen, men yo te konn genyen on ti katon yo met ladan, li rale marengwen an, e lè l rale marengwen an, marengwen an vin ladan l, ou jwenn tout marengwen yo sou li. Li deti yo, on sòt de ti aparèy men se nan kouran pou konekte l, yo met on ti bagay ladan l, odè a rale marengwen yo, li detui yo, e yo pa wè bagay sa yo ankò.

ITV: Ou pa jwenn aparèy sa ankò?

HM43: Non, e li te trè efikas.

ITV: Li te bon?

HM43: Ou konprann, paske matant mwen te konn itilize yo, nan chanm li, men lè ou leve ou jwenn pakèt marengwen ki detui, nou pa jwenn ti sòt de aparèy sa yo ankò.

ITV: Oke, nou bay anpil mezi nou te konn pran pou chase moustik, alò ki limit bagay sa yo te genyen? Nou te itilize blakatòks, begond, bay limit yo. Ki efè nou panse yo te genyen sou nou?

HM41: Ou ap pale de blakatòks.

O: Wi, begond tou.

ITV2: Tout sa nou te konn itilize yo.

HM43: Lè ou itilize blakatòks la, lè ou gen timoun, oumenm pèsonèlman ki granmoun lè ou mete l bò kote ou, ou pa ka respire vrèman vre,odè a pa bon pou ou, li nui ou, mwen menm li konn nui m lè m mete l bò kote m tèlman gen marengwen m konn mete l bò kote m, li nui m, m pa bezwen pale pou on tibebe, li fè l plis mal, ou konprann paske li pa bn pou iitlize, se sa k fè nou te oblije achte moustikè pou n mete pou timoun yo pou yo dòmi, atout m konn mete moustikè a, m konn kouvri bebe a, dè fwa lè m leve, m’ap bay bebe a lèt m konn jwenn grenn moustik k’ap pike bebe a wi, ou konprann sa vle di ke pou marengwen yo, avèk aparèy sa ki vini an, jan kamarad mwen yo di a, fòk yo ta mete plis pwodui ladanl, olye pou pouse se detui, pou l ta detui yo.

HM42: M mèt te kouche la, si ou pa banm on moustikè, m p’ap dòmi, m’ap toujou panse gen moustik la, malgre van ap vante, oubyen gen èkondisyone, fò m kouche anba moustikè a pou m dòmi, paske m vin gate, tèlman moustik lan li bay pwoblèm dayè se m alèji avè l.

ITV: Sa li fè oumenm?

HM42: Depi, li poze sou mwen, li poko menm mòde m, li gentan fè boul sou mwen. Sè ke ou konnen bèt la, lè ou wè san ou pa ale avè l, bon gen delè m konn menm enève avèk mèt lanati wi, kòm konesans pa m pa mezire, se lasyans m panse ke n’ap fè, paske m panse kesyon lasyans se le poukwa, le kòman se labib ki le poukwa, m panse n’ap jwenn nna peyi ki avanse yo, se menm syans lan, se menm bagay la, n’ap fè on koudèy nan laboratwa alatranje yo, se menm bagay yo ki la,men yo inove, m pasne ke lasyans lan fò l toujou inove kèlkeswa kote l pase, paske li mezire, li ka elaji kò ltout kote l pase, sitou nan ti peyi sa yo lè ou rankontre lè ou rankontre ka sa yo, kote moustik bèt ki anpeche ou viv, pafwa bèt la konn anpeche ou viv, menm mouch pafwa ou gendwa ap manje on bagay la,ou santi ou ta chita n kote pou manje l, andwa konn pa pwopis pou manje l, paske mouch lan tèlman enève ou, lè ou gade nan pye ou, men mouch, pou moustik lan jwe nan pye ou,l’ap nui ou, epi pou mouch lan menm nan manje a, sa vle di ke vi a pa pwopis menm pou nou menm ayisyen, m pa di, pa pa fè l jeneral, gen ti kote kanmenm sa ka pa repete, men m ka di li prèske jeneral, pafwa nan laboratwa yo, yo konn itilize anpoul ou mete l nan plafon kay la, li kouri dèyè moustik.

HM52: M’ap di on ti bagay ankò, m’ap ajoute, ensèk l tèlman nuizib, m pa ta gen pwoblèm avè l non, moustik la lè l pike m li fè boul, e ankò pandan ou ap dòmi, ou tande ‘wawawa’nan zòrèy ou sa li anpeche ou dòmi nèt, otomatikman depi ou gen on aparèy k’ap repouse, k’ap rale yo, tankou dan le tan, nou te gen ti bagay yo te pote pou nou sot lòt bò, li gen on anpoul ladan e mov ou wè kote l’ap pase a, li rale l, li detui l konplètman, men ou gendwa ap dòmi la,ou tande woooo, gade jan nou rete la pezibleman men ou pa wè on grenn marengwen,men gen on travay ki fèt kanmen, de jou an jou, lasyans ap evolye konsa tou l’ap plase sou plizyè ka, jan ou wè, ou pasne yo ka vin ak emanatè a,se konsa tou, y’ap mete plsi efikasite, pou detui marengwen konplètman, mèsi.

ITV: Oke, kounya nou pral pase nan dezyèm tèm nan, paske nou epize premye a la deja, nou ka toujou retounen si gen on bagay nou vle konplete, pou nou di nou te blite pa gen pwoblèm. Ki avantaj nou jwenn nna emanatè a? Ki avantaj a bay? Kisa li fè pou nou?

HM52: Avantaj nou jwenn nan emanatè a, li repouse marengwen yo konplètman, lè marengwen yo, a chak fwa li vle rapwoche de yo menm, tankou nou gen sou do kay la, m konnen tinyès mwen konn ap etidye n’ap wè sa nan foto a, li mete l kote l on fason pou marengwen yo pa apwoche l menm, pou yo pa pike l, paske li trè alèji avèk marengwen, nou konn nan jaden an to, nou ko nap jwe, mwen ak on patnè, nou renmen jwe ti, on ti pwen, ti kwa, ou konprann sa m di ou lan,otomatikman n’ap jwenn, nou konn mete de emanatè yo youn a goch, youn a dwat, pou repouse marengwen an, paske se avantaj sa li bay. Men de lot kote, jan ou wè m toujou di a, li manke efikasite, sa vle di le fè ke li pa tiye marengwen an.

ITV: Sa se on dezavantaj?

HM52: Dezavantaj wi, li pa tiye marengwen an, paske lefè ke li pa tiye marengwen an, sa vle di li vin on dezavantaj konplètman marengwen an li menm, emanatè a repouse l se vre, omoman ou la, pa bliye, lè ou retire l ou ap desann avè l, oswa ou ta fon ti rete sou kay la ankò, marengwen yo tounen pou lablibèl, depi emanatè limenm, li gen efikasite, gen anpil pwodui ladan li, depi l tiye marengwen an, menm marengwen an p’ap vin mòd eo ankò se on lòt ka tounen.

ITV: Mèsi HM52 kiyès moun ankò k’ap pale sou avantaj emanatè a bay?

HM43: Pou kesyon de avantaj kòmsi emanatè a bay, pran pa egzanp tankou lè m’ap fè pitit mwen etidye,oparavan lè m’ap fè l etidye m konn, li gendwa kòmanse etidye, li di m: Papa moustik ap mòde m. Epi mwen menm,avantaj li vin ban mwen lè m mete l la, li pa di m moustik ap mòde l,la se on avantaj li vin ban mwen,epi si pa egzanp tou, li gendwa gen dòmi,lontan lè l gen dòmi, ti gason m nan, pou l ka dòmi, oswa lè pa gen kouran,o vantilatè m an pann fòk mwen avèk on mayo ou byen on bagay m oblije pran pou m’ap kouri dèyè moustik pou jiskaske li dòmi,si se pa sa li p’ap ka dòmi paske,marengwen tèlman ap nui li nna zòrèy li, li di m papa: Fè van pou mwen. Se van m’ap fè pou li, la gen on kote li vin banm on avantaj, men lè l kouri dèyè moustik la, tankou kamarad la di,apre si ou retire l, ou al mete l on lòt kote, men kounya la moustik ap mòde l, m gendwa tounen la m jwenn li, epi m jwenn moustik sou li,ou konpranns e la li ban m dezavantaj la, tankou kamarad la di fòk li te ka detui moustik lan nèt se on lòt moustik ki pou ta vin mòde l.

ITV: Dakò ou te renmen pou l ta gen on pwopriyete destriktris.

HM43: Ya.

ITV: Kiyès moun ankò k’ap pale sou avantaj emanatè a bay?

HM37: Avantaj ke emanatè a bay,lè pa gen kouran dòmi nan je m,m pran l, m met l bò kote m nan pou m ka dòmi,pafwa tou, m konn ap manje,marengwen tèlman ap anmède m, m mete l bò kote m nan epi m manje alèz,se avantaj sa ke l genyen.

ITV2: Se avantaj sèlman ou jwenn ladan l?

HM37: Wi.

ITV2: Selon ou li efikas?

HM37: Wi, li efikas.

ITV2: Nou pa gen lòt bagay nou t’ap ajoute?

HM41: Se mete plis dòz nan emanatè a, sa sa k plis pwoblèm pou nou, paske kote n ye a, non sèlmamn kote m ye a, mwen anlè,men gen yon fon kanalize ravin ki pase la, la m mèt di, m nna manman penba la paske,tout dlo se la moun nan jete fatra,lè lapli vini, dlo desann,fò emanatè a gen on bon dòz ladan l pou l vrèman efikas, paske la m chaje marengwen,sitou devan lakay la, gen on tou dlo ankò, paske lè moun yo benyen dlo aoblije vin rete devan,vrèman gen moustik, men fòk dòz ki nan emanatè a, fòk li ka tiye marengwen yo, se sa m wè kòm dezavantaj.

ITV: Kiyès moun ankò k’ap pale? Padan n’ap pale a ou ka tou di avantaj ak dezavantaj ou enkonvenyan emanatè a bay?

HM42: M poko jwenn avantaj nna emanatè a, m jwenn on ti amelyorasyon, paske m te pote deja presizyon sitou a oumenm, doktè an,ke m te di yon pwodui, si ou pran l, ou sèvi avè l anndan la ki konsa, ki fèmen, espas la trè fèmen si pousantaj la 70 pou san se nòmal, ou gendwa jwenn rezilta, men si ou pran l ou mete l on kote ki vas, kote ke ou gendwa pa pote atansyon a lafimen dife, epi ou pa pote atansyon tou de moustik, lòt bèt k’ap vole alafwa, swa moustik lan ap fè aksyon pa li, mouch la nap fè aksyon pa li, epi gen lòt tibèt ankò k’ap fè akson pa l, si pwodui an ou mete nan, ou gendwa di se pou moustik ou mete l dirèkteman, men tout sa yo se ensèk k’ap vole yo ye, ou pa janm konnen tou, si li pa fè efè sou lòt bèt sa yo, lè sa kapasite pwodui an gendwa pa fin efikas pou espas ki li okipe a, se sak fè m te di ke si ou pran emanatè sa,ou ale sen domeng avèl, ou mete on pousantaj 70 pousan pwodui ladan li gendwa bay plis efikasite,ke si ou vin ayiti, ou ka mete 80, 90 li pa ba ou efikasite ke ou bezwen an, se sak fè lè n’ap …, toujou gade ka yo, anvironnman nou, nou konnen anviwonnman ayiti a se on anviwonnman ki trè sal, menm lakay ou ou ap pote kèk amenajman,pafwa lari a pa fin bon, pafwa vwazen an pa fin bon epitou espas kote ou gendwa abite a, li pa fin kòdyòm sè ke, pou pwòp se pa sèl la ki pou pwòp, si ou te ka fè bèl bagay sa se bò ravin ou te fè l, ke ou vle l ou non,li patap pwòp konsa, se paske gen on espas,depi lè ou ap rantre a, ou ap jwenn pwoprete,jiskaske ou rive ofon, ou kontinye jwenn pwoprete a epi, anviwonnman ap vin rete pi sen.

ITV: Oke, n’ap poze kesyon an ankò, èske emanatè a anpeche moustik mòde nou? Èske li pwoteje nou kont moustik?

HM42: Wi, li evakye yo, paske avèk eksperyans ke m fè, pandan n chita,yo konn anvayi vrèman, ou ap fè yo kalòt, epi aprè ou mete emanate a, de filanfil ou ap wè yo disparèt, epi lè ou wè on grenn ta gen tandans parèt,li tounen tankou fou, li ta poze sou ou,ou p’ap gen kè pou ou wè si l ta ka mòde ou, se youn nan eksperyans m bezwen fè,èske lè l poze sou mwen an l’ap ka mòde m paske m sispèk se emanantè a ki fè l fou.

ITV: Ki fè l sou?

HM42: Wi, ki fè l sou,men m pa gen kè vrèman pou m kite l on long dat pou m wè si l’ap mòde m vrèman.

ITV: Ou pa vle kite l mòde ou.

HM42: Wi.

HM52: Nòmalman, emanatè a, m fè eksperyans vrèman vre,sitou avèk emanatè a,ke m’ap itilize lakay mwen, menm manman m konn di: « Èske li anpeche marengwen mòde nou? » M di manman m repouse marengwen yo, ou ap wè m mete l otou de X lè l’ap etidye,oswa lè sè m nna ap bay papito leson,m mete yo anndan, li repouse marengwen yo konplètman,men li pa anpeche marengwen yo mòde ou,sa ka rive nòmalman, mwen menm sa ke m toujou di, e m’ap toujou di l ankò, si li te gen plis pwodui ladan li, mwen menm se detui pou l ta detui marengwen an, se pa tounen pou vin pike nou men,se pa menm marengwen an, repouse a se vre men l’ap retounen, li toujou ankò vivan,men lè l detui l la nèt, sa se on lòt kalite marengwen k’ap tounen vin mòde nou, s e pa limenm, men m fè vrèman on bèl eksperyans avè l emanatè a avan mesye a te vin pran li paske li itili timoun, otomatikman y’ap etidye m mete l pou l evakye yo, men pou mwen, li manke efikasite,difè ke li pa detui l.

ITV: Dakò, kiyès moun ankò k’ap pale?

HM43: Tankou jan kamarad la di a, emanatè a lè o mete l si timoun nan ap etidye, tankou pa egzanp madanm mwen ap lave m konn fè esperyans lan m mete l bò kote l la, marengwen pa mòde l, li jis repousel, si m retire l m mete l nan chanm nan pandan tigason m nan anndan l’ap fè l etidye kounya ap vin gen moustik k’ap mòde madanm nan pandan l’ap lave a, m konn ap lave tou, m konn lave rad tigason m nan, m konn pran l m mete l bò kote m nan,epi m’ap lave, pa gen moustik, se avantaj sa ke m jwenn ladan l, men si m retire l m’ap jwenn moustik, jan kamarad la di a fò l ta gen plis pwodui pou detui yo.

ITV: Dakò, kiyès moun ankò k’ap pale?

HM41: Pwoblèm ki nan emanatè a moustik yo pa detui sa fè youn, avantaj mwen jwenn m gen de, m mete youn nan chanm nan, madanm mwen dòmi a tifi an, m mete youn nan sal la, m dòmi a tigason an, oparavan marengwen yo te vrèman anvayi anndan, le moman m vin jwenn emanatè m mete anndan, lè m’ap dòmi m pa tande yo, men se pa sa ki pou di ke yo pa la, paske li pa detui yo, se pouse li pouse yo,ou ka di yo pa anndan pandan ke emanatè a bò kote ou, yo rete on lòt kote, men le moman ou retire emanatè a, si ou mete l nan chanm nan pa egzanp nan sal la vin genye, si ou pran sa nan chanm nan ou mete l nan sal la, chanm nan ap vin genyen,paske se pa detui li detui yo, se pouse li pouse yo.

HM37: Avantaj ke m jwenn nan emanatè a, li repouse marengwen yo pou mwen, tankou m konn ap manje, m konn ap gad televizyon, li repouse marengwen yo, men dezavantaj ke m gen ladan l, li pa tiye marengwen yo menm jan konfrè yo di sa, li pa tiye marengwen yo menm, li repouse yo, li pa touye yo, se sa m ka di.

ITV: Pou pouse yo se trè byen?

HM37: Wi, trè byen, men li pa tiye yo.

ITV: Konbyen tan ou fè ap itilize emanatè a?

HM37: M konn fè tout on jounen.

ITV: Depi kilè ou gen emanatè yo nan men ou?

HM37: M bliye dat la.

ITV: SA ou genyen an touju efikas?

ITV: Emanatè yo toujou mache?

HM52: Emanatè y toujou mache, menm avan yo te vin pran yo, li tèlman mache paske li te itil mwen, kounya sa k fè li pa itil mwen, paske yo vin pran l.

ITV: M konprann.

HM52: Yo vin pran l kounya nou pa gen emanatè lakay nou ankò, imajine ou toutolon yo te kite emanatè yo lakay nou, nou te konn ap itilize l, li te o sèvis de nou, nou te konn itilize l nna kay la, kit n’ap lave, kit moun yo ap fè manje, kit timoun ap pran leson,nou sou do kay la, ou ap wè an plennè m fè foto yo ki pral pale de sa tou, ou ap wè tou,sa vle di se pa on ti travay konsa, lejou oubyen n rete nou fè on refleksyon, nou di bon men kijan nou itilize l, konsa m pral fè foto yo pou yo fè rapwochman ansanm avè li, pou yo wè sa yo sanble,yo idantik, se konsa enspirasyon an te vini, m jis fè yo.

ITV: Li te fè anpil tan lakay nou emanatè a?

Le groupe: Wi, li te fè anpil tan.

HM52: Apeprè de mwa.

ITV: Li te toujou efikas?

HM52: Li te toujou efikas.

ITV: Ki pwoblè m emanatè a te bay lakay nou? Èske li te bay pwoblèm pandan n’ap itilize l lakay nou an?

HM52: M pa kwè nou retwouve pwoblèm avèk emanatè a non,paske menm pou moun pase, yo konnen emanatè a la,sa vle di li pa bay oken pwoblèm, tout ti pwoblèm pou l ta bay, li anpeche marengwen,emanatè a vin la pou on bi byen detèmine, pou anpeche moustik mòde nou, se pou sa li vini, sa vle di, li pa bay oken pwoblèm, daprè mwen menm.

ITV: Mèsi HM52, kiyès moun k’ap pale ankò, emanatè a te bay pwoblèm?

HM43: Pwoblèm m ka di li ban mwen pèsonèlman se lè ou gen timoun, ou gen ti bebe ki gen de mwa,paske kote m depoze la, li ka rive jwenn li, li pran l,l’ap mache avè, pou l jwe, m pat vle l touche li,ni jwe avè l paske pwodui k ladan l lan li gendwa deranje timoun nna. Men lè m vin wè, li toujou ap pran l, l’ap jwe avè l, m oblije mete l on kote pou l pa ka rive, se la li te konn banm pwoblèm nan men apresa m vin chanje li, m mete l on lòt kote, li vin pa banm pwoblèm ankò.

HM42: Depi se on bagay k’ap itil ou, ou konnen itilite l,li pa ka nui ou, li pa ka ba ou pwoblèm paske, ou konnen pou ki bi li la.

ITV: kiyès moun k’ap pale ankò, emanatè a lè l te lakay nou, daprè eksperyan nou te konn bay kèk ti pwoblèm?

HM37: Emanatè a pa banm okenn pwoblèm,men m fè on bagay ke m pat dwe fè,on lè m’al eseye pran sant li,kou m mete nen m, li soti pou l toufe m. M t’al eseye pran sant li pou m wè kisa ki ladan l,men li pa banm oken pwoblèm.

ITV: Nou pale de pwoteksyon, èske pwoteksyon sa gen limit?Èske nou santi, li te repouse moustik nèt? Nou di li pat touye moustik se on dezavantaj men pou pwoteksyon an, èske ou santi lè emanatè a bòte ou, pa gen moustik ki vini, li chase moustik? Èske se on pwoteksyon ki total? Kòman nou te wè sa?

HM41: M pa fin di emanatè a jan l pouse moustik yo sanpousan,men li pouse yo a on senkant pousan,sa k fè m di li pouse l a senkant pousan,paske lè li pouse l anndan se pandan emanatè a bò kote ou, le moman li pa bò kote ou moustik yo pran menm plas yo ankò,si l sanpousan moustik lan t’ap detui, jan konfrè a sot si a moustik sa, on lòt ap vini,li p’ap anndan kay la ankò.

ITV: Pa gen moun k’ap di on lòt bagay avan m pran on lòt kesyon? Poukisa menm nou te dakò itilize emanatè a? Kisa k konn fè nou itilize l, lè l te lakay nou? Kisa ki konn anpeche nou itiilize l? Èske nou konn pa itilize l? Si te gen on rezon ki te fè nou pa itilize emanatè a?

HM41: Rezon ki fè emanatè a fòk ou itilize l, oparavan nou te nna plakatòks, sa se poutèt moustik yo,ou vin jwenn yon lòt aparèy ki vle di ou pa bezwen nan itilize blakatòks chak jou, fòk ou gen di goud, kenz goud pou ou achte blakatòks, emanatè a li ede ou, la ou mèt sanpousan li ede ou, chak jou fòk ou ta jwenn di goud ou 15 goud pou achte on blakatòks, men emanatè a,se on sèvis li bay nan kay la, lè l vini an, li te lè, li te tan.

ITV: Ki lòt moun ki ap di on bagay?

HM52: Doktè a, lè l te vini avèk li, li te prezante l pou nou, li di l’ap pot emanatè a pou l chase marengwen, otomatikman li pote l, nou gentan konn pou kisa li pote l, se pou chase marengwen, men si li pa detui marengwen an konplètman men li chase marengwen an, li evakye marengwen an anndan kay la, nou gen de kwa pou nou itilize li,paske li jwe on gwo wòl anndan kay, paske l toujou repouse marengwen an, jan ou wè mesye a sot di a, nòmalman m estime l a senkant pou san, si l te san pou san li t’ap detui marengwen, jan ou wè m te toujou di a, si l te sanpousan se lòt marengwen ki t’ap vini, se pa menm nan ki t’ap vin mòde ou, se sa m t’ap di mèsi.

HM42: Emanatè a te vin tounen pou tigason m nan tankou se on pòtab, paske li vin konnen itilite l, depi l’ap deplase, ou wè pòtab li nan men l, avan li chita, li depoze l, kote l’ap gen bezwen l lan, aprè on dis, kenz minit, li fon gade epi li mete l nan bò sa, paske li menm depi li konnen kisa li ka fè pou ou, espri ou vin preokipe de li, ou p’ap janm bliye l.

ITV: Dakò, gen on moun k’ap di on bagay toujou?

HM43: M’ap tounen di tankou jan kamarad la di a,se vre depi ke yo ba ou on bagay pou itilize, epi lib n pou ou, si l pat bon pou ou, ou patap itilize l,epi sa n te kapab di ankò, imajine ou,prèske chak jou ou ap itilize blakatòks a di goud e pafwa ou gendwa pa men gen di goud la pou ou achte l, e li la ou itilize li, lie de ou, e se on bagay, m pa wè on jou o te kapab bliye l,pa egzanp lè timoun nan ap etidye ou mete l la, lè madanm nan ap fè manje ou mete l la, lè l’ap lave oubyen timoun yo pral dòmi ou mete l la,m wè li ede nou, m pa ka di a sanpousan, si l ta sanpousan tankou jan kamarad la di a, fò tad etui marengwen, m mete l asenkant pou san.

ITV: Lè emanatè a te vini pa gen anyen kite anpeche ou itilize l?

HM43: Non, pat gen anyen.

HM42: Avantaj emanatè a genyen, sèke si ou itilize blakatòks li fè ou pa ka respire, ou itilize begond se on lòt bagay ankò,men li menm li la, ou pa pran okenn sant, epi avant ou, doktè a te di, li p’ap fè oken enpak sou sante pèsonn moun, sa te preyokipe m anpil, depi se on bagay ki p’ap gaspiye sante, oubyen ki p’ap nui ou, fòk ou bal valè ke li genyen, mwen mande ke, pou nou pote yon lòt efikasite ladan l, fè yon lòt kalkil pou nou konnen se an ayiti nou ye, nou pa miyami, nou pa kanada,pwodui yo, ogmante dòz yo.

ITV2: Pou l ak tiye marengwen yo?

HM42: Jisteman.

ITV: Trè byen, ki lòt avantaj nou jwenn ankò nan emanatè a apa lefèt ke li chase marengwen? Ki lòt benefis ankò nan emanatè a?

HM52: Benefis nou jwenn,menm lè emanatè a pa anpil, nou plizyè nan kay la, nou te konn itilize anpil moustikè nan kay la, nou pa prèske itilize tout moustikè ankò, sof mwen menm, m toujou gen moustikè m, m mete aparèy la anndan kot manman m nan, m gen youn bò kot frè m nan, sa vle din ou fè pèmitasyon dèfwa, dèfwa m konn santi m pa ka dòmi anba moustikè, m met aparèy mwen bò kote m, lirepouse marengwen an konplètman, men gen kèk tigrenn ki konn kache, ozalantou yo, sitou ou konn tande: Wouuuu, li anpeche ou dòmi, m tèlman konn wè avantaj, kòmsi li bay on avantaj, m di ou tinyès mwen konn ap etidye m mete l bò kote l su ban pou l ka wè èske vrèman l’ap repouse marengwen, marengwen yo konn vin mòde l, li wè marengwen yo pa mode l ankò, sa vle di li repouse marengwen yo, konplètman.

ITV: Dakò, ki lòt avantaj ankò nou jwenn? Li repouse moustik yo sa on avantaj. Ki lòt avantaj ankò nou jwenn nan emanatè an, depi lè li te lakay nou?

HM43: Depi lè l te lakay mwen avantaj m vin jwenn ladan l, se paske jan ou wè kanarad la di a, mwen te konn itilize plis moustikè, mwen menm ak madanm mwen, nou te konn itilize moustikè, e tigason m te itilize,e pou kounya la, m pa itilize, sè l tibebe m nan m itiize moustikè,m pa itilize ankò, se on avantaj li vin ye, paske si sa pat sa, m t ap oblije itilize twa moustikè nna on sèl kay la, m vin itilize on sèl moustikè, se vantaj sa ke li ban mwen.

ITV: Tout bagay ap byen pase la, nou pa tèlman di dezavantaj. Èske nou ka di kèk dezavnatj emanatè a bay? Nou di li pa touye moustik se on dezavantaj,men èske egen lòt dezavantaj nou te jwenn nan emnatè a, lè l te lakay nou?

HM37: Dezavantaj emanatè a bay, sa m pat vle di l,lè li bò kote, pafwa, m pa konn si se li, men lè li bò kote m,msanti je m ap fè dlo, men m pa gen prèv ke se li k fè m sa.

ITV2: Èske ou te konsa avan emanatè a vin lakay ou?

HM37: Non.

ITV2: Se pandan li vin lakay ou a ou remake sa?

HM37: Wi, se pandan li vin lakay mwen m remake sa, men sa ka rive tou se pandan emanatè a vini m gen yon maladi tou, men m pa gen on prèv ke se li ki fè m sa,m pa ka di sa, m pa gen prèv menm.Sa se ka maladi pa m.

ITV: Ki enkonvenyan emanatè a bay? Bay lòt enkonvenyan nou jwenn nan emanatè a.

HM42: Enkonvenyan pou nou ta jwenn, se si li t’ap degaje movèz odè, paske kote ou mete l la, se la li rete.Li pa bay move sant, m pa wè l gen dezavantaj ladan l.

ITV: Ebyen n’ap pran on poz la, e pare nou pral pase nan twazyèm kesyon an, twazyèm tèm nan, apre poz dejene a, apre twazyèm tèm nan,nou pral pale de foto yo.

[POZ]

ITV: Bon la, nou nan twazyèm tèm nan, nou pral diskite sou twazyèm tèm nan ki se kòman nou itilize emanatè a? Kijan nou te konn itilize l ki bon? Nou te ka di bon pratik itilizasyon, fason nou wè ki pi efikas.

HM52: Emanatè a nou itilize l plizyè fason, nan plizyè ang tou,sitou jan m te di talè a,lè timoun yo ap etidye sitou mwen menm m kouche anlè a, m konn ap jwe m toujou mete l kote n, lè n’ap lave m mete l kote m nan, nan espas ki byen detèmine, lè n’ap dòmi tou m mete emanatè a nan chanm nan, kò m ou konnen se de li ye,se sak fè m di ou emanatè ali rann nou on gwo sèvis ou konprann? Sa vle di nou itilize yo nan plizyè ang, sa vle di nou ka anlè a n’ap jwe, a on patnè m lè apremidi nou toujou ap jwe sou do kay la, nou toujou mete l bò kote nou, nou mete youn a goch, youn a dwat, nou itilize l, lè ti nyès mwen an ap etidye leson tou, de menm ke tou, lè nou pokjo ap jwe nou ba l tou li fè menm bagay la avè l, sa vle di nou itilize l nan plizyè sans.

ITV: Trè byen, kiyès k’ap pale ankò?

HM42:Emanatè a kote li gen plis efikasite se anndan, lè li deyò li gen mwens efikasite, paske nou pa konn sis e kote li ye a ki two vas,se lè li anndan li fè plis efè.

ITV: E lè li deyò?

HM42: Lè li deyò, li ka repouse si pa egzanp se nou 5 ki Ta chita la,m ta eseye mete l ant nou de ou ka wè dènye moun ki pa fin patisipe nan kapsite ke li genyen an, sant lan oubyen odè a,oubyen li gendwa pa rive so senkyèm moun nan,sa depan de jan nou chita,ou konprann sa vle di, li vin gen plis kapasite lè l on kote ki plis fèmen ke lè l on kote ki vas.

HM43: Tankou jan kòlèg mwen di a nou itilize emanatè nan plizyè ang,pa egzanp tankou lè n’ap dòmi,nou itilize l, nou mete bò tèt kabann nan, tankou le lajounen si se pandan m’ap fè tigsaon m nan etidye, ou gendwa wè se sou galri a m’ap fè l etidye m oblije deplase l,m mete l sou galri a,e le swa ankò m’oblije itilize l nan lòt fason m mete  l bò moustikè tifi m nan, e m gen bò frem nan kote m nan,m ba l dezyèm nna pou l itilize, li konn mete l bò tèt li, le lajunne tankou lè misye ap etidye li mete l bò kote l, lè l’ap fè on bagay,li mete l bò tab la,e si se deyò l’ap itilize l li, tankou deyò oubyen sou do kay la li mete l bò kote l, sa vle di ou itilize l nan plizyè ang.

ITV: Dakò, miyès mou k’ap pale ankò?

HM37: Emanatè a, m itilize l nan plizyè ang tou,pafwa laojunen m konn nan salon an, m mete l nan salon an,lè l nan nwit m konn anndan m’ap dòmi, m mete l anndan, pafwa tou m konn anndan chanm manman m nan m’ap dòmi m mete l anndan chanm manman m nan,m itilize l nan plizyè ang.

ITV: Trè byen e oumenm?

HM41: Emanatè a mwen, m genyen yo, paske nò lakay la toujou gen de ti mesye yo,pou yo ka pran plzei yo, bwè kèk byè,jwe domino oswa jwe kat yo, m konn prete yo li, m mete l kote yo, mawengwen pa nwi yo,lè m te fèk mete la yo di kote m jwenn aparèy sa? m di non se nan on bagay USAID, yo te menm pwopoze si yo menm yo p’ap ka jwenn m te di doktè a sa, sa k fè lè m te fè entèvyou ak ou bò lakay la m te di ou sa, paske yo vrèman bezwen l.

ITV: Oke, kijan nou panse nou te konn mal itilize l?Èske gen temwanyaj sou sa? Fason nou te konn itilize l ki mal daprè nu menm.

HM37: Fason nou itilize l ki mal,m te konn mete l twòp sou mwen,ou konprann, li te on tijan twò pre m, se konsa m te konn mal itilize l, m pat sipoze mete l pre m, m te sipze mete l on ti jan ekate m.

N: Li te ba ou pwoblèm lè ou mete l pre ou la?

HM37: Wi,li fè je m fè dlo, men m pa gen prèv ke se li ki fè m sa.

ITV: Dakò, kijan nou te konn itilize l mal? Kiyès moun k’ap pale l la?

HM52: Emanatè a, franchman m’ap di ou sa, nou pat mal itilize l, m te byen itilize l.

ITV: Dakò. Ki limit emanatè a? Nou te konn itilize l men nou di tout fason nou te konn itilize l men limit nan itilizasyon an.

HM52: Emanatè a gen limit, limit emanatè a paske l pa detui marengwen yo,se limit li, mak efikasite l.

HM42: Emanatè a pat gen limit, ou bò televizyon an ou mete l, ou bò kabann nan ou mete l, ou nan kizin nan ou mete l, ou ap lava deyò ou mete l, ou anba galri ou mete l, ou sou do kay ou mete l, li pa gen limit.

HM52: Oke, m te konfonn kesyon an,si se sa li pa gen limit, paske emanatè a ou ka mete l tout kote, ou ka mete l sou do kay, m mete l anndan, timoun yo ap etidye, li pat gen limit, mèsi.

HM43: Li pat gen limit non, tout kote ou ye ou ka itilize l, m’ap lave la m ka pran l mete bò kote m nan,m gen dwa ap fè manje la, mwen men pèsonèlman m mete l la, pandan m’ap pile epis mwen, fè tout bagay m mete l bò kote m nan,sa vle di li pa gen limit.

HM37: Emanatè a pa gen limit mwenm, mwen itilize l tout kote nan kay la.

ITV: Èske nan fason nou te itilize l la te gen enkonvenyan?

HM43, HM52, HM42: Non.

ITV: Bon, kounya nou pral pase nan on gwo kesyon,cha moun la ap bay ide pou amelyore emanatè a, pou l kapab vin pi bon.

HM52: Nòmalman, emanatè a pou mwen, li manke efikasite, m ta renmen kò m mesye a si byen di, yo pral nan laboratwa avè li, yo pral renouvle li, yo pral fè l vin pi efikas, mwen menm m ta renmen sa, paske non sèlman se pa repouse marengwen an sèlman, detui marengwen an konplètman, si gen on marengwen ki ta vin pike ou, lòt ka vini men se pa li menm, m ta renmen kò m lasyans pa limite, m ta toujou renmen pou detanzantan pou yo ta renouvle l, pou yo ta fè bagay yo vin pi bon toujou, pou l vin gen plis valè.

HM42: Sa m ka pote pou teknisyen yo.

ITV: Ou mèt pale wi.

HM42: Pa gen lasyans san teyori, pa gen lasyans tous an pratik, mwen men m fè pratik la, m panse ke apre teyori a fin travay, pratik la fin travay,ke nou ka pote solisyon nan sa n’ap fè a, e ki kote nou vle rive avè l la definitivman, emanatè a manke kapasite, pwodui yo pa ekivalan a espas pwopis yo, kò m m te toujou di sa, eseye fè yon lòt etid,lè zòn nan si li pwòp ki efikasite emanatè a, si l sa l ki efikasite l epi pa rapò a lòt ensèk yo tou, tankou mouch èske li pa fè efè sou odè emanatè a ap degaje a pa rapò, olye pou emanatè a ta degaje odè pou moustik lan, èske mouch lan pa patisipe ladan l tou ki koz ke li pa rive sou moustik lan dirèktaman.

ITV: Dakò, ki lòt ide ankò nou genyen pou n amelyore emanatè a pou n fè l vin pi bon?

HM43: Pou lòt ide nou genyen tankou jan kòlèg mwen di an,m ta renmen yo mete plis pwodui ladan l pou l pi efikas, tankou se pa kòmsi se pouse pou l ta pouse marengwen yo, se detui pou l ta detui yo nèt, pou l ta gen lòt bagay pou lòt ensèk tankou mouch, depi l ta pase bò kote l pou l ta detui l tou, paske se pa sèl mawengwen an senpleman depi ta gen lòt moustik paske sa yo pote dè maladi tou, imajine ke si ou ap manje e pandan mouxh lan vole nna manje an, se on maladi ke l’ap pote kanmenm, ou konprann sa m vl di ou la la? Sa vle di fò n ta mete plis bagay pou l ka plis efikas, se pa sèl marengwen an senpleman, mèsi.

ITV: Oke, kiyès k’ap pale ankò?

HM41: Pou emanatè a se pa sè l kouri pou l kouri dèyè moustik lan, se pa sèl rete estab sou moustik lan, se pa sèl moustik ki ka ba ou maladi, ou gen dwa chita la, ou ap manje on manje epi mouch poze ladan, ou pa konn kibò mouch lan soti,ou gendwa al manje manje a, poutan lit ou kontamine ou,paske se on bèt li ye tou,e mwen menm sa ke m vle pou emanatè a,emanatè a, pa avan jan n wè l la, li kòmanse ase byen men,san pou san m poko ka jwenn li ladan l,kòm sa mwen vle di ankò, m te vle tankou chak kote emanatè sa yo ye, doktè yo te ka vizite kay moun say o pou yo te ka wè kijan moun nan ap viv,nan ki sitiyasyon paske gen de moun,ou gendwa wè kay la vrèman bèl se vre men,alantou l pa bon,paske lè ou al vizite kay moun sa ki gen emanatè a,pandan ou al vizite a, ou ka jwenn de moun, twa moun ki nan le bezwen,ki te vrèman bezwen l ki pa jwenn li,mwen men mwen di ou pa m nan ka janm ka rete lakay mwen, m oblije kite l nan men vwazinaj, men depi lè l sot nan laboratwa m poko janm pran l pou m esye l,se sak fè m mande èske yo pa ta ka elaji bagay la, epi moun sa yo te ka jwenn emanatè, paske depi lè pa m na sot nan laboratwa m poko janm itilize l.

ITV: Dakò, kounya nou pral antre nan lòt ide,bay ide pou emanatè a ta vin nan tout fwaye, pou l ta nna tout kay moun, èske nu gen ide pou sa?

HM41: Pou emanatè a vin kay tout moun, menm jan m sot di talè a, pa m nan m paka oblije fè twa jou, kat jou lakay mwen, paske yo menm yo nan bezwen, sak fè yo bewzen l, se paske yo jwen yon avantaj ladan l, si yo pat jwenn on avantaj ladan l, yo te gendwa fè m ba yo li on sèl jou swa, epi yo remèt mwen li, yo t’ap di m ke l pa bon, si moun nan oblije kenbe l, li pa janm vle ba ou l, se paske li vrèman efikas pou li.

HM52: Sa m ta renmen ajoute, nòmalman, pou emanatè a, pou l n ta ka fè distribisyon an, pou l te ka plis, pou moun yo wè efikasite emanatè a tout bon vre, fòk dòz la ogmante,otomatikman gen de moun depi li wè emanatè a pa touye marengwen an pou li,li ta twouve emanatè a pa fè travay li, sa l ta dwe fè a egzat jan li ta vle a,otomatikman emanatè a tiye marengwen an pou moun nan li efikas, sa m ta renmen, m ta renmen pou nou fè l pi efikas lè gen de moun ki pral jwenn, mwen menm m te gentan jwenn li konsa nu aksepte e nou wè travay li fè a, e m ta renmen lòt moun ki pral jwenn li an jwenn li avèk efikasite non sèlman l’ap repouse l, pou l detui marengwen an avèk lòt ensèk ki genyen yo.

HM42: Mwen men m li esansyèl ken ou ta distribiye l bay tout moun,se pa on grenn moun nan zòn nan ou de grenn moun ki pou ta genyen l, poukisa, paske gen enkonvenyan, si kote HM52 la li repouse l, l al kay lòt vwazen an ki pa genyen l lan, men si lòt vwazen an genyen l tou l’ap repouse l, lè sa li gendwa pa gen chans pou li rete nan kay menm se raje li pran pou li, kounya oumenm lè sa, si ou al anba raje an li mòde ou se ou menm lè sa k’ap chèche l.

ITV: Bèl ide.

HM42: Men si tout moun genyen l nan zòn nan, m panse l’ap genyen plis efikasite olye se ou jwenn on moun ki genyen l la, epi pi ba ou jwenn on lòt grenn moun, sa ka rive vrèman li pa bay kapasite, li pa vin gen efikasite ke lit a dwe genyen an.

ITV: Oke, kounya, bay ide pou nou ta jere l, pran swen emanatè a,ki ide nou genyen pou byen jere l, pou pran swen l?

HM52: Pou nou byen jere l, menm jan nou byen jere l la, yo te pote l pou nou pou nou jere l paske te gen on sèvis li t’ap fè definitivman, nòmalman nou abouti, yo vin pran l nna men nou, otomatikman nou rejwenn emanate a ankò, n’ap toujou byen jere l paske, se on itilite li te ye nan kay la, li repoze marengwen konplètman men, m ta renmen jan ou wè m toujou di a, m ta renmen pou l gen plis efikasite toujou.

ITV: Oke dakò.

HM42: Tout bagay ki itil ou, ou jere l byen. Ou gen on machin, li ba ou sèvis, ou ba l sèvis tou, si ou pa bal sèvis li p’ap ka itil ou.

ITV: Piske li itil li merite jere.

HM42: Wi, ou gen telefòn u li sot nan pòch ou li sot tonbe sa fè ou mal, paske li ba ou sèvis, tout bagay ki itil ou, ou dwe jere l.

ITV: Oke, kiyès k’ap di on bagay pou nou fini ak pwen sa nèt?

HM43: Jan kòlèg mwen di an, pou byen jere l, si pa egzanp bagay la ap ba ou sèvis tout kote ou pase li itil ou, fò ou byen jere l, e si m kapab pran l male m depoze l la epi m epi m kite l se paske m pa konn enpòtans li, men si m konn enpòtans li, fò m kapab byen jere l.

ITV: Pa gen moun k’ap di anyen ankò? Ebyen nou kanpe la, mèsi pou patisipasyon nou nan fokis gwo tematik sa kounya nou pral pase nan foto yo.

# FGD Women Blocks 5-6

Date: may 2019

ITV: Bon, nou pral kòmanse avèk diskisyon yo, alò premye kesyon m’ap poze, avan pwojè a te rive nan blòk bò lakay nou kisa nou te konn fè pou nou anpeche moustik mòde nou?

HM36: Bon avan pwojè a te nou te konn itilize plagatòks, ki pat yon bon bagay pou mwen menm alò, paske mwen te mal pou m respire,plagatòks la, li te konn banm pwoblèm,men m te oblije itilize li akoz nou konnen gen filaryoz ki egzizte se on maladi moun pran nan moustik ki pa enteresan lè ou kite moustik ap mòde ou, leswa lè ou ap dòmi, jèm filaryoz nan san ou,ki pa yon two bon bagay, men kounya nou twouve anpil amelyorasyon akoz emanatè a n’ap itilize a, pou mwen menm li pa degaje okenn odè, m pa twouve anyen de negatif ladan li, depi ou jis mete l bò kote ou, o pa pran okenn odè, okenn sant, li jis chase moustik pou ou.

ITV: Kiyès moun ankò ki ap pale? Kisa nou te konn fè pou konbat moustik avan emanatè a te vin lakay nou?

HM54: Avan ke yo te ban ou emanatè a,sa n te konn, nou te konn itilize plagatòks epi gen on bagay ki rele serana tou, nou konn itilize pou marengwen.

ITV: On moustikè li ye?

HM54: On moustikè li ye, li gen on pwodui ladan l, depi sou ou marengwen an pa kab poze sou ou, ou konprann se li nou te konn itilize, kounya nou vin gen sa.

ITV: Èske li vann chè serena?

HM54: Se pa mwen ki te achte l. M pa konn konbyen kòb li te vann ou konprann men kounya li vin ban nou sa, nou itilize sa, li pi bon pou nou. Menm lè mwen menm, se pa mwen ki itilize l paske m pa gen tan, men m jwenn bon randman ladan l.

ITV: Kiyès moun k’ap pale ankò m ta renmen konnen, fò tout moun pale.

HM40: Lè mesye dam yo t’ap pran aparèy la m pat la, men doktè a konn toujou vini mwen trè kontan, mwen renmen jan nou apresye moun, jan nou pran ka moun,mwen kontan natre nna pwojè a tou, emanatè a trè bon, te gen on seri de moustik, m wè kòmsi moustik yo te plis, kounya m wè yo mwens, mwen remèsye nou pou sa, nou poko ka di anyen mal, jiskaprezan n’ap swiv m te toujou di nou sa, n’ap swiv kòman sa prale men m pat tèlman wè, jan te gen marengwen m pa wè l konsa ankò.

ITV: Oke, n’ap poze ou kesyon an ankò, kisa ou te konn fè pou chase moustik lakay ou avan pwojè a?

HM40: Pou m chase gen de lè se ak on twal m te konn pouse yo, gen delè m konn achte blakatòks tou, paske ou konnen timoun yo anpil.

HM55: Emanatè a ede nou anpil paske te konn gen anpil moustik, anpil marengwen, depi lè nou met emanatè a anndan l’ap fè bn travay, nou vin pa wè yo ankò, men oparavan nou te konn itilize plakatòks lan kont moustik yo,m remake lè vin gen emanatè a, kantite mouch ki te genyen an tou, vin pa genyen l, pou mwen menm m wè li kwape ni mouch,ni moustik tou.

ITV: Ou ap di on bagay? Tout moun ka pale se nan on gwoup.

HM38: Anvan emanatè pot ko rive lakay mwen, m te konn itilize plakatòks, akoz m gen on timoun m pa vle marengwen pike l pou pa ba li vye maladi, men emanatè a trè bon, m pa retwouve anyen negatif ladan l.

ITV: Kisa nou panse menm de moustik? Bèt sa, kòman nou ka defini bèt sa ki rele moustik la?

HM36: Kòman m te ka defini moustik la? Se ti animal ki piti yo ye, ki pote mikwòb nan san ou lè yo mòde ou, ou pa konprann li kreye pa dlo sal, sa vle on bagay ki kreye pa bagay ki sal li pa ka pote bon bagay pou ou,li ka ba ou tout sòt de maladi nan san ou, se antiseptik ke yo ye,li lage tout vye tenten nan san ou,tout vye maladi nan san ou, se pa on bagay ki enteresan ditou,e sa ke m te vle di ankò, doktè Chicoye te ban ou emanatè yo, ‘dans un premier temps,’lè sa mwen te wè enpòtans li wi men,m pat akòde l twòp enpòtans pase sa okontrè mwen gen HM38 ki sa vwazin mwen lè m t’ap pale de emanatè yo avèk li, li di m wi yo chase marengwen mwen di bon dòz la manke,lè m te di doktè Chicoye sa, doktè Chicogne te redouble l pou nou ankò,se lè li pran li, li ale avèk li,konya prezans li vin pan na kay la menm ankò kounya m vin wè ki efè l te fè, paske lè prezans li vin pa nan kay la,moustik la vin tounen menm jan ankò ap anvayi, lè sa men m di a emanatè a gen enpòtans li,se lè pa itil pa la kòm di vye dikton kreyòl la pou wè si pa itil pat itil, alò m te vin wè ke li se on bagay ki te gran enpòtans.

ITV: Se on bèl temwanyaj, se lè pa bon pa la pou wè si pa bon pat bon.

HM36: Se lè li pote l vini on dezyèm fwa, m wè li gen plis enpòtans ke sa m te pretann.

ITV: Kiyès k’ap pale ankò? Kisa nou panse de moustik, kisa nou panse de bèt sa?

HM40: Bon, a nou pasne de bèt sa,vrèman te konn gen anpil moustik m mèt di jajn HM36 di an, kòmsi li bay maladi, li mete mikwòb nan san ou, li pa bon,depi lè emanatè a antre nan kay la, se pa menm jan,nou swiv menm mouch li pouse se sak fè mwen felisite nou pou sa.

ITV: Oke, kiyès k’ap pale? Kisa nou panse de moustik? Moun ki poko pale nou mèt pale.

HM55: Sa nou panse de moustik, moustik la se on vye bèt li ye, kòman m te ka di sa lè ou met on bagay sou tab, li plede vin poze sou li, bagay sa yo, plede koloe sou moun,pu byen di, mwen menm m panse emanatè a, se solisyon pou nou,ki kwape tout moustik yo, emanatè a vrèman bon,bon travay.

ITV: Nou pral poze on kesyon, nou reponn deja wi, men m’ap repoze l ankò, kisa nou te konn fè, mi mezi nou te konn pran pou nou pa kite moustik mòde nou. E pou nou touye moustik yo tou.

HM36: Mezi ke nou te konn pran pou moustik pa mòde nou,se jan ke m te di nou talè a, e gen on ti krèm tou wi, yo konn vann pou marengwen, M bliye non li, men m konn on ti jan pè pase l paske gen moun ki konn di yo alèji ak li, e m wè tou yo pa vle yo pase l sou timoun sa konn fè m on ti jan pè paske ou konen bagay ou ap pase sou kò ou, se bagay ki frajil,m konn pè pou l pa gen efè segondè, avèk nan vye koutim ayisyen lè mearengwen ap mòde ou twòp ou ba l de twa tap, ou fè l vole.

ITV: Kiyès k’ap reponn ankò? Kisa nou te konn fè pou nou chase yo, touye yo.

HM40: M konn tèlman genyen yo,m konn pouse yo a rad, gen de lè m frape menm sou yo, m touye yo,lè yo kole nan tout mi an,gen delè yo konn sa l mi an tou,son bèt ki trè degoutan,m gen on ti piti mwen gen delè, yo konn tèlman mòde l, li konn anpeche m dòmi, lè konsa m pouse avèk rad, pou m ka evakye yo.

ITV: Dakò HM40, kiyès ankò k’ap pale?

HM54: Gen on bagay yo konn, li mache a kouran ou konn mete l lè ou konekte li.

Assistan: Rakèt?

HM54: Oke, depi ou konekte l marengwen an la, l’ap bagay marengwen an,avèk lè fini tou, yo konn fè nou fè flam, pou pouse marengwen an, flam nan konn pouse marengwen an tou, ebyen se konsa nou te konn itilize lè nou pot ko gen emanatè a,men emanatè a vrèman bon,nan ki san sm;ap di ou li vrèman bon, menm ti ravèt piti li ekate.

ITV: Dakò, a kisa ou te konn fè flam nan?

HM54: Lafimen, kòmsi ou gendwa chache ti pay, ou fè lafimen an, li prale, ou konprann?

ITV: Oke, e ou madam?

HM38: M te knn achte stop moustik, krèm m konn pase l de lè konsa m konn fè on nwit ap peche marengwen sou moun bat moun, fè yo sote nan dòmi, epi kou emanatè a vini, tout bagay kanpe, pa gen bagay sa ankò.

ITV: Nou di on pakèt bagay, nou sot bay ide pou nou chase, kouri dèyè epi touye moustik,alò bagay sa yo, ki limit bagay sa yo? Ki limit teknik sa yo? Tankou plagatòks, tankou fè lafimen, tankou rakèt èske bagay sa yo gen on limit? Ki limit bagay sa yo? Èske yo te vrèman efikas anpil anpil? Di kèk bagay sou limit yo.

HM36: Limit bagay sa yo ke nou te konn itilize a, plagatòks la se pat on bagay ki te gen on garanti a san pousan, dayè se on bagay, ou limen l, li fè sann, pou li fini, sètadi ke,’à une certaine durée de la nuit, lè ou limen l l’ap fi i kanmenm, li p’ap janm ka rete pou jiska deman maten, e kou l fini,moustik la, li retounen ankò, ki vle di si ou vle pou l at fà tout nwit lan, pou l’ap ba ou on ti sèvi, fòk ou oblije leve o kou nwit lan pou al relimen on lòt ankò e se achte pou ou achte plagatòks la, dè fwa ou gendwa pa menm gen kòb la pou ou achte l tou, tout sa konn rive,ou pa konprannn, bon avèk emanatè an ki se on bagay ke y oba ou gratis, fò m presize sa tou,m jije li pi bon, pi pi nesesè, yo ba ou l gratis ou pa pran okenn odè, ou pa fè anyen, depi ou jis depose afè ou on kote epi ou ap jwenn sèvis la, m’ap di mèsi tou pou sa.

ITV: Dakò, limit mwayen nou te konn itilize pou pwoteje nou yo, kiyès k’ap pale ankò? Pa pè pale.

HM55: Pou mwen, m p’ap ba ou manti, a san pou san, m pi renmen emanatè a, paske plagatòks la lè ou limen l dè fwa men odè a ou konn pa ka pran mwen menm m son moun dè fwa menm mwen menm m pa ka pran sant li lè l limen, li fè m anvi toufe,avèk ankò pou moustikè a tojou, m konn mete l dèfwa, lè ou mete l, lè kò ou pase ladan l, paske ou konnen li gen on bagay ladan l li grate o, li boule ou, pou mwen menm m wè emanatè a se tout afè, tout bagay bon,tout bagay nòmal, m prefere emanatè a.

ITV: Kiyès ankò k’ap di on bagay?

HM40: Emanatè a vrèman bon, sak fè m di li vrèman bon, tankou ou gendwa gen moso rad la nan men ou ou pouse moustik la epi apre on titan ankò, ou wè l retounen li vini,se konsa ou konprann, sa vle di m trè kontan emanatè a, emanatè a fè anpil bagay, li pouse moustik anpil, anpil anndan kay la, nou pa wè yo jan nou te konn wè yo a, sa vle di emanatè a ban ou anpil sekirite anndan kay la.

ITV: Oke, si gen on moun k’ap di on lòt bagay, on lòt kesyon m’ap poze paske nou gentan bay repons li deja men m’ap poze kesyon an kanmenm pou nou te ka bay plis repons. Ki avantaj nou jwenn nan emanatè a? Ki avantaj emanatè a bay pa rapò avèk lòt mwayen nou te konn itilize pou nou chase oubyen touye moustik? Ki avantaj nou jwenn nan emanatè a?

HM54: Se menm bagay lan wi, avantaj ke nou jwenn ladan li jis li pouse marengwen yo pou nou, ke nou avèk on moso twa lap pouse osinon n’ap achte plagatòks la, epui n’ap fè lafimen an, epi limenm li kote nou an,nou itiliz eli epi li pouse marengwen an,non mwen m gendwa ap lave m mete l bò kote m, e sèl lè konsa m ka itilize l plis,bon m te menm di doktè a konsa,èske m pa ka ale avèk youn nan travay mwen an, tèlman kote m ye a gen marengwen li di non, l’ap fè swivi an pou mwen pou konnen si m kab ale avè l.

ITV: Se on bèl temwayej, menm lè ou redi bagay lan,ou mete on lòt bagay.

HM54: Wi, m te poze l kesyon de sa, li di m l’ap fè swivi an pou mwen pou l konnen si m ka deplase al nan travay la ansanm avè l poutèt marengwen, ou konprann men li trè itil san repwòch.

ITV: Kiyès moun ankò, k’ap di ki avantaj, ki benefis nou jwenn nan emanatè a?

HM36: Emanatè a li vrèman gen anpil avantaj ladan l, kòm m sot di ou talè a, paske on bagay ou mete ki ap ba ou sèvis ki pa degaje oken odè, ou pa pran oken odè ladan l, ki pa, li pa vrèman on bagay tou ki pran on gwo espas,nenpòt ti kote tou piti ou depoze l li bon, li jis ba ou sèvis ou bezwen an, bon sa se yon gwo avantaj kanmenm puiske li chase moustik pou ou, ki se yon trè bòn choz on bagay k’ap chase moustik, k’ap anpeche moustik mòde ou, se gratis, kanmenm se on gwo avantaj.

ITV: Se gratis, kanmenm se on gwo avantaj.Kilès ankò ki ap di on bagay?

HM36: Ou di se gratis, gen on lè li p’ap gratis ankò m ta renmen konnen?

ITV: Bon, li ka komèsyalize,li p’ap chè men li ka pa gratis la toutan m sipoze, pa gen k’ap pale ankò? Nou vini pou pale, fòk ou pa pè pale paske temwanyaj la enpòtan, fòk ou pa ale ak li nan tèt ou. Ki pwoblèm emanatè a konn bay? Ki pwoblèm nou te jwenn nna itilizasyon emanatè a? Nou te di bagay pozitif men si te gen, si emanatè a te bay n pwoblèm lakay ou, ou te viv sa temwanye sou sa.

HM36: Bon, m pa wè ki pwoblèm li te bay, m pa ka di ou ki pwoblèm li te bay, paske m pat wè l te bay pwoblèm sinon ke m te di doktè Chicoye lè l te pote yon sèl grenn an m te di l ke mwen te santi li two fèb. Sa sèlman apresa li te di li t’ap double dòz la ladan l, li te double l,M te wè li te trè byen, m pa wè oken pwoblèm ke li bay. M pa ka di ou ayen de sa.

ITV: Bon, men ou pale, li sensè, sa li di a se sa, ki pwoblèm emanatè a te bay lakay nou, lè nou t’ap itilize l?

HM55: Pou mwen menm m wè emanatè a pat bay pyès pwoblèm okontrè emanatè ede nou, paske lèou ap dòmi leswa, lè marengwen ap mòde ou, ou konn ap plede leve, paske marengwen konn ap fè ou leve nan dòmi, pa rapò de emanatè a, tout moun dòmi byen nèt ale, li pat bay pyès pwoblèm.

ITV: Oke, bèl temwanyaj.

HM54: Premye fwa lè l te fenk vini an,ti mesye yo te di m konsa key o pa ka pran odè a, m di a, ou konnen medikaman ki ladan l nan, n’ap fè on ti pasyans po nou wè si odè a p’ap desann men peape yo kòmanse abitye avè l, li pa gen pwoblèm ankò. Gen delè lè youn pa jwenn pou l dòmi se on pawòl anpil sa di ou se avè l’ap dòmi, kounya yo vin twò renmen l pase m, pa gen kenn pwoblèm.

HM38: Emanatè a pa banm oken pwoblèm, lie de m anpil, men li pa banm pwoblèm.

HM40: Lè aparèy la te fèk vini m te wè l nan men HM41 m’ap mande sa sa ye, se lè a l’ap esplike m men, m te panse se travay li ki ba li aparèy la, mwen m pat konn anyen de li, de kisa sa ye, se lè sa l’ap esplike m li di m konsa piga m manyen tout kote emanatè a, paske gen kèk kote ki pa pou mwen ladan l, paske m pat konnen se lè s al’ap esplike mwen kòman sa ye, men emanatè a, nou pa gen anyen mal, nou ka di de li, li trè byen, li trè pwoteje n anfen li gaye moustik yo pou nou, nou toujou rete n’ap swiv, n ap swiv toujou pou nu wè ki plis sa l’ap ban ou men nou pok ka di anyen mal de li.

ITV: Dakò, ki lòt avantaj nu jwenn nan emanatè a? A pa lefèt ke li chase moustik, ki lòt benefis nou jwenn nan aparèy sa?

HM40: Nou jwenn anpil benefis paske, nou konn gen moustik tout nan lakou a, tout anndan nou pa prèske wè sa ankò, sa vle di emanatè a limenm li fè sa li kapab nou npokom ka di anyen de mal de emanatè a.

HM54: Emanatè a pa repwòch ditou, li fè sa l kapab.

ITV: Li pwoteje nu kont moustik, ki limit nou wè? Èske lè l te fèk vini li te pi efikas? Èske avèk tan efikasite a diminye? Èske li te touju bon malgre li te pase anpil tan lakay nou? M bezwen temwanyaj sou sa.

HM40: Wi, li diminye paske anvan te gen anpil anpil, li plizoumwen mwens nou pat konn tèlman gen twòp marengwen.

ITV: Èske fikasite a diminye, sa vle di èske nan yon premye tan li te chase, èske li toujnou chase moustik malgre li te fè anpil tan lakay nou?

HM40: Wi, li toujou chase moustik paske m te konn wè yo an vòg apresa, m pat prèske wè yo ankò.

HM36: Pou mwen menm efikasite l pa diminye non, m wè l toujou bay menm kantite sèvis li t’ap bay la.

ITV: Kiyès k’ap temwanye sou sa? Èske nou wè efikasite a redui?

HM55: Li toujou pouse marengwen menm jan, li pa diminye.

ITV: Èske gen on moun ankò k’ap di on bagay? Èske emanatè a lè l vini li chase moustik? Èske avèk tan li chase moustik mwens, èske li toujou fò?

Le groupe: Li toujou fò.

HM36: Li toujou efikas, li tounjou chase marengwen menm jan ak premye jou a.

ITV: M pral nan dènye pwen sa la, kisa ki fè nou te aksepte itilize emanatè a lakay nou?

HM36: Se doktè a kite vini, ki te prezante nou pwojè a, bon m te jis ap eseye, m te jis pran on chans, doktè akonvenk nou, li montre nou pa a plis b, men kòman, men kòman itilizasyon an ap bon pou nou, li ap benefik alò m te jis pran on chans, mwen te asepte itilize li, se jis doktè a ki te konvenk nou. Nou men tou nou te wè, nou te gen anpil moustik tou, sitou du coté de kanapevè, ou konnen la gen anpil ravin, anpil moustik la, gen anpil moustik lè doktè a te vini avèk pwojè a pou nou, m te wè se te yon bèl bagay. M te jis aksepte, m te itilize l, se tou.

ITV: Dakò, kiyès k’ap reponn ankò? Kisa ki fè nou te akspete itilize emanatè a?

HM54: Bon, se on jou maten nou te chita, epi doktè a vin parèt l’ap pale ansanm avèk nou, men li pot ko vini ak emanatè a non. Li t’ap pale de sa ansanm avè nou, epi nou di n’ap antre nan pwojè a, nou bay non noue pi nimezo telefòn no, epi on lè li pote l pou nou, nou menm nou pran emanatè a nou itilize l, men li te bon pou nou, lè l te mete m nan pwojè a, pwojè te byen bon paske m pa wè marengwen, li pouse marengwen yo vrèman pou nou. Se konsa nou vin antre nan pwojè a, pwojè a byen bon, li byen itil nou.

ITV: Èske gen on moun k’ap di on lòt bagay ankò sou emanatè a? Pa gen anyen ki te anpeche nou itilize emanatè a? Èske te gen retisans? O depa èske nou te mete l nan kwen? Èske gen on bagay ki te anpeche nou itilize emanatè?

HM54: Pat gen anyen ki te anpeche m itilize l, mwen men m te itilize l nenpòt kote, m gendwa pral kouche atè a la epi m annik pran l m mete bò kote m, m sou kabann nan m mete l bò kote m, m’ap lave m mete l bò kote m, m’ap lave veso m mete l bò kote m paske se konsa doktè a te di nou pou n itilize l, pou n te ka wè ki efè li fè. E vrèman m vin wè ki efè li fè.

ITV: Bèl temwanyaj, kiyès k’ap pale ankò?

HM36: Kòman nou te sèvi ak emanatè a, se jan doktè a esplike nou an, tout sa n’ap fè nou mete l bò kote nou, paske nan tout sa ou ap fè vre, depi ou ap fè on bagay ou oblije ap tape,yo toujou ap mòde ou, mwen gen on timoun piti tou, li toujou ap kriye leswa moustik ap mòde l, moustik ap mòde l, kounya sa pa fèt ankò.Tout s aou ap fè ou met l bò kote ou,ou mete l bò kabann nan, ou gendwa ap lave veso a la,konya ou pran l ou mete l bò kote ou, chase moustik yo,vrèman vre ou pa santi l, a la minit ke ou pran emanatè a, ou mete l bò kote ou, yo p’ap mòde ou menm jan, ou p’ap santi l, li chase yo trè byen se on bon bagay, m’ap di mèsi ankò, se on bon bagay.

ITV: Èske gen on moun la ki te pè emanatè a? Èske n te di a èske m ka itilize bagay sa? Èske te gen te retisans?

HM55: Wi, lè emanatè a te fèk vini,m pat panse si l t’ap bon konsa, paske m wè menm mouch li pouse mouch. Paske okontrè m te konn ap di konsa poukisa doktè a pote bagay sa? Pou mwen menm m pasne li patap itil konsa, jan m di sa,paske li pouse ni moustik, ni mouch, m wè li fè toulede travay sa yo, m wè li bon.

HM38: Avan lè mesye a te vini avèk pwojè a, se pandan ke mwen bò lakay mwen m wè l’ap pale avèk medam yo, HM36 epi HM55, avèk HM54 epi manman m vin rele m, manman m di konsa, men gen on mesye ki genyen, on doktè wi ki vin avèk on pwojè,vin ekri non m, kounya m kouri, m’ale vre epi m mande doktè Chicoye, kòman pwosesis la ap ye kounya la, li esplike m kòman l’ap ye, bagay aprèsa mwen dakò antre ladan lmen lè li vin vini ansanm avè lm pat pran sa mal, m te jis pran l mwen mete l nan on kwen lakay lan paske se atè mwen dòmi avèk tibebe mwen genyen, pitit mwen epi mwen mete l, m konn leve nan nwit vrèman, m jwenn marengwen sou li, paske li pa vle kouvri, men depi lè emanatè a vini mwen mete l bò kote l epi li dòmi nèt al kole li pa menm fè kri menm lannwit li dòmi, apresa lakay la te gen anpil ti ravèt piti mwen wè li kouri dèyè yo, li trè bon.

ITV: Nou ap pran on poz la, e apre dejene an, n’ap diskite sou twazyèm tèm nan.

[POZ]

ITV: Nou nan dènye pati fokis gwoup la, nou konn itilize emanatè a,nou konn itilize l plizyè fason, nan tout fason sa yo, kijan nou wè, kisa n wè ki te pi bon nan tout fason nou te konn itilize l, ki meyè fason ou ka itilize l emanatè a?

HM36: Tankou meyè fason m wè on moun ka itilize l se lè ou ap fè on bagay epi ou mete l pwòch ou, paske tank li pi pwòch ou, li bay plis sèvis tankou pa egzanp lè ou ap lave veso, ou pran l ou mete l bò kote ou, ou gendwa ap fè manje epi nou pran l, nou mete tou pre nou, nou ap dòmi nou pran l nou mete l tou pre kabann nou, tout sa, se meyè fason yo, tank li pi pwòch ou, li ba ou plis sèvis.

ITV: Kijan nou konn itilize emanatè a nou wè ki bon?

HM54: Mwen itilize l lè m’ap dòmi, mwen itilize l lè m’ap lave, mwen itilize l lè m’ap fè manje, osinon m gendwa fè on ti kouche atè a, epi m mete l bò kote m nan, tout sa se itilizasyon ke m’ap fè, men lè mete l atè abò kote m nan, m dòmi, mouch pa anmède m, marengwen pa anmède m,

ITV: Kijan ou konn itilize emanatè a ki bon? Ki bon pratik itilizasyn, on fason ou konn itilize ou wè l vrèman bon.

HM40: Lè li pi pwòch nou, nou jwenn plis avantaj, e marengwen yo on ti jan evakye nou, li trè bon.

HM38: Lè l pi pwòch mwen, li pi bon, se sa m t’ap di.

ITV: Eseye imajine nan tout fason nou konn itilize l, tankou la nou reponn byen. Bay tout fason nou kapab itilize emanatè a.

HM55: Tout fason nou kapab itilize emanatè a, m panse menm lè m’ap dòmi li ka bò kote m, lè m’ap fè manje li ka bò kote m, Paske gen on foto m fè se manje m t’a fè, li bò kote m, m panse menm lè m ap lave tou li ka bò kote m.

ITV: Ou te fè foto?

HM55: Wi m te fè foto, kote m t’ap fè manje.

ITV: Èske tout moun la te fè foto avèk emanatè a?

Le groupe: Wi.

ITV2: Nou ka kontinye pale wi, ki fason nou itilize emanatè a nou wè ki bon pou nou?

HM40: Nou itilize l tout jan, lè nou chita, lè nou kouche, n’ap lave, fè, manje, nou deplase l san pwoblèm. Lè n’ap bay blag nou mete l bò kote nou. Nou wè li trè bon.

ITV2: Oke, ki fason nou itilize emanatè a nou wè ki pa bon? Talè a nou sot pale de fason nou itilize l ki bon yo, kounya di ki fason nou itilize l kip a twò kòrèk?

HM36: Bon, fason nou wè kip a two kòrèk tankou, lè n’ap itilize l fò n pran prekonsyon, pa mete l kote ki gen dlo, kote ki sal, ou jis chèche on kote ki pwòp, kote moun p’ap pede touche ladan l, ou konnen nòmalman pwodui a si moun ap plede frape l, touche l, manyen l, poud la prale. Li p’ap gen menm efikasite a, ou oblije manyen l nan pati fè a, ou mete l epi ou itilize l. Men pa gen fason kip a bon non, tout fason ki ta vin pa bon an la, se ou k’ap fè l pa bon, ki pou pran on maksimòm de prekosyon avèk li.

ITV: Èske gen moun la ki panse yo konn mal itilize emanatè a nan on premye tan?

HM40: Wi, paske m pat konnen l, mwen pat konnen l ditou, se lè m vini m ap mande esplikasyon, lè l te fèk vini an, m pat konn valè l, m pat konn si l te gen tout valè sa,ou konprann se ofieamezi, lè nou kòmanse ap itilize l,n’ap wè vrèman ki valè li genyen,men emanatè vrèman bon.

ITV: Dakò, èske gen enkonvenyan li reprezante? Èske gen pwoblèm li konn bay?

HM40: Non, sa vle si tankou si ou gen timoun, tankou mwen menm, m gen de timoun piti, gen de lè si se pa on kote ou mete l, ou oblije mete l nan on kwen, ou konprann paske timoun nan la, li ka al ladan l,li ka jwe li ka pike, ou oblije itilize l, gen on jan pou itilize l, ou konprann?

ITV: Trè byen, èske ou konn pike ladan l?

HM40: Non, gen delè m gendwa ap fè travay nan tèt cho, se lè l te fèk vini an, m pot ko konnen kòman li ye, men li pa bay twò gwo pwoblèm.

ITV2: Pa gen lòt moun k’ap ajoute anyen? Nou pat konn mal itilize l?

HM56: Non, m pa t konn mal itilize l,depi yo ban mwen l lan, m mete l nan tèt kabann nan, m di pral eseye pou m wè kòman li ye, m pral dòmi li bò kote m. Bn m gendwa ap soti deyò a, m pral tande mizik, m tou pran ti afè m m soti deyò a, m mete l kote m nan m’al tande mizik.

ITV: Trè byen

HM38: M pat konn mal itilize l, depi lè yo ban mwen l lan, m itilize l byen.

ITV: Daprè nu menm èske emanatè a gen limit? Ki limit li daprè nou menm?

HM56: Nou pa limite l, paske tout kote nou prale, omwen sèl sa, nou gendwa pral soti nou ka pa kenbe l nan men nou pou nou soti avè l, men depi se lakay nou ye, nou nan lakou, nou itilize l nenpòt kote nan lakou a, sa vle di nou wè l pa gen limit.

HM36: Bon mwen nan sans m konprann limit lan, lè ou ap itilize li, nan espas ou ap itilize l la, li bay sèvis, li p’ap kapab si ou mete l nan on gran lakou,li p’ap ka rive chase marengwen nan tout gwo, gwo lakou a, men pa egzanp si ou nan on chanm, yon chanm kay, ou mete l nan chanm nan li ap detui marengwen ki nan espas pa l la,nan espas chanm li ye a, pa egzanp nu tout la nou fè on ti sèk n’ap konvèse, li pap kite marenwgen vini kote nou ye a, l’ap pwoteje nou kanmenm.

ITV: M konprann. N’ap kontinye, kounyala nou gen on lòt kesyon, ki ide nou genyen pout a bay pou ta amelyore emanatè a? Ide pout a fè li vin pi bon, pi efikas.

HM38: Ide m genyen pou l vin pi efikas se mete plis pwodui ladan l.

ITV: Ide pou amelyre emanatè a, kiyès ki ap pale toujou?

HM54: Bon, sa m te ka di, tank n’ap itilize l nou menm nou ta wè kòman l ye epi pou doktè a ta toujou gade l pou nou, pou l wè,si gen bagay ki manke pou l ta plis ogmante l pou l te ka pi efikas, men li efikas wi,m pa di li pa efikas non, men ou konnen tank ou ap itilize l l ap abitye, n ap mete plis medikaman pou l ka vin pi efikas.

ITV: Dakò, e oumenm madanm, ki lòt ki ide ou ka bay pou emanatè a vin pi bon?

HM55: Pou emanatè a ta pi bon,daprè mwen menm m ta renmen konnne èske yo pa ta ka mete lòt pwodui ladan l ki pou anpeche ravèt ak lòt tibèt ki genyen yo? Tankou pinèz èske y opa ta ka mete pwodui ladan pou chase jan de bèt sa yo?

ITV: Dakò, men èske ou remake emanatè a chase ravèt?

HM55: Pou ravèt la nn, pou mouch lan wi.

ITV: Ebyen n’ap kontinye, kounyaya, n’ap bay ide pou emanatè a ka vini nan tout fwaye yo, kay tout moun, pou tout moun ka genyen l lakay yo pou itilize l, kòman yo te ka fè sa?

HM54: Ide m te ka bay doktè yo, k’ap mete medikaman nan emanatè yo, jis kòmsi si espas sa gran ou konnen li p’ap ka touye marengwen nan tout espas yo,m ta swete nou ta mete medikaman ladan l, si kòmsi nou tout chita la konsa nan on sal de klas,osinon on kote epi pou l te ka tiye tout marengwen ki nan espas kote n chita paske li pa ka tiye marenwegn nna gran espas kòmsi pou lakou an gran pou li rale marengwen an, li pakab.

ITV: Mikroespas.

HM54: Wi, petèt on chanm kay de chanm kay li ka bagay li men,li p’ap ka fèl nan gran espas se ide sa senpleman m te ka bay.

ITV: Sa se ide pou amelyore l, trè byen.

HM36: Reprann kesyon an.

ITV: Ide pou emanatè a ka vin nan tout fwaye, lakay tout moun.

HM36: Ide m te kapab bay pou l ta vini nan tout fwaye, mwen konnen li p’ap fasil pou ap bay yon bagay gratui konsa nan tout fwaye,ou pa konprann, la vin oblije gen on patisipasyon men lè se te on frè moun nan ta bay, nou pa di nou t’ap fè moun nna peye a pri ke li vo a, men m panse a on vil pri, a on pri ki pa two elve nou te kapab mache ofri moun li,fè moun ki fè eksperyans avèk emanatè a deja, al ofri moun li, al konvenk moun yo, pou chak moun omwen te ka achte youn,te ka achte de met lakay yo, m wè se t’ap trè itil,ou pa konprann m jwenn li gratis men kòm li fèk kòmanse men m panse ofieamezi, li kapab vin komèsyalize, pou moun k’ap itlize l deja, mache ofri moun li,konvenk moun nan, di l men ki avantaj li gen ladan l, men sa ou ap jwen lè ou itilize li, epi pou chak fwaye te kapab achte l youn ou de ou twa emanatè se sa m wè.

ITV: Bon ide.

HM40: Reprann kesyon an pou mwen stp.

ITV: Ide pou emanatè a vin nan tout fwaye,lakay tout moun.

HM40: M t’ap trè kontan paske gen plizyè moun ki bezwen antre nan pwojè a, sa t’ap trè efikas pou ou paske yo santi pwojè a trè efikas, ou konprann, se sa sèlman nou t’ap di.

ITV: Kounya n’ap bay ide pou jere l, pou pran swen l.Paske lè ou gen on bagay ou ap itilize fòk ou pran swen li.

HM40: Wi, m pran swen li wi paske kòmsi mari m nan on tèt cho li ye lè li konn kite li, li konn chita avèk li, li vire li kite l,mwen men m pase men m pran yo,m mete yo nan on kwen,oubyen m gendwa pran yo, m mete l kote m nan,paske yo rele l la li bliye l, se lè l vini ‘o kote bagay la Minouche m di an m te antre yo pou ou frè. Se mwen ki toujou ap ranmase.

ITV: Ide pou jere l, pou swaye l, kiyès ki gen ide ankò?

HM54: Tout ide nou genyen pou n jere l jis nou pa kite lip ou timoun kraze l,paske li itil nou,nou jere l, se sèl ide sa senpleman.

ITV: Ide pou jere l.

HM55: Daprè mwen menm sa m pasne de emanatè a, yo te sipoze mete on ti bagay ladan l tankou ou gendwa kwoke l nna nenpòt kote ou pa ta bezwen chak kou nan deplase l, ou gendwa kwoke l on kote anndan kay la, yo te sipoze mete on bagay ladan, apresa nap pwoteje emanatè apaske nou gentan konnen nou bezwen l, l’ap itit nou.

ITV: Kounya nou pral fini, nou pral fè on ti pale avèk HM36 sou foto sa yo, di nou nan ki kontèks ou te fè foto sa yo? Kisa foto sa yo vle di pou ou? Ki sans yo genyen pou ou?

HM36: Mwen vle pale nan kad foto yo, foto sa yo gen anpil sans pou mwen paske se pa imajinasyon ke mwen kreye, ou pa konprann se reyalite ke yo ye,nan fason ke nou itilize emanatè yo, sa sa madmwazèl sa la ki se HM38 ki t’ap etidye, li te mete emanatè a bò kote li, jis pou marengwen te ka pa vin anpeche l nan sa l’ap fè a epi m te tou pran l an foto, mwen gen la se on jenòm ki rete lakay la mi t’ap dòmi epi pou moustik pa nwi li, li mete emanatè yo bò kote li,pou ka dòmi, ou ka wè la se on moun, ki ap dòmi nan tout lapè li, nan tout trankilite li paske li pa gen moustik k’ap anpeche li, sa se fason ke mwen mete emanatè a bò kabann nan epi mwen fè foto a, la mwen gen sa, foto sa se HM55 ki t’ap fè manje li mete emanatè a bò kote li epi ou kapab wè sa, se, manje li t’ap fè li mete emanatè a bò kote li epi sa se mwen ki t’ap etidye m mete emanatè a bò kote mwen pou moustik pa vin nwi m, sa se on moun ki malad, o kontrè li chita, li fè n ti chita la,epi mwne mete emanatè a bò kote l pou moustik pa vin nwi l, sa se devan pòt la, mwen t’ap fè travay epi vwazen sa vin kanpe m tou pran l an foto, li kanpe bò kot emanatè a, tou sa ki vle di mwen itilize li nna divès fason diferan sa son moun ankò ki t ap dòmi mwen mete emanatè a bò kote l pou l ka dòmi, pou marengwen pa nwi li, ki vle di se on bagay mwen itilize, mwen kapab di mwen itilize emanatè a nab tout sa ke m’ap fè li patisipe avèk tout s ake m’ap fè lakay mwen, mwen pran on foto souvni de yo menm. Ki vle di lè m pral amche alvann emanatè lòt semèn nan, m prale ak foto yo, m’ap konvenk kliyan yo, m’ap di kliyan yo men kòman m itilize l tou.

ITV: Lè emanatè a tounen on pwodui komèsyal, ou ap déjà on ajan maketin.

HM36: Ebyen, mèsi anpil pou patisipasyon nou sa fè mwn plezi, tout moun la te motive, tout moun reponn.

# PV-FGD Men Blocks 5-6

Date: May 2019

ITV: Mwen kontan avèk nou apremidi an, nou pral fè pn bagy ki rele gwoup diskisyon’ photo voice’nou te fè anpil photo kòm nou se gwo fotograf kounya chak moun pral pale de istwa foto sa yo, n’ap kòmanse avèk HM42 dabò, kisa foto sa oy reprezante pou ou? Bay istwa foto sa yo, ki sans yo genyen, ann ale.

HM42: Foto sa yo ou kapab gade la, mwen fè l se pandan l’ap etidye, le pli souvan m gen on kote m etidye ak pitit pa mwen, se sou lakou a nou ye la, nou sou lakou an, nou gen tablo, nou gen chèz, nou gen ti tab tou, sa se on pitit fi ke m genyen, sa se pitit gason m, pandan l’ap etidye n’ap gade youn nan emanatè yo, sa se on lòt ki apwopriye bò ti pitit lan, tifi an, sa se bò ti gason an, ou wè espas sa, pa fouti gen moustik menm, li p’ap genyen,tout la ankò, ou ka jwenn kèk grenn moustik k’ap epapiye pa dèyè yo, men sifas sa p’ap genyen paske emanatè a la. Dezyèm foto sa ankò, m fè li se pandan yon dam t ap lave veso toujou sou lakou a, ke n fè l anndan,m fè l foto sa se pandan l’ap lave veso yo, epi te gen anpil moustik bò pye li paske nwa te kòmanse ap fèmen, epi m fè foto a pou li. Twazyèm foto sa, se on gran frè m kite pase wè m, epi pandan li te an bout pantalon men pou m byen di ou mwen menm m abite nan rejyon sa se anlè kay la, limenm lè l vini se anlè li fè desant, se pandan li te anlè a, li chita, epi li di m monkonpè m gen anpil moustik wi la k’ap nwi m, epi m di banm vin met on bagay bò pye ou, epi m pran emanatè a ke m mete bò pye l, epi m fè foto an pou li, epi apre li di m vrèman li efikas, paske yo te anpil bò pye l, li pa wè yo ankò, sa ankò toujou nan menm fonksyon de tip la, se etidye l’ap etidye la, sa ankò, se chita m chita bò lari an, on ti biznis epi piske li te fèmen li pat ko ouvri, biznis la pot ko ouvri, m chita, yo vrèman kouvri m, epi m pran emanatè a m mete bò pye m la, yo ale, se de menm ke sa se on ti gran moun, ki rele bòs Jilbè, se on faktè bòlèt ki devan l’ ap vann bòlèt, epi m wè yo bò pye l paske li pa renmen mete gran pantalon, m’al pran l m di banm rann ou on sèvis m mete emanatè a bò pye l, apre li evakye yo.

ITV: Se byen sa, men foto sa yo raple ou ke emanatè a pwoteje oumenm avèk lòt moun sa yo kont moustik.

HM42: Se sak fè mwen menm fò m tounen jwenn yo, pi vit ke posib, paske em chagren de yo.

ITV: Dakò kounya n’ap pran moun sa.

HM52: kesyon an se kòman?

ITV: Se fot say o, ou ap bay istwa foto yo,ki lè ou te fè l,kisa yo reprezante pou ou? Ki sans li genyen pou ou? Poukisa ou te fè l? Ki kote ou te fè l?

HM52: Nòmalman foto sa yo m te fè yo, m te gentan resevwa on lòd doktè a te di l’ap gen bezwen seyans foto sa yo, pou yo ka pote nan laboratwa, m di pa gen pwoblèm, li di fòk nou fè foto, mwen m se on nèg m toujou ap panse, m di bon, si pou m fè foto yo,mwen an fonksyon jan moun yo ap fonksyone lakay la, m’ap fè foto yo konsa, kounya la, o moman sè m nan ap fè neve m nan etidye, m di bon bon m chwazi fè foto a pou li, pandan l’ap etidye, efektivman ou ap gade, pandan l’ap etidye vrèman ou wè yo chita chita sou kabann nan, mwen fè foto a pou yo, e se trè byen sa, lè m gade m wè, m di kèt mesye lè fin fè foto a, m’ap gade m wè sa, foto byen parèt,mwen kontan paske, oke mwen di la m gentan fini avèk on priz toujou nan foto yo, kounya m pran dezyèm sa, se matant li ki la ansanm avè l, men gen on ti jwèt ou fòme l an pwason, men malgre sa nou pa janm kite emanatè a, toujou ap sèvi, ou wè kote m te mete l, nan premye foto an, ou wè l, mwen mete l anlè sa vle di pandan menm l’ap montre l leson emanatè a menm ap fè travay li, l’ap repouse, marengwen an.

ITV: Trè byen.

HM52: La dezyèm foto sa, emanatè a yo kote, yo antoure neve m nan, limenm l’ap fòme pwason an,matant lan la l’ap asiste tou mwen menm kameramann m’ap fè foto a pou yo, twazyèmman, pandan l’ap dòmi, se reyèlman li t’ap dòmi, m mete emanatè a bò kote li paske m te wè kèk grenn marengwen k’ap epapiye nan zòn nan, m di bon, m te gen youn nan chanm nan youn devan, m pran sa sou galri an, m di bon m mete sa bò kote l paske l ka evakye marengwen yo, kounya m mete l, ou wè kote mesye yo ap dòmi,m mete li, se trè byen sa, katriyèm foto sa, se manman m, di chak apremidi se sou do kay la mwen menm ak patnè m konn vin jwe, ki plen tikwa,kounya manman m di chak jou m wè nou anlè a, jodya fò m goute ti kabann nan tou ,li di m wè ou mache a tout aparèy ou,m di wi paske marengwen ap anmède moun,ap nui moun, nui sante moun, m pa vle kèk marengwen sot kote li sòti, sitou gen on ravin kote nou an, ou abitye wè ravin nan ki pase a, pou marengwen pa sot kote li sòti pou l vin pike nou, m kwè m sou senkyèm foto la, foto sa se tinyès mwen an ki vini li di tonton prêt em emanatè yo non, m di o bon emanatè yo la,ou di prete m ou li, m di:sa ou pral fè, li di m: m pral etidye, m di li pran li,li twouve youn twò piti, li mande m de, li pran tou le de li kite m anba poukont mwen san anyen li pran li, li mete youn a kote, li mete youn anlè devan sou ban nou wè li, kote li mete li, li chita sou ban, chak apremidi anlè a nou toujou ye,konsa jan ou wè a, n’ap jwe ti jwèt nou, youn a goch, youn a dwat, sa nou nan sizyèm foto a,sa se mesye m yo,ou ap wè menm sou konfrè a yo te parèt,paske nèg yo konnen menm mezi ou wè ki nan pa HM42 la, sa se toujou bòs J, li toujou ap vann ti bòlèt mobil li, mesye sa yo ou wè k’ap jwe domino an la, gen kèk nèg ki gen pantalon long,ki kèk nèg kip a gen pantalon long, kounya misye di: kot ti aparèy ou a? M di li la wi, li di prete m lin non papa atan done ke m la marengwen yo ap nui m la, m di pa gen pwoblèm, m al lakay la, m pran youn nan emanatè yo, m mete l bò kote, mesye yo pou yo ka wè efikasite l tou,men malgre sa,nèg yo twouve, yo di yo wè l repouse marengwen an vre jan, men nèg yo mande fò l te bay plis randman, fò l te ka touye yo, pou yo pa tounen vin pike yo la, oke m sou setyèm foto a, apresa m ranvèse l, m mete lòt kote, gen de twa mesye ki chita la, m gen on patnè ki chita la marengwen ap mòde misye nan ponyèt li la,ti le pè a la ansanm avèk nou ap distribye domino a toujou, n’ap jwe la, epi m vire l lòt sans,pou m montre nèg yo efikasite paske eli p’ap nui, yo t’ap nui ponyèt misye, kounya li pa wè l tèlman ankò, lepè di, li pa aple de marengwen menm anyen li pa bay, sa vle di misye ki santi marengwen ap nui ponyèt li, kounya m jis mete l depi lè m fin mete l la, marengwen pa nui ponyèt li sa vle di pwodui ki ladan repouse li chase marengwen yo, sak fè m mande plis efikasite, pou lè l pral jwenn vwazinaj yo, lòt vwazen, vwazin yo pou l ka, pou wè lè m ap pale yo de emanatè a, pou yo wè, se pa on ti aparèy piti ki la.

ITV: Dakò.

HM52: uityèm foto, sa se ti nyès mwen an la ki la ankò k’ap penn on tablo, kounya li menm li pa fonksyone a on tablo limenm, a on sèl emanatè, a de emanatè li fonksyone,si l’ap fè on bagay la, li pa vle marengwen mòde li menm, paske li alèji avè l depi li mòde l, menm mwen menm depi li pike m la, ou wè l fè on ti boul, li fè on ti glòb, limenm li pa dakò bagay sa yo, ou ap wè a tou le de li fonksyone la, men li sa se tablo a l’ap fè a,on dam ki gen on lalin anndan vant li, on solèy anndan vant li, se li menm l’ap fè la, ou wè m met l kote li, sou kapèt la men li,m mete yo, sa vle emanatè yo,enstale limenm l’ap fè tablo a l’ap penn tablo l, sa se dizyèm foto sa, vwazinaj sa k’ap lave la,li toupre a, li konn abitye vin kote m nan jaden an l’ap fè on lesiv,kounya li te wè m te mete l bò kote misye yo, li di èske ou panse li repouse marengwen yo vre, paske gen on pakèt marengwen ka’ mòd em la, èske ou ka pwouve m sa, m di o byen si ke m ka pwouve ou sa,m vini m mete youn bò kote l la, li lave avèk trankilite, sof marengwen pa mòde li, li repouse li, men chodyè prete pa kwit pwa sèch,m patap kite l bò kote li, m pran afè m m’al lakay mwen avè l apresa, kan m fin fè foto a, m pran afè m m’al lakay mwen, ebyen se sa ki esplike dizyèm foto a.

ITV: Oke, mèsi.

HM43: Ou ap gade premye foto a, la se sou on mach eskalye an desandan lakay la, m chita la, kòm dabitid, lè apremidi, m toujou ap fè ti nouvèl mwen, avèk telefòn mwen, ou gade la telefòn nan pa nan men m paske pitit gason m nan ki gentan pran telefòn nan men m, ou wè m pran aparèy mwen m mete l,bò kote m nan ala, pou moustik pa mòde m, e sa se premye foto a la, ou ap gade dezyèm nan sa se madanm mwen k ap dòmi, mwen pran li m mete l bò tèt li, ou konprann, pou marengwen pa mòde l, twazyèm nan se dòmi l’ap dòmi toujou, paske depi l’ap dòmi li toujou mande l ti aparèy la ou konprann mwen mete l la, kounya m te mete l bò isi kounya m chanje l pozisyon, m mete l konsa.

ITV: Madanm ou?

HM43: Wi, pou m pwoteje madanm mwen kont moustik, la ou ap gade la, ou pa wè kivèt la parèt se on ti lesiv m t’ap fè, m t’ap fwote kèk tirad la epi, ou ap wè m se toujou sou mach la, men se desandan mach la,epi m chita sou ti kote a, la se tablo ou ap gade, m te ekri kèlke bagay pou tigason m nan etidye, epi pandan tou mwen m’ap fè on lòt travay,m mete l la, epi kounya la, m chita la, se de twa ti rad ke m ap fwote epi m pran aparèy mwen m mete l bò kote m nan la, la ou ap gade se pitit gason m la ki sot lekòl, pandan li sot lekòl la li mande l paa kot aparèy la kounya li chita, la se kay on vwazinaj li chita la, di: a ki bagay prete ou aparèy sa la, li di papa prete m li pandan m chita la m poko pral retire rad sou mwen, epi li chita la, li fè foto a la,la ou ap gade ankò, la se mwen ki chita la toujou, se on lòt ti bagay ke m t’ap fè sou, lesiv toujou, m akoupi kòm la, epi la se bò mi yo, la se de bò mi, bò lakay la, m di a si m pat mete l nan pozisyon sa, m’ap mete l pozisyon sa pito, epi m chita la, kounya la m’ap fè ti lesiv mwen, se menm kalaite foto, la ou ap gade, tigason m nan la,li sou kabann nan la, se on liv ki nan men l, men valiz li epi kounya la, m mte aparèy la bò kote l pou l kabab etidye.

ITV: Pwoteje fanmi an.

HM43: Wi, pou l pwoteje fanmi an, l’ap ou ap gade se mwen menm ki chita la,avèk tifi m nan, pitit fi m nan, m chita ansanm avè l la, epi m mete aparèy la, l’ap kapab pwoteje li,pou moustik pa mòde l, la se tigason m nan, sa se uityèm foto a,se etidye misye ap etidye la,men la se deyò a l’ap etidye, la pou ap gade pwent mayo sa, se pwent mayo pa tifi m nan m mete l la, l’ap etidye, li di: papa pandan m’ap etidye a vin pran aparèy la mete bò kote m la epi kounya m tou fè foto a la, m fè foto a pandna misye ap etidye a la, ou ap gade dizyèm foto a toujou,ou wè se do m ki parèt men se etidye misye ap etidye do m ki parèt la, m ap fè l etidye,la te kòmanse gen farinay lapli kounya m rantre anndan la, men liv la la epi misye ap etidye.

ITV: Trè byen.

HM41: Ou ap wè fot sa, tankou talè a m t’ap pale m di m prete vwazinaj aparèy la, sa se mesye a, li sou mach eskalye lakay li, men li kanpe l’ap fè on nonb de tan paske ou konnen se on atis li ye, li toujou ap fè ouvraj, li di prete m aparèy ou a, m’ale m pran l m ba li l, pandna m kanpe a, m di jan ou kanpe a banm tou pran on poz foto, ou ap gade sa, sa se pòt ki pa anba lakay la, kounya la m prete l li, men se premye fwa li pral wè eksperyans emanatè a paske li pot ko sèvi avè l,se lè m fin ba li l, li di o li bon wi menm tou kite l nan men m non, m di l non m pa ka kite l nna men ou, m ka fè demach pou jwenn youn,se sak fè ou wè m di ou yo pa vle kite pa m yo lakay la,fò n ban mwen pou m ba yo, dezyèm fot sa, sa se on lòt ajan sekirite parèy mwen vin lakay mwen pandan l anndan, m t’ap bwè ti gwòg mwen epi m mete emanatè a epi m tou fè foto a.

ITV: Oke

HM41: Sa se twzyèm foto a, sa se anndan lakay la m mete l, epi m annik fè foto jan l ye anndan m di pou yo ka wè l, se sa k fè talè a,m te di m gen j3, m gen j6 la, m gen de emanatè.

ITV: Oke.

HM41: Sa se katriyèm foto a, nou chita nan dèyè on kamyonèt n’ap pale, n’ap pataje ide, misye nan telefòn, mwen m mete emanatè a dèyè kamyonèt la paske te gen moustik ki te nan dèyè kamyonèt la, on kamyonèt ki pa sèvi, m chita ladan l pou n ka pran detant nou, sa se anndan lakay la anba on pandri m te mete emanatè a epi m fè foto a, m di, m’ap kontwole pou moustik paske yo toujou di la yo toujou gen moustik, m met emanatè a anba l epi m fè foto a, sa se nan on lakou,on lòt moun pote emanatè a men nan on lakou anfas kote m te fè ou vire a, bò kay mè a, m prete yo emanatè a, men misye toujou di anndan lakay la gen moustik, m prete l li, epi m fè l kanpe, m fè foto a konsa, sa se on mekanisyen k’ap ranje machin, lè l’ap ranje machin nan lè a gentan rive sou li, chak l’ap bagay li moustik anpeche l bagay li, m’ale m prete l emanatè a, epi pou l ka rete pou l fin ranje machin nan.

ITV: Oke HM41, n’ap pase a HM37 kounya.

HM37: M se HM37, jan n’ap gade foto sa la, sa se pandan ke nou chita nan salon an, m santi marengwen ap nwi m, m pran l mete bò kote m, m fè foto a.

HM37: Sa se on dezyèm foto ke n’ap gade,mwen chita menm kote a tou, m pran emanatè a, m mete l bò kote m.

ITV: Sa se lakay ou?

HM37: Lakay mwen, marengwen ap mòde m, m mete l bò kote m, twazyèm fot sa, se on tidam ki la, k’ap dòmi, men mwen fè foto a li pa konnen.

ITV: Dakò. Lakay ou?

HM37: Lakay mwen, m santi marengwen ap anmède l, m fè l san l pa konnen.

ITV: Dakò.

HM37: Sa se manman m ki anndan, li chita, m wè li gen marengwen bò kote l la, m mete l bò kote l m fè foto a.

ITV: Dakò, se te pou pwoteje l.

HM37: Sa se mwen ki kouche, m pran l, m mete l bò kote m, mwen fè yo fè foto a pou mwen, m santi marengwen t’ap anmède m. Foto sa m pa konnen l.

ITV: Se pa pou ou?

HM37: Non.

ITV: Ebyen yo fè on erè.

HM41: Sa se doktè a ki te fè l pou X, li gen emanatè nan men l tou.

ITV: Se li ki pa vini an?

HM37: Sa se pandan ke nou nan chanm, misye ap gad televizyon, l’ap pran plezi, l’ap gad ti komik, m met emanatè a bò kote l, m fè foto a.

ITV: Kiyès moun sa?

HM37: On ti kouzen m.

ITV: Ki lakay ou?

HM37: Ki lakay mwen. Sa se on ti patnè m gen lakay la, yo renmen emanatè a pou jan li repouse marengwen, kounya misye pral kale on bagay, misye di m prete l li pou m mete l bò kote m nan, ou ap gade ou wè l ò kot misye a.

ITV: Oke.

HM37: Sa se on lòt tou, ki t’al fè manje, ki di m prete l li, pou l mete bò kote m nan.

ITV: Dakò, ebyen nou prèske fini la, paske nou prezante foto yo, istwa yo, kisa yo reprezante pou nou, kilè n te fè yo, poukisa nou te fè yo,se trè bye, m’ap poze kèk kesyon. Èske foto sa yo raple nou ke emanatè a pwoteje nou kont moustik?

Le groupe: Wi, li raple nou.

ITV: Paske chak fwa nou fè foto sa yo, se montre nou montre nou ap konbat moustik avèk emanatè a. Èsek foto sa to montre nou ke gen avantaj emanatè a ban ou?

HM41: Wi, paske li pouse moustik, li bay avantaj, paske li pouse moustik, nan sitiyasyon ke ou te ye, oparavan ou pa ladan l, menm si li pas an pou san men ou jwenn on senkant pousan la.

HM37: Foto sa yo montre m anpil avantaj emanatè a ban mwen, paske mwen menm, nòmalman si gen on mon ki chita, marengwen ap nwi l ou prete l li, pa gen marengwen ankò, si ou chita ou ap gad televizyon, li bò kote ou, ou pa wè marengwen vini ankò, se avantaj sa m wè emanatè a bay.

ITV: Trè byen HM37, kiyès k’ap pale ankò?

HM43: Avantaj ke li ban mwen, ou imajine,madanm mwen k’ap dòmi m mete l bò tèt li, pou marengwen pa mòde li ankò, si se pat sa, li te ka nwi l, fè l pa ka dòmi,men pa rapò de aparèy la, marengwen pa mòde l, de menm pou tigason m nan ou wè l’ap etidye sa n kòmsi tanzantan l’ap nwi l, e lè sa, li te ka kontrarye l, li pote avantaj pou mwen.

ITV: Trè byen HM43. Kiyès ki ap pale ankò?

HM52: Foto sa yo, yo vrèman konte pou mwen, paske se sa, a lèd de priz foto sa yo, ki pwouve tout travay ke emanatè a fè, tout efikasite li, se limenm pa rapò avè l, ki fè jounen jodya, nou vin ak foto yo, pou n montre nan diferant ang, men kòman nou te konn itilize li, nou abitye avè l.

ITV: Oke, èske,nou te konn fè move foto tou? paske la nou moutre bon foto. Èske nou te konn fè foto ki pa bon?

HM52: M fè plizyè foto nan ti aparèy la, mesye a konstate yop, vin wè vrèman vre yo vrèman bon, nou pa ka fè move foto ladan l, m pa konnen, nou pa fè move foto. E si ta egn on move foto nou t’ap wè l tou, pa gen move foto, nou fè bon bagay pou lè moun yo al gade, pou yo vrèman ke nou te mete n o travay ansanm avè yo.

ITV: Oke HM52, chak fason, chak fwa nou itilize emanatè a on fason, nou chèche fè on foto pou sonje.

HM52: Jisteman. Sa vle di pou apwopriye ansanm avè l sitou, doktè a te di nou pral vin repale ankò sou emanatè a avèk imaj yo, fòk nou pale de yo menm, m di anben pa gen pwoblèm nou fè li nan optik sa vle di, sa n’ap di pou l apwopriye avè l, pou li idantik.

ITV: Dakò, men kisa k te pouse nou menm pou nou fè foto sa yo pou nou raple on seri de bagay nou te wè, nou te viv?

HM52: Nòmalman, le jou menm dòk la te vin kote mwen, ou te la tou jou sa, ou te wè sa, m kwè se mwen ki t ap fè pa m yo avan, mwen di bon, oke dòk pa gen pwoblèm, m’ap prepare foto yo pou ou, otomatikman, moun lakay yo,nòmalman m felisite yo, yo rete la, yo te ankouraje m tou, nan sa m’ap fè a. Si ou wè manman m, sè m,neve m, nyès mwen akonpaye m nan sa m’ap fè a paske yo konn enpòtans li itilite l tou paske nyès mwen an li menm, dabò pou li etidye fòk li gen emanatè a, aktyèlman emanatè a manke l anpil, lè l’ap etidye la, li p’ap jwenn emanatè a ankò, mèsi.

ITV: Dakò, èske foto sa yo ba nou ide pou nou ta amelyore emanatè a?

HM52: Jisteman, m ta renmen nou amelyore m repete sa a plizyè repriz, paske emanatè a li jwe on gwo wòl anndan kay la, e nou konstata l to nou menm, si n genyen l, nou itilize l, se sak fè nou fè divès kalite foto tou, nou wè,men mwen menm sa m ta vle, popilasyon ki nan zòn nan, sa vle di vwazen, vwazin, k’ap viv alentou nan zòn kote m ye a,m ta renmen yo genyen emanatè a tou, paske yo enterese ak li, paske se mwen k’ap pale yo de emanatè a, paske mesye a lè l jwenn ak nou li di men tèl jan men tèl bagay, m di oke pa gen pwoblèm, m pale yo de emanatè a, m di m ta renmen pwojè a rive sou yo tou, men pandan m ta renmen pwojè rive sou yo, m ta renmen li rive a fòs, a tout efikasite l, non sèlman se pa pou repouse marengwen sèlman, pou l detui marengwen an tou, avèk tout lòt ensèk k’ap nui moun.

ITV: Dakò HM52, èske nou te konstate, tankou sa y oba nou ide, èske foto sa yo fè nou raple emanatè a konn chase lòt bèt tou? sèlman moustik pa gen lòt bèt?

HM52: M byen di ou wi, nòmalman, si emanatè a chase lòt bèt, nou pa konnen, otomatikman nou konnen se, li la pou chase pou chase moustik, men tout ensèk ki ka frape de emanatè a ke yo frape. Men nou menm nou ka pa teni kont de sa, ou konprann sa m di ou an, e sa fè tou, nou mande plis efikasite, se pa sèl marengwen ki pou mouri, mouch lan tou, krikèt la, ravèt lan tou depi apwoche bò kote l, ke li mouri.

ITV: Dakò, èske foto sa yo raple nou avantaj emanatè a, men èske yo raple nou limit emanatè a tou?

HM41: Li montre nou efikasite emanatè a, men li pa ka montre nou limit emanatè a, mwen di sa ankò, emanatè a pa gen limit. Ou ka pran l la, ou mete l nan machin ou, ou al nan on ti pwogram jakmèl avè l, ou ka pran l la, ou mete l, ou al wadèvè kote ak li. li pa gen limit.

ITV: Oke, trè byen. Ki ide foto sa y oba nou pou yo ta distribye emanatè a kay tout moun, pou pran swen li tou, pou antreteni l?

HM41: Pa rapò, jan blòk lakay la moun yo ap fonksyone a,yo wè emanatè a, yo sanse pa janm vle kite l lakay la, mwen menm pou m sèvi avè l, paske chak fwa m montre on moun ki pot ko fè rankont ak emanatè a, lè yo mete l lakay yo, se kòmsi,y opa vle bay li ankò,se sak fè menm jan konfrè a sot di an, fòk dòz ki nan aparèy la gen matirite, dòz la ta fò ladan l, pou l detui tout ensèk avèk marengwen se touye pou l touye yo, pu l detui yo.

ITV: Dakò, èske foto sa yo raple nou nou te chèche imajine tout fason pou nou itilize emanatè a, piske nou pat menm bezwen fè foto, nou chache itilize l, anpil fason pou nou kapab eksplwate l, èke foto sa yo raple nou sa?

HM41: Wi, se sak fè pa gen on bagay ke ou ap fè,pou pa gen on souvni ladan l, se tankou jan ou wè ou fè foto sa, se sak pral di,ou gen dwa rive on kote ou di ou fè on foto pou emanatè, men fò ou ka dekri foto a, ou di tèl lè m te fè fot sa,men kote m te ye, men poukisa m te fè l, men ki mezi m te pran, men kijan emanatè a te ye, paske sa se j6 men kisa j6 te ye, annatandan li te konn evakye moustik men kounya ; li touye yo,pa rapò nou ogmante dòz la.

ITV: Ki sans foto sa yo gen pou nou? ki esplikasyon, ki enpòtans yo genyen pou nou foto sa yo?

HM41: Pou mwen menm m di foto yo gen sans, sak fè di yo gen sans, doktè a oumenm, si nou di ou nou sèvi ak emanatè a,se sak vin montre kòm prèv nou sèvi ak li, ou wè nou itilize l.

ITV: Se vre.

HM41: Si nou parèt nou dimou, nou itilize l, li bon, li bon, men toutan nou pa parèt a on prèv alamen pou n di men ki sa l fè, men ki efè l fè l, se kòmsi se lave men siye atè ou p’ap janm konnen ke nou itilize l, nou sèvi avè l.

HM52: M te vle ajoute on bagay tou, non sèlman tou, foto yo se on prèv pou montre men sa nou fè, e nou menm tou ki ap distribye emanatè yo tou, yo wè nou t’ap fè on travay sou teren, menm lè nou vin anchene avèk travay la.

HM43: Foto yo vin kòm on souvni, lè m pale de on souvni, imajine ke chak grenn foto ou fè yo, ou fè yo nan on sans, vwazin nan ki bò kote ou la,menm lè ou te gendwa ap pale l de aparèy la, li pa genyen li, men pa rapò de foto yo, ou gendwa montre l, men nan ki sans ke m itilize li, li menm l’ap di ou prete l li, e m pa ta renmen se prete pou m prete l li, m ta renmen ke chak vwazinaj ki alantu m nan, ta genyen on aparèy konsa pou yo itilize l, e jan pou yo ta genyan l lan tou, se pa a 50 pou san, men a 100 pou san, lè l ateri a pou tout moun omwen,pou yo ta di m: m te itilize l la,m wè li detui parengwen yo, paske gen anpil moun ou konnen anviwonnman yo pa bon,li sal, li gen dlo, li gen tout bagay k’ap pike timoun, e gen moun nan ki pa gen mwayen an pou l achte bagay pou l pwoteje timoun nan, men si pa egzanp, li ta vin jwenn ak aparèy sa la, li t’ap ede l anpil, se sa m t’ap di mèsi.

ITV: Ki moun k ap pale ankò sou enpòtans imaj say o, foto say o genyen?

HM37: Foto yo gen anpil sans pou nou, menm kòlèg la sot di foto yo se souvni, jan misye di l la, si ke nou di nou itilize l, nou pa gen foto pou nou montre ou, ou ka pa kwè, se youn nan rezon nou fè foto yo, e nou montre ou, ke nou itiliz el, yo gen anpil sans pou mwen.

ITV: Èske foto yo ban ou ide sou enkonvenyan emanatè a bay?

HM37: Wi, li ban ou ide.

ITV: Ki ide?

HM37: Li pa nou anpil ides ou li, tankou, m te ka di, yo pran emanatè a yo met l bò kote ou,li repouse marengwen yo,li bay lòi ide ankò.

ITV: Kisa nou t’ap di sou foto yo kòm dènye bagay avan nou chwazi dis foto ki pi bon yo?

HM52: Pou mwen sa fè m plezi, difèt ke mwen o sèvis de USAID, malgre sa nou pa gen on prèv definitivman kòmsi ki pou ta di vre nou gen on souvni, nou gen on souvni se vre sou foto a, men pou mwen ta di kèt se tèl òganizasyon wi, pou nou di sa ou wè emanatè a, se USAID ki te vini avè l, mwen pou ta di pou ou ta wè menm anblèm yo oswa on sin,on bagay kèlkonk pou ta idantifye ke nou travay avèk USAUD pou gwo travay l’ap fè paske m ta renmen travay la,pa sèlman nan blòk lakay mwen, jan ou wè li kanapevè a, nan tout lòt kote, pou moun yo fè menm ti bagay ansanm avèk nou pou nou wè ki enpòtans foto a, ki enpòtans emanatè a, se on prèv li ye pou nou se sa ke m te ka di angwo.

ITV: Mèsi pou tan sa nou bay, nou di anpil sou foto yo, nou di pou kisa nou te itilize yo, kòman nou te itilize l, nan ki kontèks,kisa sa ka raple nou, on pakèt enfòmasyon, m panse se on bon ‘photovoice’, mèsi anpil pou patisipasyon nou nan travay la. Kounya nou pral chwazi dis foto ki pi bon yo. Gwoup sa pral chwzi ansanm, epi n’ap remèt mwen yo sou plas.
